# Supplementary material for: Copper-catalyzed Z-selective synthesis of acrylamides and polyacrylamides via alkylidene ketenimines
Source: Nat Commun. 2022 Jul 27;13:4362. doi: 10.1038/s41467-022-32082-w (PMC9329291; doi:10.1038/s41467-022-32082-w)
Supplement: Supplementary file 1 — Supplementary Information [file 41467_2022_32082_MOESM1_ESM.pdf]

Supplementary Information

Copper-catalyzed *Z*-selective synthesis of acrylamides and polyacrylamides via alkylidene ketenimines

Xuelun Duan[1,3], Nan Zheng[2,3]*, Ming Li[2,3], Gongbo Liu[1,3], Xinhao Sun[1,3], Qiming Wu[1,3], Wangze Song[1,3]*

[1]State Key Laboratory of Fine Chemicals, School of Chemical Engineering, Dalian University of Technology, Dalian, 116024, China.
[2]Department of Polymer Science & Materials, School of Chemical Engineering, Dalian University of Technology, Dalian, 116024, China.
[3]Department of Pharmaceutical Science, School of Chemical Engineering, Dalian University of Technology, Dalian, 116024, China.

* nzheng@dlut.edu.cn; wzsong@dlut.edu.cn

1 Supplementary Methods....2
1.1 General remarks....2
1.2 General procedure for the preparation of substrates....2
1.3 Representative procedures for the synthesis of azides....2
1.4 Representative procedures for the synthesis of OBoc-alkynes....3
1.5 Representative procedures for the synthesis of *Z*-acrylamides....3
1.6 Representative procedures for the synthesis of polymers....4
2 Supplementary Discussion....4
2.1 Mechanistic experiments....4
2.2 Examination of other nucleophiles....13
2.3 Examination of leaving groups....13
2.4 Monomers of *di*-azides for MCP....14
2.5 Structure analysis of polymers....15
3 Supplementary Notes....16
3.1 Characterization data of products....16
3.2 NMR spectra....29
4 Supplementary References....150

1 Supplementary Methods

1.1 General remarks

Unless otherwise noted, all commercially available reagents and solvents were used without further additional purification. Thin layer chromatography was performed using precoated silica gel plates and visualized with UV light at 254 nm. Flash column chromatography was performed with silica gel (40-60μm). ^{1}H and ^{13}C nuclear magnetic resonance spectra (NMR) were obtained on Bruker Avance II 400 MHz, Bruker Avance III 500 MHz and Bruker Avance NEO 600M recorded in ppm (δ) downfield of TMS (δ=0) in $CDCl_3$ unless noted otherwise. Signal splitting patterns were described as singlet (s), doublet (d), triplet (t), quartet (q), quintet (quint), or multiplet (m), with coupling constants (*J*) in hertz (Hz). High resolution mass spectra (HRMS) were performed by an Agilent apparatus (TOF mass analyzer type) on an Electron Spray Injection (ESI) mass spectrometer. Matrix-assisted laser desorption-ionization time of flight-mass spectrometer spectra (MALDI-TOF) were obtained by Burker ultraflextreme. Melting points were determined by an XP-4 melting point apparatus. GC-MS were determined by a Thermo Fisher apparatus (Exactive GC).

1.2 General procedure for the preparation of substrates

The *di*-OBoc-alkyne **5a** were prepared according to the literature procedure.[1]

1.3 Representative procedures for the synthesis of azides

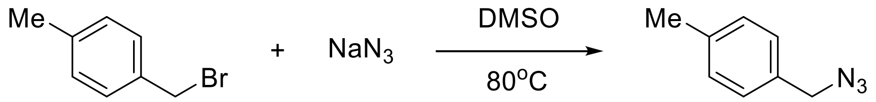

2b

The bromide (37 mg, 0.2 mmol) was added into DMSO (0.4 mL), NaN_3 (19.5 mg, 0.3 mmol) was then added, the temperature was raised to 80°C and stirred overnight. The reaction was quenched with water, extracted with ethyl acetate for three times, and washed with NaCl saturated solution to obtain **2b** as colorless liquid (26 mg, 90% yield).

1.4 Representative procedures for the synthesis of OBoc-alkynes

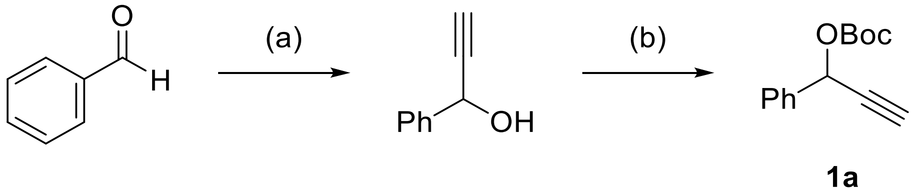

1a

a) Benzaldehyde (510 μL, 5.0 mmol, 1.0 equiv) was dissolved in a 250 mL oven-dried round-bottomed flask containing THF (35 mL), followed by the addition of ethynylmagnesium bromide (15 mL, 7.5 mmol, 1.5 equiv, 0.5 M in THF) at 0°C. The mixture was stirred at room temperature for 8 h. When the reaction was completed as determined by TLC, the mixture was quenched with a saturated aqueous NH_4Cl solution (30 mL) and then extracted with ethyl acetate (3 × 30 mL). The organic phase was washed with brine, dried over Na_2SO_4, and concentrated in vacuo. The residue was purified with flash column chromatography (ethyl acetate : petroleum ether = 1:4) to give product 1-phenylprop-2-yn-1-ol (594 mg, 90% yield) as a yellow oil.

b) *di*-*tert*-Butyl dicarbonate (1.6 mL, 6.8 mmol, 1.5 equiv) was added to a stirred solution of 1-phenylprop-2-yn-1-ol (594 mg, 4.5 mmol, 1.0 equiv) in THF (9 mL) at 0°C, followed by the addition of DMAP (55 mg, 0.45 mmol, 0.1 equiv). Then the reaction was allowed to proceed at room temperature for 12 h. When the reaction was completed as determined by TLC, the mixture was concentrated in vacuo. The residue was purified with flash column chromatography (ethyl acetate : petroleum ether = 1:5) to give product *tert*-butyl (1-phenylprop-2-yn-1-yl) carbonate **1a** (898 mg, 86% yield) as colorless oil.

1.5 Representative procedures for the synthesis of *Z*-acrylamides

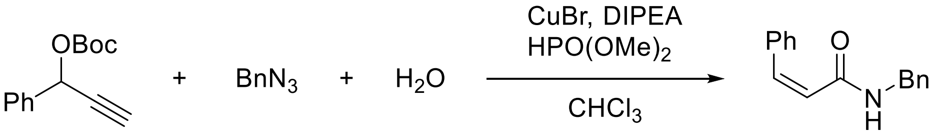

1a **2a** **3a**

OBoc-alkyne **1a** (46.4 mg, 0.2 mmol, 1.0 equiv) was added to a vial containing $CHCl_3$ (2 mL), DIPEA (66 μL, 2.0 equiv), $HPO(OMe)_2$ (2 μL, 10 mol%), H_2O (3.6 μL, 1.0 equiv), CuBr (3.8 mg, 10 mol%) and benzyl azide **2a** (37.5 μL, 1.5 equiv) were then added. The vial was closed and the mixture was stirred for 6 h at room temperature. Remove the solvent under reduced pressure. The residue was purified with flash column chromatography (33% EtOAc in petroleum ether) to give product **3a** (36 mg, 75% yield) as a yellow solid.

1.6 Representative procedures for the synthesis of polymers

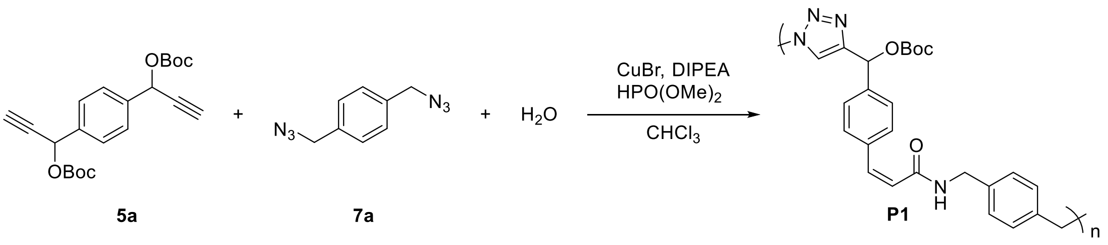

di-OBoc-alkyne **5a** (38.6 mg, 0.1 mmol, 1.0 equiv) was added to a vial containing $CHCl_3$ (2 mL), DIPEA (66 µL, 4.0 equiv), $HPO(OMe)_2$ (2 µL, 20 mol%), H_2O (3.6 µL, 2.0 equiv), CuBr (3.8 mg, 20 mol%) and *di*-benzyl azide **7a** (18.8 mg, 1.0 equiv) were then added. The vial was closed and the mixture was stirred for 12 h at room temperature. The product was precipitated using Et_2O, and collected by centrifugation. Finally, the product was dried under vacuum to obtain polymer **P1** as a brown solid. (48 mg, 83% yield).

2 Supplementary Discussion

2.1 Mechanistic experiments

The effect of water amount was systematically investigated in Supplementary Figure 1 in the Supplementary Information. When the amount of H_2O was controlled less than 5 equiv, the yield did not change significantly. In addition, the excessive amount of water could not obviously influence *Z/E* selectivity.

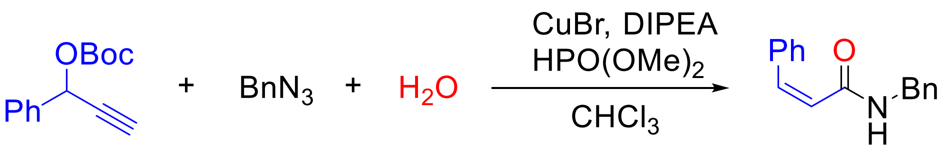

1a **2a** **3a**

Equiv	Yield(%)	*Z:E*
1	75	>19:1
2	75	>19:1
5	72	>19:1

Supplementary Figure 1. The effect of water equivalent on the reaction.

Z-acrylamides were afforded by adding water to alkylidene ketenimine intermediates. When D_2O or $H_2{}^{18}O$ were used instead of H_2O, the results were showed in Supplementary Figure 2.

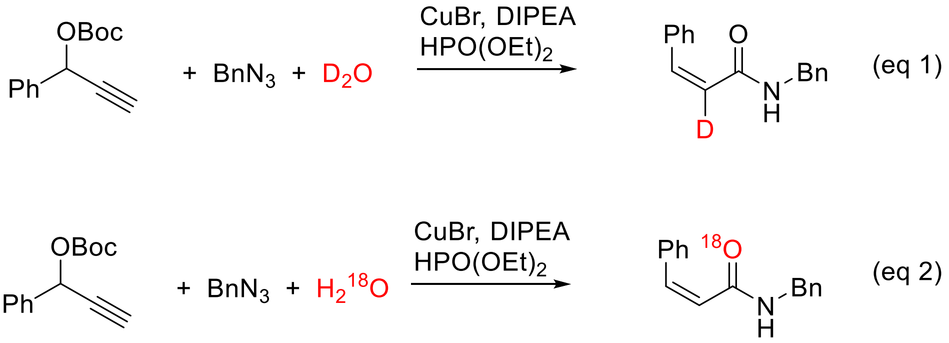

Supplementary Figure 2. The investigation for mechanism.

To further investigation the details of the mechanism, two mechanistic experiments were designed in Supplementary Figure 2. First, D_2O was used as a reactant. According to the ^{1}H NMR spectra, the hydrogen on the olefin double bond was partially replaced by deuterium (see Supplementary Figure 3). Next, heavy-oxygen water ($H_2{}^{18}O$) was used instead of H_2O, and the ^{18}O substituted product was successfully characterized by high resolution mass spectrometry (HRMS) as m/z $(M+Na)^+$ 262.1088 (see Supplementary Figure 4). It demonstrated that the water could *cis*-add to the middle double bond of **E** (see Fig. 8) to give the imino acid **3a'**, which quickly isomerized to *Z*-acrylamide compound **3a**.

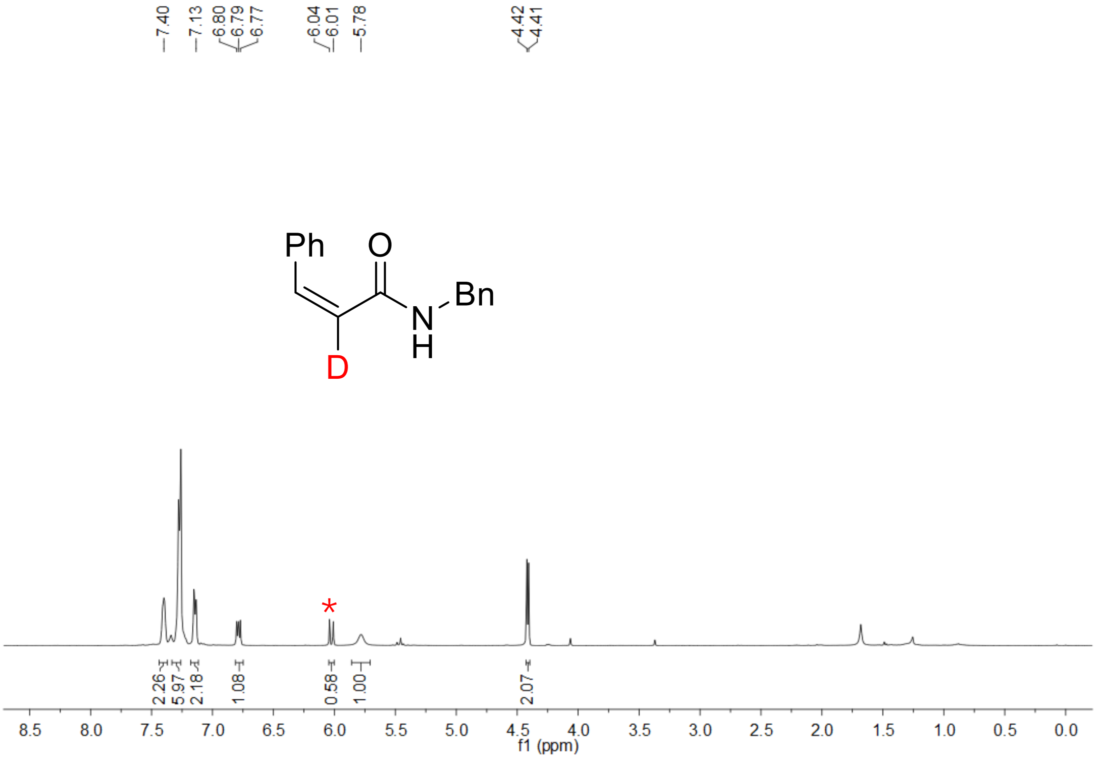

Supplementary Figure 3. ^{1}H NMR spectra of deuterium *Z*-acrylamide.

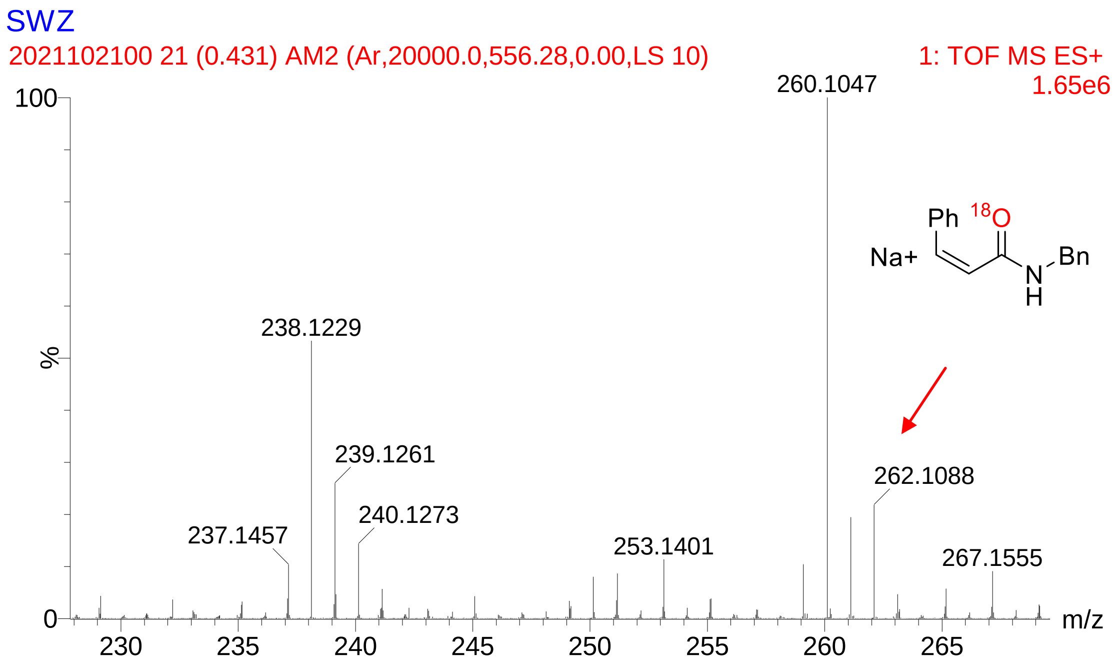

Supplementary Figure 4. HRMS of ^{18}O substituted *Z*-acrylamide.

Subsequently, we further try to trap the intermediates during *Z*-acrylamide formation by GC-MS (gas chromatography-mass spectrometry) with HRMS (Supplementary Figure 5). Fortunately, the carbene intermediate **D** ([M+2H]) was observed at m/z 249.1259 (calcd is 249.1266). More importantly, alkylidene ketenimine **E** could also be found at m/z 219.1042 (calcd is 219.1048), which undoubtedly confirmed the formation of alkylidene ketenimine intermediate.

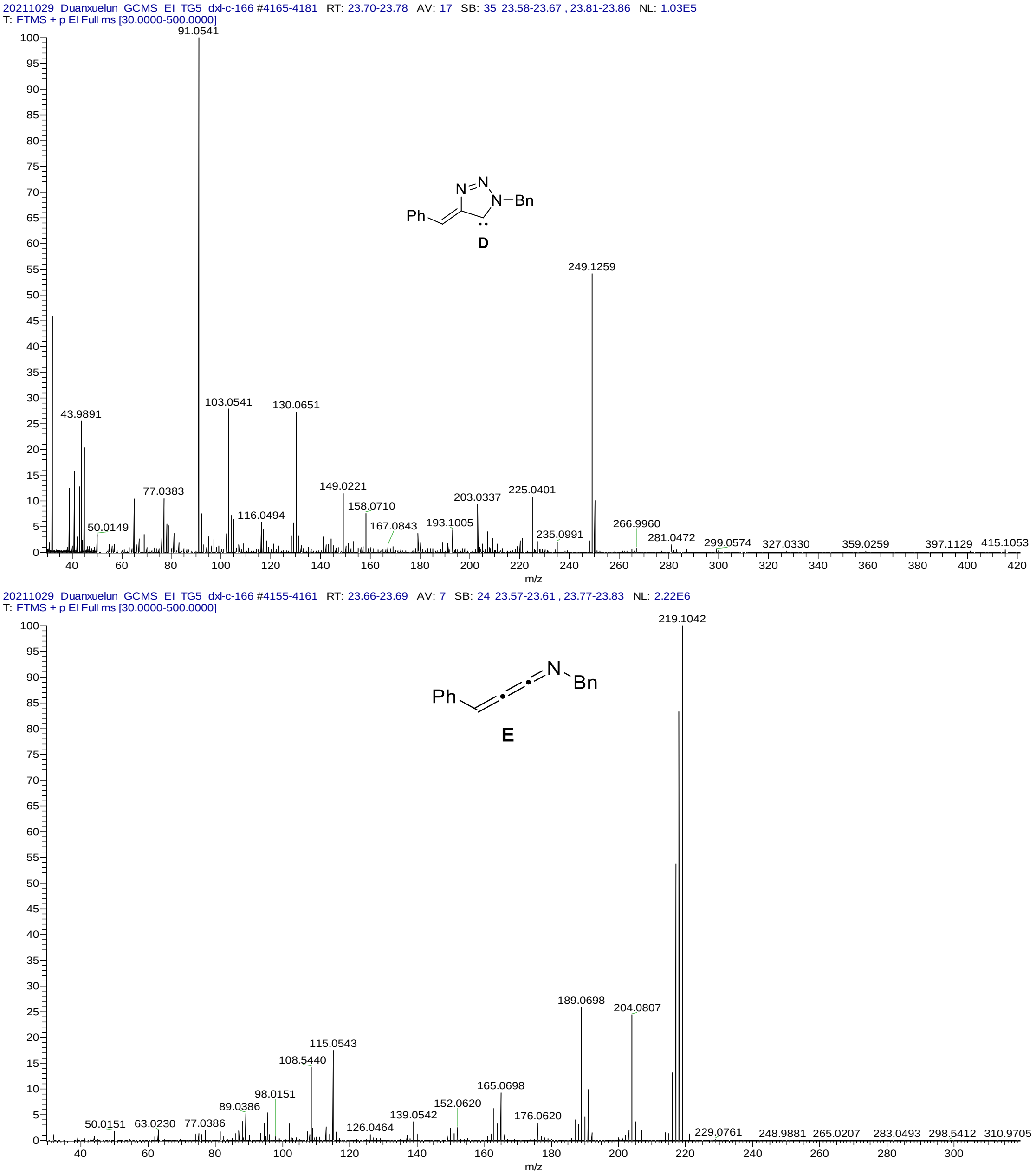

Supplementary Figure 5. Traping intermediates by GC-MS with HRMS.

Next, some control experiments were set up in Supplementary Figure 6. First, if no dimethyl phosphite was added, trace desired acrylamide was observed from crude ^{1}H-NMR spectrum (Supplementary Figure 6a). Second, if azide was removed from the system, only allenyl phosphonate was isolated instead of acrylamide. This transformation has already been reported.[2] Treating allenyl phosphonate by azide in standard reaction conditions, no any reactions occurred (Supplementary Figure 6b). Third, if alkyne was removed from system, no any reactions occurred (Supplementary Figure 6c). Finally, we prepared OBoc-triazole as substrate by other method. Treating OBoc-triazole by azide and water in standard reaction conditions, no any reactions occurred (Supplementary Figure 6d).

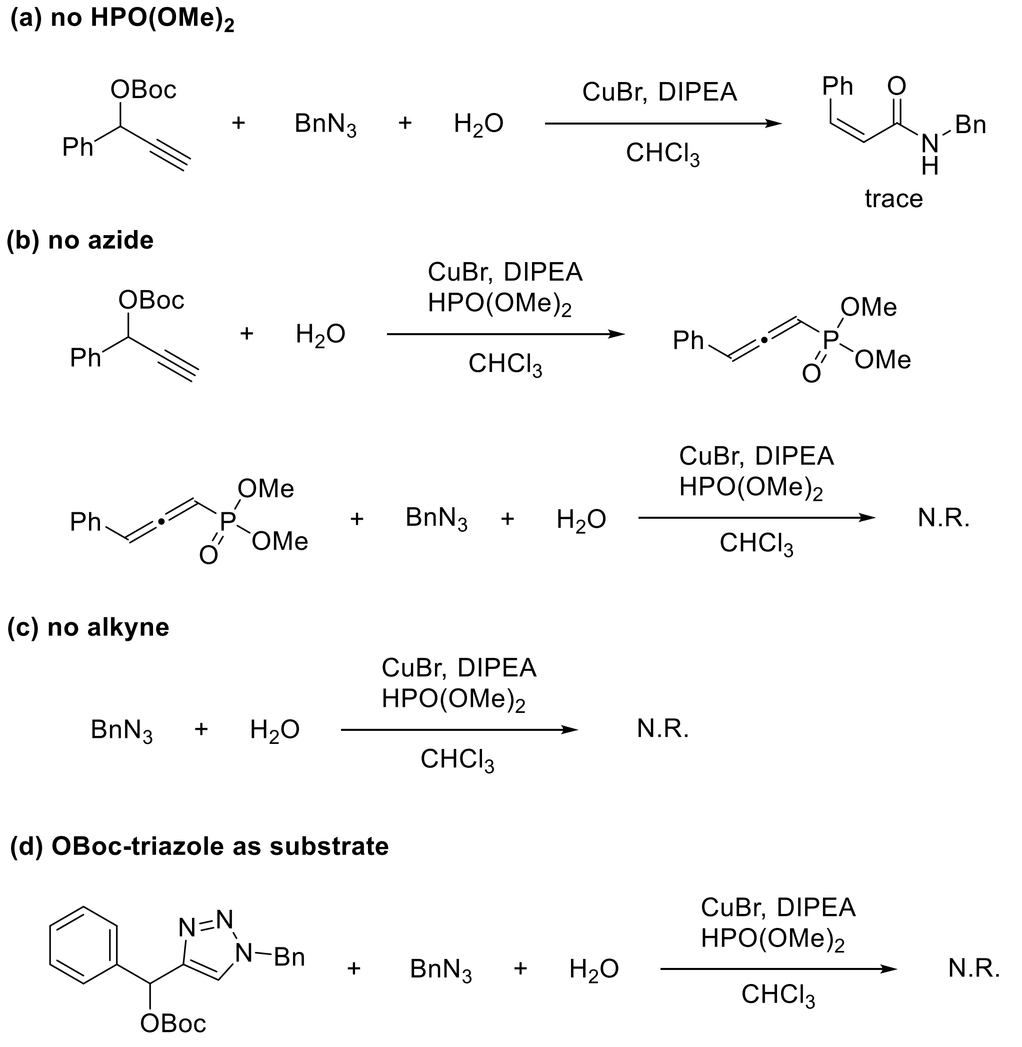

Supplementary Figure 6. The control experiments. (a) The reaction was conducted without dimethyl phosphite. (b) The reaction was conducted without azide. (c) The reaction was conducted without alkyne. (d) The reaction was conducted using OBoc-triazole as substrate.

Subsequently, after screening various phosphites, we found that only dimethyl phosphite ($HPO(OMe)_2$) and diethyl phosphite ($HPO(OEt)_2$) could promote this transformation (75% yield for dimethyl phosphite and 60% yield for diethyl phosphite). However, the yield for diisopropyl phosphite ($HPO(O^iPr)_2$) would be dramatically decreased to 30%. Therefore, we have considered that dimethyl phosphite may be as important assisted nucleophile or ligand with copper, which could promote the copper elimination from unstable intermediate **C** to generate carbene intermediate **D** as follows (Supplementary Figure 7):

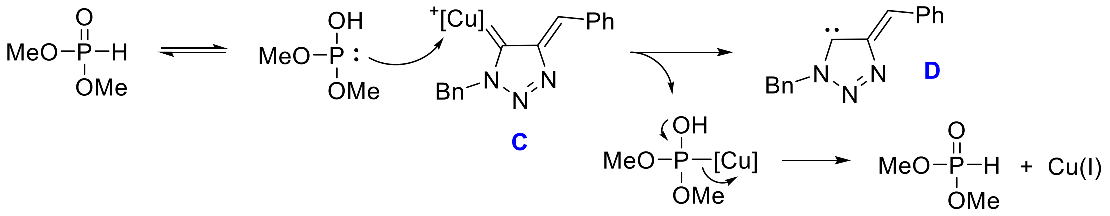

Supplementary Figure 7. The details for phosphite-assist carbene formation.

Next, ^{31}P-NMR spectra were used to monitor this transformation in Supplementary Figure 8. The chemical shift of $HPO(OMe)_2$ was δ 10.44 ppm in $CDCl_3$. After we set up the reaction in small scales in NMR tube using $CDCl_3$ as solvent, a new peak was observed in δ -17.72 ppm within 10 min. We considered that the intermediate [Cu]-$P(OH)(OMe)_2$ could be observed in -17.72 ppm. After 1 hour, we could still observed the major peaks in δ 10.44, -17.73 ppm, which could maintain unchangeably during the whole reaction process. Above ^{31}P-NMR spectra could support the proposed mechanism in Supplementary Figure 7.

(a) ^{31}P-NMR spectrum of $HPO(OMe)_2$

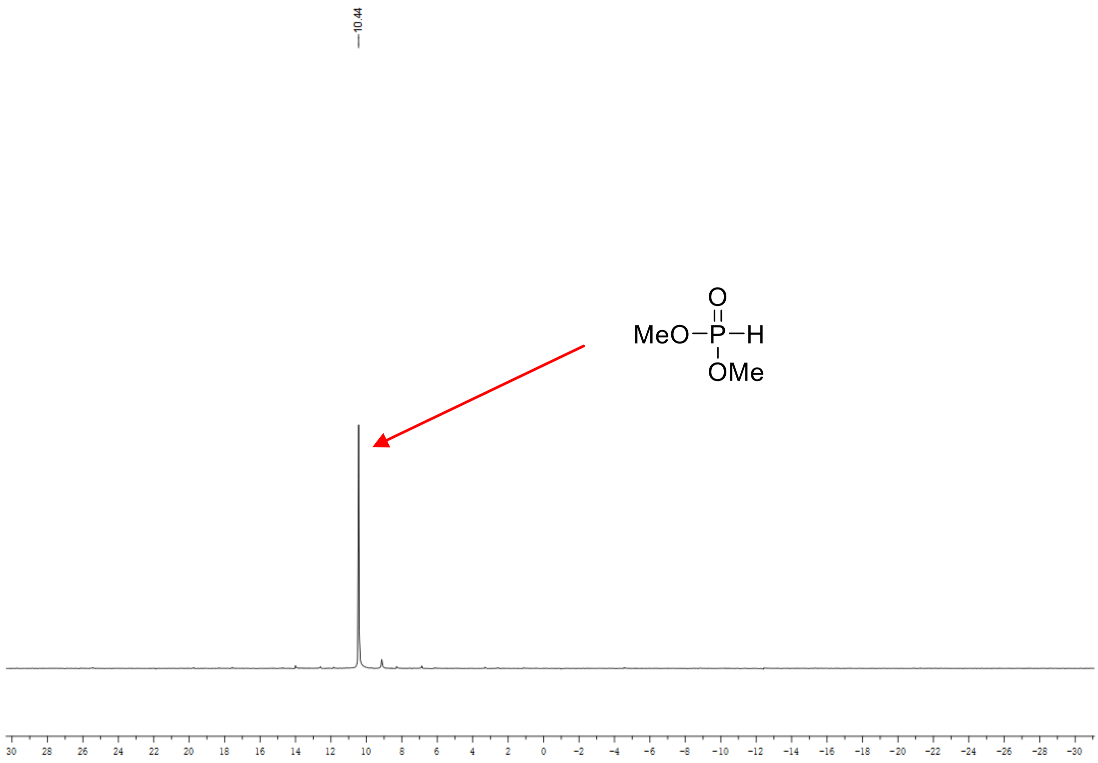

(b) ^{31}P-NMR spectrum of reaction mixture within 10 min

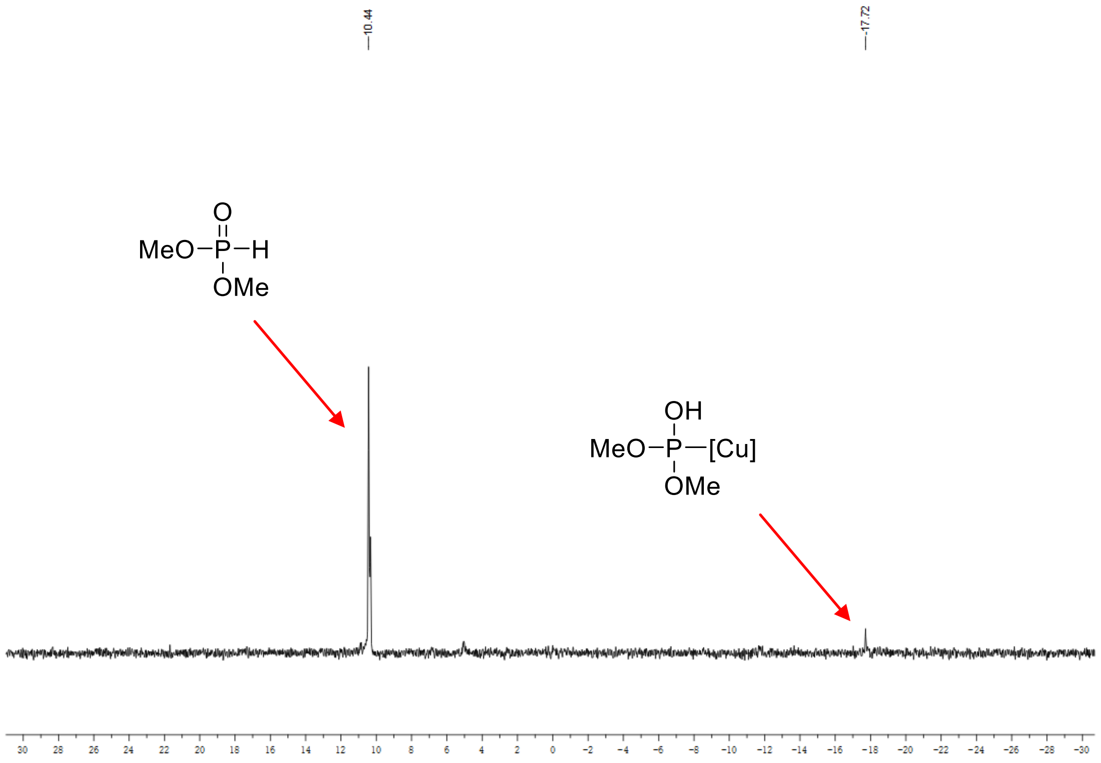

(c) ^{31}P-NMR spectrum of reaction mixture after 1 hour

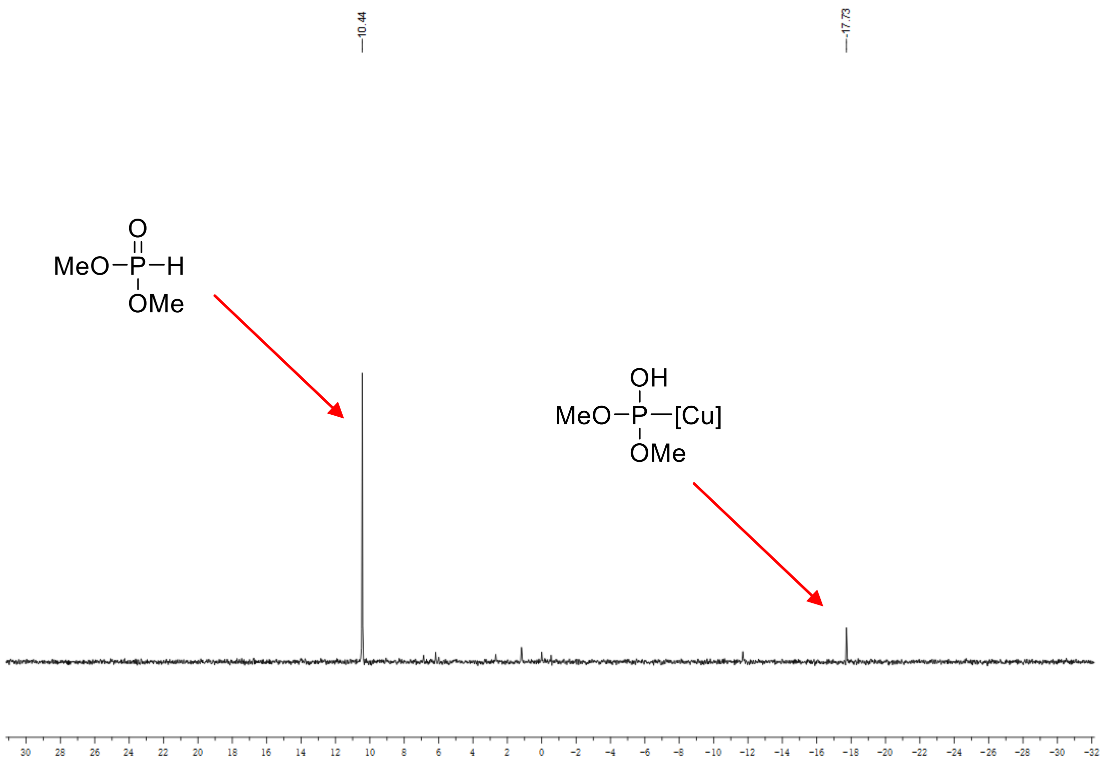

Supplementary Figure 8. ^{31}P-NMR spectra for monitoring the transformation. (a) ^{31}P-NMR spectrum of $HPO(OMe)_2$. (b) ^{31}P-NMR spectrum of reaction mixture within 10 min. (c) ^{31}P-NMR spectrum of reaction mixture after 1 hour.

Intermediate **F** could be trapped by MALDI-TOF (see Supplementary Figure 13). The *Z*-selectivity was determined in the formation of imino acid **3a'**. We have considered that the long pair from nitrogen may contribute to the formation of *Z*-selectivity by the lone pair-π interaction (see Supplementary Figure 9). We have calculated the stable configuration of **3a'** and **3a"** by density functional theory (DFT) calculations performing at the M06/6-31G(d)//B3LYP/6-31G(d)-LANL2DZ level.[3] The distance from nitrogen atom to aryl group is about 3.2 Å, which is suitable for the lone pair-π interaction. It also may be the reason why the reaction would become very messy without any selectivity using alkyl substituted groups instead of aryl groups in alkyne moieties.

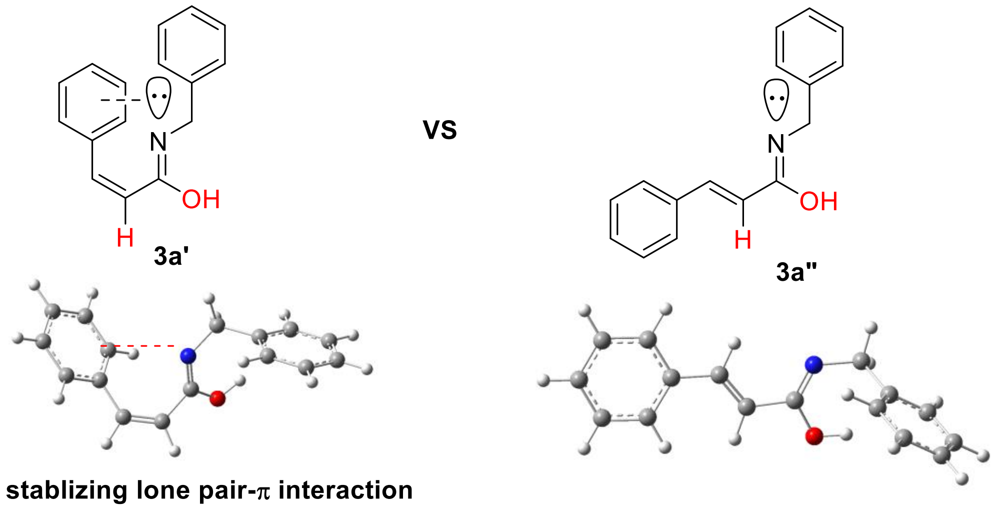

Supplementary Figure 9. The stable configurations of imino acids.

2.2 Examination of other nucleophiles

We have examined other nucleophiles such as alcohols, amines and mercaptans. However, only trace product was observed in crude ^{1}H-NMR spectrum using benzyl alcohol as nucleophile (Supplementary Figure 10a). We have tried to improve the yields under various reaction conditions but failed. When primary or secondary amines were used as nucleophiles, corresponding amino-triazoles **S1** and **S2** were obtained instead of acrylamidines due to the strong nucleophilicity of amine groups (Supplementary Figure 10b). If benzyl mercaptan was used as nucleophile, only unknown insoluble solid was acquired which was undissolved in almost all organic solvents and water (Supplementary Figure 10c).

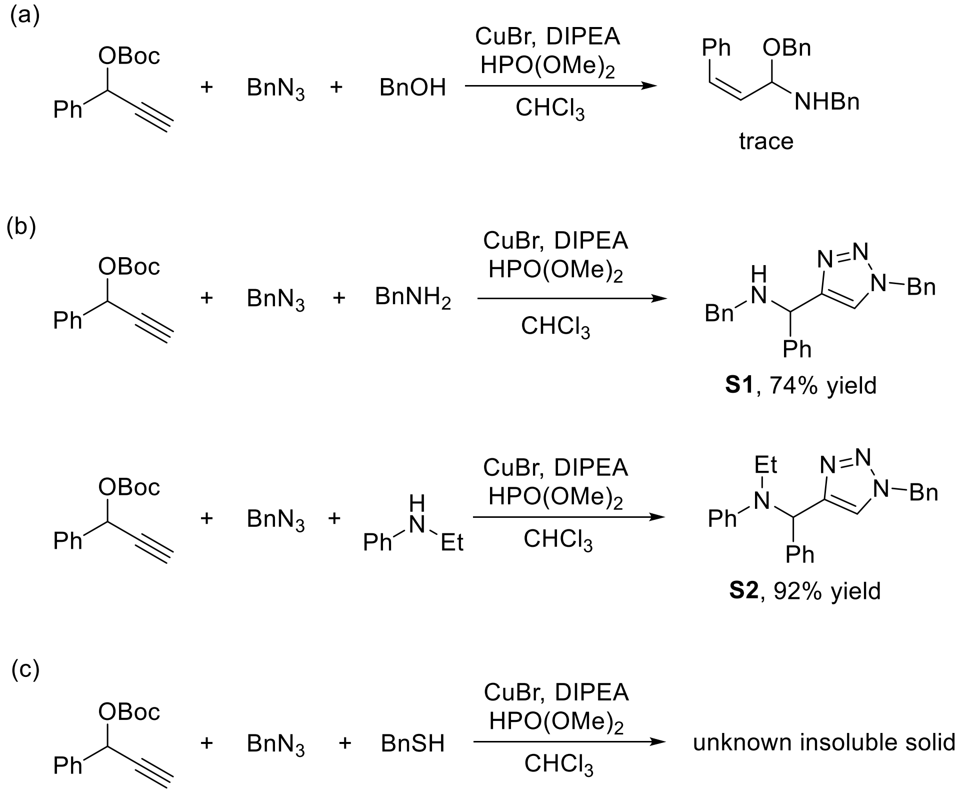

Supplementary Figure 10. The examination of other nucleophiles. (a) Benzyl alcohol as nucleophile. (b) Primary or secondary amines as nucleophiles. (c) benzyl mercaptan as nucleophile.

2.3 Examination of leaving groups

We have screened different leaving groups and Boc group was demonstrated to be necessary for this transformations. If Ac and Piv groups were used instead of Boc group, only triazoles **S3** and **S4** were afforded with 85% and 83% yields respectively instead of acrylamides under reaction conditions (Supplementary Figure 11).

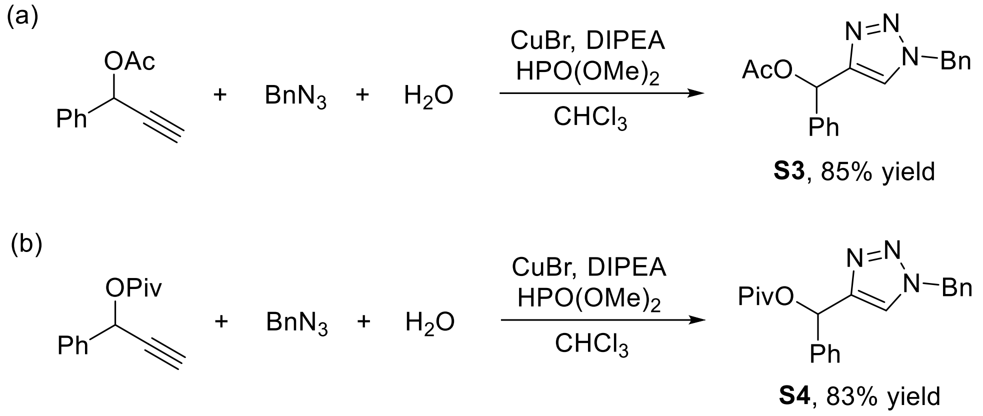

Supplementary Figure 11. The examination of leaving groups. (a) OAc as leaving group. (b) OPiv as leaving group.

2.4 Monomers of *di*-azides for MCP

The monomers of *di*-azides for MCP were showed in Supplementary Figure 12.

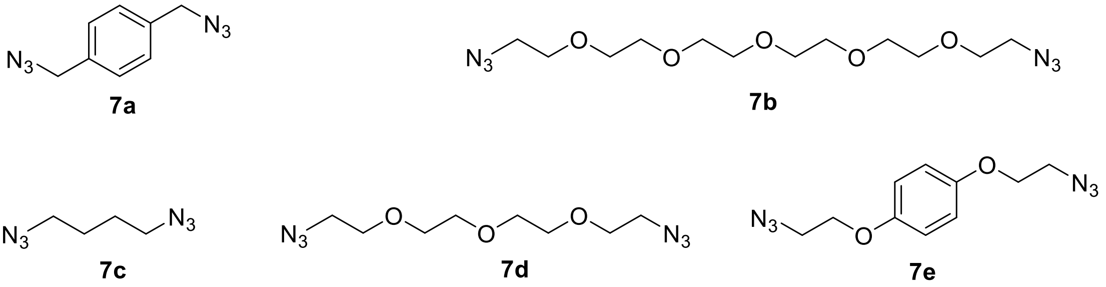

Supplementary Figure 12. The monomers of *di*-azides for MCP.

2.5 Structure analysis of polymers

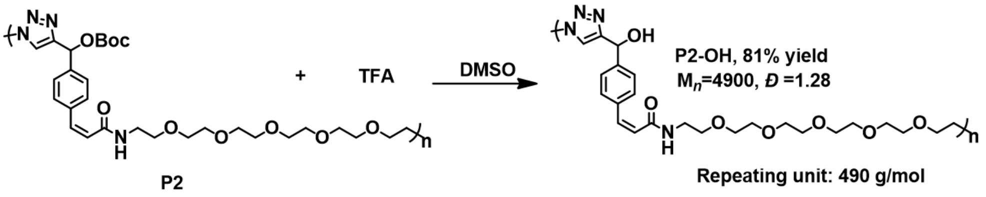

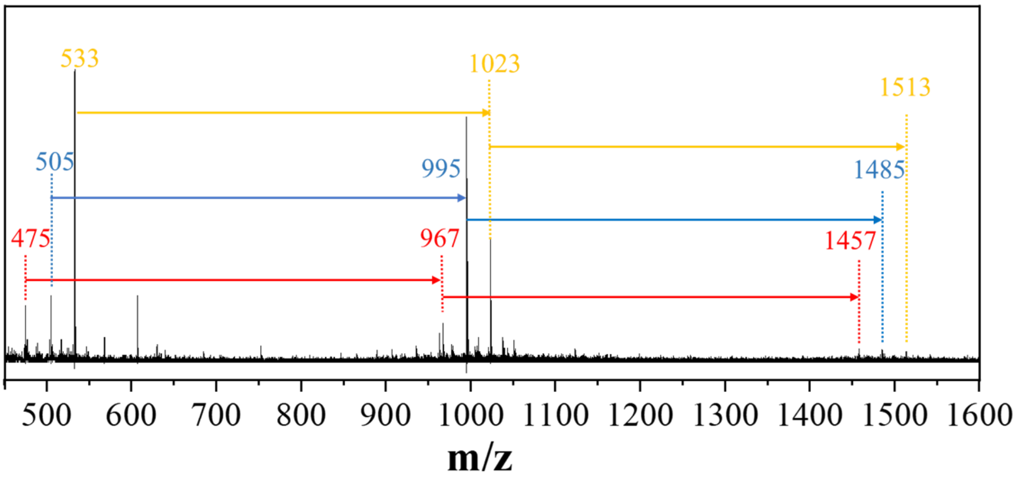

Measured (g/mol)	Exact Mass (expected, g/mol)	End-group
475	472	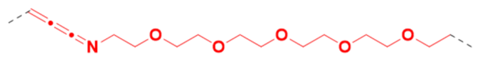
505	502	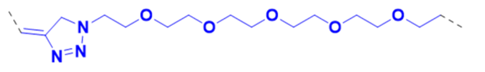
533	535	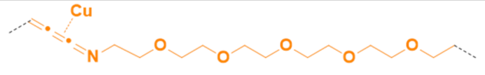
967	962	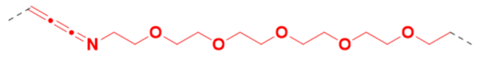
995	995	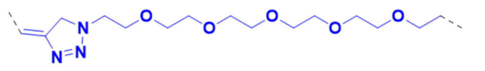
1024	1025	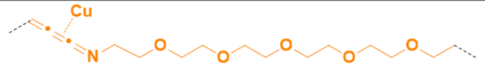
1457	1452	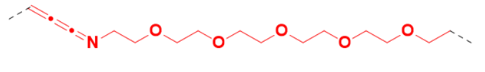
1485	1482	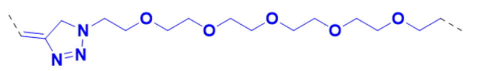
1513	1515	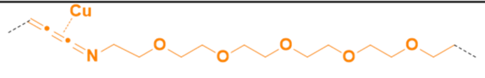

Supplementary Figure 13. MALDI-TOF spectrum and analysis of **P2-OH**.

3 Supplementary Notes

3.1 Characterization data of products

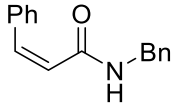

(*Z*)-*N*-Benzyl-3-phenylacrylamide (3a)

36 mg, 75% yield, yellow solid, mp = 58-60°C. ^{1}H NMR (400 MHz, $CDCl_3$) δ 7.42 – 7.37 (m, 2H), 7.29 – 7.24 (m, 6H), 7.14 (dd, *J* = 7.4, 1.9 Hz, 2H), 6.78 (d, *J* = 12.5 Hz, 1H), 6.02 (d, *J* = 12.5 Hz, 1H), 5.79 (s, 1H), 4.41 (d, *J* = 5.8 Hz, 2H).

Compound **3a** is known compound, and the proton spectrum is fully consistent with literature reported.[4,5]

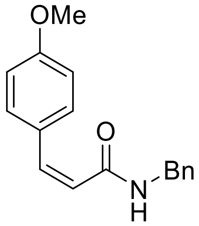

(*Z*)-*N*-Benzyl-3-(4-methoxyphenyl)acrylamide (3b)

37 mg, 69% yield, yellow oil. ^{1}H NMR (400 MHz, $CDCl_3$) δ 7.44 – 7.38 (m, 2H), 7.30 – 7.23 (m, 3H), 7.22 – 7.18 (m, 2H), 6.79 – 6.76 (m, 2H), 6.69 (d, *J* = 12.5 Hz, 1H), 5.89 (s, 1H), 5.88 (d, *J* = 12.5 Hz, 1H), 4.44 (d, *J* = 5.9 Hz, 2H), 3.79 (s, 3H).

Compound **3b** is known compound, and the proton spectrum is fully consistent with literature reported.[6]

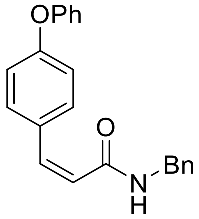

(*Z*)-*N*-Benzyl-3-(4-phenoxyphenyl)acrylamide (3c)

39 mg, 60% yield, white solid, mp = 93-95°C. ^{1}H NMR (400 MHz, $CDCl_3$) δ 7.46 (d, *J* = 8.3 Hz, 2H), 7.38 (t, *J* = 7.7 Hz, 3H), 7.31 (dd, *J* = 13.1, 5.9 Hz, 2H), 7.24 (d, *J* = 7.5 Hz, 2H), 7.17 (t, *J* = 7.0 Hz, 1H), 7.03 (d, *J* = 8.3 Hz, 2H), 6.90 (d, *J* = 8.2 Hz, 2H), 6.74 (d, *J* = 12.5 Hz, 1H), 5.97 (d, *J* = 12.5 Hz, 1H), 5.88 (s, 1H), 4.48 (d, *J* = 5.8 Hz, 2H). ^{13}C NMR (100 MHz, $CDCl_3$) δ 166.9, 157.9, 156.5, 137.7, 136.3, 130.9, 129.9, 129.6, 128.7, 128.1, 127.6, 123.8, 123.3, 119.4, 118.2, 43.7. HRMS (ESI-TOF) m/z calcd for $C_{22}H_{19}NO_2$ $(M+H)^+$ 330.1489, found 330.1498.

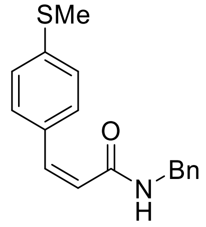

(*Z*)-*N*-Benzyl-3-(4-(methylthio)phenyl)acrylamide (3d)

40 mg, 71% yield, white solid, mp = 54-56°C. ^{1}H NMR (400 MHz, $CDCl_3$) δ 7.36 (d, *J* = 8.0 Hz, 2H), 7.29 (d, *J* = 7.1 Hz, 3H), 7.19 (d, *J* = 7.4 Hz, 2H), 7.10 (d, *J* = 8.2 Hz, 2H), 6.69 (d, *J* = 12.5 Hz, 1H), 5.95 (d, *J* = 12.5 Hz, 1H), 5.88 (s, 1H), 4.43 (d, *J* = 5.8 Hz, 2H), 2.46 (s, 3H). ^{13}C NMR (100 MHz, $CDCl_3$) δ 166.9, 139.6, 137.7, 136.3, 131.4, 129.6, 128.7, 128.1, 127.6, 125.8, 123.8, 43.6, 15.4. HRMS (ESI-TOF) m/z calcd for $C_{17}H_{17}NOS$ $(M+H)^+$ 284.1104, found 284.1109.

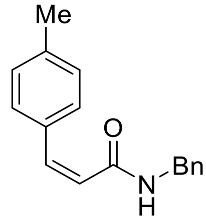

(*Z*)-*N*-Benzyl-3-(*p*-tolyl)acrylamide (3e)

38 mg, 76% yield, white solid. mp = 66-68°C. ^{1}H NMR (400 MHz, $CDCl_3$) δ 7.32 – 7.23 (m, 5H), 7.16 – 7.13 (m, 2H), 7.06 (d, *J* = 8.0 Hz, 2H), 6.72 (d, *J* = 12.5 Hz, 1H), 5.94 (d, *J* = 12.5 Hz, 1H), 5.91 (s, 1H), 4.39 (d, *J* = 5.8 Hz, 2H), 2.31 (s, 3H). ^{13}C NMR (100 MHz, $CDCl_3$) δ 167.1, 138.6, 137.8, 136.7, 132.1, 129.1, 129.0, 128.6, 128.0, 127.5, 123.9, 43.6, 21.3. HRMS (ESI-TOF) m/z calcd for $C_{17}H_{17}NO$ $(M+H)^+$ 252.1383, found 252.1390.

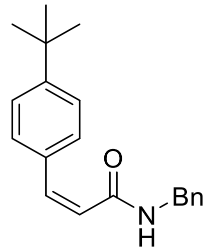

(*Z*)-*N*-Benzyl-3-(4-(*tert*-butyl)phenyl)acrylamide (3f)

35 mg, 60% yield, yellow oil. ^{1}H NMR (400 MHz, $CDCl_3$) δ 7.34 (d, *J* = 8.2 Hz, 3H), 7.31 – 7.26 (m, 4H), 7.18 – 7.14 (m, 2H), 6.76 (d, *J* = 12.5 Hz, 1H), 5.97 (d, *J* = 12.5 Hz, 1H), 5.77 (s, 1H), 4.43 (d, *J* = 5.7 Hz, 2H), 1.30 (s, 9H). ^{13}C NMR (100 MHz, $CDCl_3$) δ 167.0, 151.9, 137.6, 136.4, 131.9, 128.8, 128.7, 128.1, 127.5, 125.4, 124.0, 43.7, 34.7, 31.2. HRMS (ESI-TOF) m/z calcd for $C_{20}H_{23}NO$ $(M+H)^+$ 294.1852, found 294.1858.

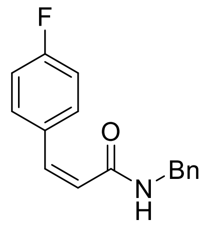

(*Z*)-*N*-Benzyl-3-(4-fluorophenyl)acrylamide (3g)
33 mg, 65% yield, yellow oil. ^{1}H NMR (400 MHz, $CDCl_3$) δ 7.42 – 7.32 (m, 2H), 7.24 – 7.16 (m, 3H), 7.13 – 7.10 (m, 2H), 6.86 (t, *J* = 8.7 Hz, 2H), 6.62 (d, *J* = 12.5 Hz, 1H), 5.89 (d, *J* = 12.5 Hz, 1H), 5.79 (s, 1H), 4.35 (d, *J* = 5.8 Hz, 2H). ^{13}C NMR (100 MHz, $CDCl_3$) δ 166.6, 164.00, 161.5, 137.6, 135.9, 131.2 (d, *J* = 8.2 Hz), 128.7, 128.0, 127.6, 124.1, 115.3 (d, *J* = 21.6 Hz), 43.6. ^{19}F NMR (565 MHz, $CDCl_3$) δ 112.2. HRMS (ESI-TOF) m/z calcd for $C_{16}H_{14}FNO$ $(M+H)^+$ 256.1132, found 256.1138.

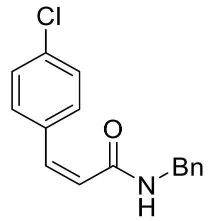

(*Z*)-*N*-Benzyl-3-(4-chlorophenyl)acrylamide (3h)
33 mg, 60% yield, yellow oil. ^{1}H NMR (400 MHz, $CDCl_3$) δ 7.37 (d, *J* = 8.3 Hz, 2H), 7.30 (q, *J* = 5.9 Hz, 3H), 7.22 (d, *J* = 8.3 Hz, 2H), 7.18 (d, *J* = 7.4 Hz, 2H), 6.69 (d, *J* = 12.5 Hz, 1H), 6.01 (d, *J* = 12.5 Hz, 1H), 5.85 (s, 1H), 4.42 (d, *J* = 5.8 Hz, 2H).^{13}C NMR (100 MHz, $CDCl_3$) δ 166.5, 137.5, 135.7, 134.5, 133.3, 130.5, 128.7, 128.6, 128.1, 127.7, 124.9, 43.6. HRMS (ESI-TOF) m/z calcd for $C_{16}H_{14}{}^{35}ClNO$ $(M+H)^+$ 272.0837, found 272.0841, $C_{16}H_{14}{}^{37}ClNO$ $(M+H)^+$ 274.0807, found 274.0814.

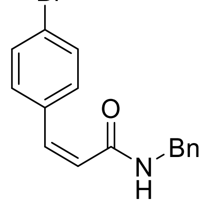

(*Z*)-*N*-Benzyl-3-(4-bromophenyl)acrylamide (3i)
42 mg, 67% yield, yellow oil. ^{1}H NMR (400 MHz, $CDCl_3$) δ 7.40 (d, *J* = 8.3 Hz, 2H), 7.36 – 7.23 (m, 5H), 7.20 (d, *J* = 7.4 Hz, 2H), 6.72 (d, *J* = 12.5 Hz, 1H), 6.03 (d, *J* = 12.5 Hz, 1H), 5.88 (s, 1H), 4.45 (d, *J* = 5.8 Hz, 2H). ^{13}C NMR (100 MHz, $CDCl_3$) δ 166.5, 137.5, 135.7, 133.8, 131.5, 130.7, 128.7, 128.1, 127.7, 125.0, 122.8, 43.6. HRMS (ESI-TOF) m/z calcd for $C_{16}H_{14}{}^{79}BrNO(M+H)^+$ 316.0332, found 316.0339, $C_{16}H_{14}{}^{81}BrNO(M+H)^+$ 318.0311, found 316.0320.

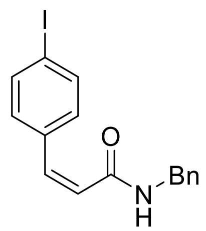

(*Z*)-*N*-Benzyl-3-(4-iodophenyl)acrylamide (3j)
44 mg, 60% yield, yellow solid, mp = 84-86°C. ^{1}H NMR (400 MHz, $CDCl_3$) δ 7.61 (d, *J* = 8.3 Hz, 2H), 7.39 – 7.31 (m, 3H), 7.19 (dd, *J* = 8.3, 5.0 Hz, 4H), 6.70 (d, *J* = 12.5 Hz, 1H), 6.05 (d, *J* = 12.5 Hz, 1H), 5.84 (s, 1H), 4.45 (d, *J* = 5.8 Hz, 2H). ^{13}C NMR (100 MHz, $CDCl_3$) δ 166.4, 137.5, 137.5, 135.8, 134.4, 130.7, 128.8, 128.1, 127.7, 125.2, 94.6, 43.6. HRMS (ESI-TOF) m/z calcd for $C_{16}H_{14}INO$ $(M+H)^+$ 364.0193, found 364.0200.

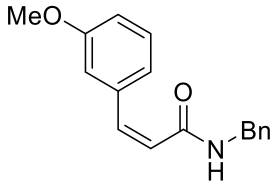

(*Z*)-*N*-Benzyl-3-(3-methoxyphenyl)acrylamide (3k)
35 mg, 65% yield, yellow oil. ^{1}H NMR (400 MHz, $CDCl_3$) δ 7.32 – 7.25 (m, 4H), 7.18 – 7.13 (m, 2H), 7.03 – 6.97 (m, 2H), 6.86 (dd, *J* = 8.3, 2.3 Hz, 1H), 6.79 (d, *J* = 12.5 Hz, 1H), 6.05 (d, *J* = 12.5 Hz, 1H), 5.84 (s, 1H), 4.43 (d, *J* = 5.8 Hz, 2H), 3.77 (s, 3H). ^{13}C NMR (100MHz, $CDCl_3$) δ 166.9, 159.6, 137.6, 136.3, 136.3, 129.5, 128.6, 127.9, 127.5, 125.1, 121.3, 114.6, 113.9, 55.2, 43.6. HRMS (ESI-TOF) m/z calcd for $C_{17}H_{17}NO_2$ $(M+H)^+$ 268.1332, found 268.1339.

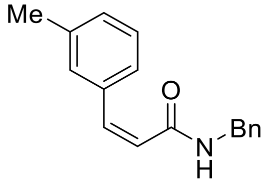

(*Z*)-*N*-Benzyl-3-(*m*-tolyl)acrylamide (3l)
34 mg, 68% yield, yellow oil. ^{1}H NMR (400 MHz, $CDCl_3$) δ 7.30 – 7.23 (m, 3H), 7.22 – 7.16 (m, 3H), 7.15 – 7.11 (m, 2H), 7.09 (d, *J* = 7.0 Hz, 1H), 6.76 (t, *J* = 9.7 Hz, 1H), 6.01 (d, *J* = 12.5 Hz, 1H), 5.77 (s, 1H), 4.41 (d, *J* = 5.8 Hz, 2H), 2.28 (s, 3H). ^{13}C NMR (100 MHz, $CDCl_3$) δ 167.0, 138.2, 137.7, 136.5, 135.0, 129.5, 129.4, 129.1, 128.6, 128.4, 127.9, 127.5, 125.9, 124.8, 43.6, 21.3. HRMS (ESI-TOF) m/z calcd for $C_{17}H_{17}NO$ $(M+H)^+$ 252.1383, found 252.1388.

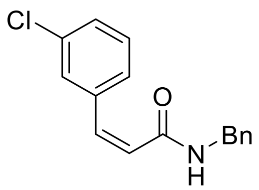

(*Z*)-*N*-Benzyl-3-(3-chlorophenyl)acrylamide (3m)
33 mg, 60% yield, yellow oil. ^{1}H NMR (400 MHz, $CDCl_3$) δ 7.38 (d, *J* = 8.5 Hz, 2H), 7.33 – 7.27 (m, 5H), 7.20 – 7.15 (m, 2H), 6.68 (d, *J* = 12.5 Hz, 1H), 6.02 (d, *J* = 12.5 Hz, 1H), 5.82 (s, 1H), 4.42 (d, *J* = 5.9 Hz, 2H). ^{13}C NMR (100 MHz, $CDCl_3$) δ 166.5, 137.5, 135.7, 133.8, 131.5, 130.7, 128.7, 128.0, 127.7, 125.0, 122.8, 43.6. HRMS (ESI-TOF) m/z calcd for $C_{16}H_{14}{}^{35}ClNO$ $(M+H)^+$ 272.0837, found 272.0844, $C_{16}H_{14}{}^{37}ClNO$ $(M+H)^+$ 274.0807, found 274.0814.

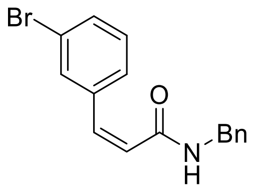

(*Z*)-*N*-Benzyl-3-(3-bromophenyl)acrylamide (3n)
38 mg, 61% yield, yellow soild, mp = 55-57°C. ^{1}H NMR (400 MHz, $CDCl_3$) δ 7.60 (s, 1H), 7.39 (dd, *J* = 16.8, 7.9 Hz, 2H), 7.33 – 7.27 (m, 3H), 7.18 (d, *J* = 6.6 Hz, 2H), 7.13 (t, *J* = 7.9 Hz, 1H), 6.68 (t, *J* = 11.4 Hz, 1H), 6.05 (d, *J* = 12.5 Hz, 1H), 5.77 (s, 1H), 4.44 (d, *J* = 5.8 Hz, 2H). ^{13}C NMR (100 MHz, $CDCl_3$) δ 166.3, 137.4, 137.0, 135.1, 131.8, 131.6, 130.0, 128.8, 128.0, 127.7, 127.6, 125.8, 122.5, 43.7. HRMS (ESI-TOF) m/z calcd for $C_{16}H_{14}{}^{79}BrNO$ $(M+H)^+$ 316.0332, found 316.0341, $C_{16}H_{14}{}^{81}BrNO$ $(M+H)^+$ 318.0311, found 318.0321.

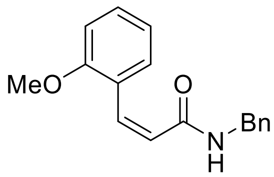

(*Z*)-*N*-Benzyl-3-(2-methoxyphenyl)acrylamide (3o)
30 mg, 56% yield, yellow oil. ^{1}H NMR (400 MHz, $CDCl_3$) δ 7.37 (d, *J* = 7.5 Hz, 1H), 7.31 – 7.26 (m, 4H), 7.08 (d, *J* = 6.8 Hz, 2H), 6.96 (d, *J* = 12.4 Hz, 1H), 6.89 – 6.77 (m, 2H), 6.05 (d, *J* = 12.5 Hz, 1H), 5.87 (s, 1H), 4.37 (d, *J* = 5.7 Hz, 2H), 3.78 (s, 3H). ^{13}C NMR (100 MHz, $CDCl_3$) δ 167.1, 156.8, 137.8, 132.4, 130.2, 130.1, 128.6, 127.9, 127.4, 125.1, 123.9, 120.5, 110.5, 55.4, 43.5. HRMS (ESI-TOF) m/z calcd for $C_{17}H_{17}NO_2$ $(M+H)^+$ 268.1332, found 268.1340.

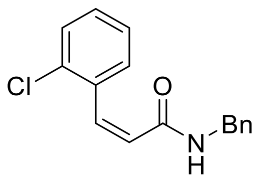

(*Z*)-*N*-Benzyl-3-(2-chlorophenyl)acrylamide (3p)
29 mg, 54% yield, yellow oil. ^{1}H NMR (600 MHz, $CDCl_3$) δ 7.45 (dd, *J* = 7.7, 1.3 Hz, 1H), 7.37 – 7.34 (m, 1H), 7.25 – 7.20 (m, 4H), 7.14 – 7.10 (m, 1H), 7.10 – 7.06 (m, 2H), 6.96 (d, *J* = 12.5 Hz, 1H), 6.21 (d, *J* = 12.5 Hz, 1H), 5.76 (s, 1H), 4.50 (d, *J* = 3.9 Hz, 2H). ^{13}C NMR (100 MHz, $CDCl_3$) δ 166.2, 137.5, 133.8, 133.6, 133.3, 130.6, 129.7, 129.4, 128.6, 127.9, 127.5, 126.7, 126.4, 43.5. HRMS (ESI-TOF) m/z calcd for $C_{16}H_{14}{}^{35}ClNO$ $(M+H)^+$ 272.0837, found 272.0845, $C_{16}H_{143}{}^{37}ClNO$ $(M+H)^+$ 274.0807, found 272.0815.

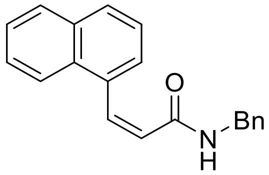

(*Z*)-*N*-Benzyl-3-(naphthalen-1-yl)acrylamide (3q)
32 mg, 55% yield, yellow oil. ^{1}H NMR (400 MHz, $CDCl_3$) δ 7.98 – 7.92 (m, 1H), 7.89 – 7.85 (m, 1H), 7.80 (d, *J* = 8.2 Hz, 1H), 7.53 (dd, *J* = 6.1, 3.3 Hz, 2H), 7.46 (d, *J* = 7.0 Hz, 1H), 7.41 – 7.31 (m, 2H), 7.19 – 7.09 (m, 3H), 6.77 (d, *J* = 7.0 Hz, 2H), 6.34 (d, *J* = 12.3 Hz, 1H), 5.57 (s, 1H), 4.22 (d, *J* = 5.7 Hz, 2H). ^{13}C NMR (100 MHz, $CDCl_3$) δ 166.5, 137.4, 135.0, 133.5, 132.5, 131.0, 128.9, 128.7, 128.5, 127.9, 127.5, 127.2, 126.8, 126.6, 126.4, 125.5, 124.3, 43.4. HRMS (ESI-TOF) m/z calcd for $C_{20}H_{17}NO$ $(M+H)^+$ 288.1383, found 288.1389.

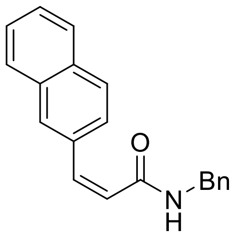

(*Z*)-*N*-Benzyl-3-(naphthalen-2-yl)acrylamide (3r)
36 mg, 62% yield, yellow solid, mp = 78-80℃. ^{1}H NMR (400 MHz, $CDCl_3$) δ 7.87 (s, 1H), 7.84 – 7.70 (m, 3H), 7.56 – 7.46 (m, 3H), 7.21 – 7.13 (m, 3H), 7.08 (d, *J* = 6.6 Hz, 2H), 6.94 (d, *J* = 12.5 Hz, 1H), 6.11 (d, *J* = 12.5 Hz, 1H), 5.82 (s, 1H), 4.42 (d, *J* = 5.8 Hz, 2H). ^{13}C NMR (100 MHz, $CDCl_3$) δ 167.0, 137.6, 136.5, 133.2, 133.1, 132.5, 128.7, 128.6, 128.3, 128.1, 127.9, 127.7, 127.5, 126.7, 126.4, 126.3, 125.0, 43.6. HRMS (ESI-TOF) m/z calcd for $C_{20}H_{17}NO$ $(M+H)^+$ 288.1383, found 288.1387.

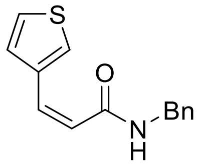

(*Z*)-*N*-Benzyl-3-(thiophen-3-yl)acrylamide (3s)
30 mg, 61% yield, yellow solid, mp = 58-60°C. ^{1}H NMR (400 MHz, $CDCl_3$) δ 7.74 (s, 1H), 7.41 – 7.27 (m, 5H), 7.25 – 7.18 (m, 2H), 6.68 (d, *J* = 12.5 Hz, 1H), 6.02 (s, 1H), 5.82 (d, *J* = 12.5 Hz, 1H), 4.47 (d, *J* = 5.5 Hz, 2H). ^{13}C NMR (100 MHz, $CDCl_3$) δ 166.6, 137.9, 136.3, 131.5, 129.1, 128.7, 128.0, 128.0, 127.6, 125.3, 121.6, 43.7. HRMS (ESI-TOF) m/z calcd for $C_{14}H_{13}NOS$ $(M+H)^+$ 244.0791, found 244.0796.

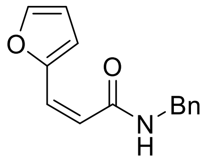

(*Z*)-*N*-Benzyl-3-(furan-2-yl)acrylamide (3t)
30 mg, 65% yield, yellow solid, mp = 88-90°C. ^{1}H NMR (400 MHz, $CDCl_3$) δ 7.38 – 7.32 (m, 5H), 7.29 (dd, *J* = 8.4, 3.9 Hz, 1H), 7.21 (d, *J* = 3.3 Hz, 1H), 6.56 (d, *J* = 13.0 Hz, 1H), 6.44 (s, 1H), 6.22 (s, 1H), 5.73 (d, *J* = 13.0 Hz, 1H), 4.55 (d, *J* = 5.7 Hz, 2H). ^{13}C NMR (100 MHz, $CDCl_3$) δ 166.0, 150.7, 143.4, 138.1, 128.7, 128.0, 127.6, 125.0, 118.9, 115.4, 112.2, 43.7. HRMS (ESI-TOF) m/z calcd for $C_{14}H_{13}NO_2$ $(M+H)^+$ 228.1019, found 228.1025.

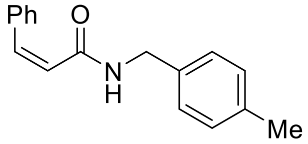

(*Z*)-*N*-(4-Methylbenzyl)-3-phenylacrylamide (3u)
37 mg, 74% yield, yellow oil. ^{1}H NMR (400 MHz, $CDCl_3$) δ 7.43 – 7.37 (m, 2H), 7.32 – 7.26 (m, 3H), 7.08 (d, *J* = 8.0 Hz, 2H), 7.03 (d, *J* = 8.0 Hz, 2H), 6.77 (d, *J* = 12.5 Hz, 1H), 6.01 (d, *J* = 12.5 Hz, 1H), 5.75 (s, 1H), 4.37 (d, *J* = 5.7 Hz, 2H), 2.32 (s, 3H). ^{13}C NMR (100 MHz, $CDCl_3$) δ 166.9, 137.2, 136.4, 135.0, 134.6, 129.3, 128.9, 128.6, 128.5, 128.0, 124.8, 43.4, 21.1. HRMS (ESI-TOF) m/z calcd for $C_{17}H_{17}NO$ $(M+H)^+$ 252.1383, found 252.1388.

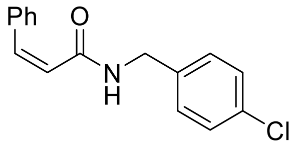

(*Z*)-*N*-(4-Chlorobenzyl)-3-phenylacrylamide (3v)
38 mg, 70% yield, yellow oil. ^{1}H NMR (400 MHz, $CDCl_3$) δ 7.40 – 7.34 (m, 2H), 7.31 – 7.26 (m, 3H), 7.23 (d, *J* = 8.4 Hz, 2H), 7.07 (d, *J* = 8.4 Hz, 2H), 6.80 (d, *J* = 12.5 Hz, 1H), 6.02 (d, *J* = 12.5 Hz, 1H), 5.80 (s, 1H), 4.36 (d, *J* = 5.9 Hz, 2H). ^{13}C

NMR (100 MHz, $CDCl_3$) δ 167.0, 136.7, 136.2, 134.9, 133.3, 129.3, 128.9, 128.7, 128.6, 128.5, 124.7, 42.8. HRMS (ESI-TOF) m/z calcd for $C_{16}H_{14}{}^{35}ClNO$ $(M+H)^+$ 272.0837, found 272.0845, $C_{16}H_{14}{}^{37}ClNO$ $(M+H)^+$ 274.0807, found 274.0814.

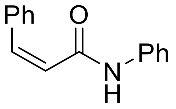

(*Z*)-*N*,3-Diphenylacrylamide (3w)
22 mg, 50% yield, white solid. m.p = 93 – 94 ℃. ^{1}H NMR (600 MHz, $CDCl_3$) δ 7.50 (d, *J* = 7.1 Hz, 2H), 7.39 – 7.32 (m, 5H), 7.30 – 7.26 (m, 2H), 7.22 (s, 1H), 7.09 (t, *J* = 7.3 Hz, 1H), 6.92 (d, *J* = 12.5 Hz, 1H), 6.11 (d, *J* = 12.5 Hz, 1H).
Compound **3w** is known compound, and the proton spectrum is fully consistent with literature reported.[4,5]

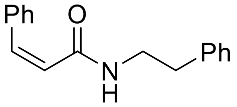

(*Z*)-*N*-Phenethyl-3-phenylacrylamide (3x)
40 mg, 74% yield, yellow oil. ^{1}H NMR (400 MHz, $CDCl_3$) δ 7.44 – 7.39 (m, 2H), 7.36 – 7.29 (m, 3H), 7.25 – 7.16 (m, 3H), 7.03 – 6.99 (m, 2H), 6.74 (d, *J* = 12.5 Hz, 1H), 5.96 (d, *J* = 12.5 Hz, 1H), 5.52 (s, 1H), 3.50 (dd, *J* = 13.0, 7.0 Hz, 2H), 2.71 (t, *J* = 7.0 Hz, 2H). ^{13}C NMR (100 MHz, $CDCl_3$) δ 167.1, 138.6, 136.2, 135.1, 128.9, 128.6, 128.6, 128.5, 126.5, 125.0, 40.4, 35.2. HRMS (ESI-TOF) m/z calcd for $C_{17}H_{17}NO$ $(M+Na)^+$ 274.1202, found 274.1210.

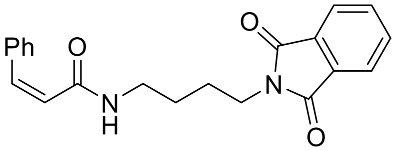

(*Z*)-*N*-(4-(1,3-Dioxoisoindolin-2-yl)butyl)-3-phenylacrylamide (3y)
51 mg, 73% yield, yellow oil. ^{1}H NMR (600 MHz, $CDCl_3$) δ 7.86 – 7.81 (m, 2H), 7.74 – 7.69 (m, 2H), 7.41 (d, *J* = 7.5 Hz, 2H), 7.31 (t, *J* = 7.6 Hz, 2H), 7.23 (t, *J* = 7.4 Hz, 1H), 6.76 (d, *J* = 12.5 Hz, 1H), 5.98 (d, *J* = 12.5 Hz, 1H), 5.67 (s, 1H), 3.62 (t, *J* = 7.1 Hz, 2H), 3.26 (dd, *J* = 13.2, 6.9 Hz, 2H), 1.63 – 1.54 (m, 2H), 1.49 – 1.40 (m, 2H). ^{13}C NMR (100 MHz, $CDCl_3$) δ 168.4, 167.1, 136.1, 135.1, 134.0, 132.1, 128.9, 128.5, 128.4, 125.1, 123.2, 38.9, 37.4, 26.4, 26.1. HRMS (ESI-TOF) m/z calcd for $C_{21}H_{20}N_2O_3$ $(M+H)^+$ 349.1547, found 349.1556.

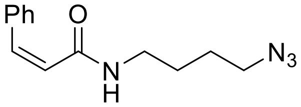

(*Z*)-*N*-(4-Azidobutyl)-3-phenylacrylamide (3z)
29 mg, 60% yield, yellow oil. ^{1}H NMR (400 MHz, $CDCl_3$) δ 7.42 (d, *J* = 6.6 Hz, 2H), 7.34 (dd, *J* = 15.0, 7.3 Hz, 3H), 6.79 (d, *J* = 12.5 Hz, 1H), 6.00 (d, *J* = 12.5 Hz, 1H),

5.50 (s, 1H), 3.28 – 3.19 (m, 4H), 1.50 – 1.44 (m, 4H). ^{13}C NMR (100 MHz, $CDCl_3$) δ 167.2, 136.2, 135.1, 128.8, 128.7, 128.5, 125.2, 51.0, 38.8, 26.5, 26.3. HRMS (ESI-TOF) m/z calcd for $C_{13}H_{16}N_4O$ $(M+H)^+$ 245.1397, found 245.1404.

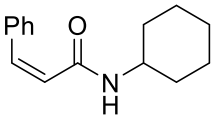

(*Z*)-*N*-Cyclohexyl-3-phenylacrylamide (3aa)

34 mg, 74% yield, white solid, mp = 65-67°C. ^{1}H NMR (600 MHz, $CDCl_3$) δ 7.38 – 7.33 (m, 2H), 7.29 – 7.21 (m, 3H), 6.68 (d, *J* = 12.5 Hz, 1H), 5.92 (d, *J* = 12.5 Hz, 1H), 5.29 (s, 1H), 3.79 – 3.67 (m, 1H), 1.78 – 1.71 (m, 2H), 1.57 – 1.46 (m, 3H), 1.28 – 1.16 (m, 2H), 1.05 – 0.96 (m, 1H), 0.93 – 0.80 (m, 2H). ^{13}C NMR (100 MHz, $CDCl_3$) δ 166.1, 135.6, 135.1, 128.9, 128.5, 128.4, 125.6, 48.0, 32.6, 25.4, 24.7. HRMS (ESI-TOF) m/z calcd for $C_{15}H_{19}NO$ $(M+H)^+$ 230.1539, found 230.1545.

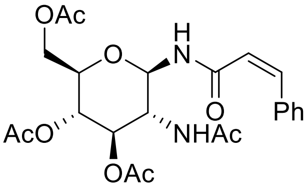

(*2R,3S,4R,5R,6R*)-5-Acetamido-2-(acetoxymethyl)-6-((*Z*)-3-phenylacrylamido)tetrahydro-2*H*-pyran-3,4-diyl diacetate (3ab)

68 mg, 72% yield, yellow oil. ^{1}H NMR (400 MHz, $CDCl_3$) δ 7.57 (d, *J* = 7.7 Hz, 2H), 7.37 – 7.31 (m, 3H), 7.12 (d, *J* = 8.3 Hz, 1H), 6.80 (d, *J* = 12.6 Hz, 1H), 6.09 (d, *J* = 8.3 Hz, 1H), 5.90 (d, *J* = 12.6 Hz, 1H), 5.18 – 5.03 (m, 3H), 4.32 (dd, *J* = 12.4, 4.2 Hz, 1H), 4.12 (dd, *J* = 16.4, 8.0 Hz, 2H), 3.83 – 3.69 (m, 1H), 2.11 (s, 3H), 2.07 (d, *J* = 1.4 Hz, 3H), 2.07 (d, *J* = 1.4 Hz, 3H), 1.83 (s, 3H). ^{13}C NMR (100 MHz, $CDCl_3$) δ 171.9, 171.9, 170.7, 169.3, 167.1, 139.8, 134.6, 129.7, 128.9, 128.2, 122.3, 80.2, 73.5, 73.1, 67.8, 61.8, 53.3, 23.0, 20.7, 20.7, 20.6. HRMS (ESI-TOF) m/z calcd for $C_{23}H_{28}N_2O_9$ $(M+H)^+$ 477.1868, found 477.1875.

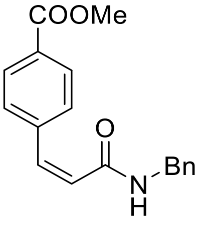

Methyl (*Z*)-4-(3-(benzylamino)-3-oxoprop-1-en-1-yl)benzoate (3ac)

12 mg, 20% yield, white solid, mp = 58-60°C. ^{1}H NMR (400 MHz, $CDCl_3$) δ 7.93 (d, *J* = 8.4 Hz, 2H), 7.47 (d, *J* = 8.3 Hz, 2H), 7.31 – 7.26 (m, 3H), 7.20 – 7.13 (m, 2H), 6.79 (d, *J* = 12.5 Hz, 1H), 6.12 (d, *J* = 12.5 Hz, 1H), 5.74 (s, 1H), 4.42 (d, *J* = 5.8 Hz, 2H), 3.92 (s, 3H). ^{13}C NMR (100 MHz, $CDCl_3$) δ 166.6, 166.3, 139.4, 137.4, 135.6, 129.9, 129.6, 128.9, 128.7, 128.0, 127.7, 126.3, 52.2, 43.7. HRMS (ESI-TOF) m/z calcd for $C_{18}H_{17}NO_3$ $(M+H)^+$ 296.1281, found 296.1286.

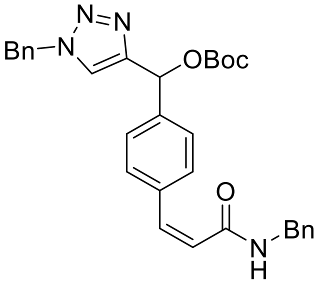

(*Z*)-(1-Benzyl-1*H*-1,2,3-triazol-4-yl)(4-(3-(benzylamino)-3-oxoprop-1-en-1-yl)phenyl)methyl *tert*-butyl carbonate (3ad)

79 mg, 75% yield, yellow oil. ^{1}H NMR (400 MHz, $CDCl_3$) δ 7.44 (d, *J* = 8.1 Hz, 2H), 7.37 (t, *J* = 6.1 Hz, 7H), 7.27 (s, 4H), 7.18 (d, *J* = 6.9 Hz, 2H), 6.80 (s, 1H), 6.74 (d, *J* = 12.6 Hz, 1H), 6.03 (d, *J* = 12.5 Hz, 1H), 5.85 (s, 1H), 5.56 – 5.41 (m, 2H), 4.43 (d, *J* = 5.8 Hz, 2H), 1.47 (s, 9H). ^{13}C NMR (100 MHz, $CDCl_3$) δ 166.7, 152.4, 147.4, 138.8, 137.6, 136.1, 134.9, 134.3, 129.4, 129.2, 128.8, 128.7, 128.1, 128.0, 127.6, 126.9, 124.8, 122.2, 83.0, 72.8, 54.2, 43.6, 27.8. ^{1}H NMR (400 MHz, d_6-DMSO) δ 8.67 (s, 1H), 8.16 (s, 1H), 7.60 (d, *J* = 8.1 Hz, 2H), 7.40 – 7.24 (m, 12H), 6.68 (d, *J* = 14.4 Hz, 2H), 6.09 (d, *J* = 12.8 Hz, 1H), 5.58 (s, 2H), 4.32 (d, *J* = 5.9 Hz, 2H), 1.39 (s, 9H). ^{13}C NMR (100 MHz, d_6-DMSO) δ 166.2, 152.4, 139.6, 136.4, 135.9, 135.7, 130.2, 129.2, 128.7, 128.7, 128.5, 128.0, 127.3, 126.8, 125.3, 124.1, 82.8, 72.7, 53.3, 42.6, 27.8. HRMS (ESI-TOF) m/z calcd for $C_{31}H_{32}N_4O_4$ $(M+H)^+$525.2496, found 525.2501.

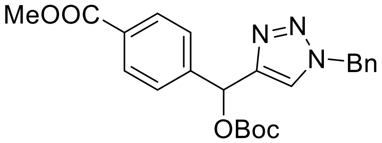

Methyl 4-((1-benzyl-1*H*-1,2,3-triazol-4-yl)((*tert*-butoxycarbonyl)oxy)methyl)benzoate (4a)

45 mg, 51 % yield, white solid, mp = 98-100°C. ^{1}H NMR (400 MHz, $CDCl_3$) δ 8.02 (d, *J* = 8.3 Hz, 2H), 7.51 (d, *J* = 8.3 Hz, 2H), 7.38 – 7.31 (m, 4H), 7.23 (dd, *J* = 6.6, 2.7 Hz, 2H), 6.83 (s, 1H), 5.47 (q, *J* = 14.8 Hz, 2H), 3.89 (s, 3H), 1.43 (s, 9H). ^{13}C NMR (100 MHz, $CDCl_3$) δ 166.7, 152.3, 147.0, 143.3, 134.2, 130.1, 130.0, 129.2, 128.9, 128.2, 126.7, 122.3, 83.2, 72.6, 54.3, 52.2, 27.7. HRMS (ESI-TOF) m/z calcd for $C_{23}H_{25}N_3O_5$ $(M+H)^+$ 446.1686, found 446.1687.

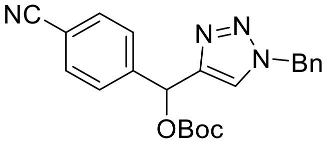

(1-Benzyl-1*H*-1,2,3-triazol-4-yl)(4-cyanophenyl)methyl *tert*-butyl carbonate (4b)

62 mg, 80% yield, yellow oil. ^{1}H NMR (400 MHz, $CDCl_3$) δ 7.64 (d, *J* = 8.2 Hz, 2H), 7.56 (d, *J* = 8.3 Hz, 2H), 7.40 (s, 1H), 7.38 – 7.33 (m, 3H), 7.28 – 7.20 (m, 2H), 6.79 (s, 1H), 5.48 (q, *J* = 14.8 Hz, 2H), 1.43 (s, 9H). ^{13}C NMR (100 MHz, $CDCl_3$) δ 152.2, 146.4, 143.5, 134.2, 132.5, 129.2, 128.9, 128.2, 127.5, 122.2, 118.5, 112.3, 83.5, 72.2,

54.3, 27.7. HRMS (ESI-TOF) m/z calcd for $C_{22}H_{22}N_4O_3$ $(M+H)^+$ 391.1765, found 391.1766.

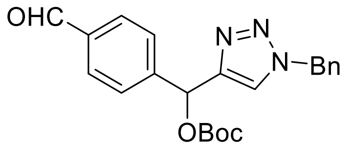

(1-Benzyl-1*H*-1,2,3-triazol-4-yl)(4-formylphenyl)methyl *tert*-butyl carbonate (4c)
70 mg, 84% yield, yellow oil. ^{1}H NMR (400 MHz, $CDCl_3$) δ 10.00 (s, 1H), 7.87 (d, *J* = 8.2 Hz, 2H), 7.62 (d, *J* = 8.2 Hz, 2H), 7.38 – 7.33 (m, 4H), 7.24 (dd, *J* = 6.6, 2.8 H z, 2H), 6.84 (s, 1H), 5.56 – 5.37 (m, 2H), 1.44 (s, 9H). ^{13}C NMR (100 MHz, $CDCl_3$) δ 191.8, 152.3, 146.8, 144.9, 136.2, 134.2, 130.1, 129.2, 128.9, 128.2, 127.3, 122.2, 83.4, 72.6, 54.3, 27.7. HRMS (ESI-TOF) m/z calcd for $C_{22}H_{23}N_3O_4$ $(M+Na)^+$ 416.1581, found 416.1586.

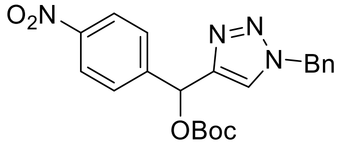

(1-Benzyl-1*H*-1,2,3-triazol-4-yl)(4-nitrophenyl)methyl *tert*-butyl carbonate (4d)
67 mg, 82% yield, yellow oil. ^{1}H NMR (400 MHz, $CDCl_3$) δ 8.20 (d, *J* = 8.7 Hz, 2H), 7.63 (d, *J* = 8.7 Hz, 2H), 7.41 (s, 1H), 7.39 – 7.33 (m, 3H), 7.24 (dd, *J* = 6.7, 2.9 Hz, 2H), 6.84 (s, 1H), 5.49 (q, *J* = 14.8 Hz, 2H), 1.44 (s, 9H). ^{13}C NMR (100 MHz, $CDCl_3$) δ 152.2, 147.8, 146.3, 145.4, 134.1, 129.2, 129.0, 128.2, 127.7, 123.9, 122.2, 83.6, 72.1, 54.4, 27.7. HRMS (ESI-TOF) m/z calcd for $C_{21}H_{22}N_4O_5$ $(M+H)^+$ 411.1663, found 411.1664.

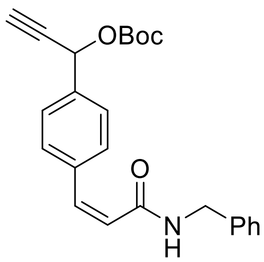

(*Z*)-1-(4-(3-(Benzylamino)-3-oxoprop-1-en-1-yl)phenyl)prop-2-yn-1-yl *tert*-butyl carbonate (6a)
43 mg, 55% yield, yellow oil. ^{1}H NMR (600 MHz, $CDCl_3$) δ 7.43 (q, *J* = 8.3 Hz, 4H), 7.30 (t, *J* = 7.2 Hz, 3H), 7.15 (d, *J* = 7.1 Hz, 2H), 6.77 (d, *J* = 12.5 Hz, 1H), 6.21 (d, *J* = 2.2 Hz, 1H), 6.05 (d, *J* = 12.5 Hz, 1H), 5.72 (s, 1H), 4.42 (d, *J* = 5.8 Hz, 2H), 2.70 (d, *J* = 2.2 Hz, 1H), 1.50 (s, 9H). ^{13}C NMR (100 MHz, $CDCl_3$) δ 166.7, 152.4, 137.5, 136.5, 135.7, 129.2, 128.7, 128.0, 127.8, 127.6, 125.4, 83.3, 79.7, 76.1, 67.7, 43.7, 29.7, 27.8. HRMS (ESI-TOF) m/z calcd for $C_{24}H_{25}NO_4$ $(M+H)^+$ 392.1856, found 392.1863.

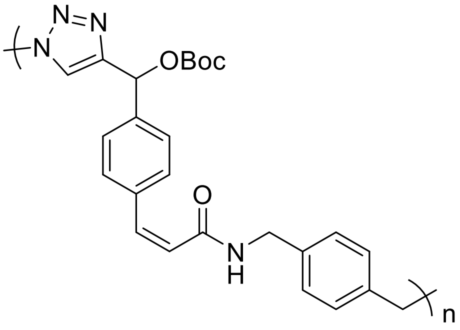

P1 (5a/7a/H_2O)

48 mg, 83% yield, brown soild. ^{1}H NMR (400 MHz, DMSO) δ 8.55 (1H), 8.07 (1H), 7.55 – 7.15 (8H), 6.62 (1H), 6.58 (1H), 5.98 (1H), 5.46 (2H), 4.27 (2H), 1.27 (9H).

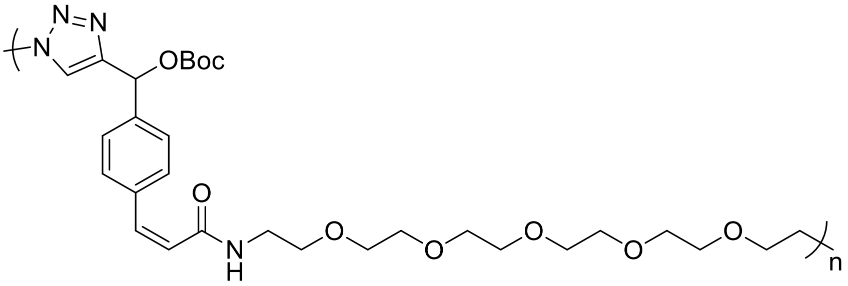

P2 (5a/7b/H_2O)

64 mg, 87% yield, brown soild. ^{1}H NMR (400 MHz, DMSO) δ 8.20 (1H), 8.05 (1H), 7.74 – 7.25 (4H), 6.73 (1H), 6.64 (1H), 6.02 (1H), 4.47 (4H), 3.78 (4H), 3.53 – 3.37 (16H), 1.38 (9H).

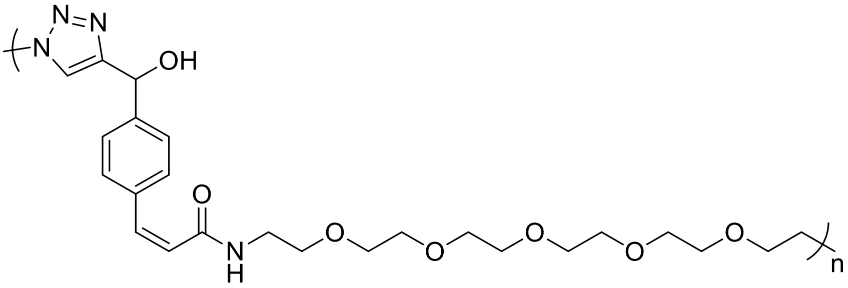

P2-OH

51 mg, 81% yield, brown soild.^{1}H NMR (400 MHz, DMSO) δ 8.17 (1H), 8.03 (1H), 7.70 – 7.20 (4H), 6.73 (1H), 6.62 (1H), 6.00 (1H), 4.48 (4H), 3.78 (4H), 3.59-3 – 3.39 (16H).

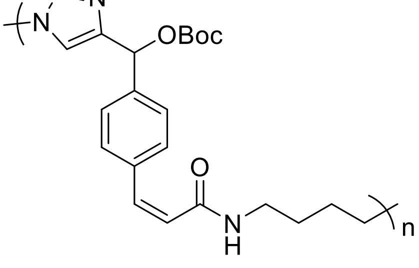

P3 (5a/7c/H_2O)

45 mg, 82% yield, brown soild. ^{1}H NMR (600 MHz, DMSO) δ 8.02 (2H), 7.61 – 7.11 (4H), 6.65 (1H), 6.55 (1H), 5.91 (1H), 4.26 (4H), 1.67 (4H), 1.29 (9H).

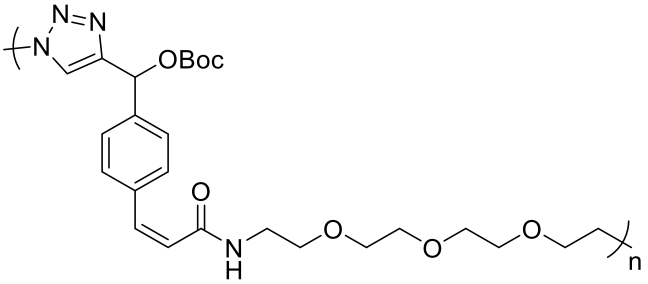

P4 (5a/7d/H_2O)

55 mg, 85% yield, brown soild. ^{1}H NMR (400 MHz, DMSO) δ 8.18 (1H), 8.04 (1H), 7.72 – 7.10 (4H), 6.73 (1H), 6.62 (1H), 6.01 (1H), 4.46 (4H), 3.75 (4H), 3.43 (8H), 1.36 (9H).

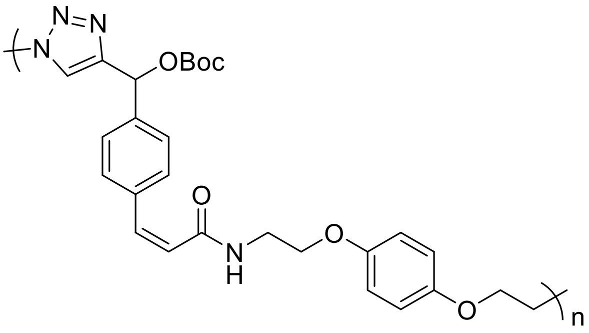

P5 (5a/7e/H_2O)

57 mg, 88% yield, brown soild. ^{1}H NMR (600 MHz, DMSO) δ 8.29 (1H), 8.07 (1H), 7.61 – 7.18 (4H), 6.82 – 6.50 (6H), 5.96 (1H), 4.60 (4H), 4.22 (4H), 1.28 (9H).

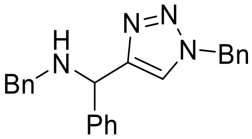

***N*-Benzyl-1-(1-benzyl-1*H*-1,2,3-triazol-4-yl)-1-phenylmethanamine (S1)**

52 mg, 74% yield, yellow solid, mp = 136 – 138 °C. ^{1}H NMR (400 MHz, $CDCl_3$) δ 7.45 (d, *J* = 7.6 Hz, 2H), 7.38 – 7.30 (m, 5H), 7.29 (d, *J* = 4.4 Hz, 4H), 7.26 – 7.18 (m, 4H), 7.14 (s, 1H), 5.49 – 5.37 (m, 2H), 5.07 (s, 1H), 3.78 – 3.66 (m, 2H), 2.22 (br, 1H). ^{13}C NMR (150 MHz, $CDCl_3$) δ 151.4, 141.6, 139.7, 134.7, 129.1, 128.7, 128.7, 128.4, 128.4, 128.0, 127.7, 127.7, 127.1, 121.4, 58.8, 54.1, 51.6. HRMS (ESI-TOF) m/z calcd for $C_{23}H_{22}N_4$ $(M+H)^+$ 355.1517, found 355.1919.

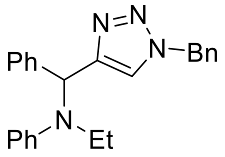

***N*-((1-Benzyl-1*H*-1,2,3-triazol-4-yl)(phenyl)methyl)-*N*-ethylaniline (S2)**

68 mg, 92% yield, yellow oil. ^{1}H NMR (400 MHz, $CDCl_3$) δ 7.37 – 7.31 (m, 3H), 7.30 – 7.20 (m, 6H), 7.18 – 7.11 (m, 4H), 6.78 (d, *J* = 8.4 Hz, 2H), 6.71 (t, *J* = 7.3 Hz, 1H), 6.26 (s, 1H), 5.53 – 5.43 (m, 2H), 3.41 – 3.26 (m, 2H), 0.87 (t, *J* = 7.0 Hz, 3H). ^{13}C NMR (100 MHz, $CDCl_3$) δ 148.6, 148.3, 139.8, 134.9, 129.1, 129.1, 128.6, 128.5, 128.2, 127.7, 127.4, 123.0, 117.7, 115.0, 60.0, 54.1, 42. 8, 13.5. (ESI-TOF) m/z calcd for $C_{25}H_{24}N_4O$ $(M+H)^+$ 369.2073; found, 369.2077.

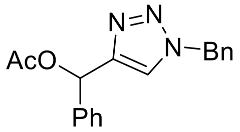

(1-Benzyl-1*H*-1,2,3-triazol-4-yl)(phenyl)methyl acetate (S3)

52 mg, 85% yield, yellow oil, ^{1}H NMR (400 MHz, $CDCl_3$) δ 7.35 (dd, *J* = 8.1, 1.3 Hz, 2H), 7.29 – 7.22 (m, 6H), 7.18 – 7.14 (m, 2H), 6.91 (s, 1H), 5.46 – 5.32 (m, 2 H), 2.02 (s, 3H). ^{13}C NMR (100 MHz, $CDCl_3$) δ 169.9, 147.7, 138.4, 134.4, 129.2, 128.8, 128.6, 128.4, 128.1, 127.2, 122.3, 70.1, 54.2, 21.2. HRMS (ESI-TOF) m/z calcd for $C_{18}H_{17}N_3O_2$ $(M+H)^+$308.1394, found 308.1399.

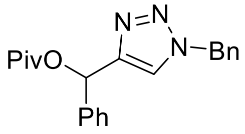

(1-Benzyl-1*H*-1,2,3-triazol-4-yl)(phenyl)methyl pivalate (S4)

58 mg, 83% yield, yellow oil, ^{1}H NMR (400 MHz, $CDCl_3$) δ 7.45 (d, *J* = 7.0 Hz, 2H), 7.39 – 7.31 (m, 6H), 7.28 – 7.21 (m, 2H), 6.99 (s, 1H), 5.50 (d, *J* = 5.2 Hz, 2H), 1.23 (s, 9H). ^{13}C NMR (100 MHz, $CDCl_3$) δ 177.1, 148.0, 138.7, 134.5, 129.1, 128.8, 128.6, 128.2, 128.0, 126.9, 122.1, 69.9, 54.1, 38.8, 27.1. HRMS (ESI-TOF) m/z calcd for $C_{18}H_{17}N_3O_2$ $(M+H)^+$350.1863, found 350.1868.

3.2 NMR spectra

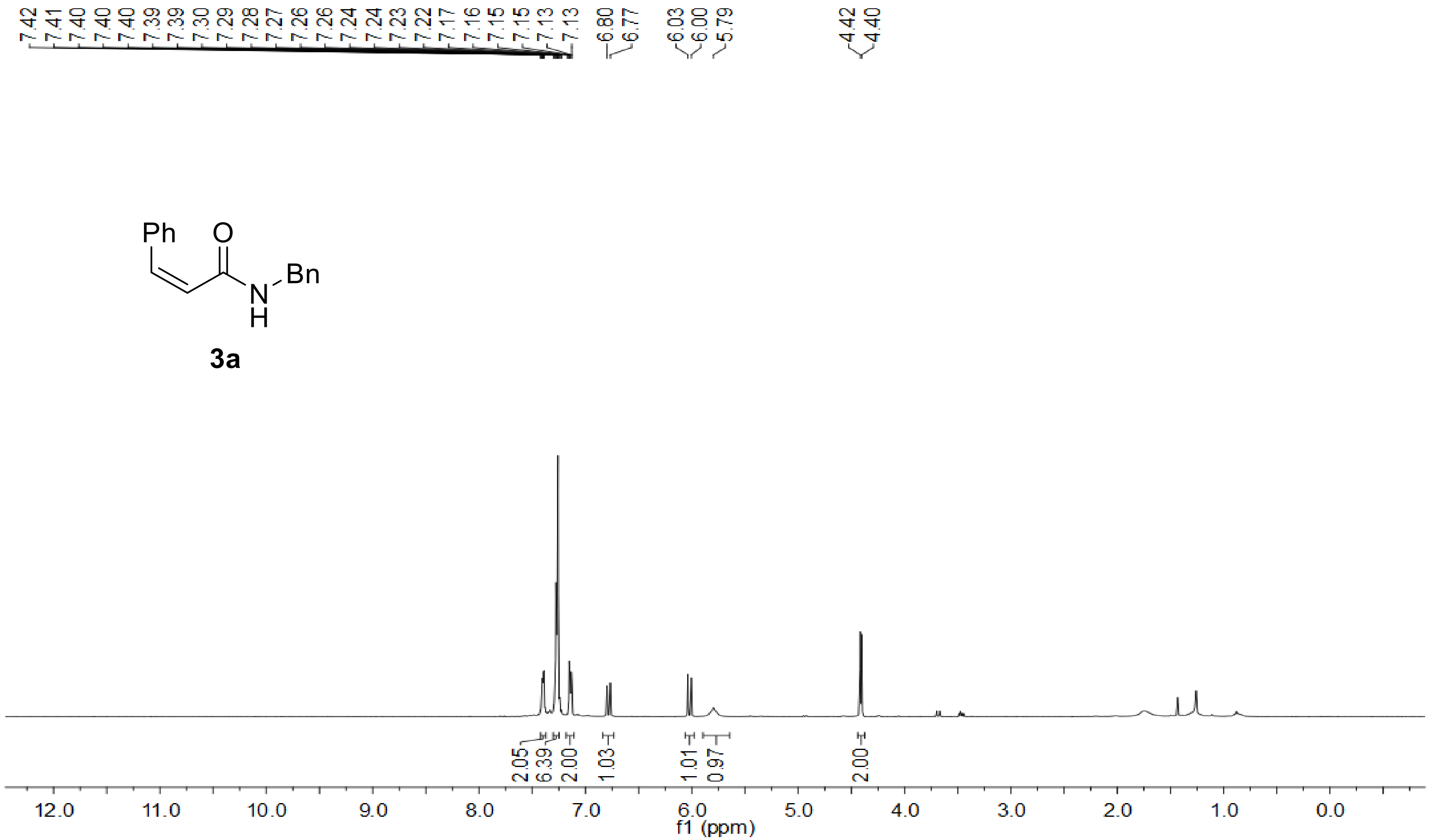

3a

Supplementary Figure 14. ^{1}H NMR spectrum of (*Z*)-*N*-benzyl-3-phenylacrylamide **(3a)** in $CDCl_3$ (400 MHz) at 23°C.

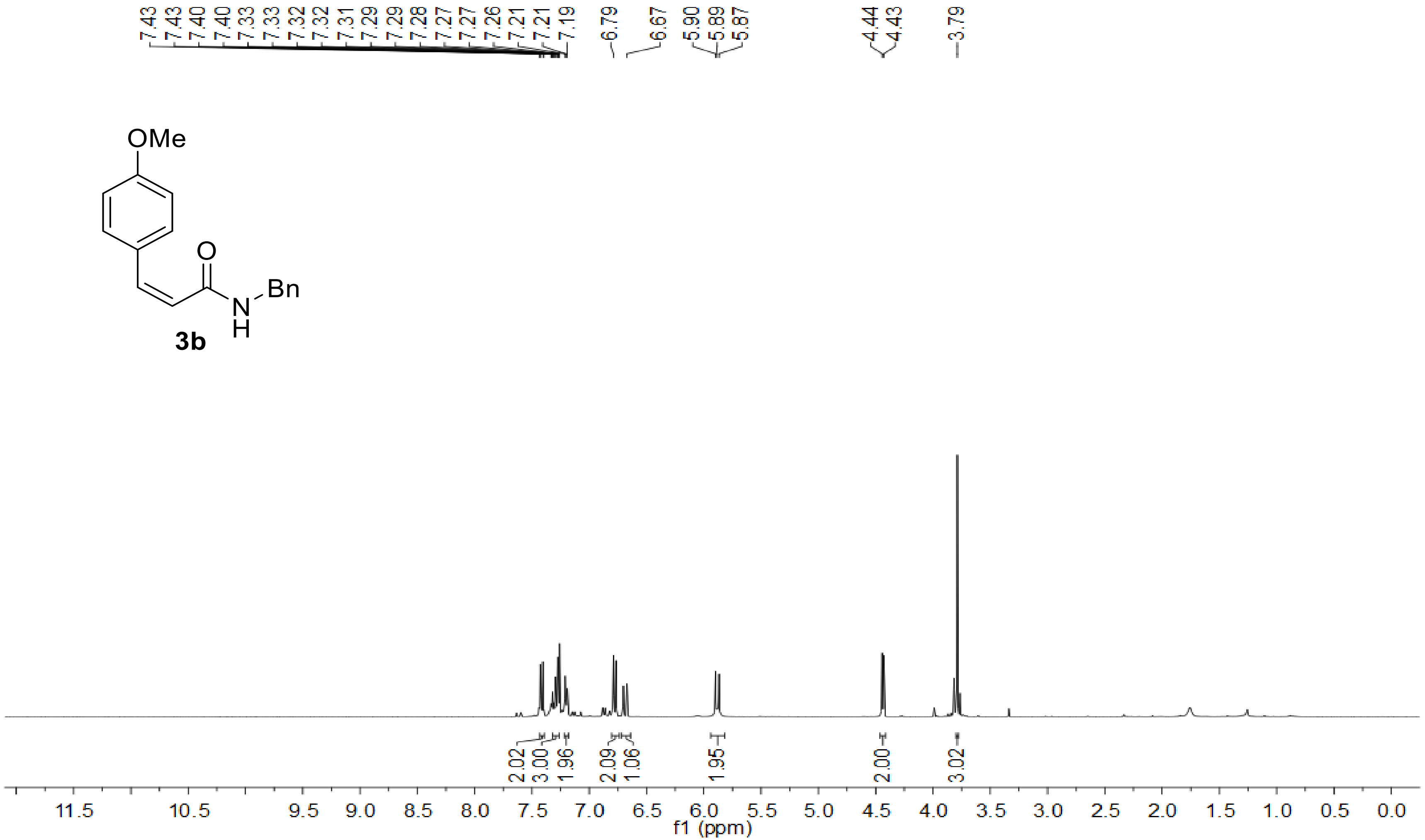

Supplementary Figure 15. ^{1}H NMR spectrum of (*Z*)-*N*-benzyl-3-(4-methoxyphenyl)acrylamide **(3b)** in $CDCl_3$ (400 MHz) at 23℃.

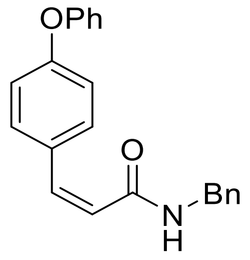

3c

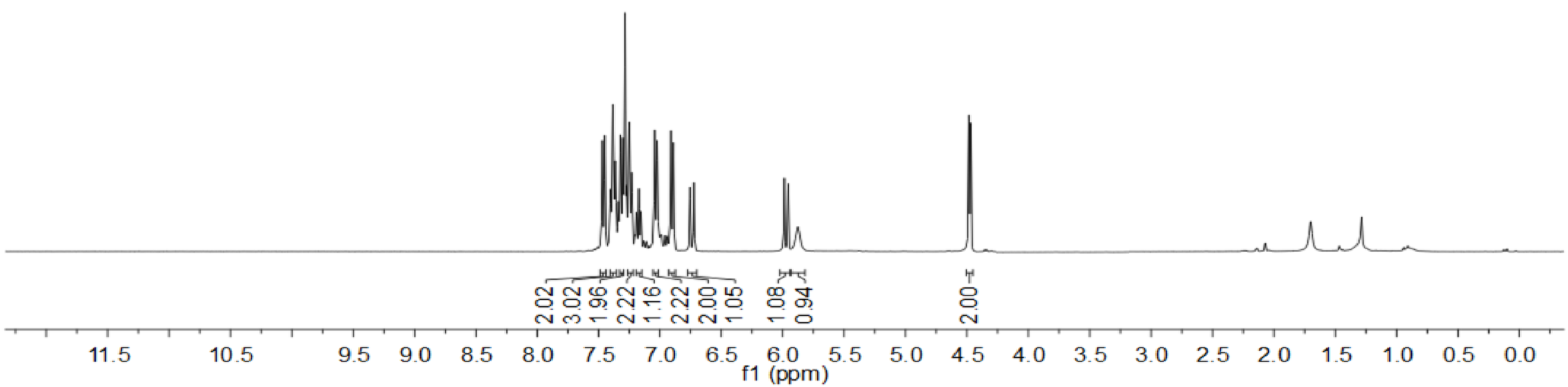

Supplementary Figure 16. ^{1}H NMR spectrum of (*Z*)-*N*-benzyl-3-(4-phenoxyphenyl)acrylamide **(3c)** in $CDCl_3$ (400 MHz) at 23°C.

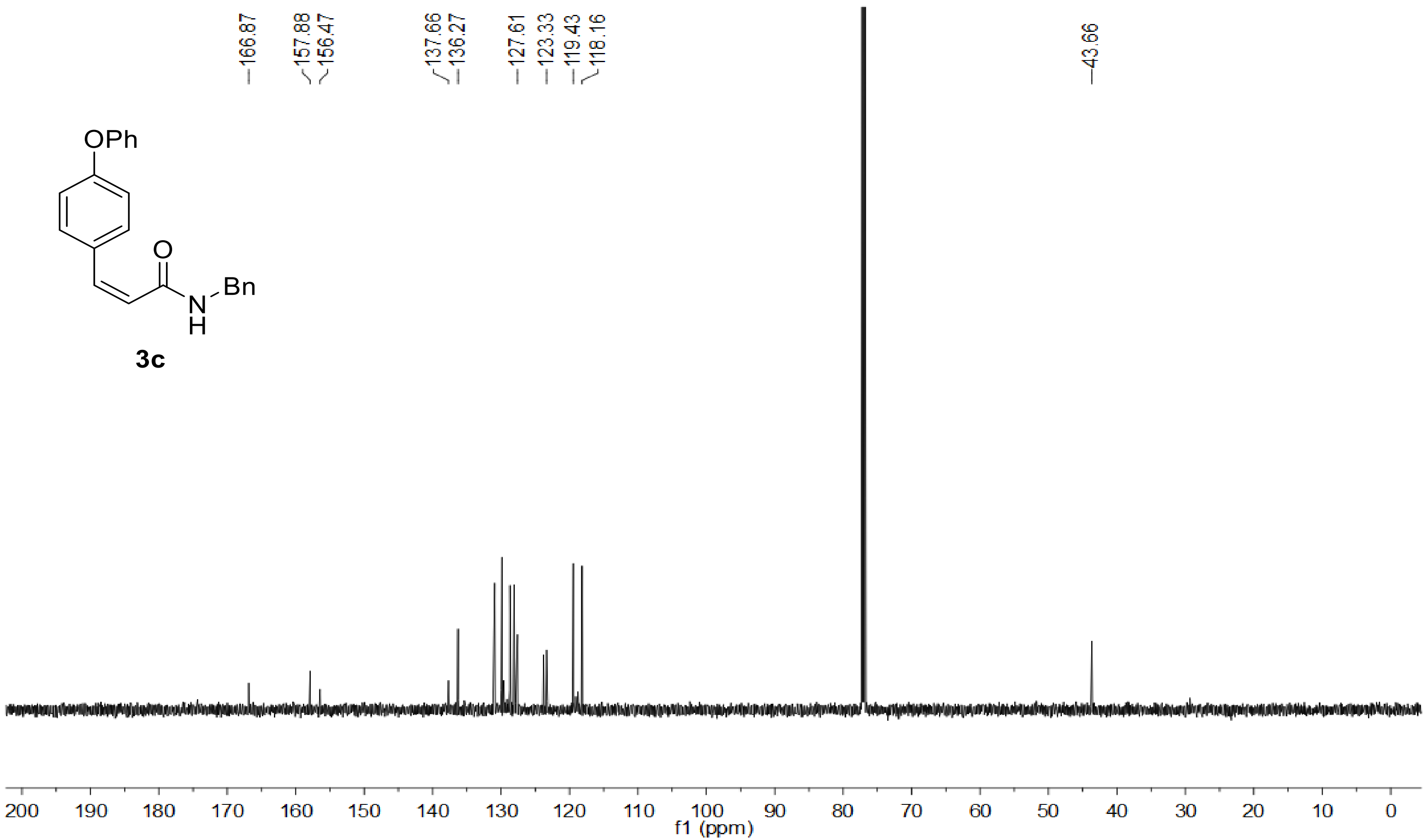

Supplementary Figure 17. ^{13}C NMR spectrum of (*Z*)-*N*-benzyl-3-(4-phenoxyphenyl)acrylamide **(3c)** in $CDCl_3$ (100 MHz) at 23°C.

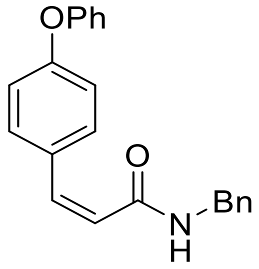

3c

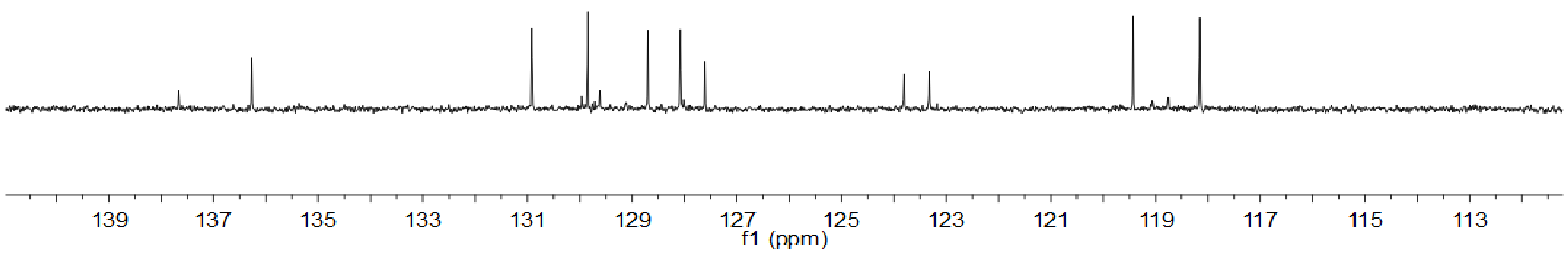

Supplementary Figure 18. Local magnification ^{13}C NMR spectrum of (*Z*)-*N*-benzyl-3-(4-phenoxyphenyl)acrylamide **(3c)** in $CDCl_3$ (100 MHz) at 23℃.

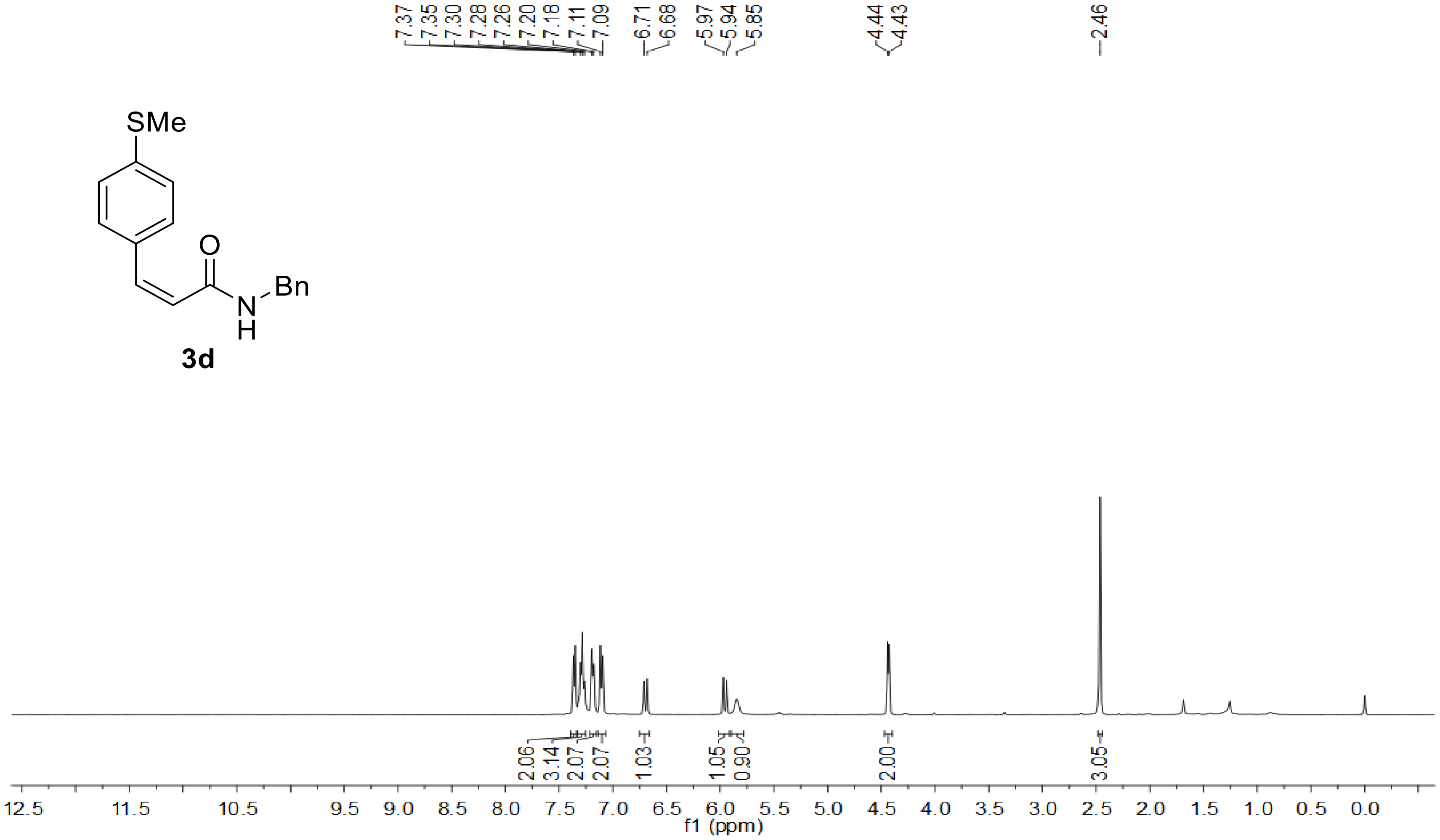

Supplementary Figure 19. ^{1}H NMR spectrum of (*Z*)-*N*-benzyl-3-(4-(methylthio)phenyl)acrylamide **(3d)** in $CDCl_3$ (400 MHz) at 23℃.

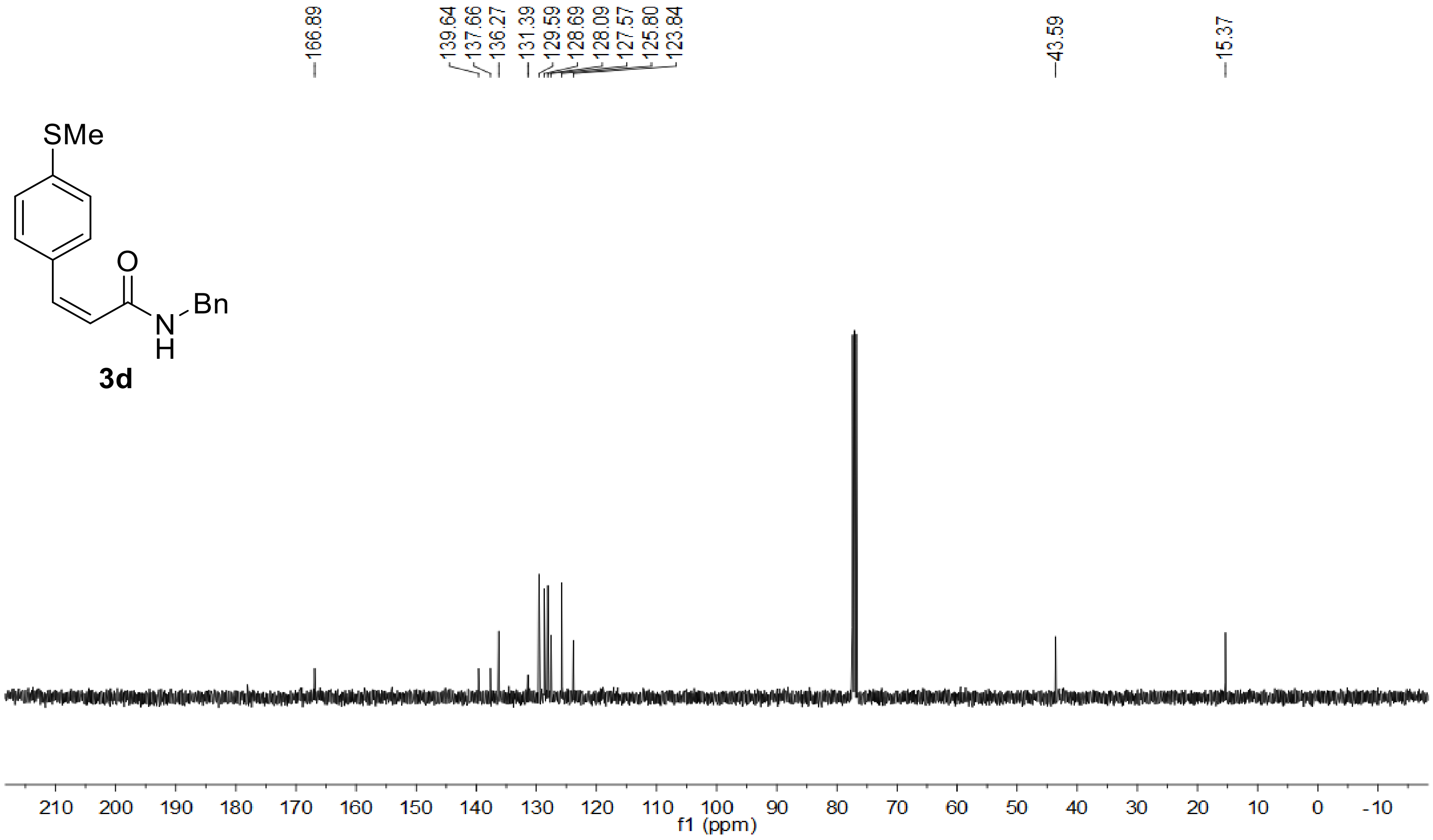

Supplementary Figure 20. ^{13}C NMR spectrum of (*Z*)-*N*-benzyl-3-(4-(methylthio)phenyl)acrylamide **(3d)** in $CDCl_3$ (100 MHz) at 23℃.

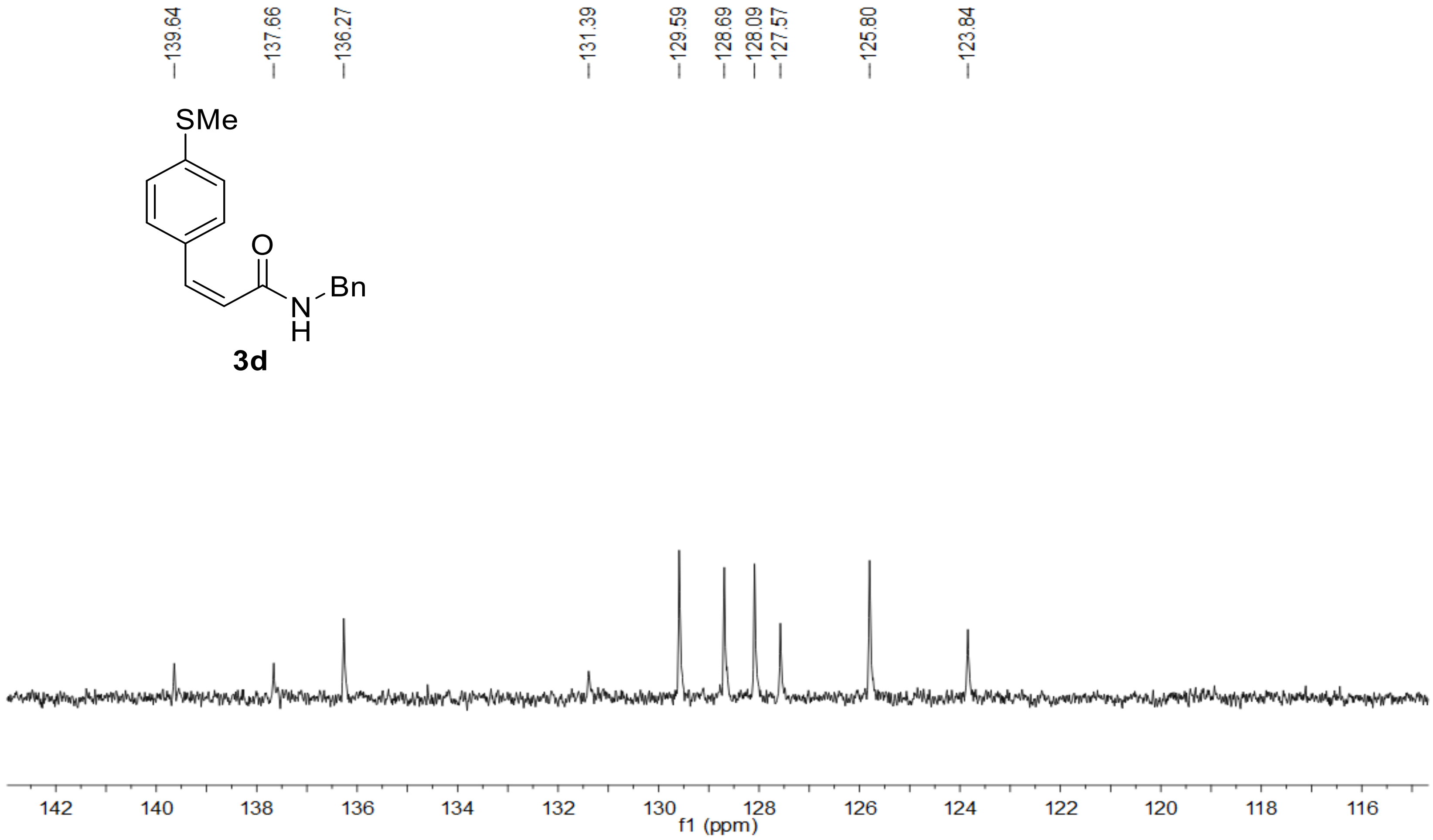

Supplementary Figure 21. Local magnification ^{13}C NMR spectrum of (*Z*)-*N*-benzyl-3-(4-(methylthio)phenyl)acrylamide **(3d)** in $CDCl_3$ (100 MHz) at 23℃.

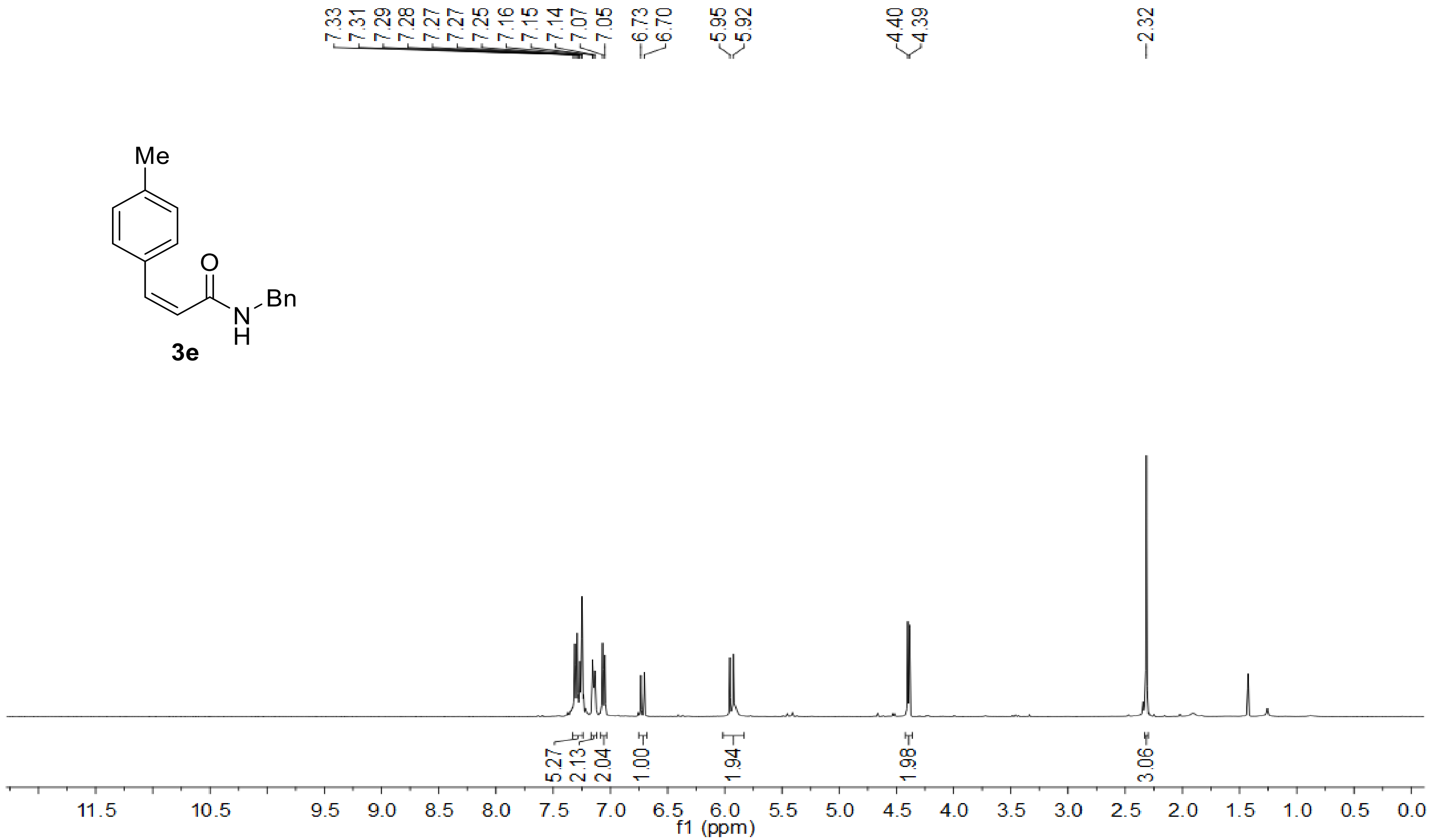

Supplementary Figure 22. ^{1}H NMR spectrum of (*Z*)-*N*-benzyl-3-(*p*-tolyl)acrylamide **(3e)** in $CDCl_3$ (400 MHz) at 23℃.

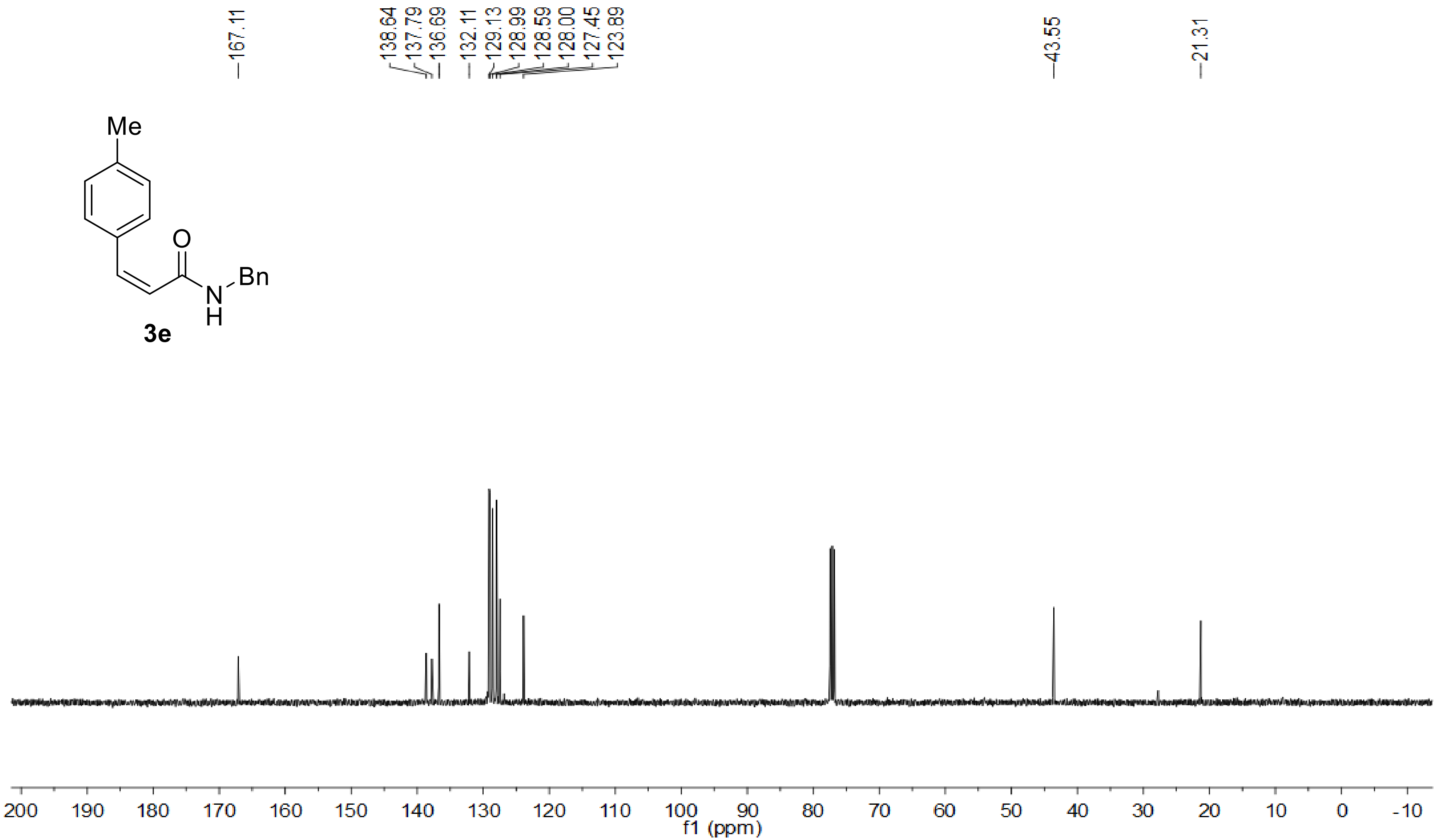

Supplementary Figure 23. ^{13}C NMR spectrum of (*Z*)-*N*-benzyl-3-(*p*-tolyl)acrylamide **(3e)** in $CDCl_3$ (100 MHz) at 23°C.

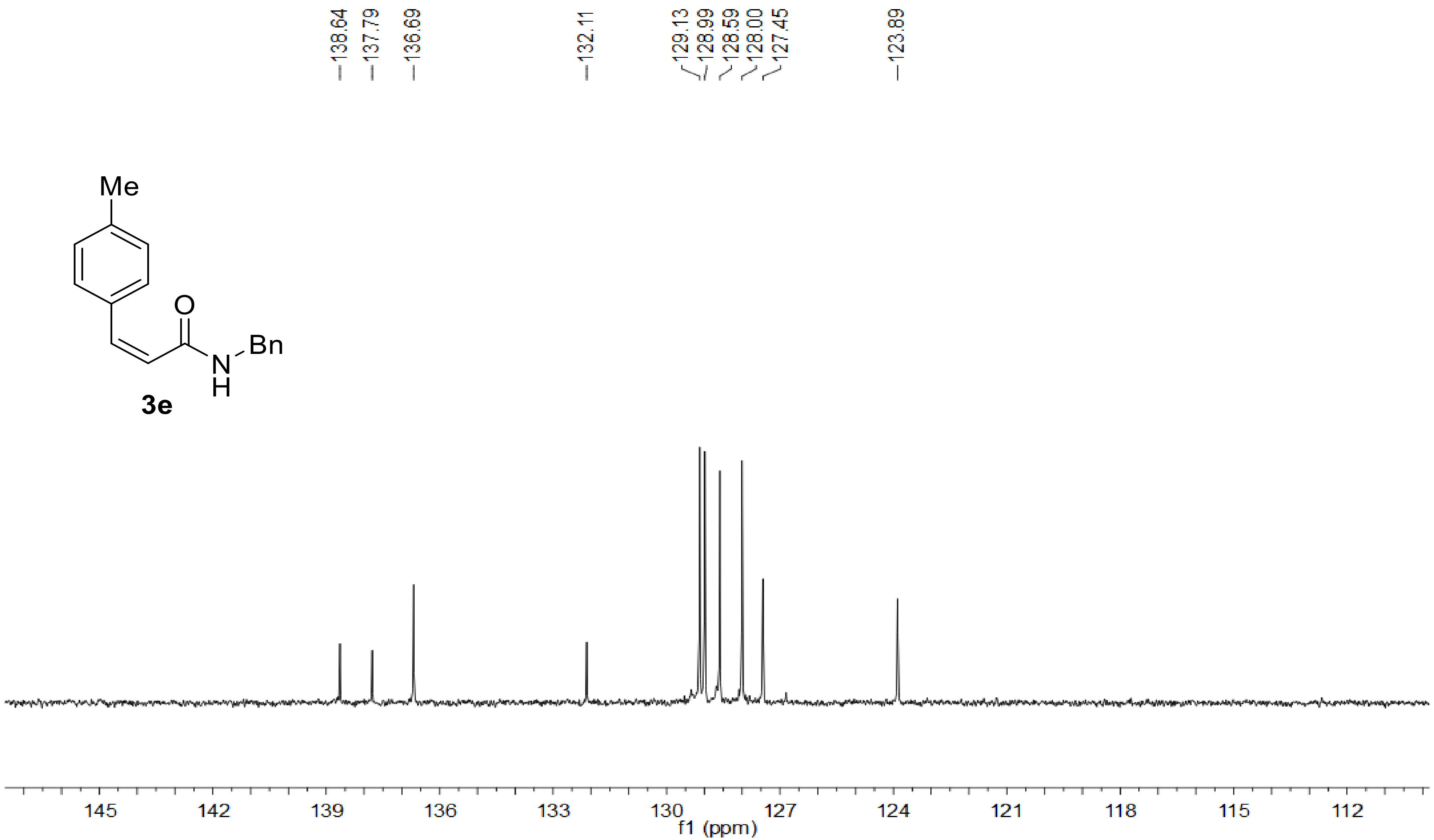

Supplementary Figure 24. Local magnification ^{13}C NMR spectrum of (*Z*)-*N*-benzyl-3-(*p*-tolyl)acrylamide **(3e)** in $CDCl_3$ (100 MHz) at 23°C.

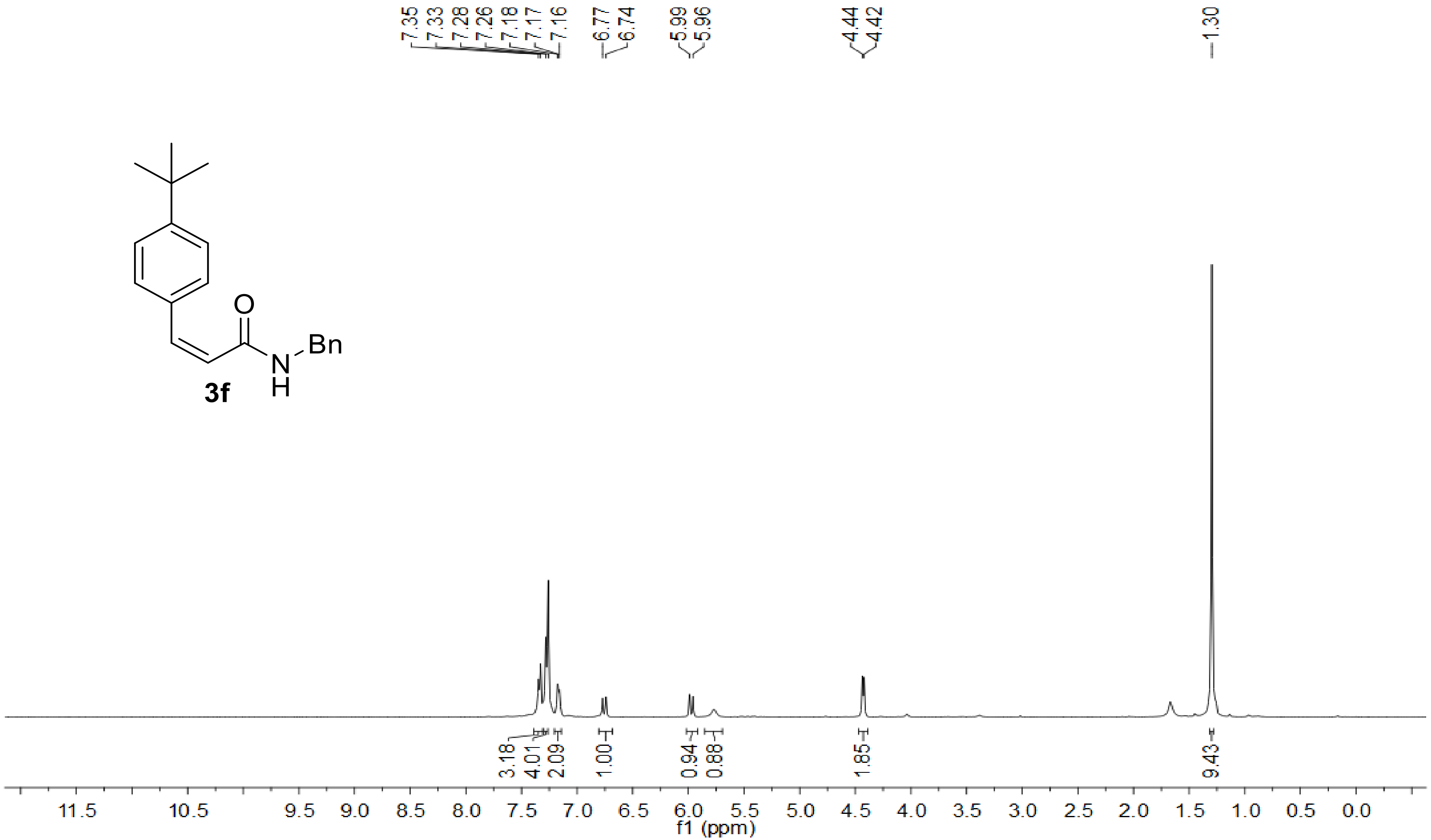

Supplementary Figure 25. ^{1}H NMR spectrum of (*Z*)-*N*-benzyl-3-(4-(*tert*-butyl)phenyl)acrylamide **(3f)** in $CDCl_3$ (400 MHz) at 23℃.

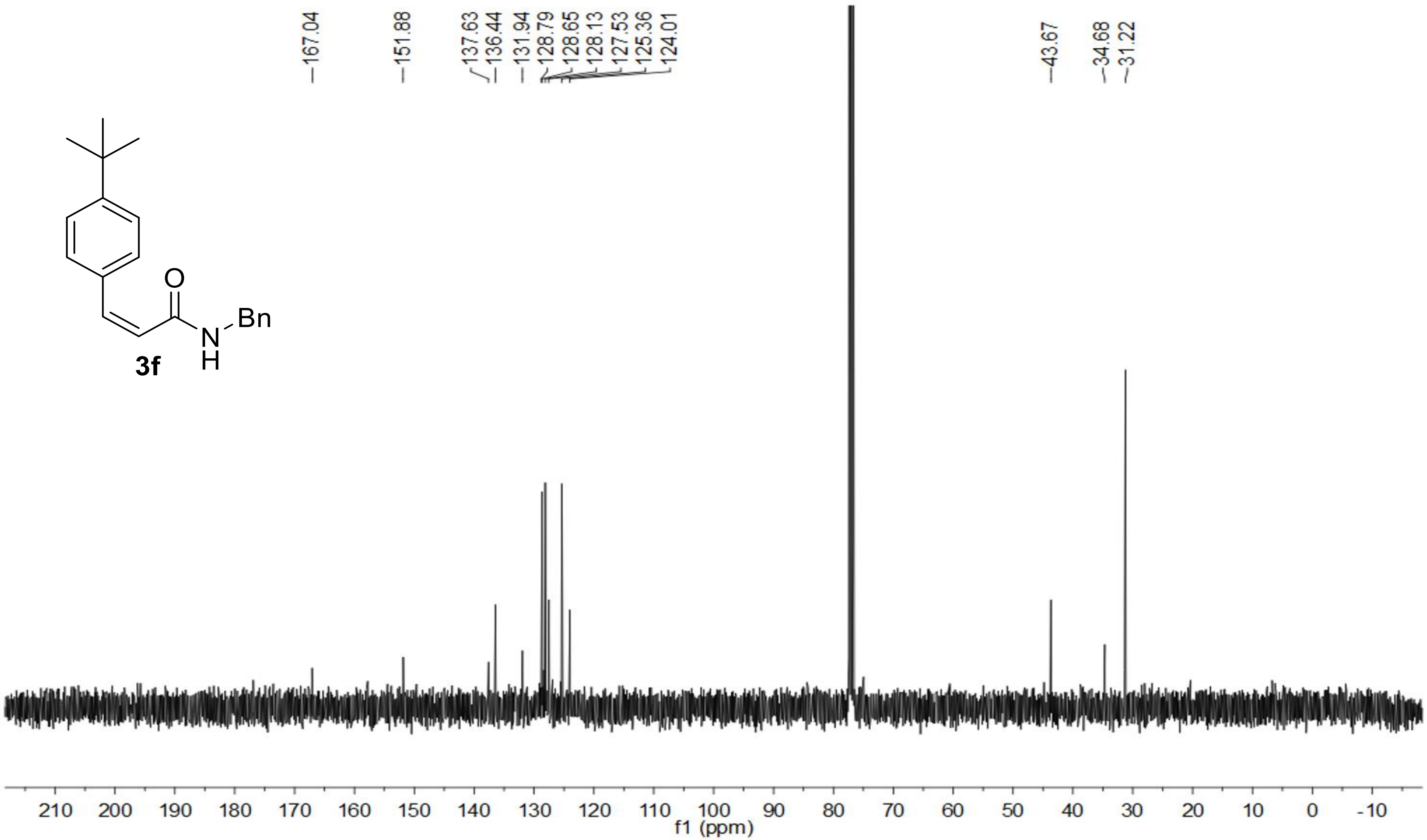

Supplementary Figure 26. ^{13}C NMR spectrum of (*Z*)-*N*-benzyl-3-(4-(*tert*-butyl)phenyl)acrylamide **(3f)** in $CDCl_3$ (100 MHz) at 23°C.

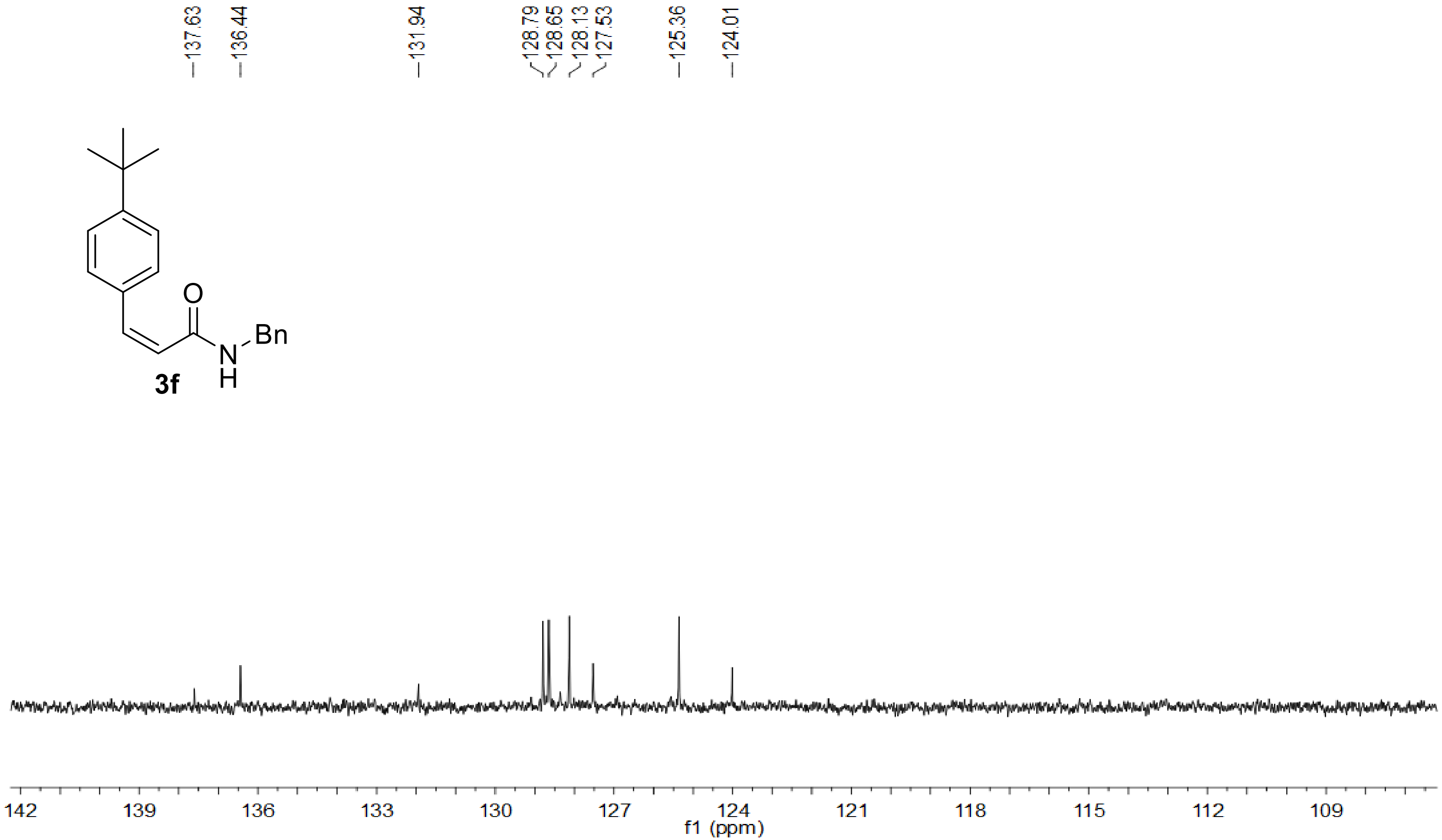

Supplementary Figure 27. Local magnification ^{13}C NMR spectrum of (*Z*)-*N*-benzyl-3-(4-(*tert*-butyl)phenyl)acrylamide **(3f)** in $CDCl_3$ (100 MHz) at 23℃.

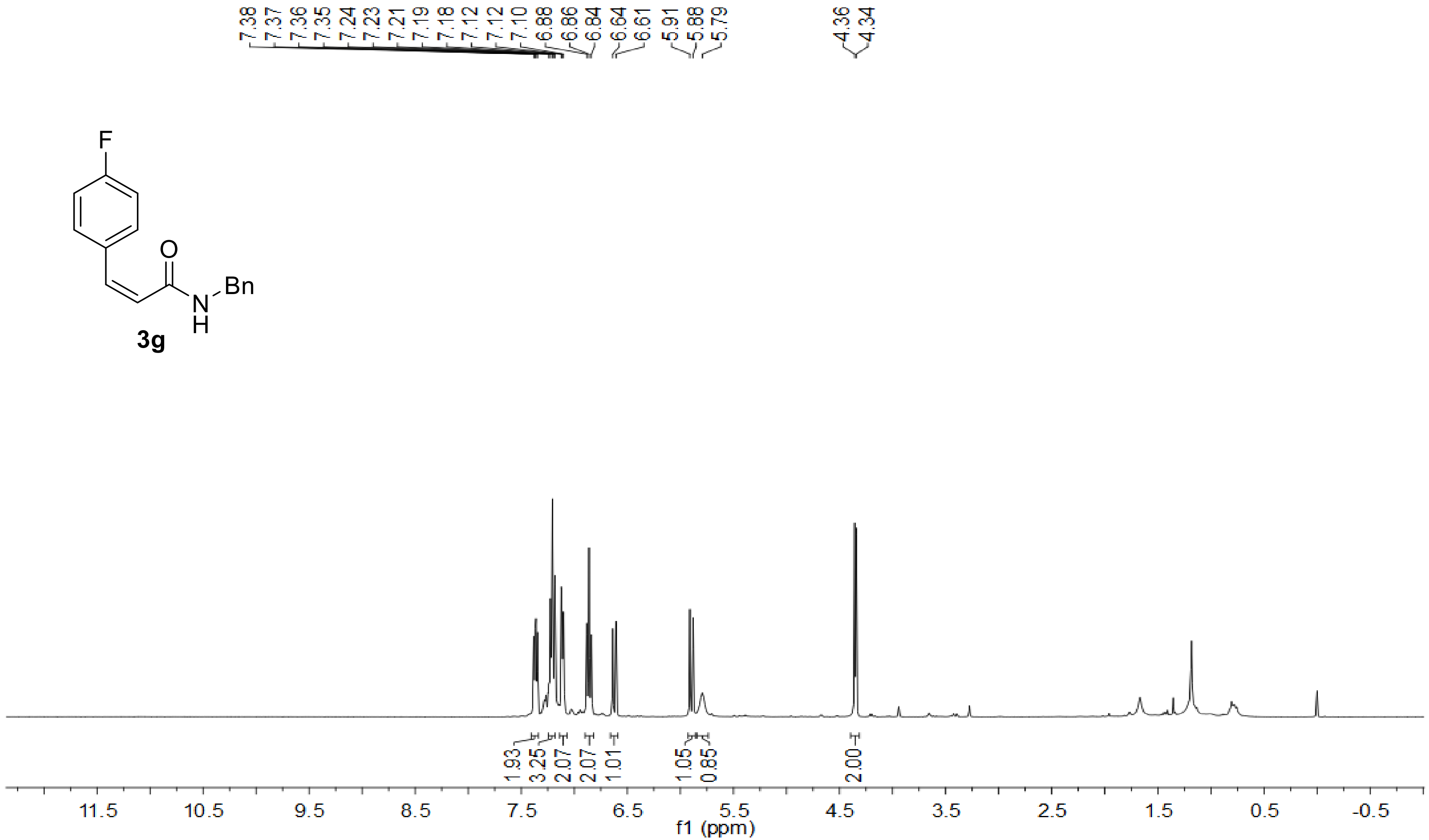

3g

Supplementary Figure 28. ^{1}H NMR spectrum of (*Z*)-*N*-benzyl-3-(4-fluorophenyl)acrylamide **(3g)** in $CDCl_3$ (400 MHz) at 23°C.

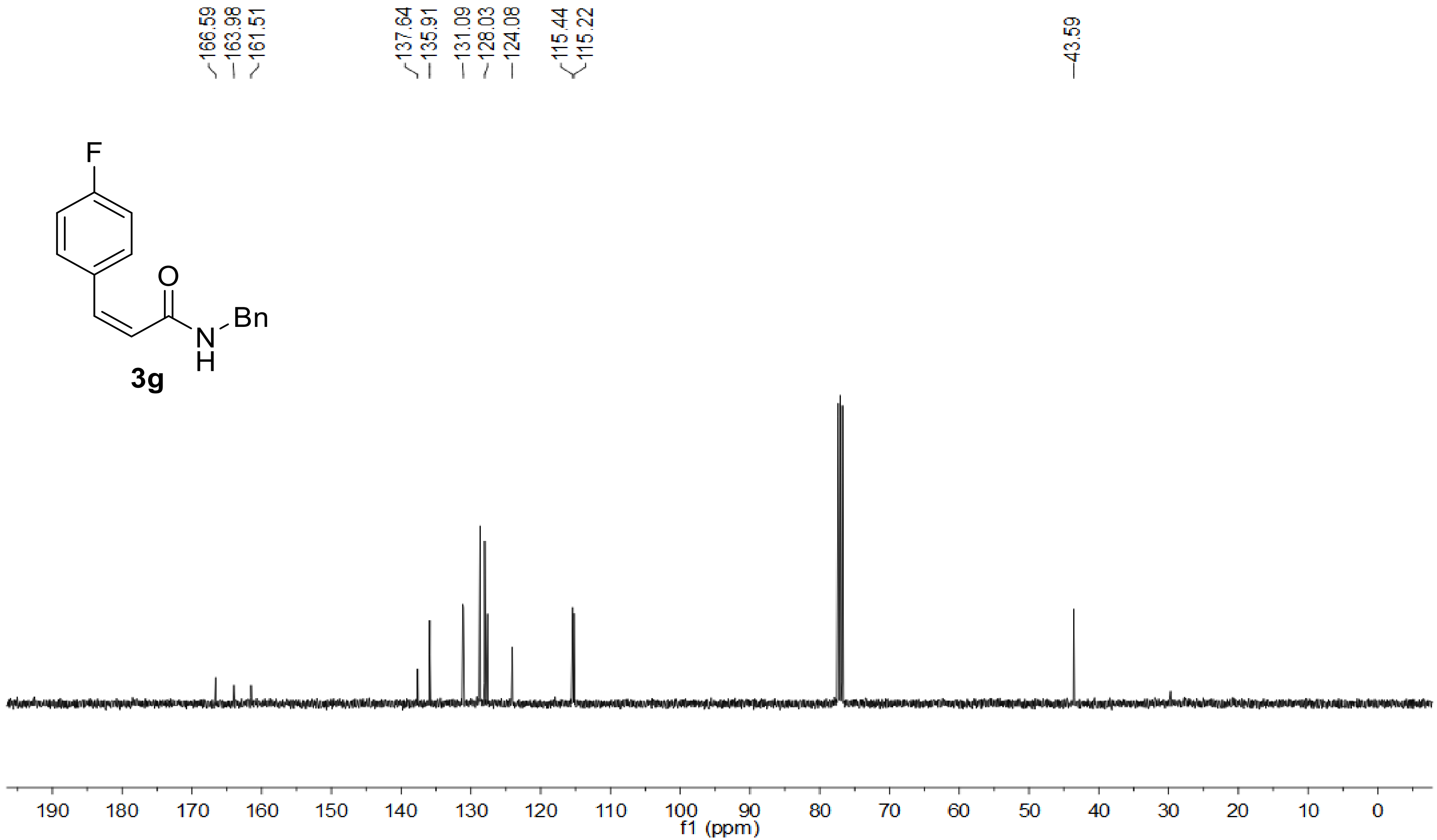

Supplementary Figure 29. ^{13}C NMR spectrum of (*Z*)-*N*-benzyl-3-(4-fluorophenyl)acrylamide **(3g)** in $CDCl_3$ (100 MHz) at 23℃.

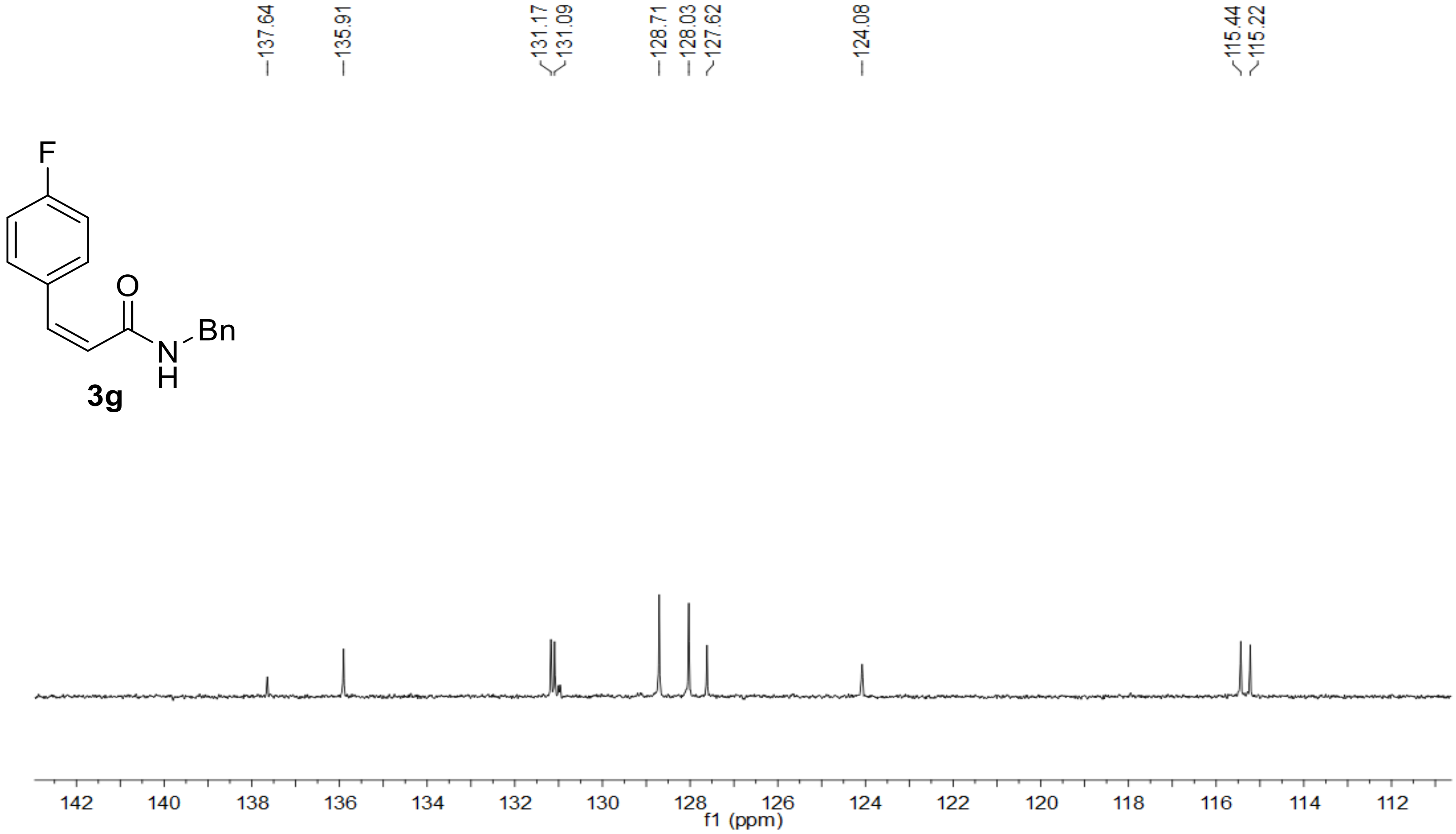

Supplementary Figure 30. Local magnification ^{13}C NMR spectrum of (*Z*)-*N*-benzyl-3-(4-fluorophenyl)acrylamide **(3g)** in $CDCl_3$ (100 MHz) at 23℃.

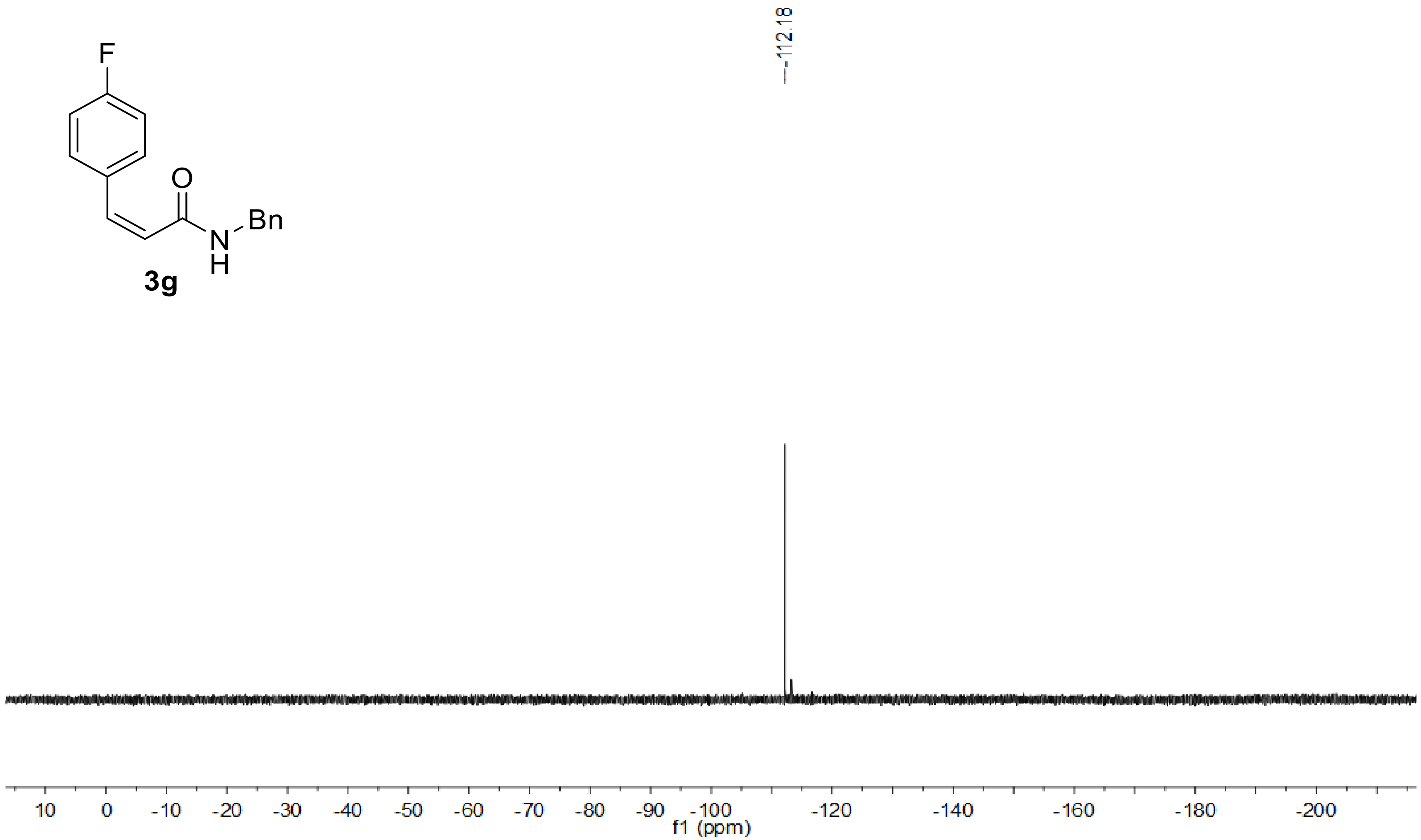

Supplementary Figure 31. ^{19}F NMR spectrum of (*Z*)-*N*-benzyl-3-(4-fluorophenyl)acrylamide **(3g)** in $CDCl_3$ (565 MHz) at 23℃.

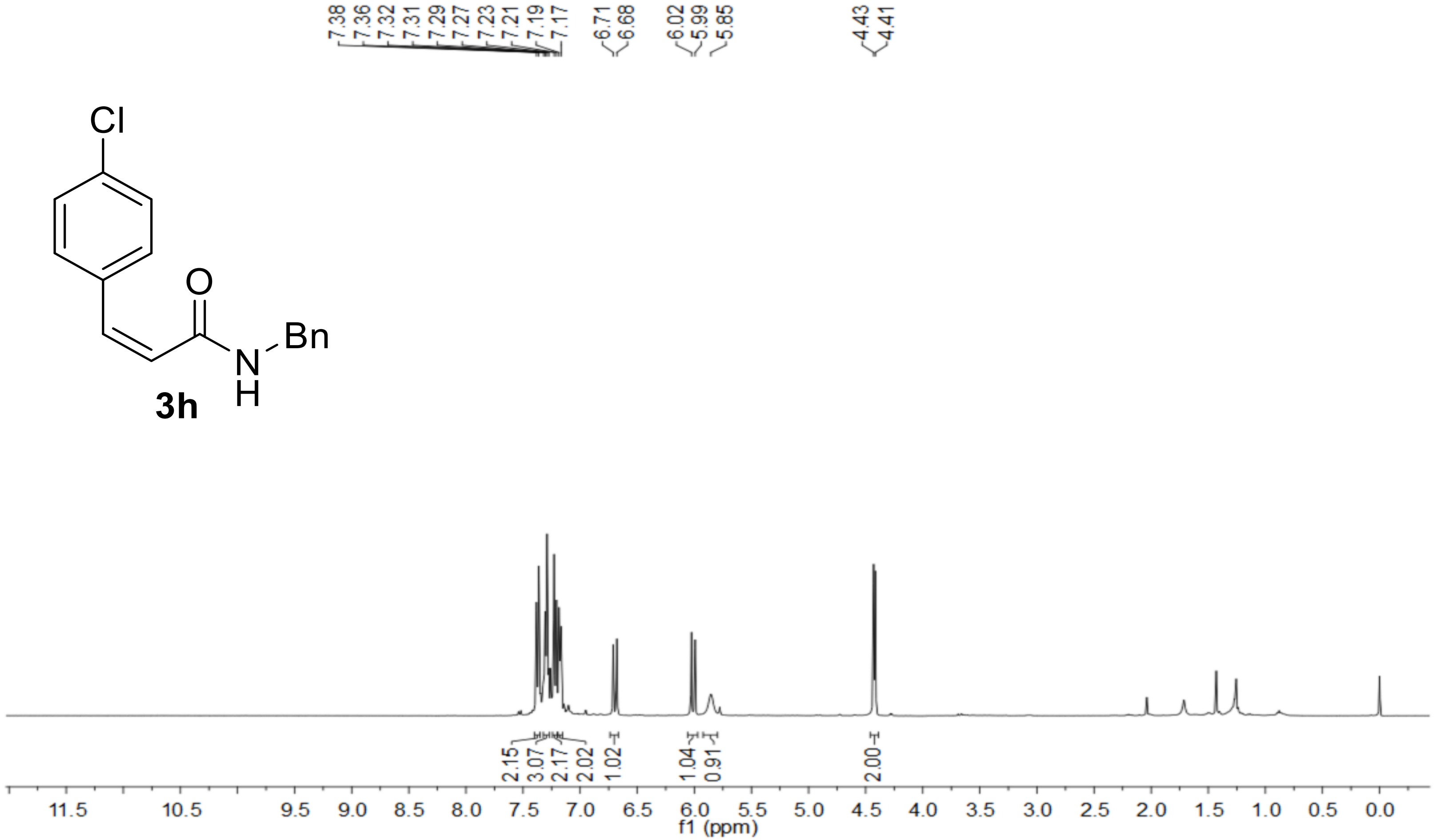

Supplementary Figure 32. ^{1}H NMR spectrum of (*Z*)-*N*-benzyl-3-(4-chlorophenyl)acrylamide **(3h)** in $CDCl_3$ (400 MHz) at 23℃.

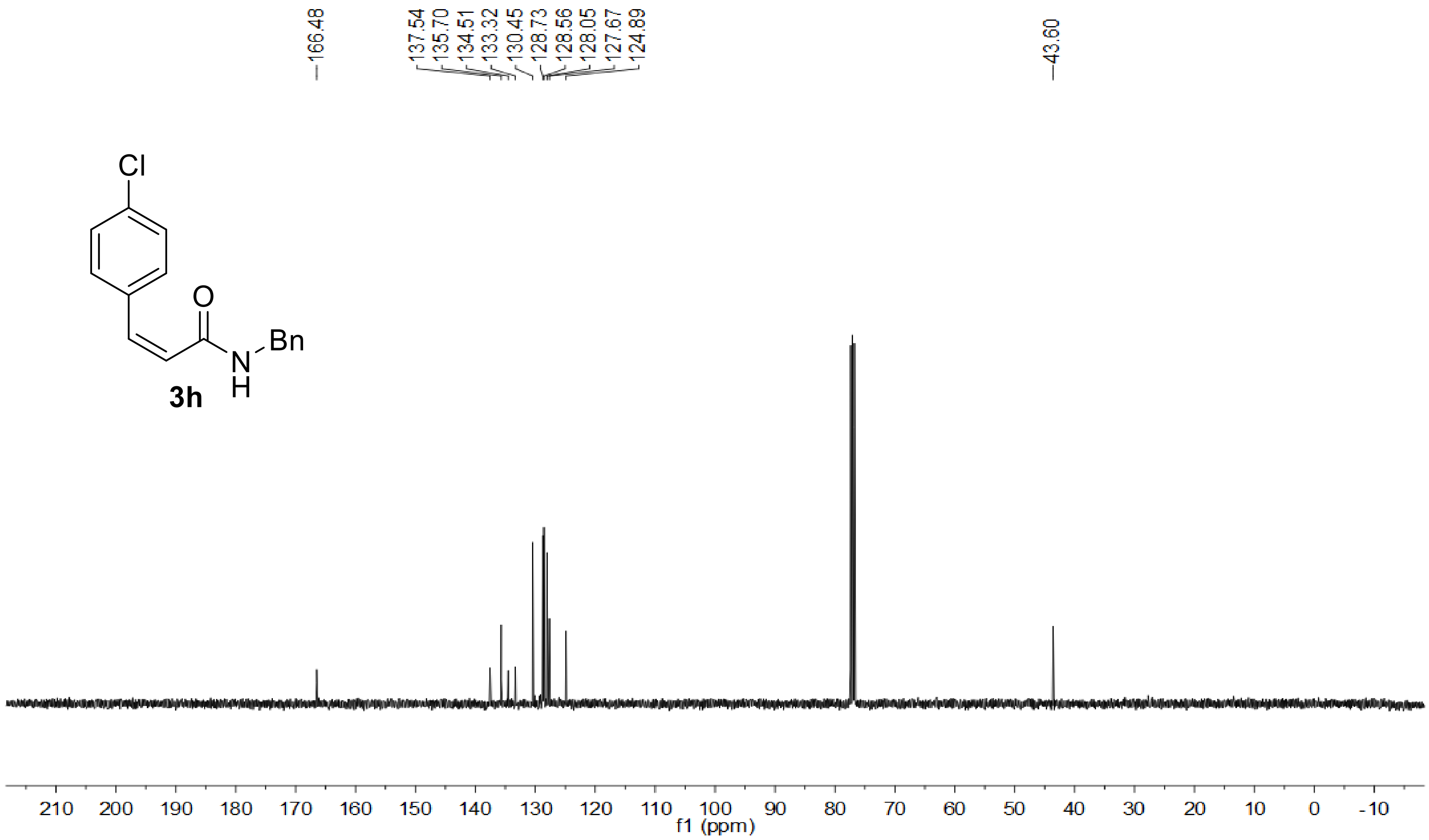

Supplementary Figure 33. ^{13}C NMR spectrum of (*Z*)-*N*-benzyl-3-(4-chlorophenyl)acrylamide **(3h)** in $CDCl_3$ (100 MHz) at 23°C.

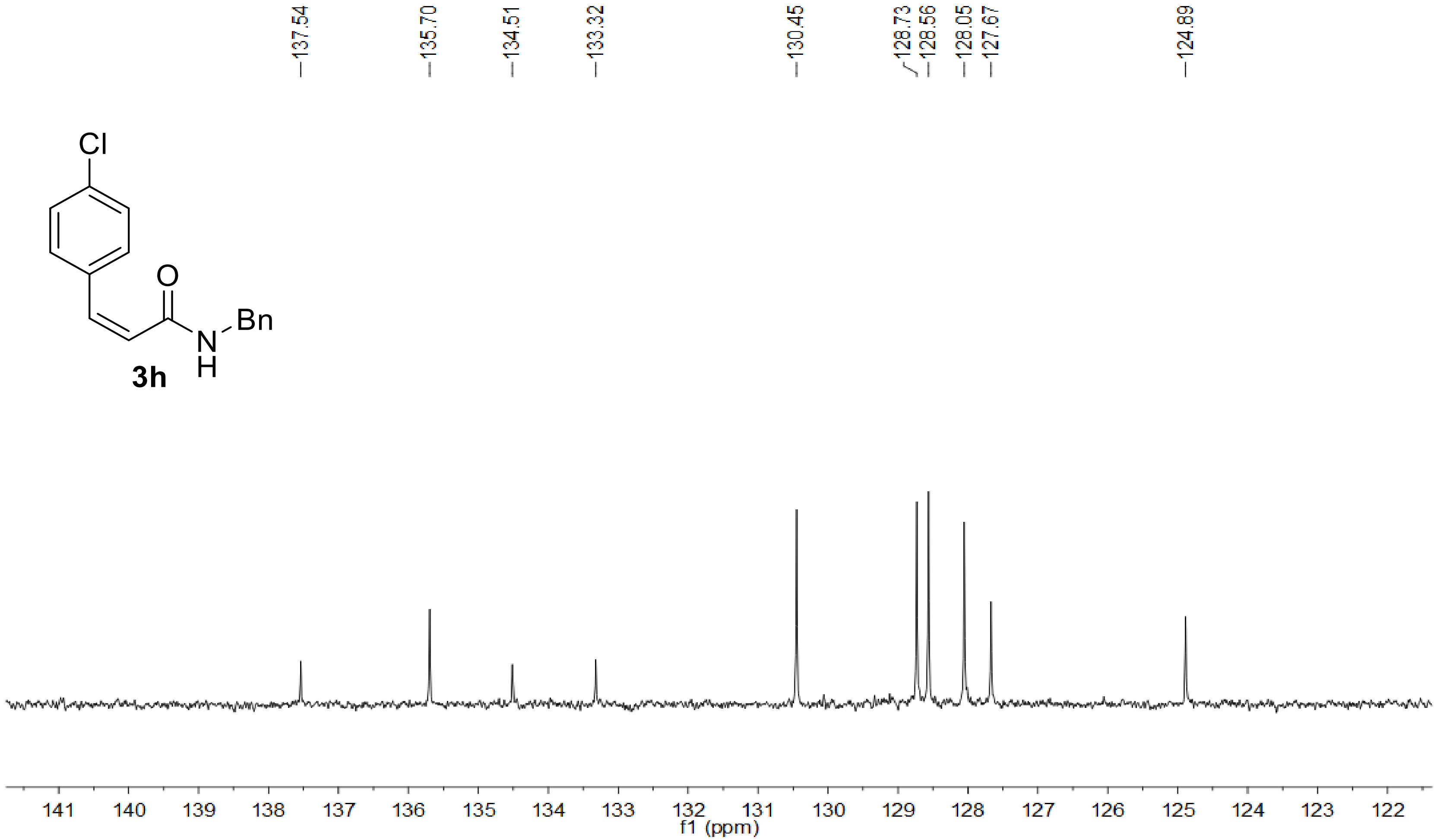

Supplementary Figure 34. Local magnification ^{13}C NMR spectrum of (*Z*)-*N*-benzyl-3-(4-chlorophenyl)acrylamide **(3h)** in $CDCl_3$ (100 MHz) at 23℃.

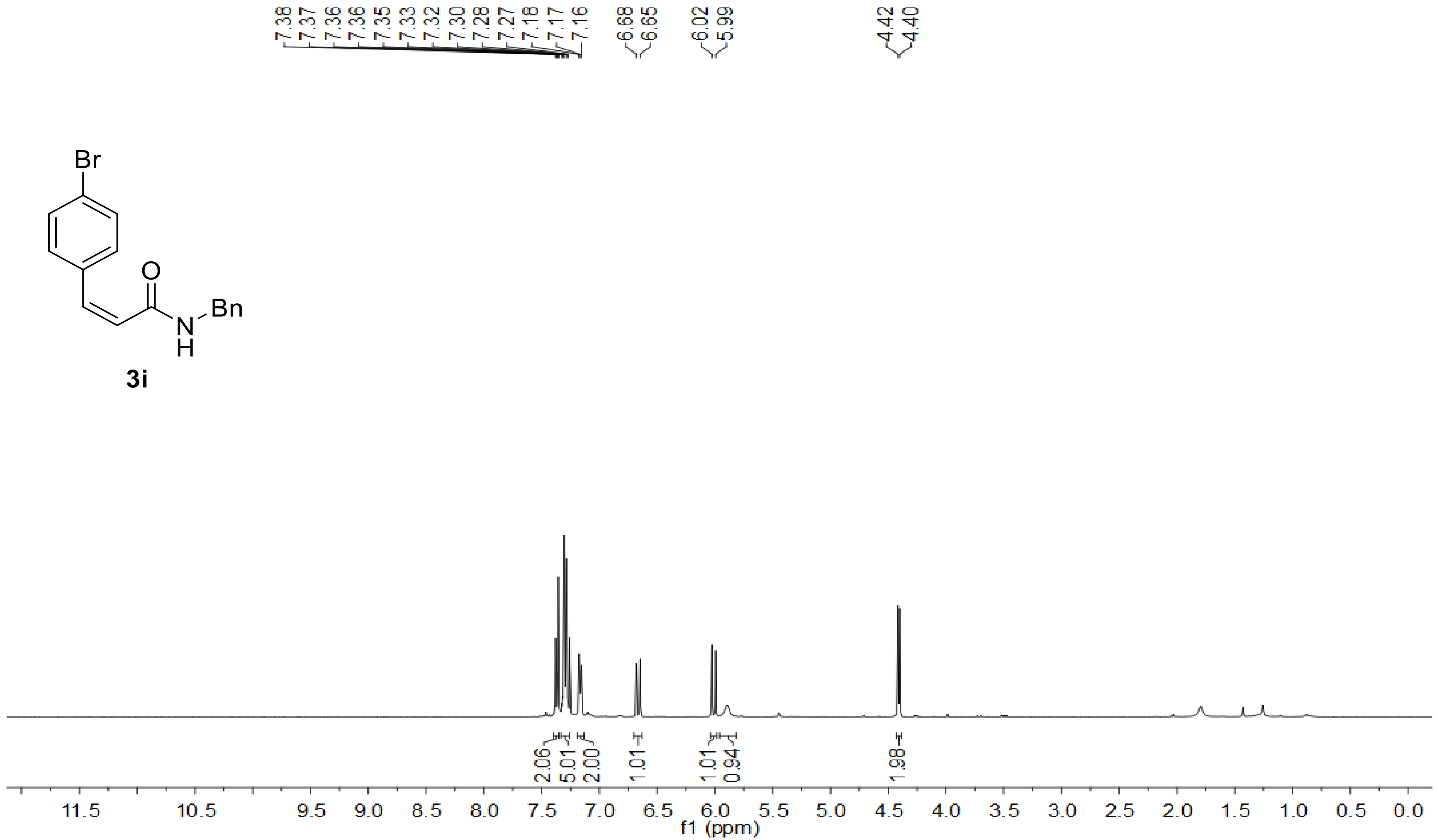

Supplementary Figure 35. ^{1}H NMR spectrum of **(***Z*)-*N*-benzyl-3-(4-bromophenyl)acrylamide **(3i)** in $CDCl_3$ (400 MHz) at 23°C.

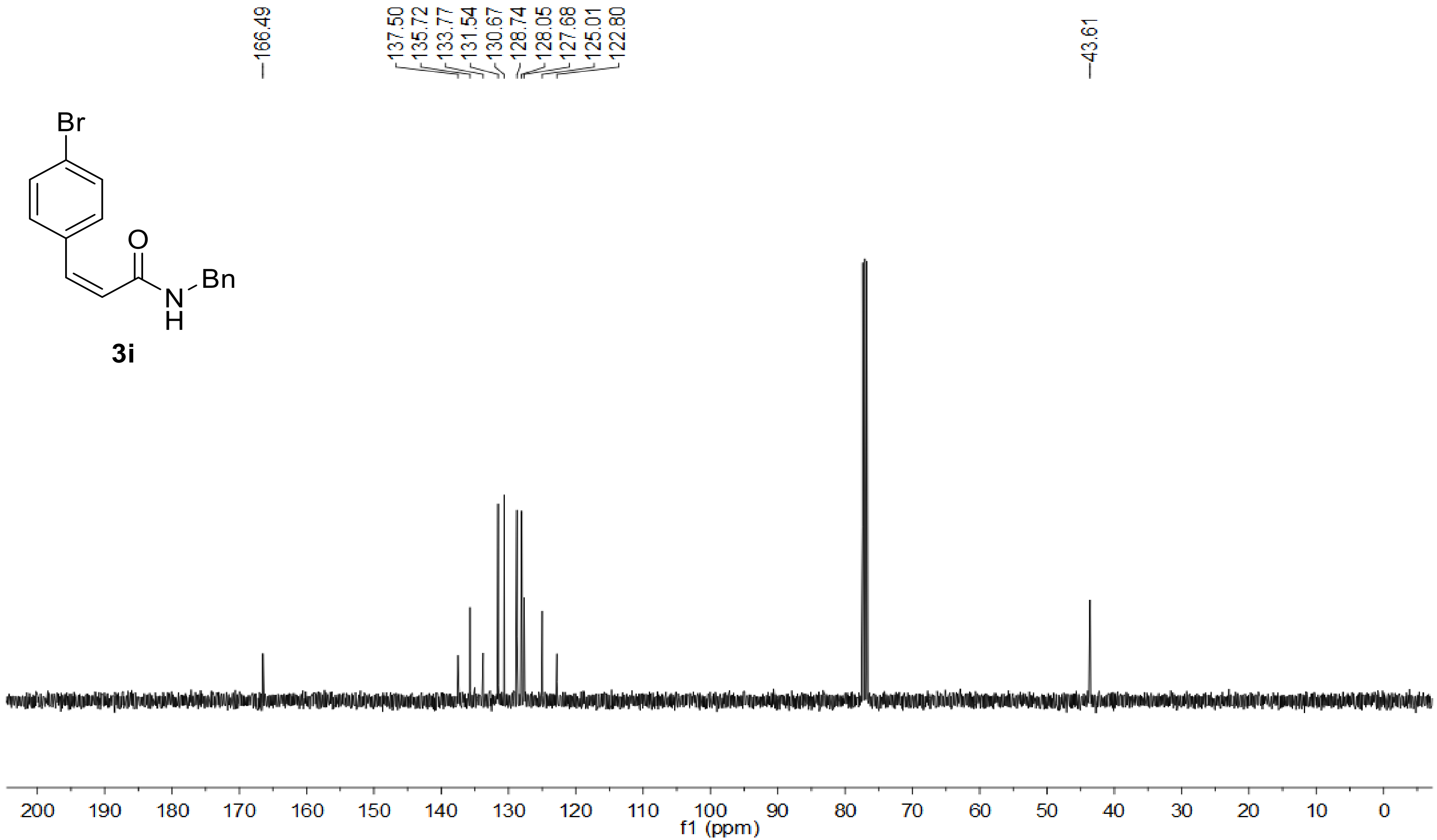

Supplementary Figure 36. ^{13}C NMR spectrum of **(***Z***)**-*N*-benzyl-3-(4-bromophenyl)acrylamide **(3i)** in $CDCl_3$ (100 MHz) at 23℃.

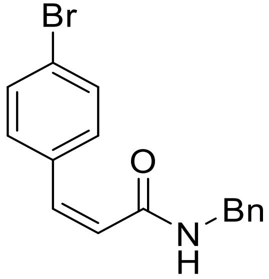

3i

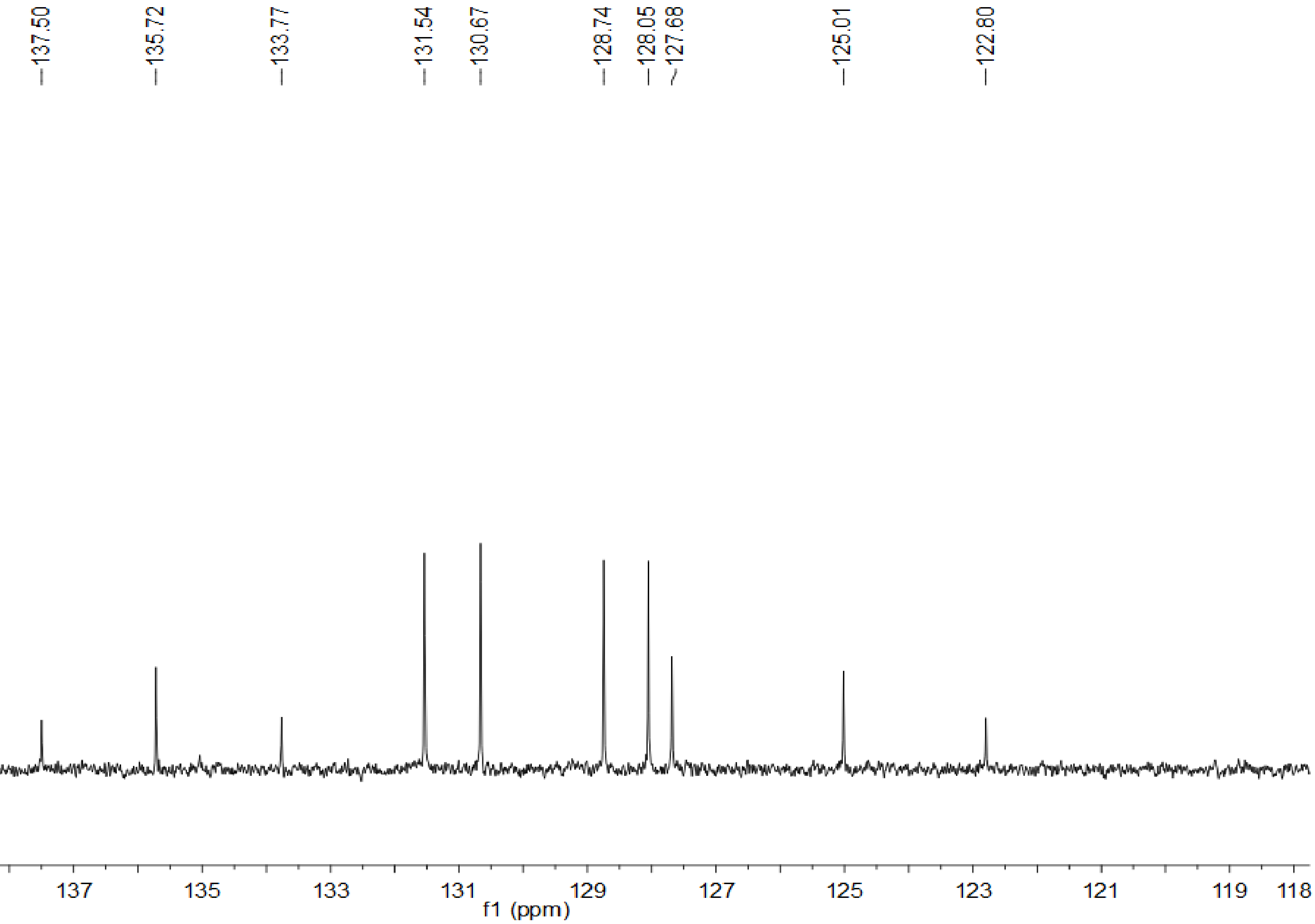

Supplementary Figure 37. Local magnification ^{13}C NMR spectrum of **(***Z***)**-*N*-benzyl-3-(4-bromophenyl)acrylamide **(3i)** in $CDCl_3$ (100 MHz) at 23℃.

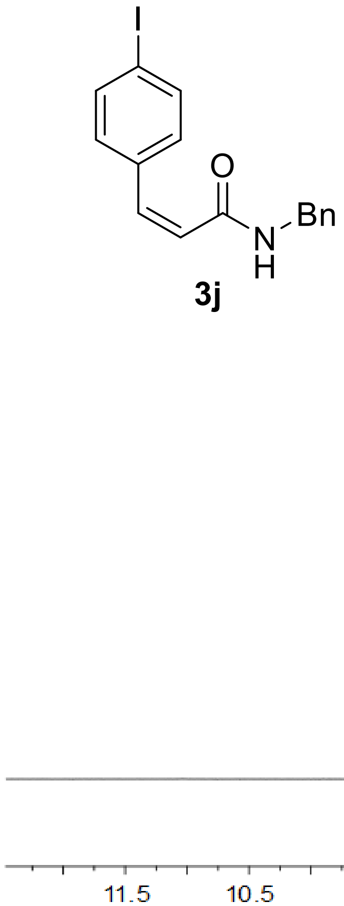

3j

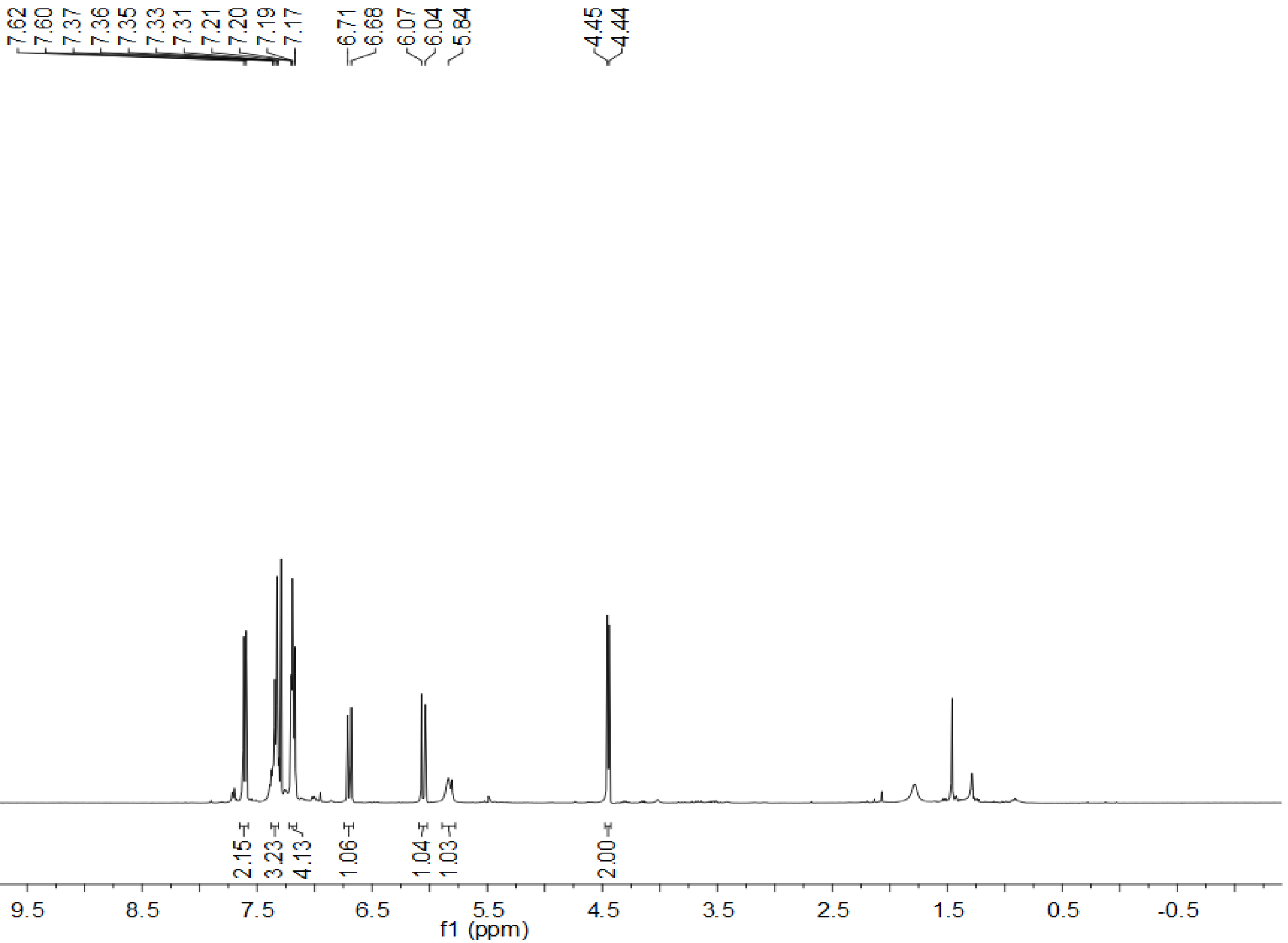

Supplementary Figure 38. ^{1}H NMR spectrum of (*Z*)-*N*-benzyl-3-(4-iodophenyl)acrylamide **(3j)** in $CDCl_3$ (400 MHz) at 23℃.

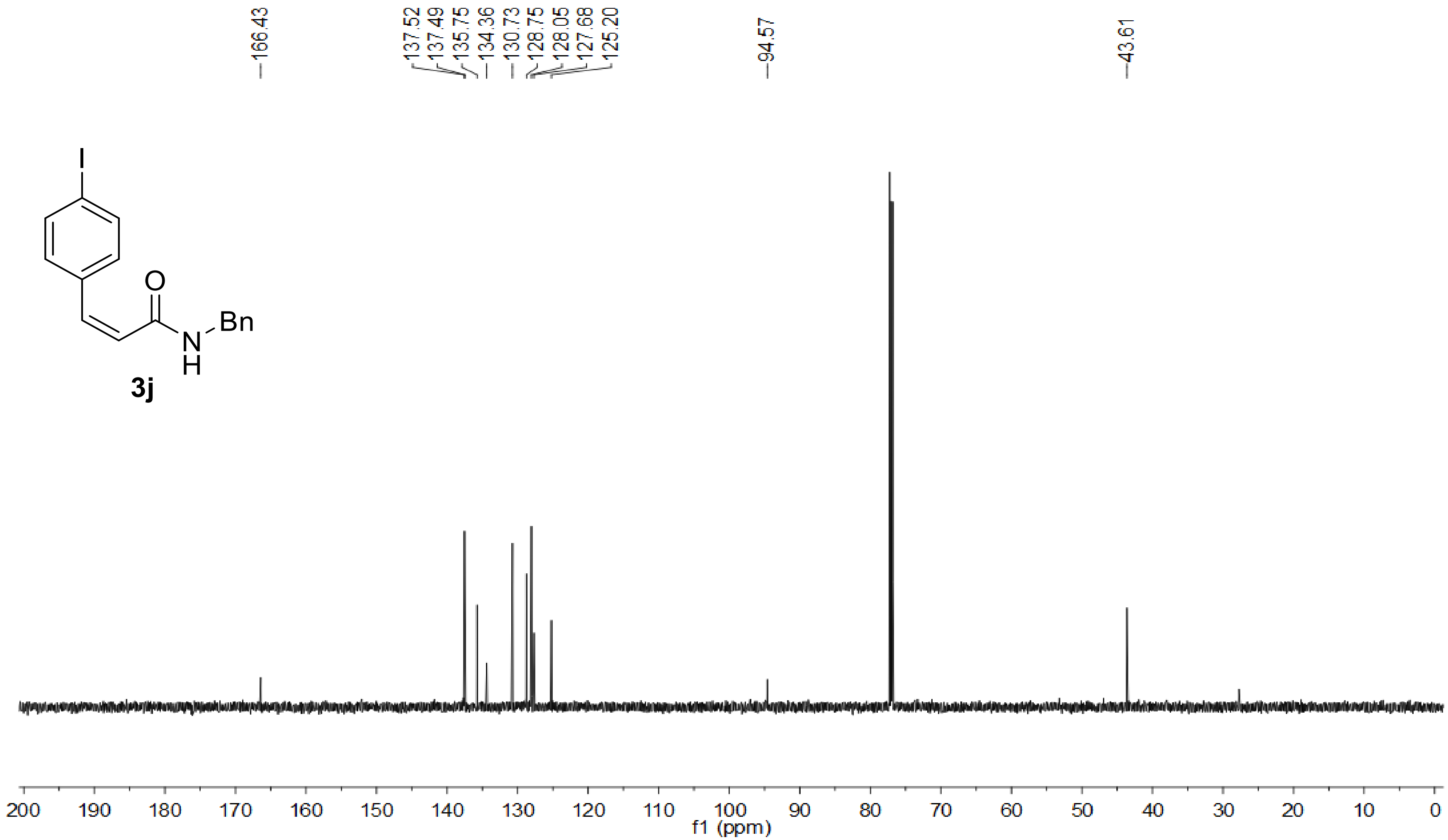

Supplementary Figure 39. ^{13}C NMR spectrum of (*Z*)-*N*-benzyl-3-(4-iodophenyl)acrylamide **(3j)** in $CDCl_3$ (100 MHz) at 23°C.

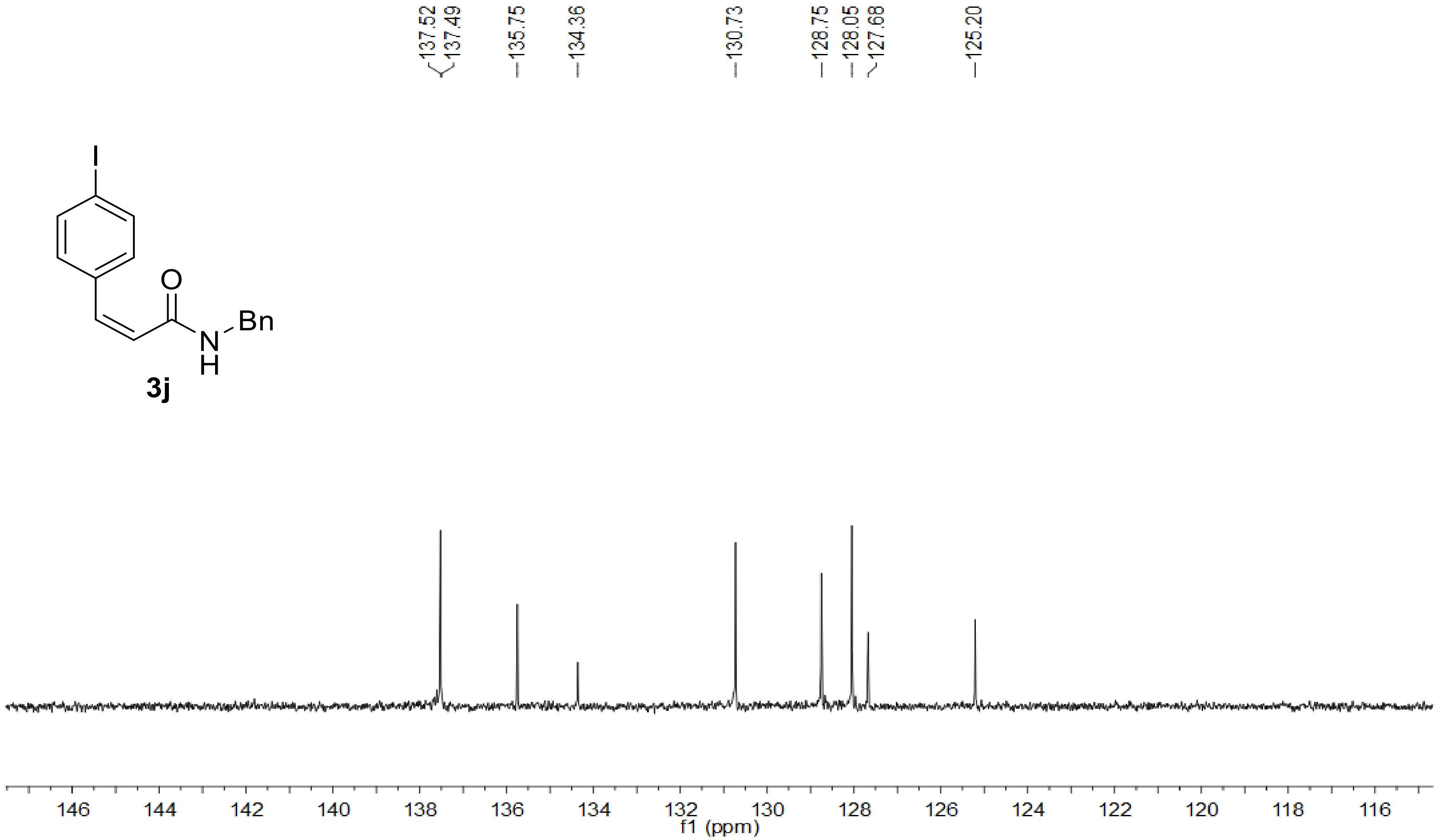

Supplementary Figure 40. Local magnification ^{13}C NMR spectrum of (*Z*)-*N*-benzyl-3-(4-iodophenyl)acrylamide **(3j)** in $CDCl_3$ (100 MHz) at 23℃.

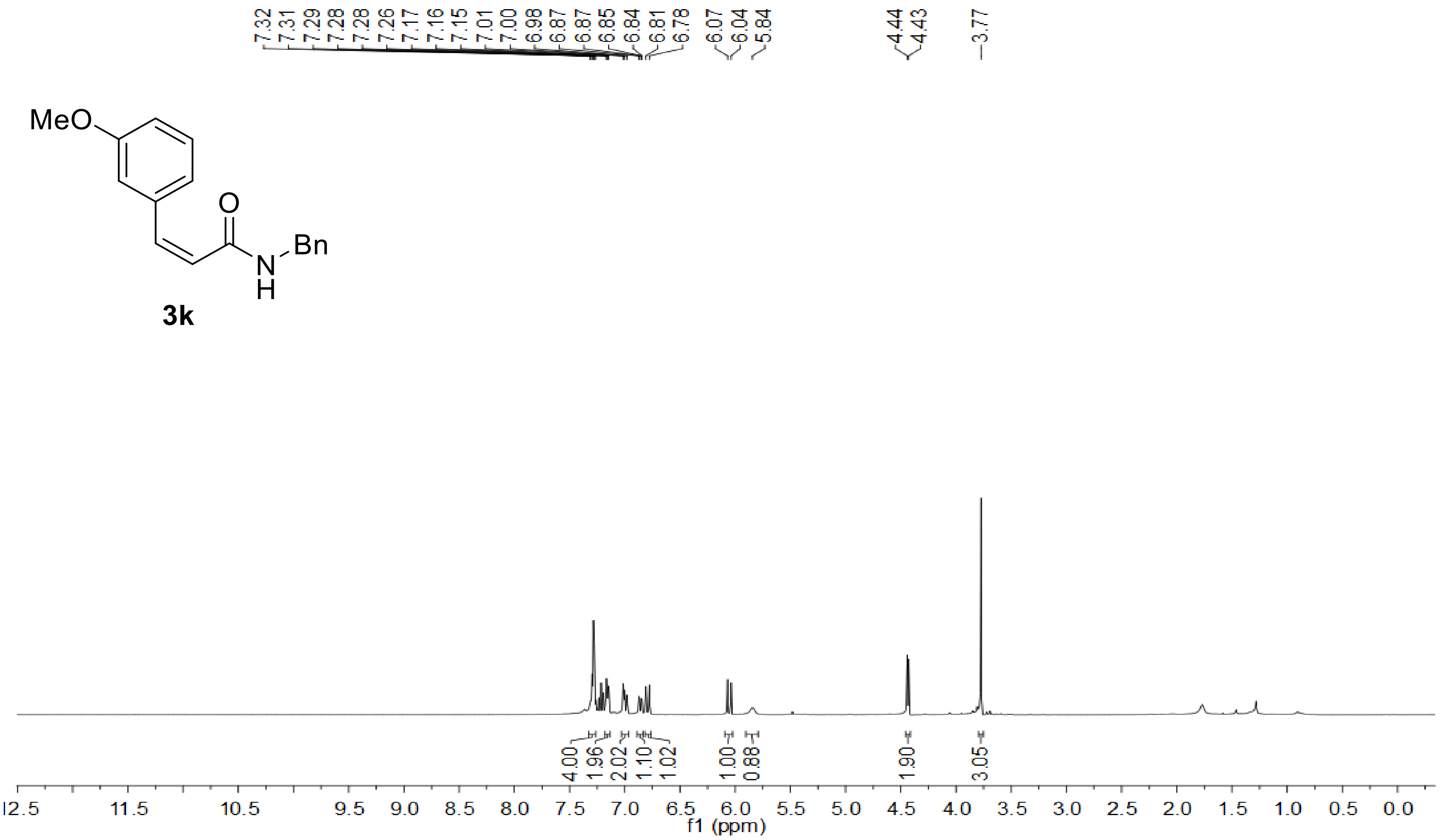

Supplementary Figure 41. ^{1}H NMR spectrum of (*Z*)-*N*-benzyl-3-(3-methoxyphenyl)acrylamide **(3k)** in $CDCl_3$ (400 MHz) at 23℃.

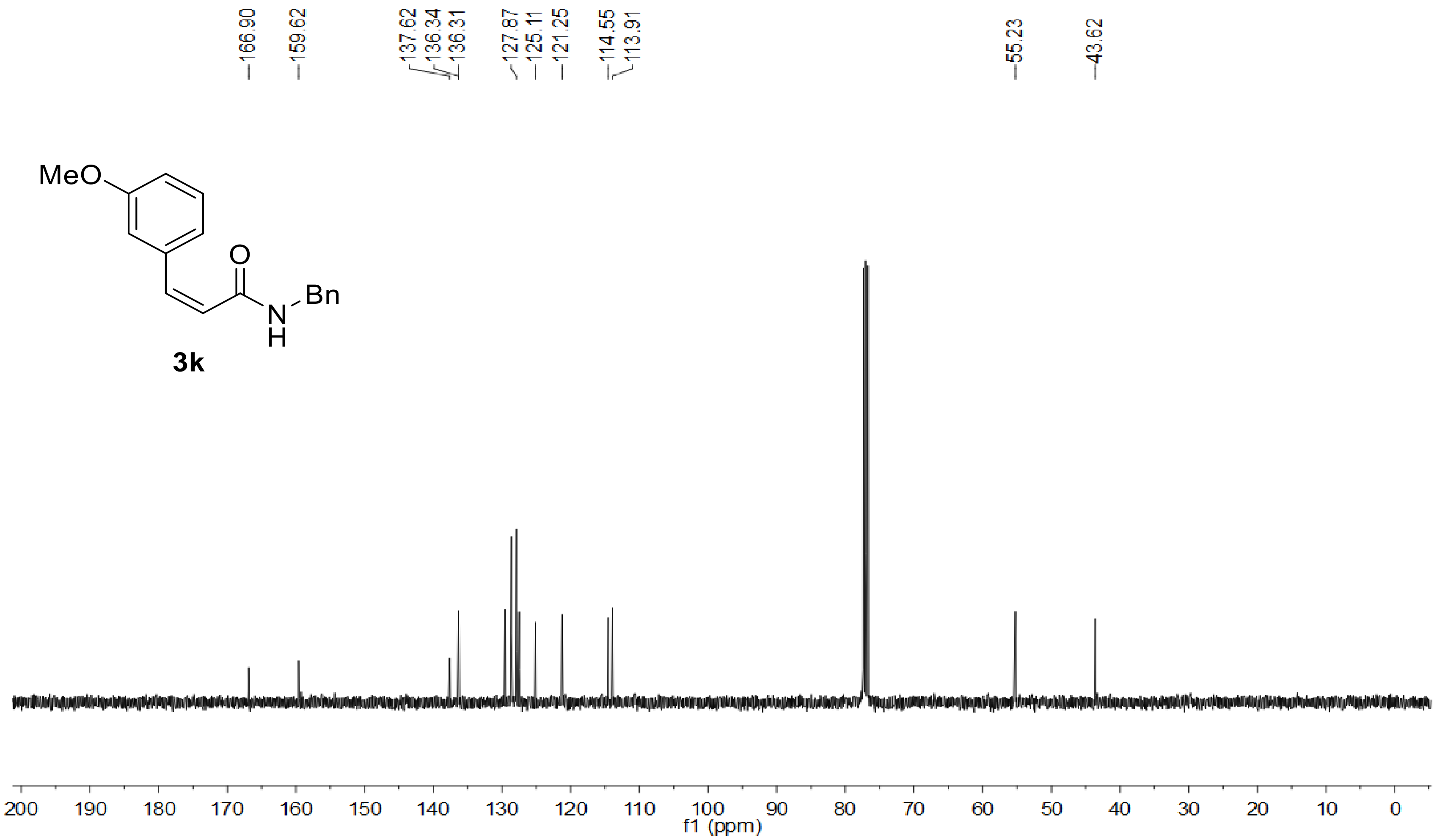

Supplementary Figure 42. ^{13}C NMR spectrum of (*Z*)-*N*-benzyl-3-(3-methoxyphenyl)acrylamide **(3k)** in $CDCl_3$ (100 MHz) at 23℃.

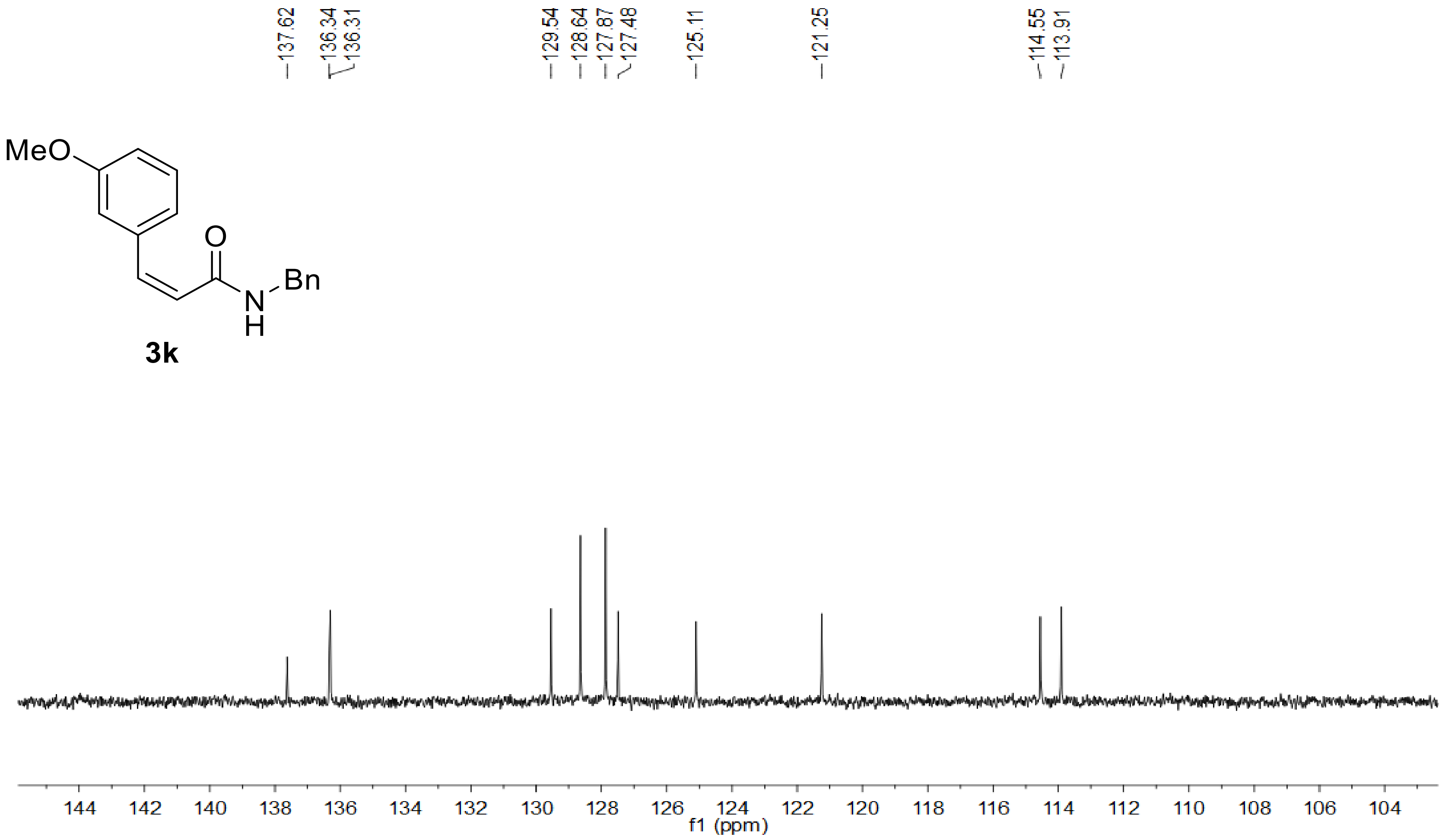

Supplementary Figure 43. Local magnification ^{13}C NMR spectrum of (*Z*)-*N*-benzyl-3-(3-methoxyphenyl)acrylamide **(3k)** in $CDCl_3$ (100 MHz) at 23℃.

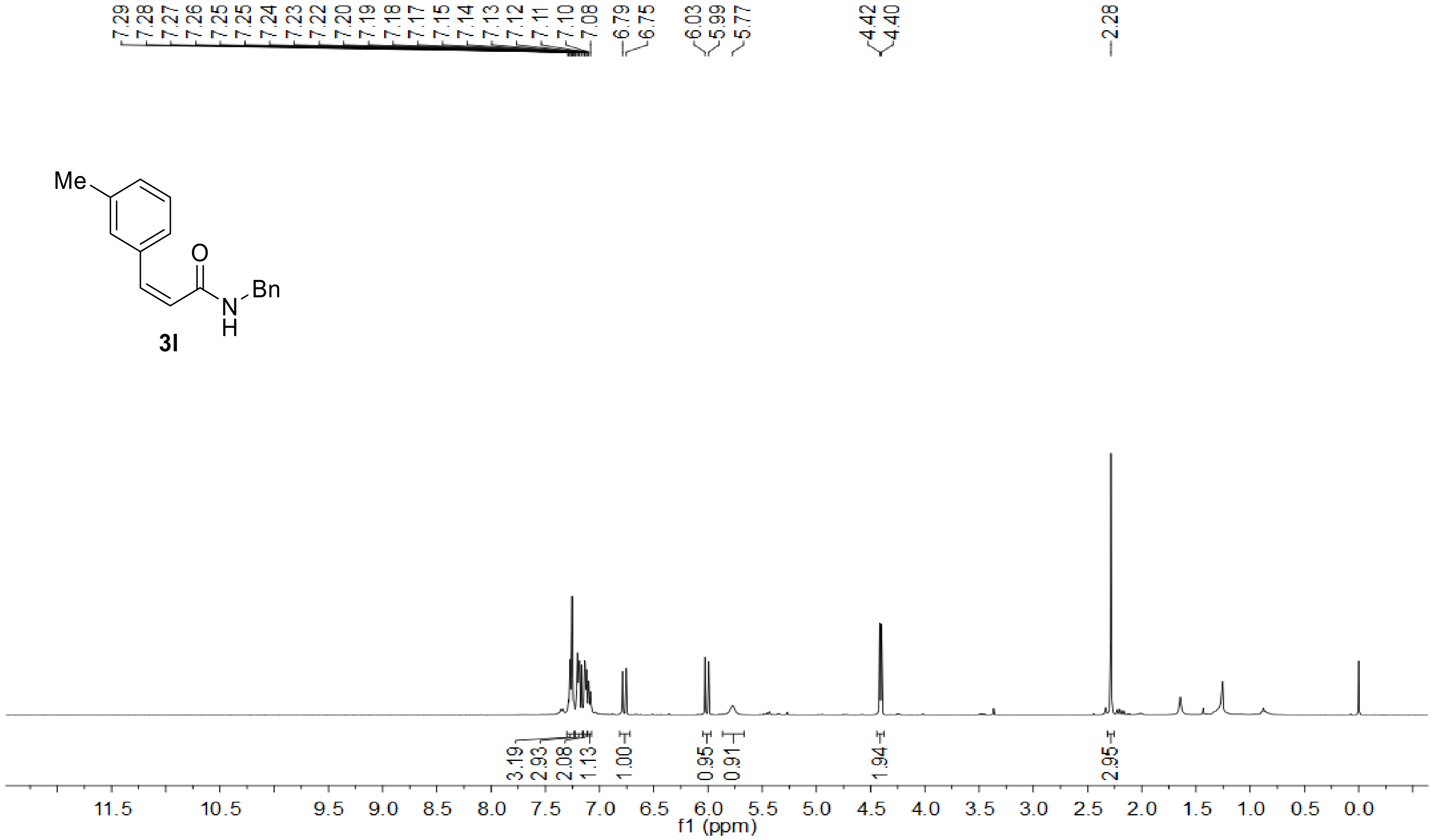

Supplementary Figure 44. ^{1}H NMR spectrum of (*Z*)-*N*-benzyl-3-(*m*-tolyl)acrylamide **(3l)** in $CDCl_3$ (400 MHz) at 23℃.

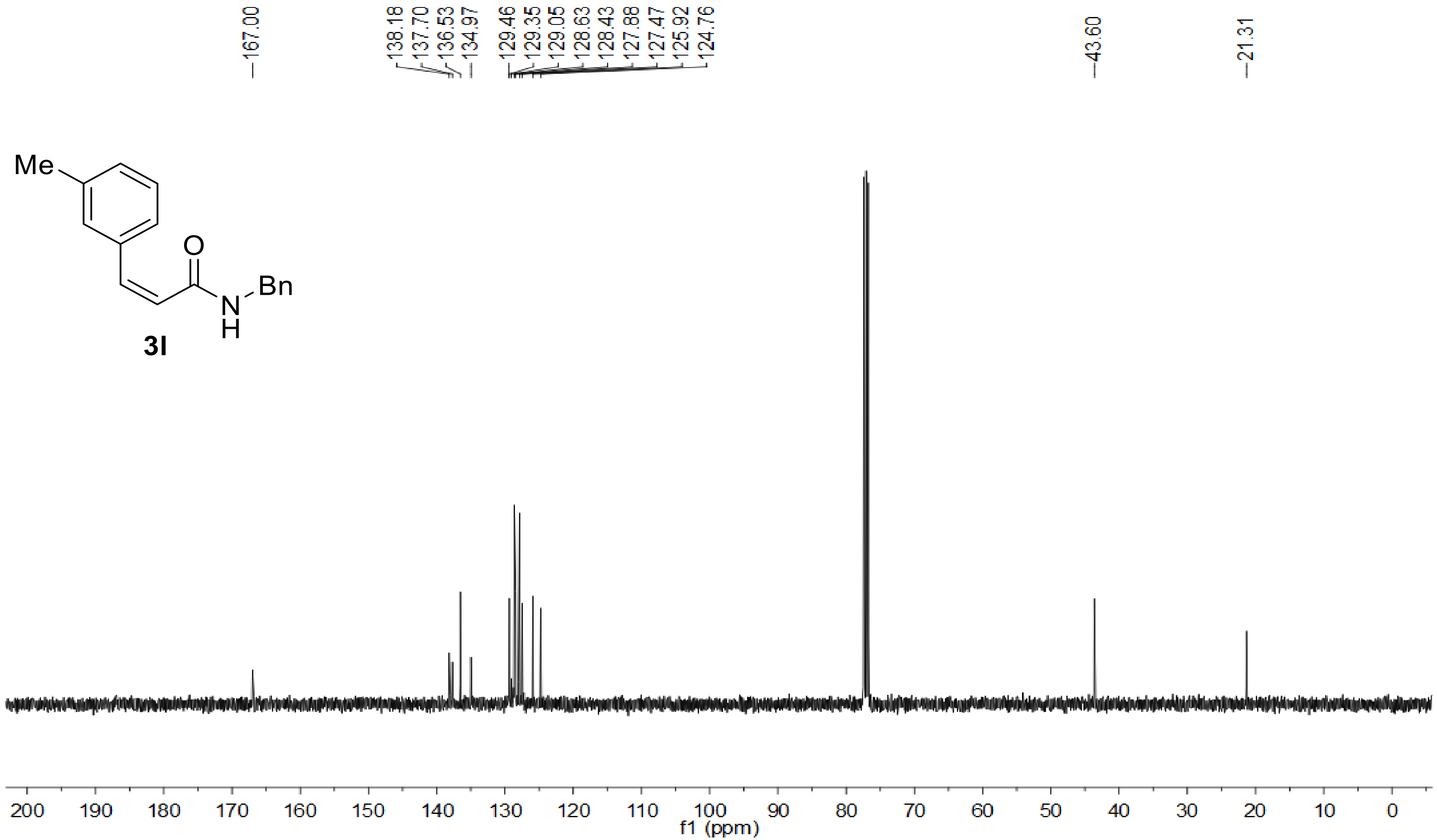

Supplementary Figure 45. ^{13}C NMR spectrum of (*Z*)-*N*-benzyl-3-(*m*-tolyl)acrylamide **(3l)** in $CDCl_3$ (100 MHz) at 23°C.

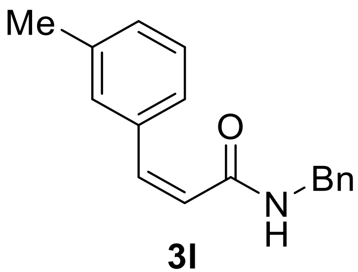

3l

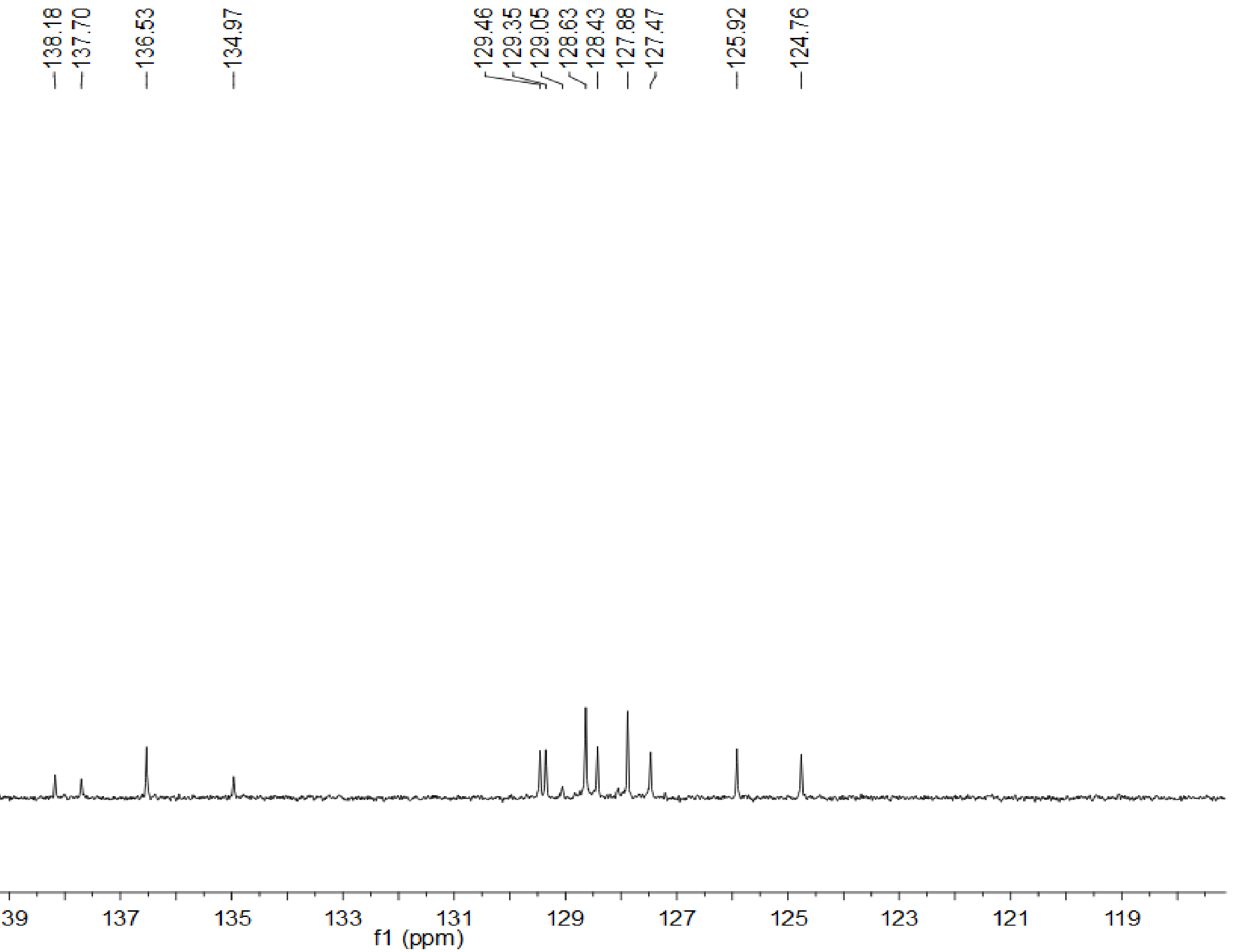

Supplementary Figure 46. Local magnification ^{13}C NMR spectrum of (*Z*)-*N*-benzyl-3-(*m*-tolyl)acrylamide **(3l)** in $CDCl_3$ (100 MHz) at 23℃.

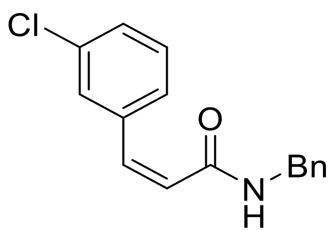

3m

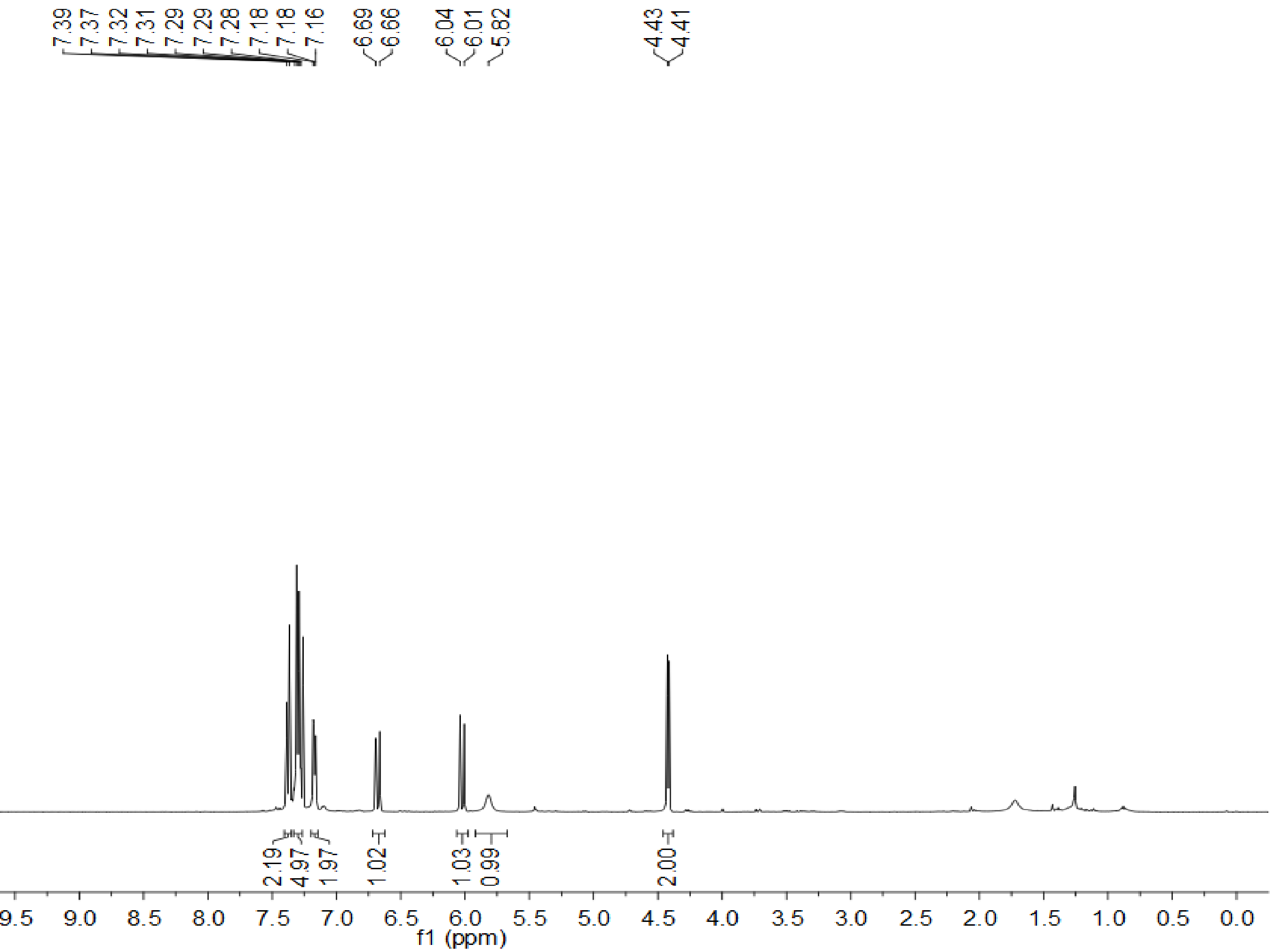

Supplementary Figure 47. ^{1}H NMR spectrum of (*Z*)-*N*-benzyl-3-(3-chlorophenyl)acrylamide **(3m)** in $CDCl_3$ (400 MHz) at 23℃.

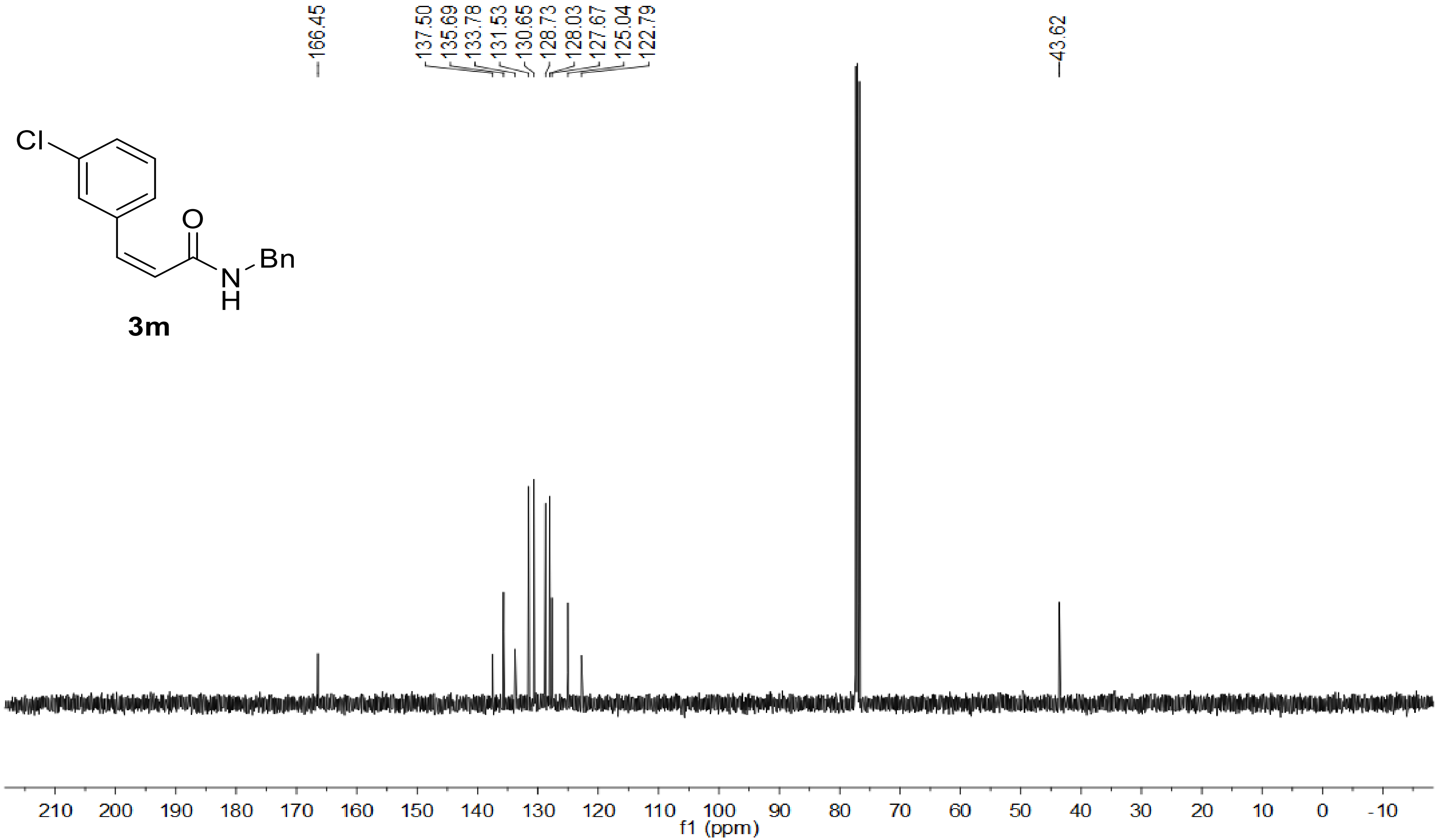

Supplementary Figure 48. ^{13}C NMR spectrum of (*Z*)-*N*-benzyl-3-(3-chlorophenyl)acrylamide **(3m)** in $CDCl_3$ (100 MHz) at 23°C.

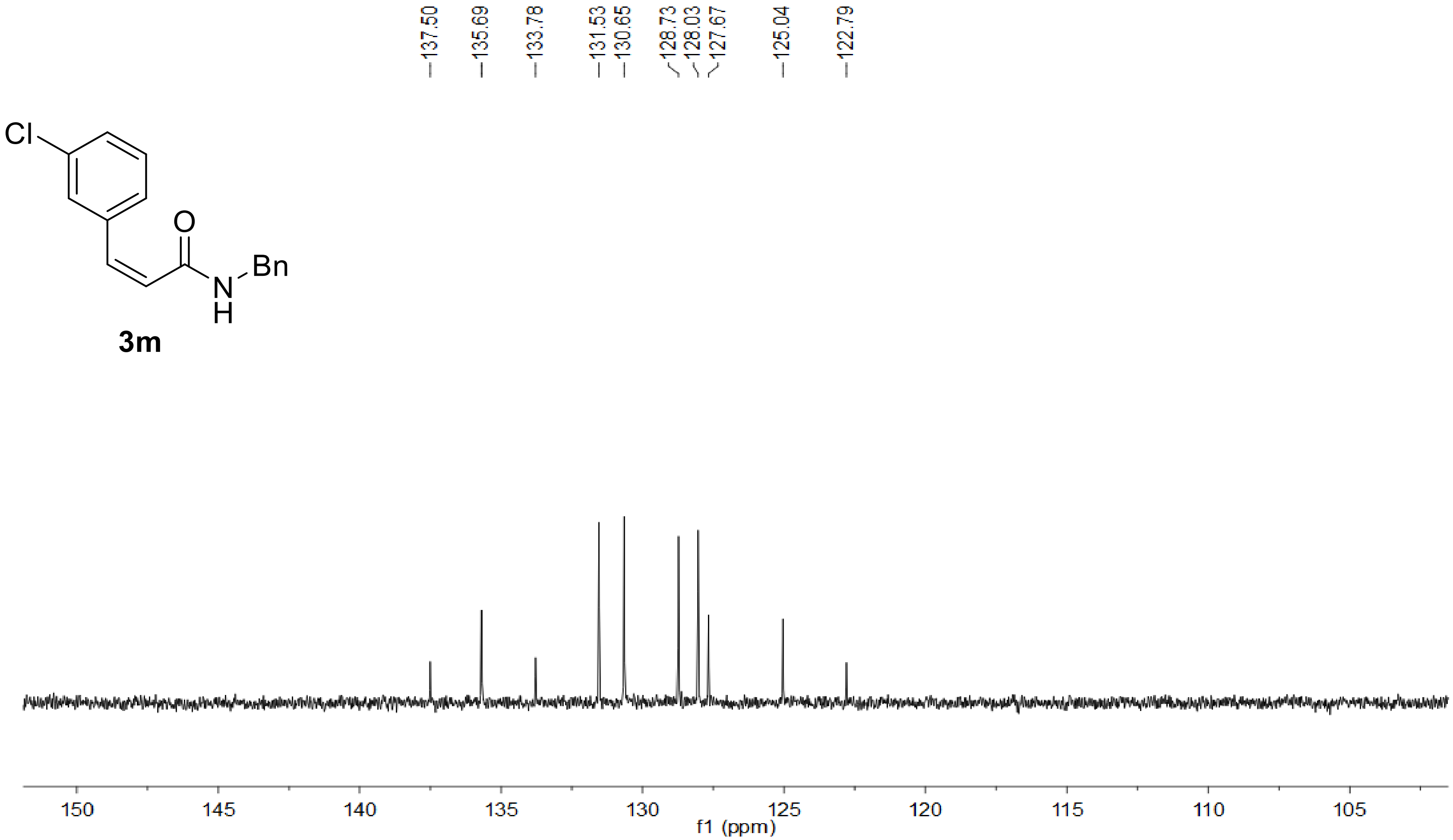

Supplementary Figure 49. Local magnification ^{13}C NMR spectrum of (*Z*)-*N*-benzyl-3-(3-chlorophenyl)acrylamide **(3m)** in $CDCl_3$ (100 MHz) at 23℃.

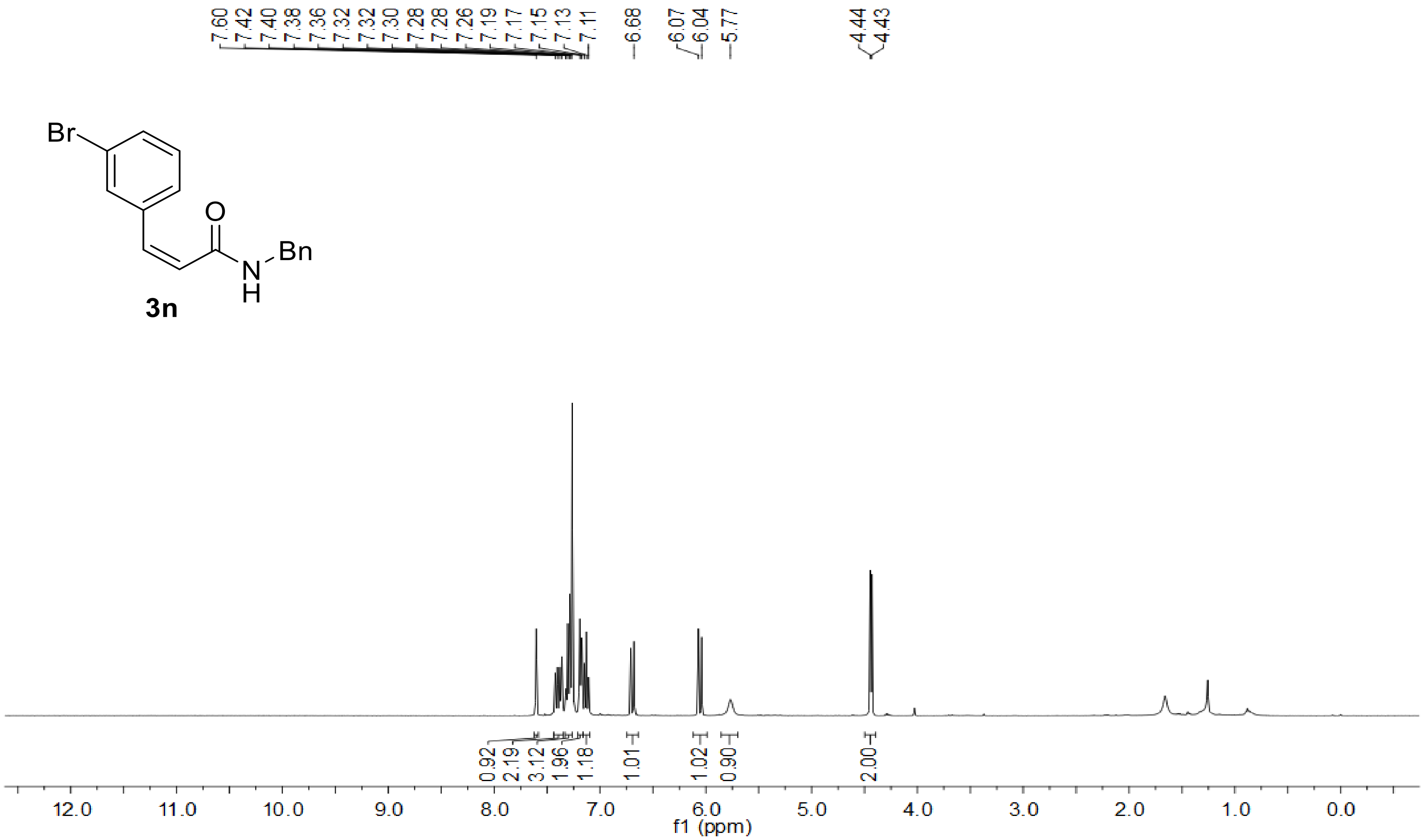

Supplementary Figure 50. ^{1}H NMR spectrum of (*Z*)-*N*-benzyl-3-(3-bromophenyl)acrylamide **(3n)** in $CDCl_3$ (400 MHz) at 23℃.

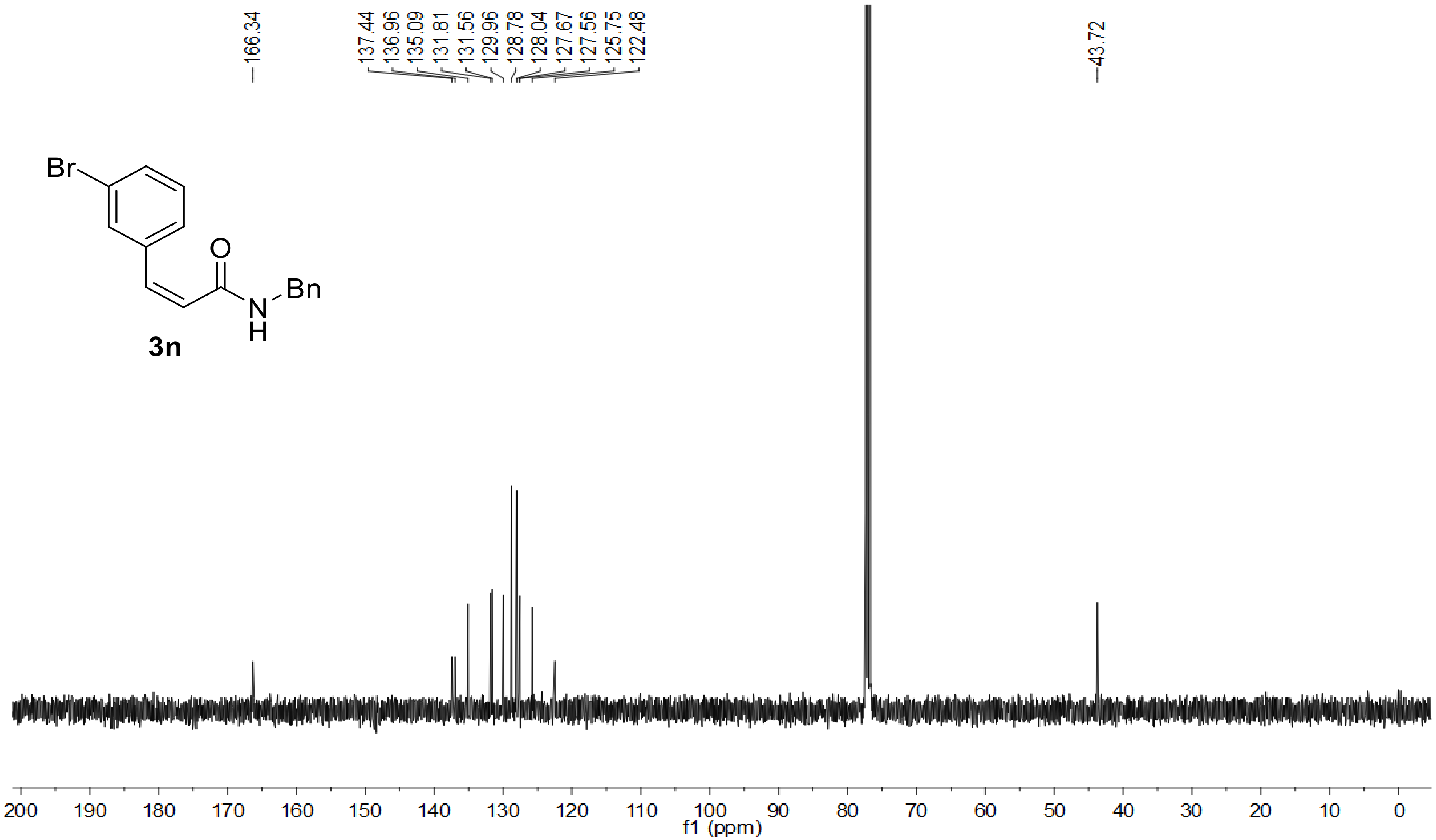

Supplementary Figure 51. ^{13}C NMR spectrum of (*Z*)-*N*-benzyl-3-(3-bromophenyl)acrylamide **(3n)** in $CDCl_3$ (100 MHz) at 23°C.

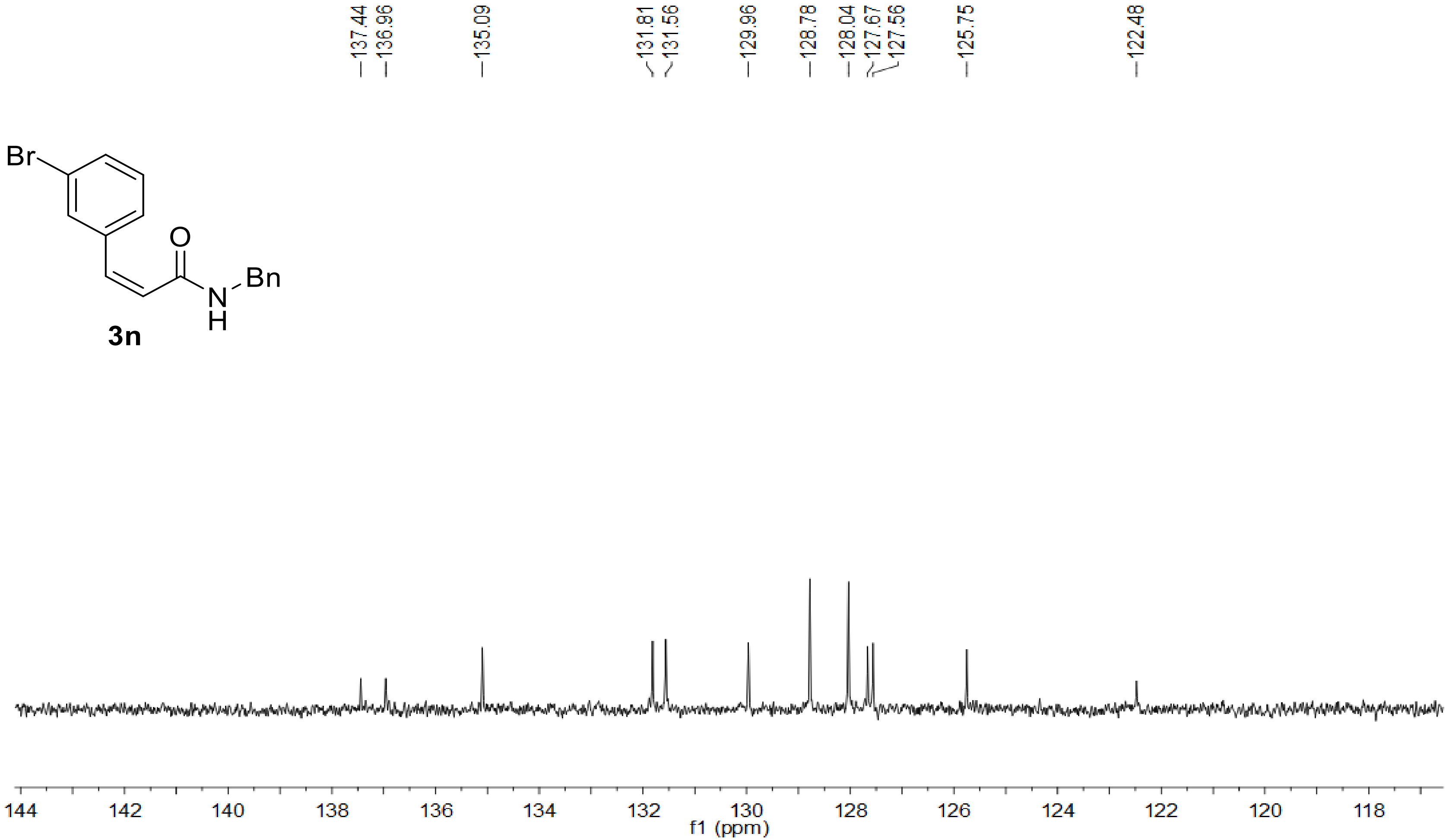

Supplementary Figure 52. Local magnification ^{13}C NMR spectrum of (*Z*)-*N*-benzyl-3-(3-bromophenyl)acrylamide **(3n)** in $CDCl_3$ (100 MHz) at 23℃.

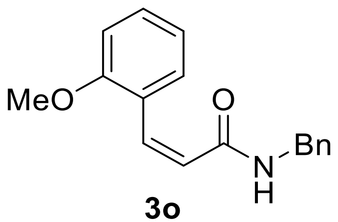

3o

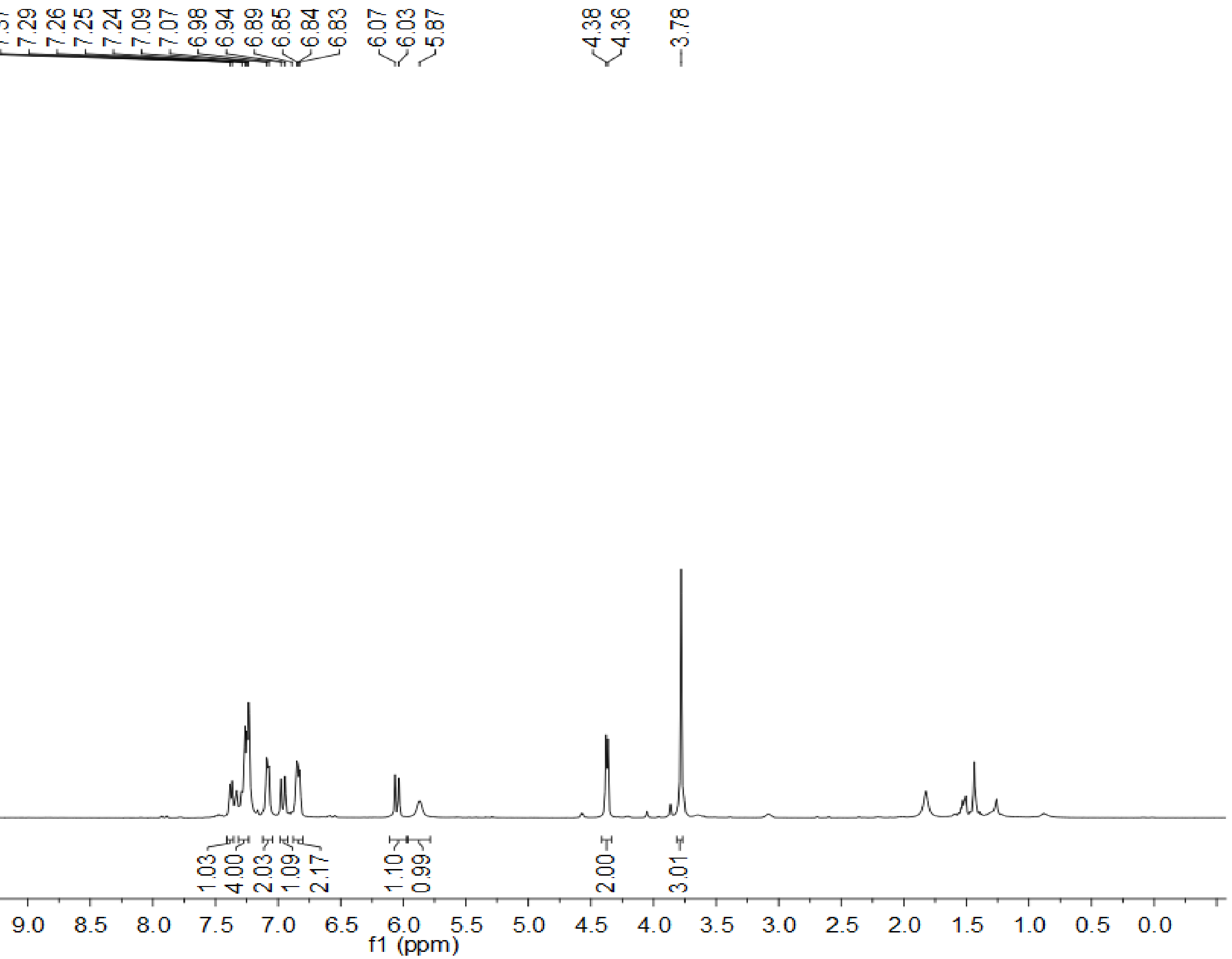

Supplementary Figure 53. ^{1}H NMR spectrum of (*Z*)-*N*-benzyl-3-(2-methoxyphenyl)acrylamide **(3o)** in $CDCl_3$ (400 MHz) at 23℃.

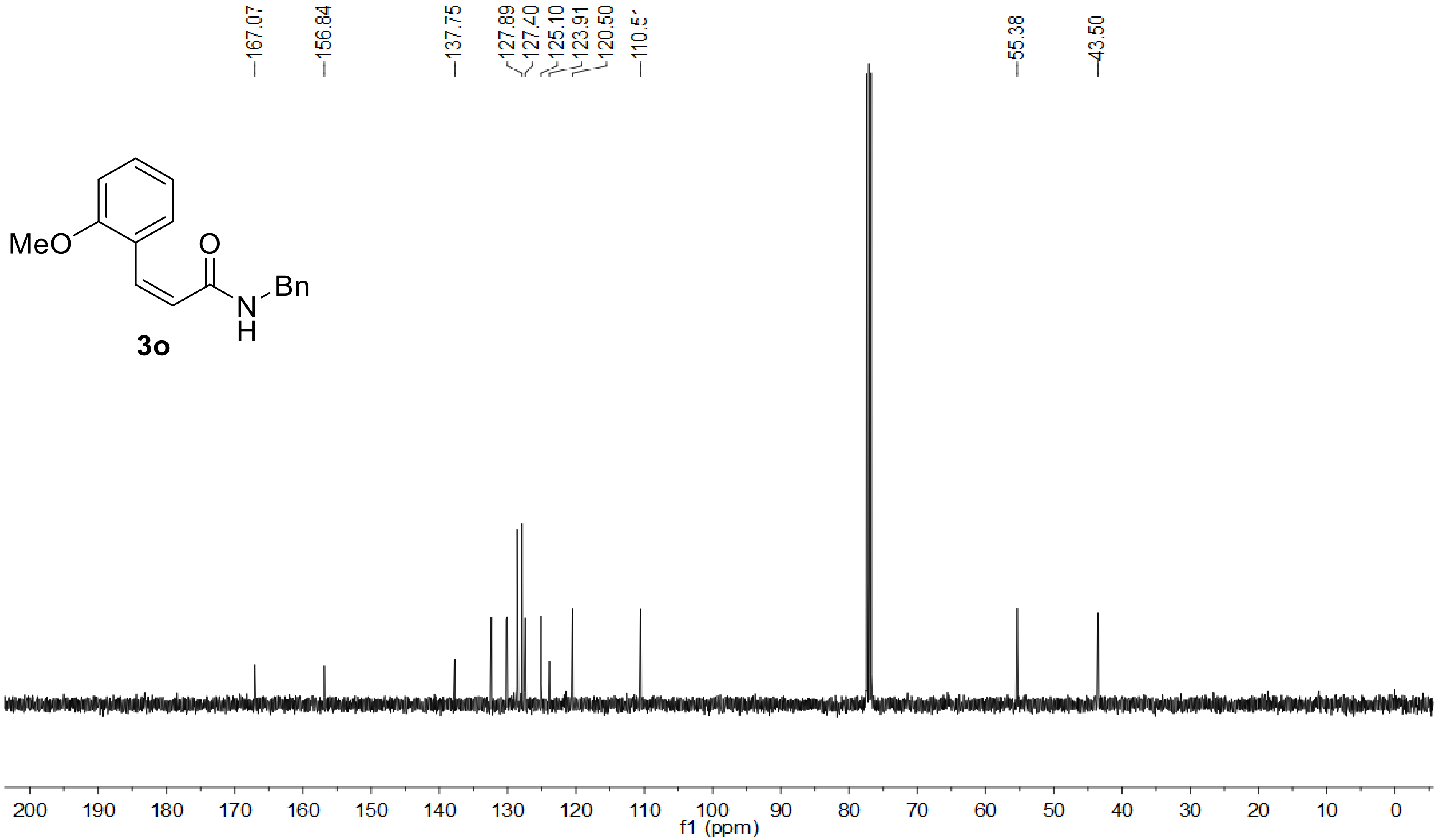

Supplementary Figure 54. ^{13}C NMR spectrum of (*Z*)-*N*-benzyl-3-(2-methoxyphenyl)acrylamide **(3o)** in $CDCl_3$ (100 MHz) at 23℃.

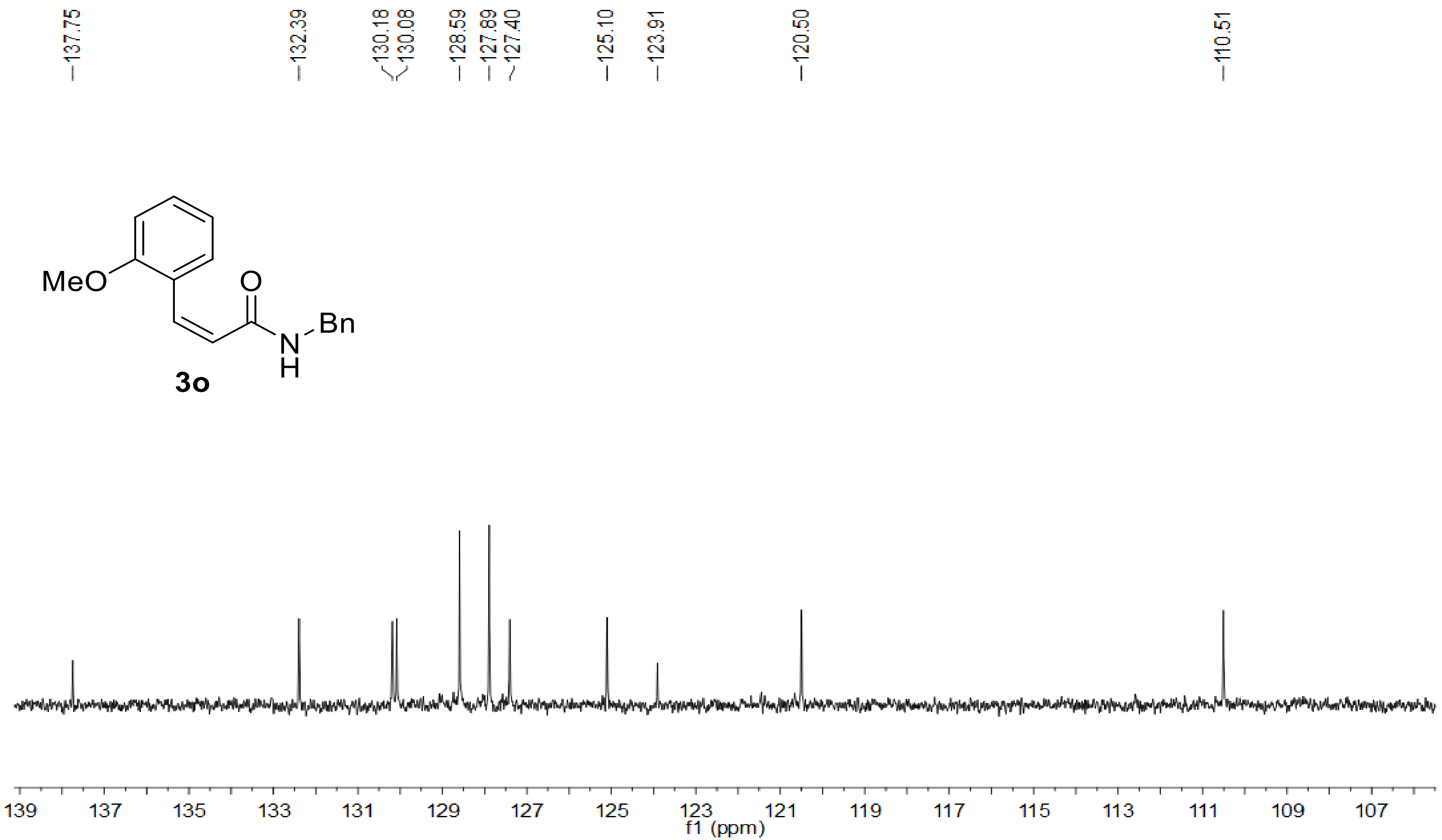

Supplementary Figure 55. Local magnification ^{13}C NMR spectrum of (*Z*)-*N*-benzyl-3-(2-methoxyphenyl)acrylamide **(3o)** in $CDCl_3$ (100 MHz) at 23℃.

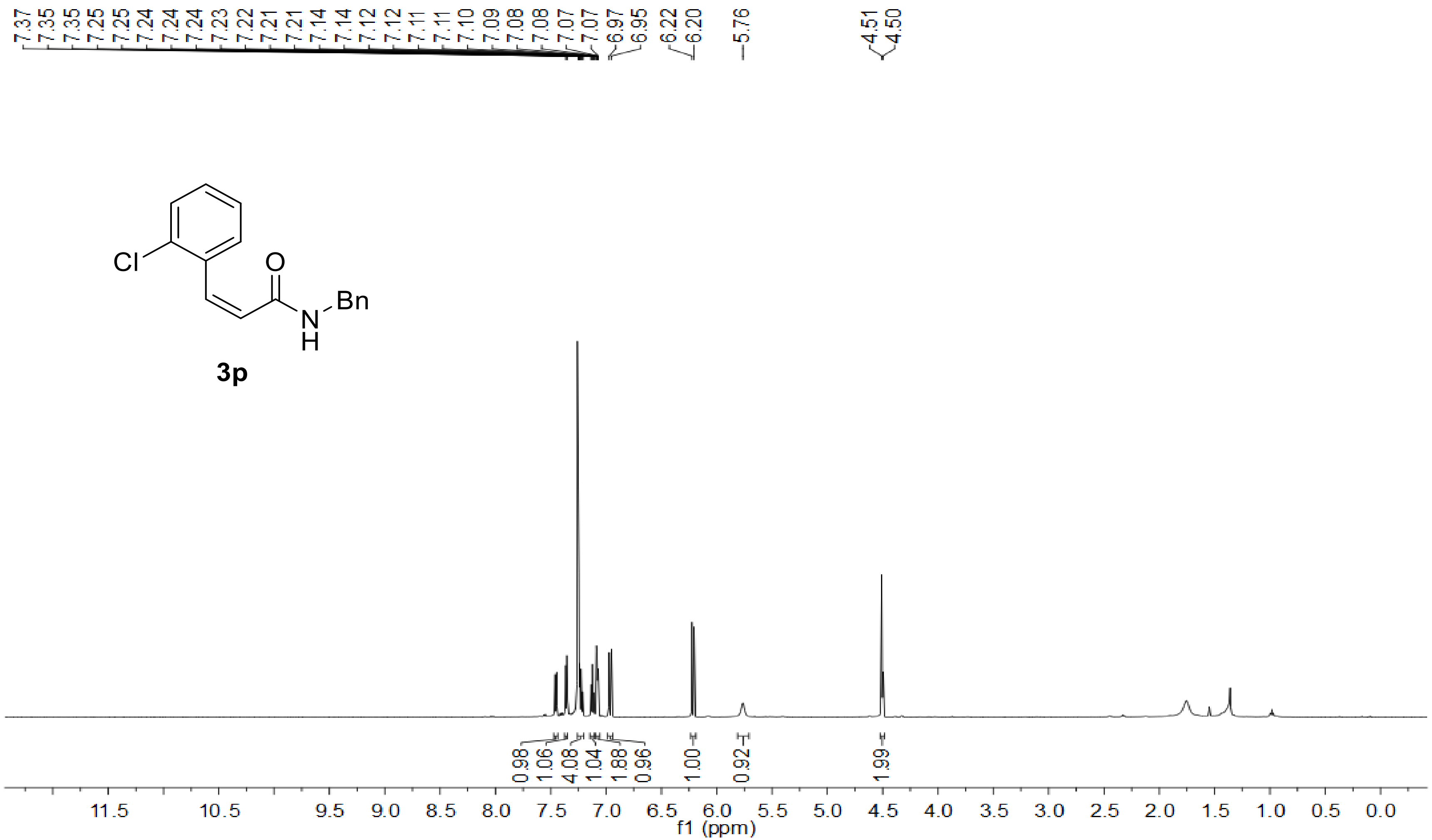

3p

Supplementary Figure 56. ^{1}H NMR spectrum of (*Z*)-*N*-benzyl-3-(2-chlorophenyl)acrylamide **(3p)** in $CDCl_3$ (600 MHz) at 23℃.

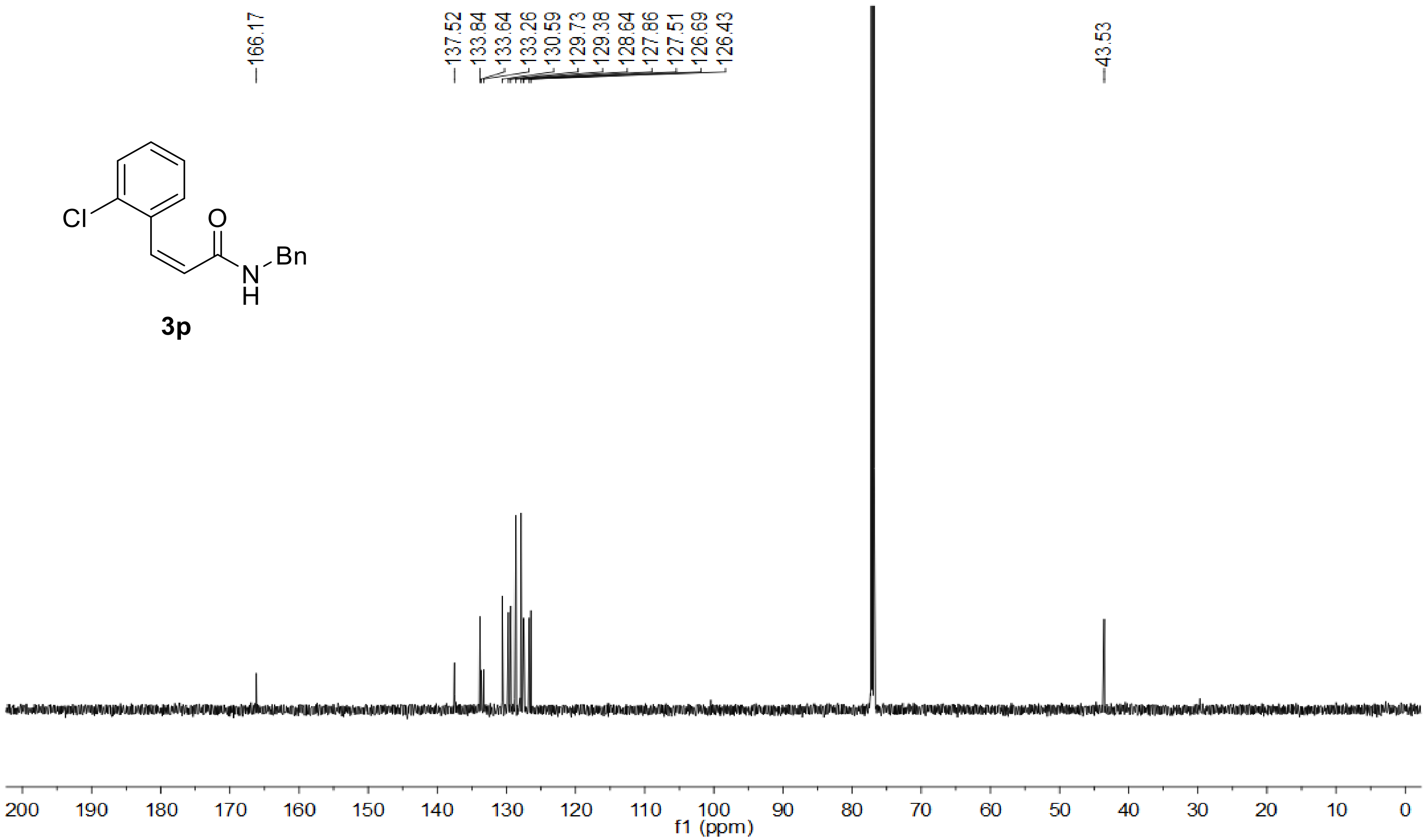

Supplementary Figure 57. ^{13}C NMR spectrum of (*Z*)-*N*-benzyl-3-(2-chlorophenyl)acrylamide **(3p)** in $CDCl_3$ (100 MHz) at 23°C.

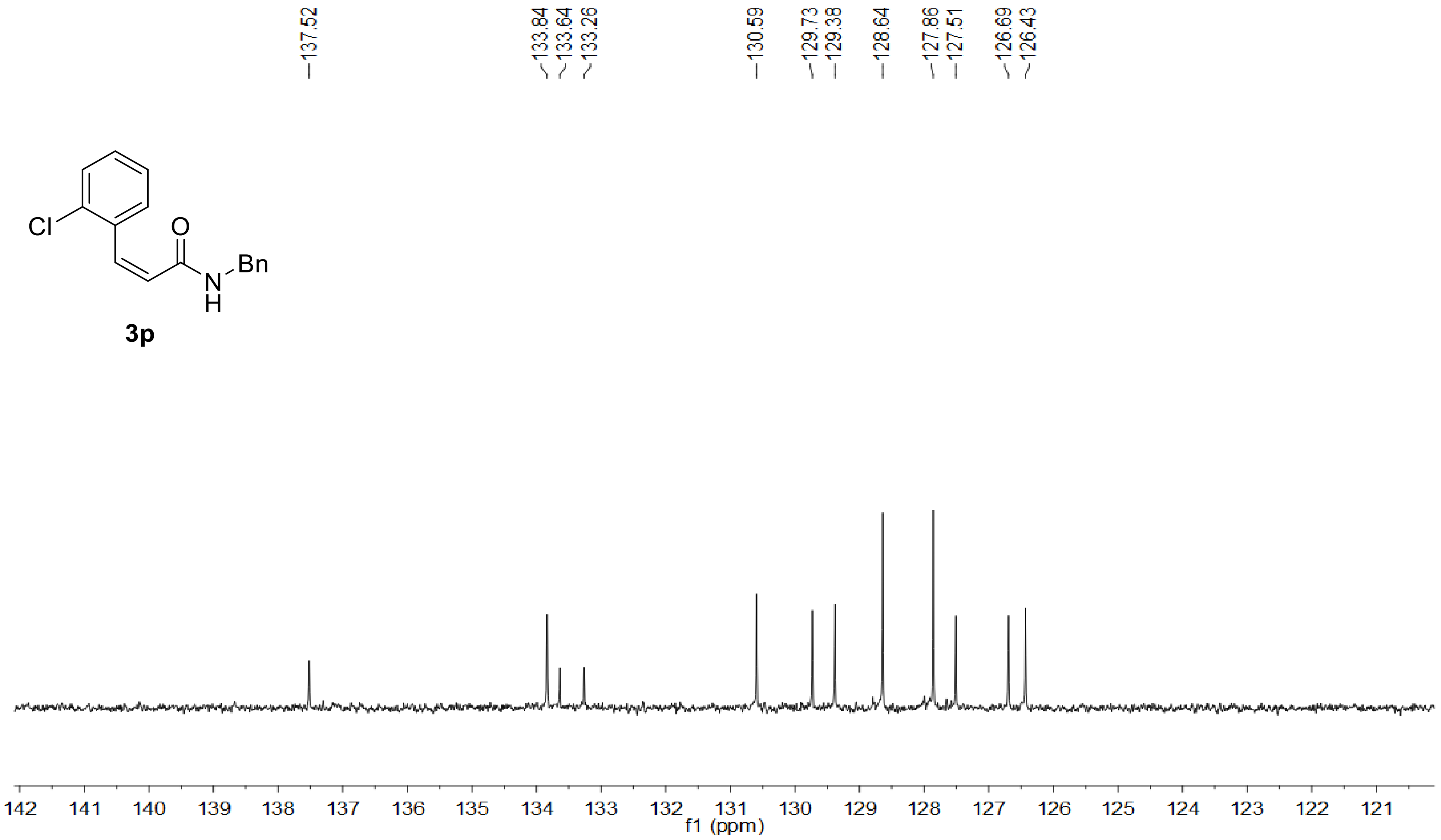

Supplementary Figure 58. Local magnification ^{13}C NMR spectrum of (*Z*)-*N*-benzyl-3-(2-chlorophenyl)acrylamide **(3p)** in $CDCl_3$ (100 MHz) at 23℃.

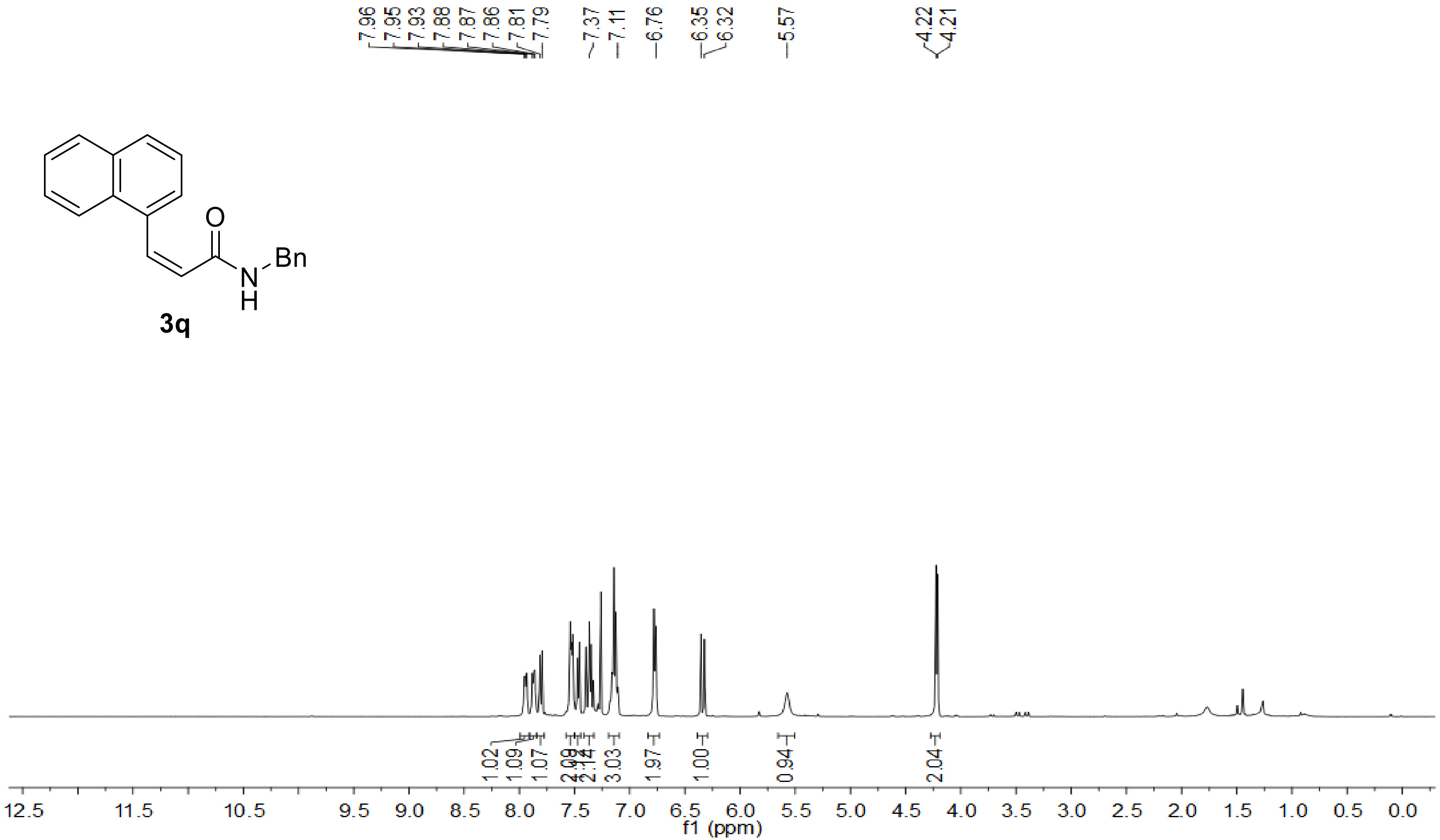

Supplementary Figure 59. ^{1}H NMR spectrum of (*Z*)-*N*-benzyl-3-(naphthalen-1-yl)acrylamide **(3q)** in $CDCl_3$ (400 MHz) at 23°C.

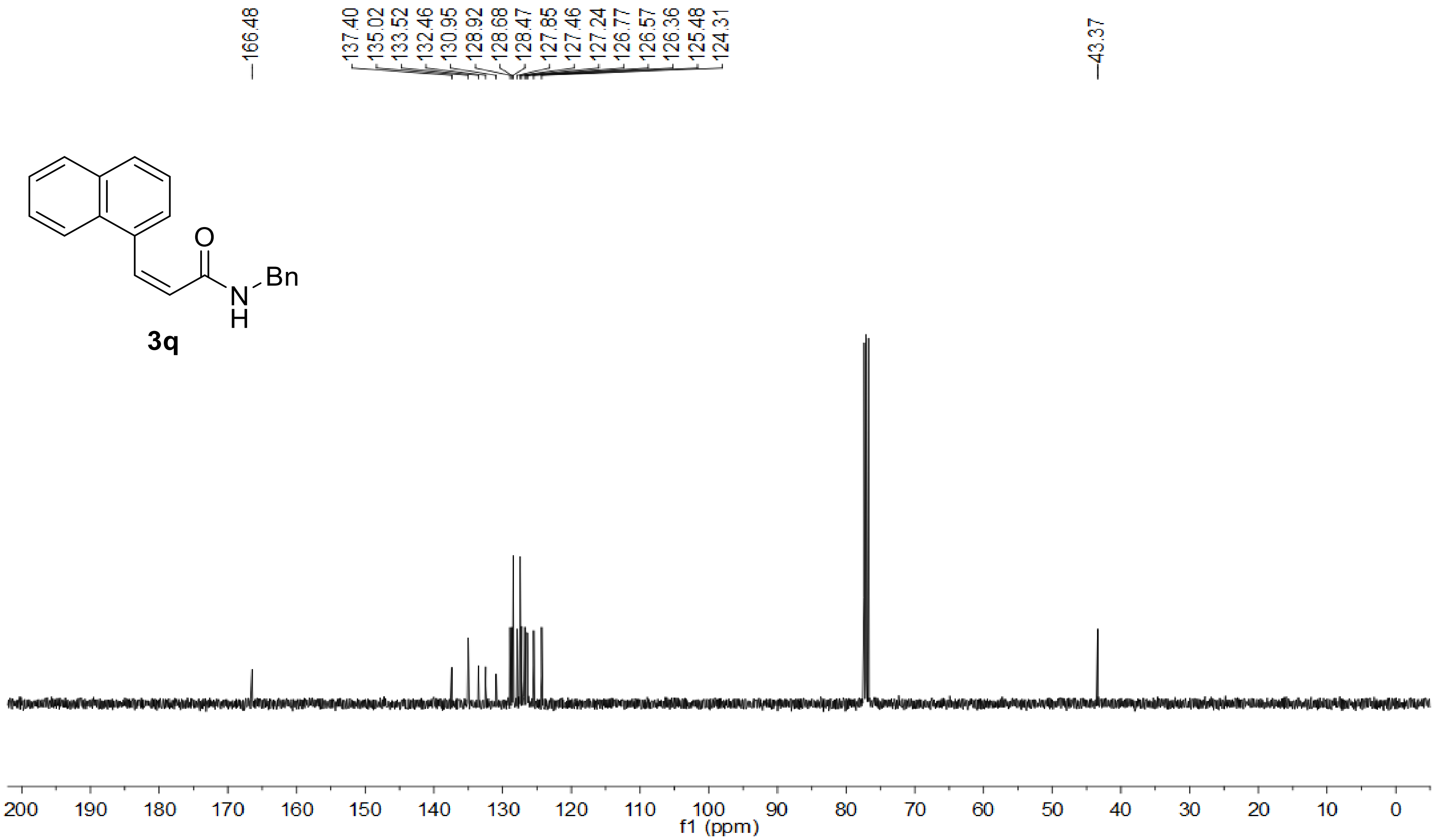

Supplementary Figure 60. ^{13}C NMR spectrum of (*Z*)-*N*-benzyl-3-(naphthalen-1-yl)acrylamide **(3q)** in $CDCl_3$ (100 MHz) at 23°C.

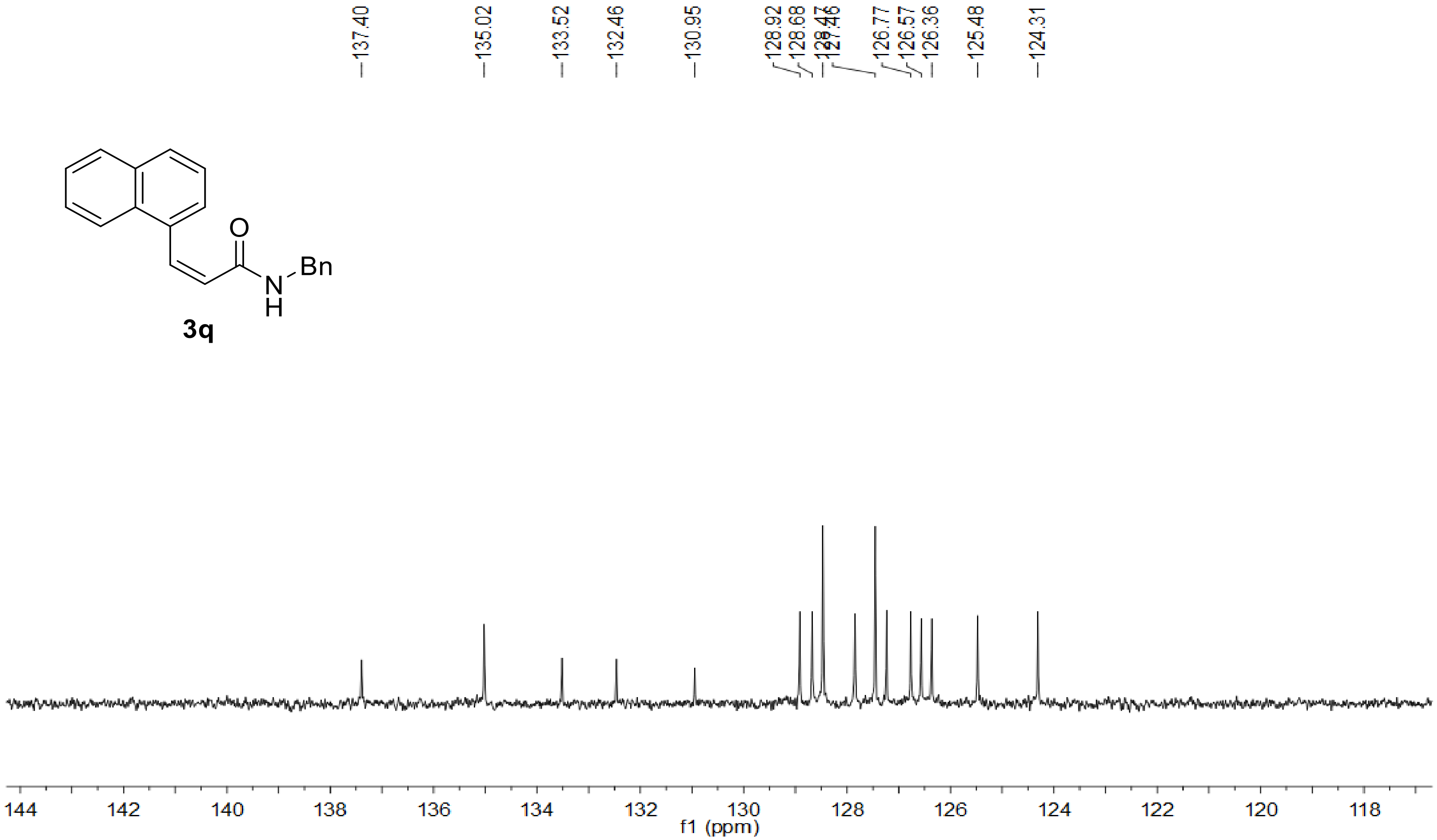

Supplementary Figure 61. Local magnification ^{13}C NMR spectrum of (*Z*)-*N*-benzyl-3-(naphthalen-1-yl)acrylamide **(3q)** in $CDCl_3$ (100 MHz) at 23℃.

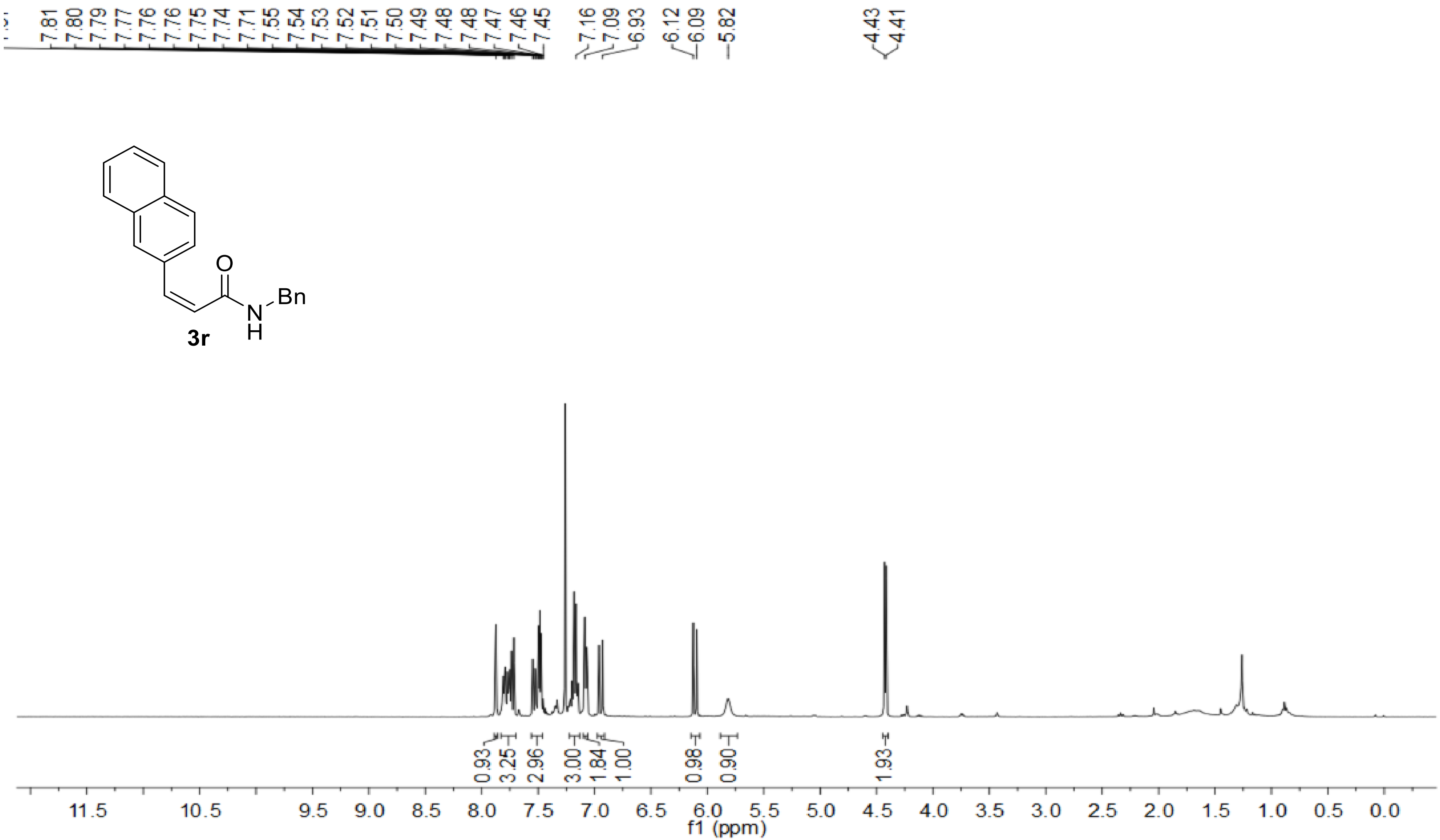

Supplementary Figure 62. ^{1}H NMR spectrum of (*Z*)-*N*-benzyl-3-(naphthalen-2-yl)acrylamide **(3r)** in $CDCl_3$ (400 MHz) at 23℃.

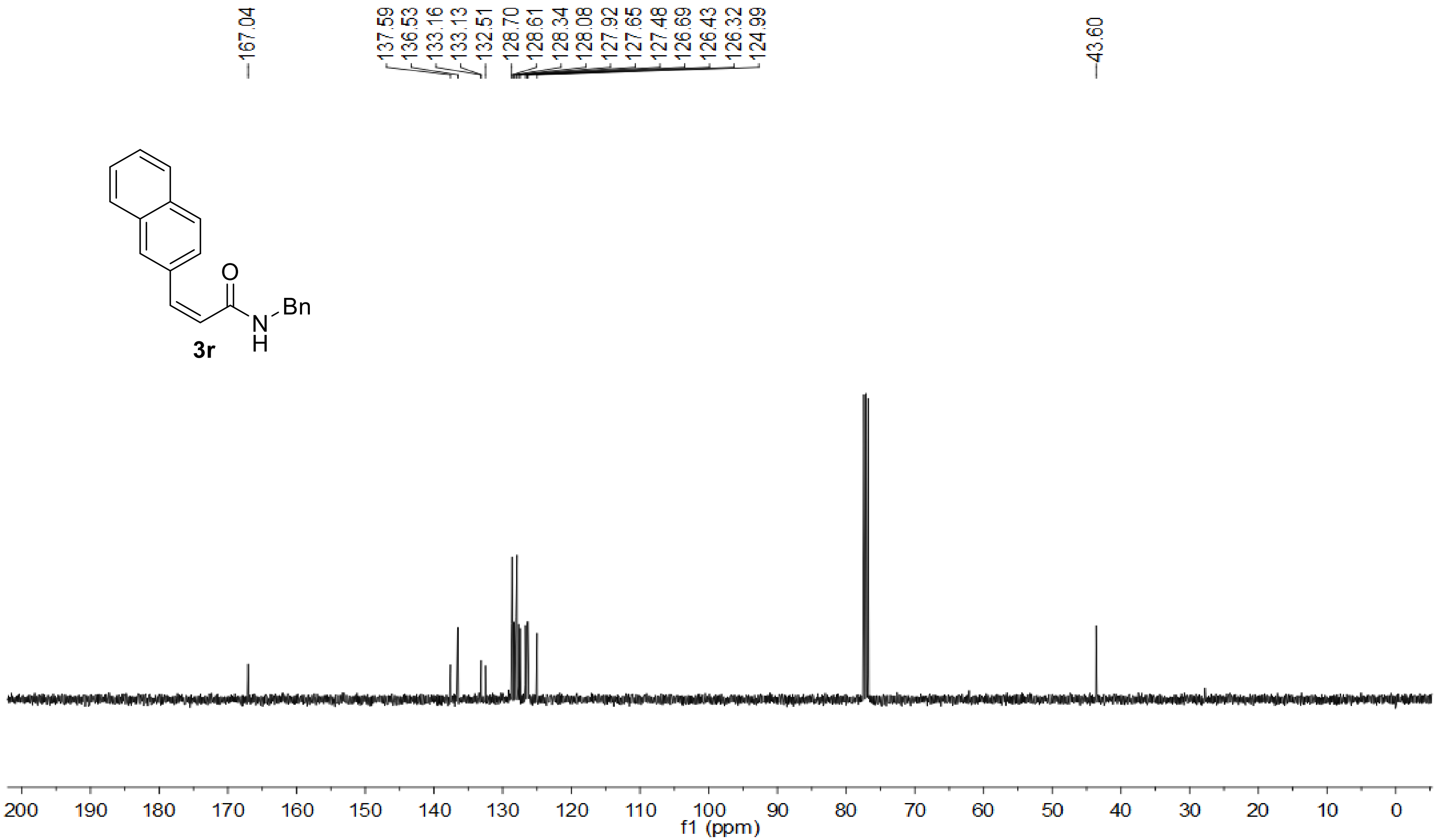

Supplementary Figure 63. ^{13}C NMR spectrum of (*Z*)-*N*-benzyl-3-(naphthalen-2-yl)acrylamide **(3r)** in $CDCl_3$ (100 MHz) at 23℃.

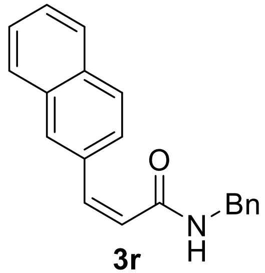

3r

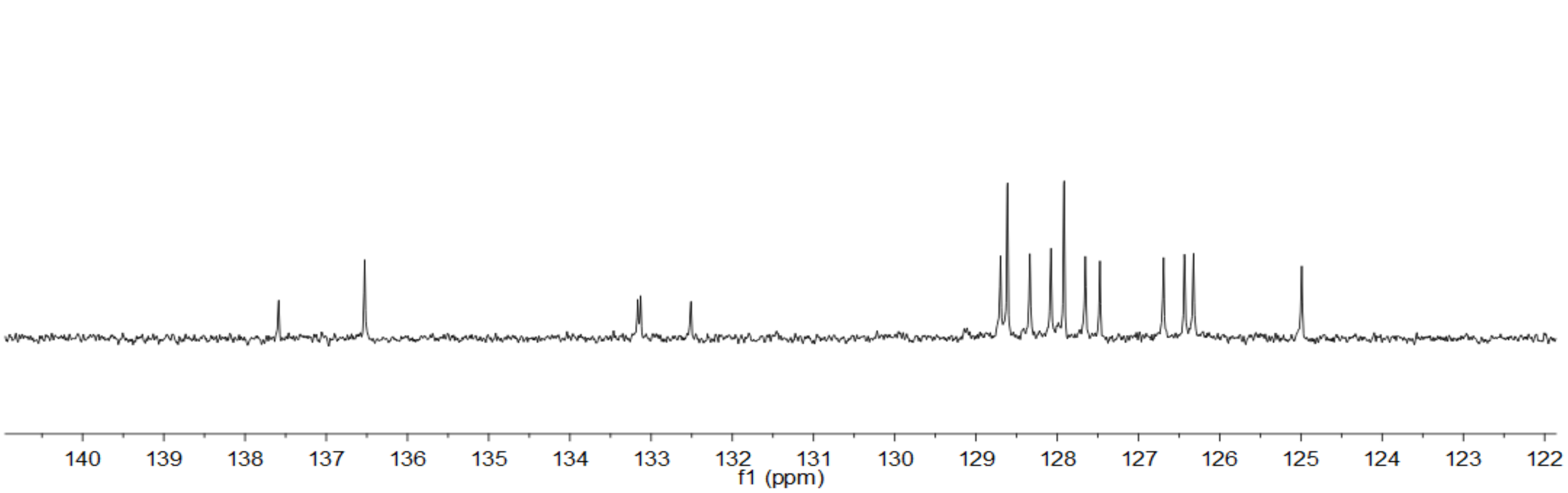

Supplementary Figure 64. Local magnification ^{13}C NMR spectrum of (*Z*)-*N*-benzyl-3-(naphthalen-2-yl)acrylamide **(3r)** in $CDCl_3$ (100 MHz) at 23℃.

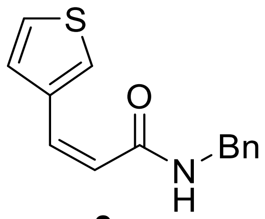

3s

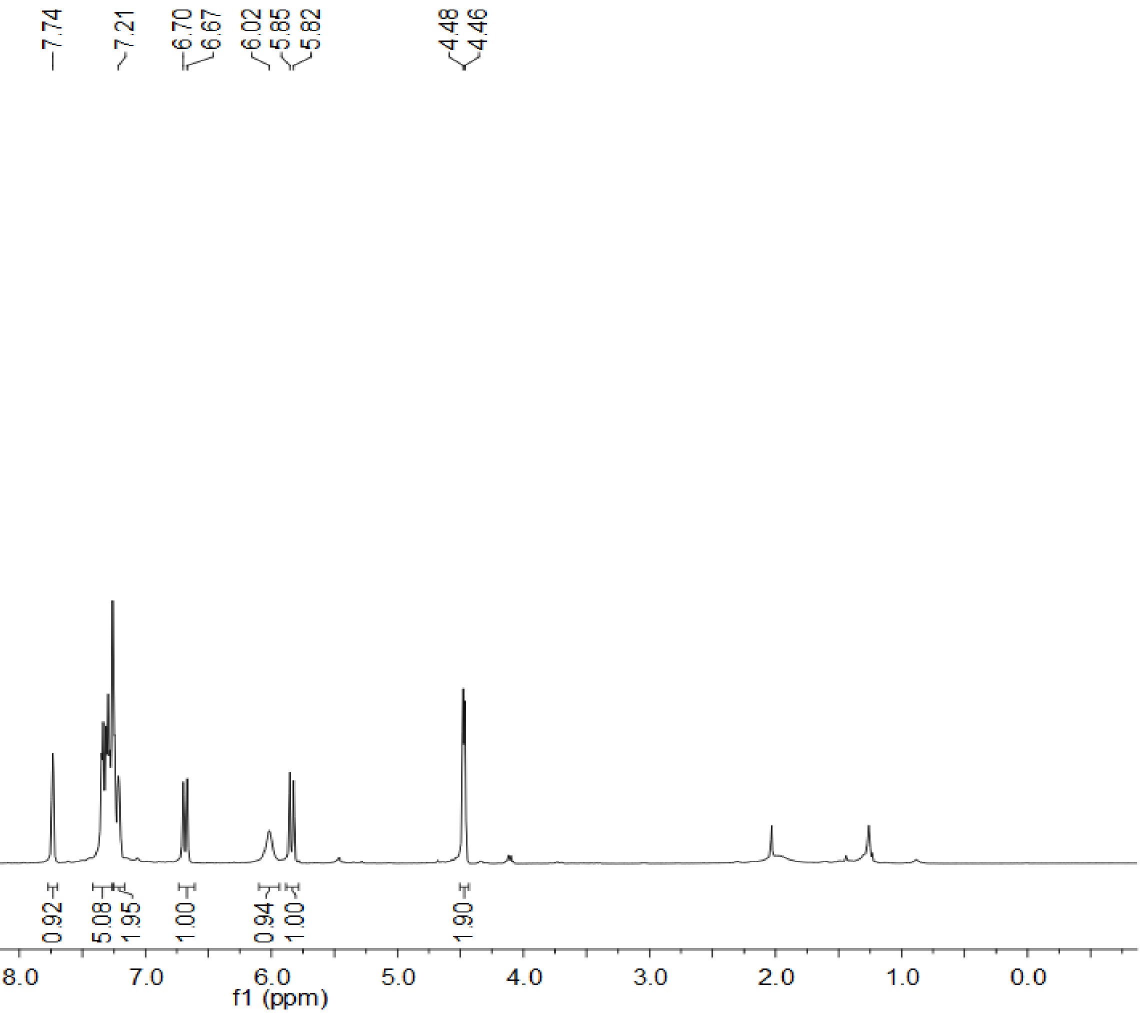

Supplementary Figure 65. ^{1}H NMR spectrum of (*Z*)-*N*-benzyl-3-(thiophen-3-yl)acrylamide **(3s)** in $CDCl_3$ (400 MHz) at 23℃.

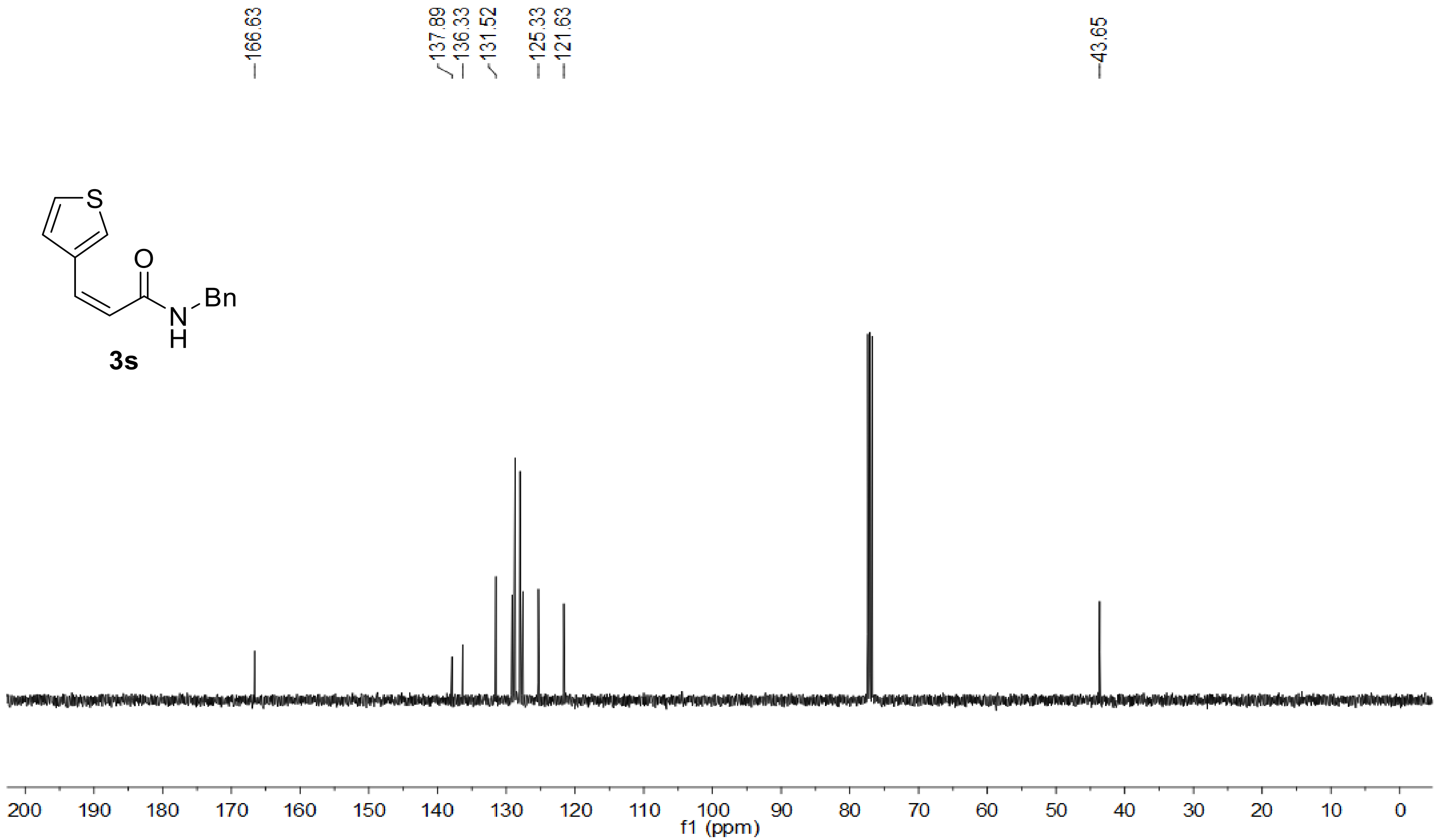

Supplementary Figure 66. ^{13}C NMR spectrum of (*Z*)-*N*-benzyl-3-(thiophen-3-yl)acrylamide **(3s)** in $CDCl_3$ (100 MHz) at 23℃.

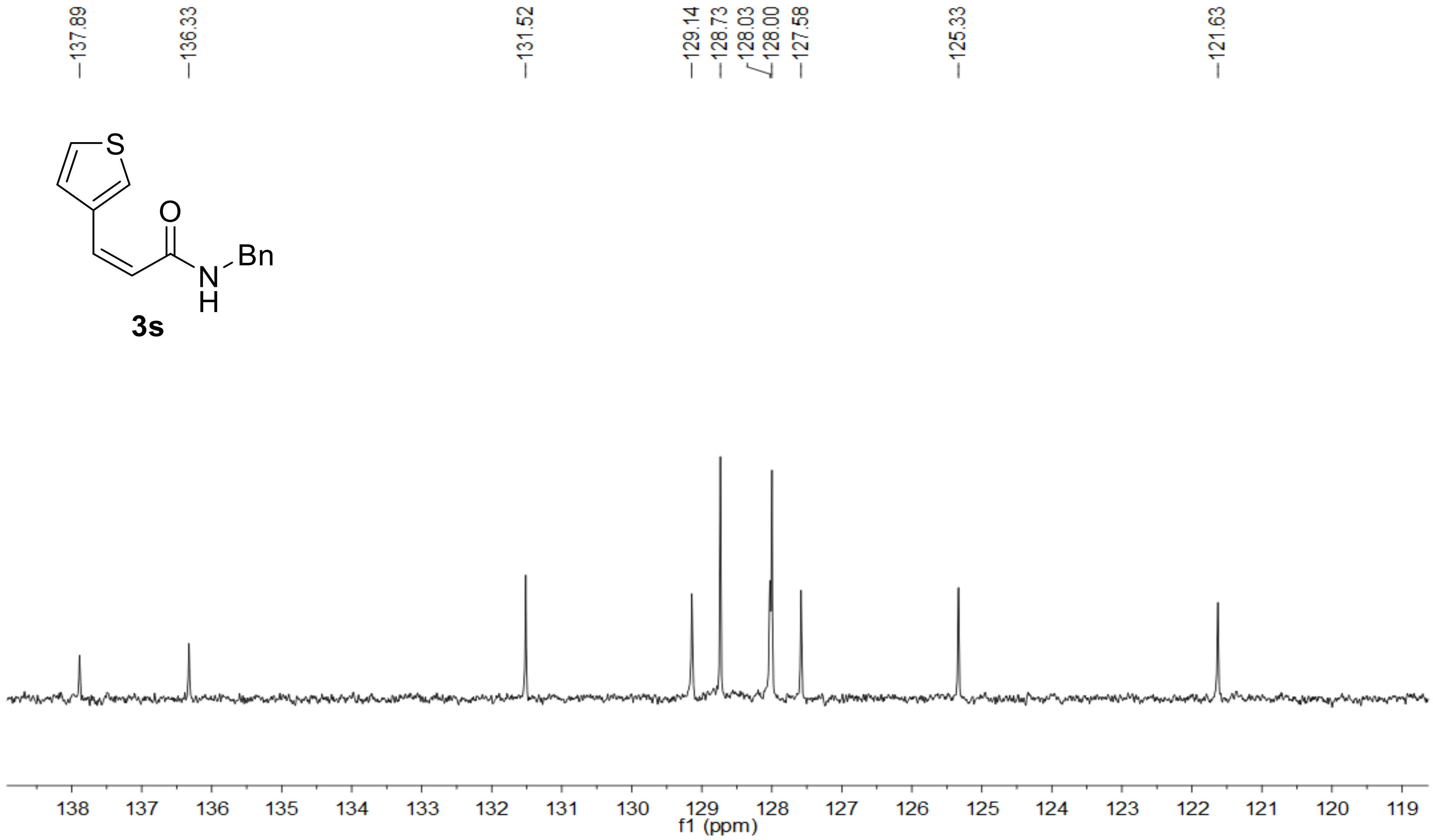

Supplementary Figure 67. Local magnification ^{13}C NMR spectrum of (*Z*)-*N*-benzyl-3-(thiophen-3-yl)acrylamide **(3s)** in $CDCl_3$ (100 MHz) at 23℃.

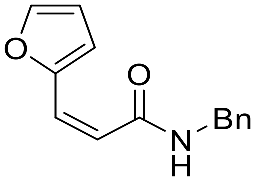

3t

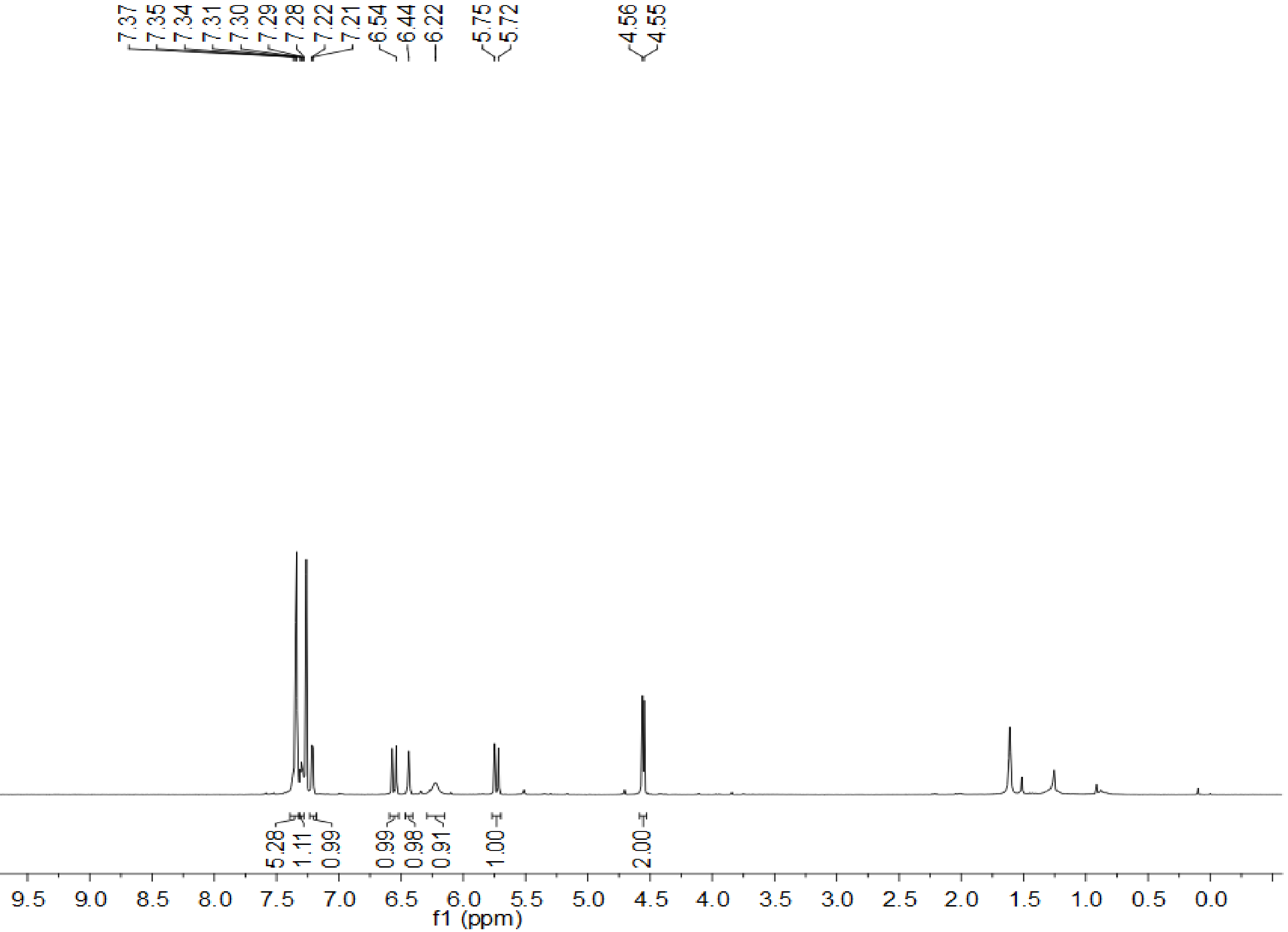

Supplementary Figure 68. ^{1}H NMR spectrum of (*Z*)-*N*-benzyl-3-(furan-2-yl)acrylamide **(3t)** in $CDCl_3$ (400 MHz) at 23℃.

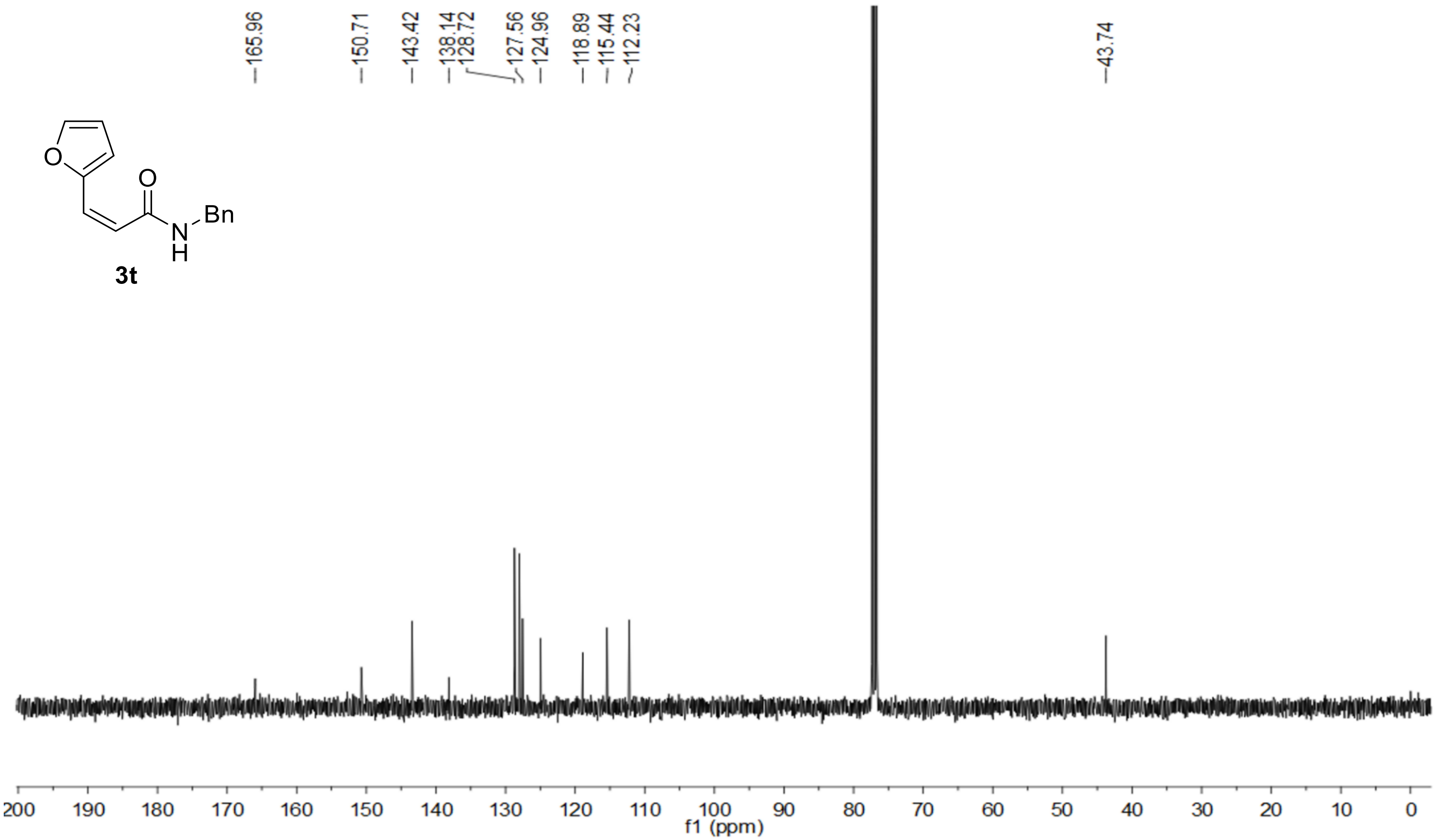

Supplementary Figure 69. ^{13}C NMR spectrum of (*Z*)-*N*-benzyl-3-(furan-2-yl)acrylamide **(3t)** in $CDCl_3$ (100 MHz) at 23℃.

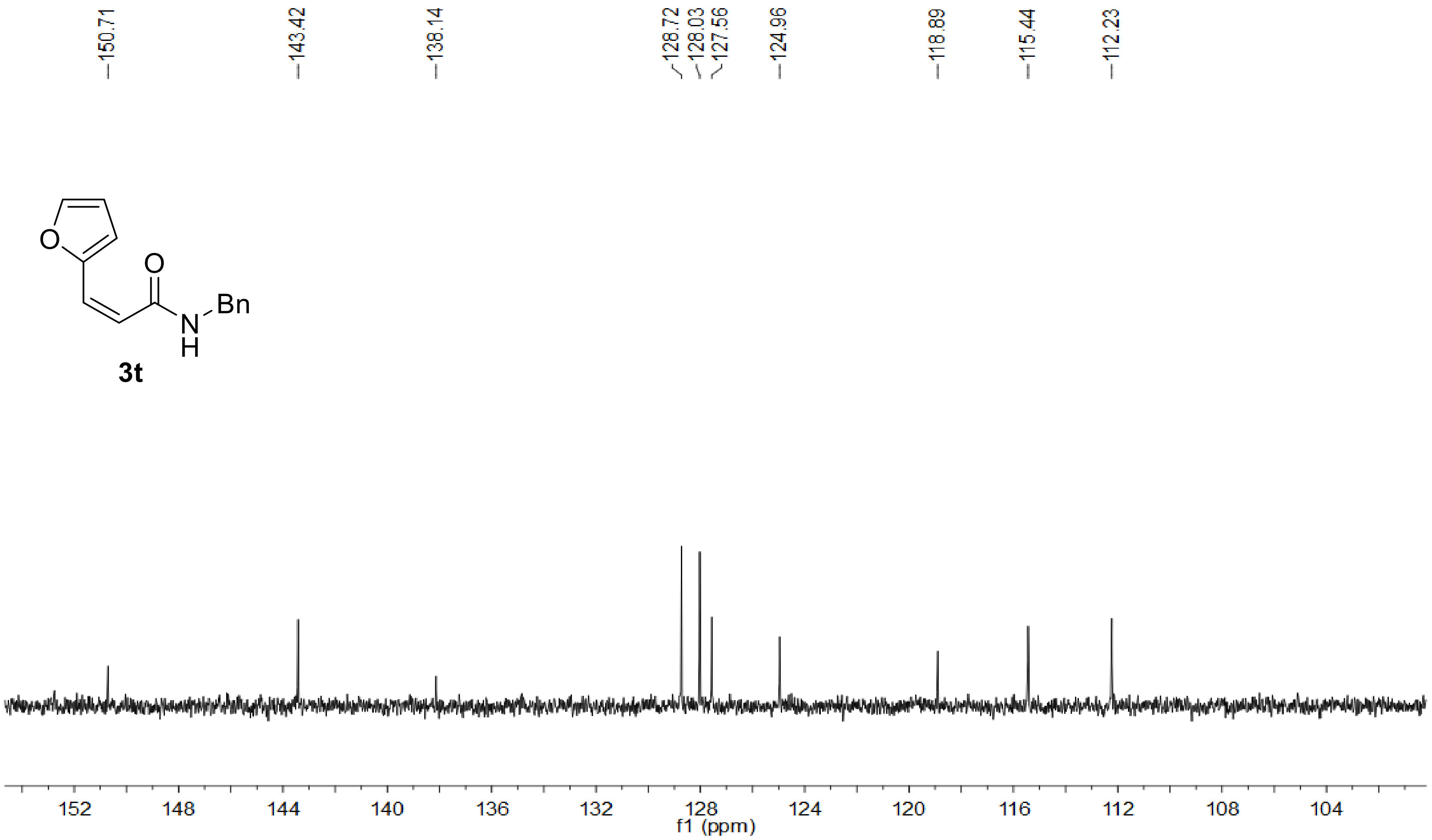

Supplementary Figure 70. Local magnification ^{13}C NMR spectrum of (*Z*)-*N*-benzyl-3-(furan-2-yl)acrylamide **(3t)** in $CDCl_3$ (100 MHz) at 23℃.

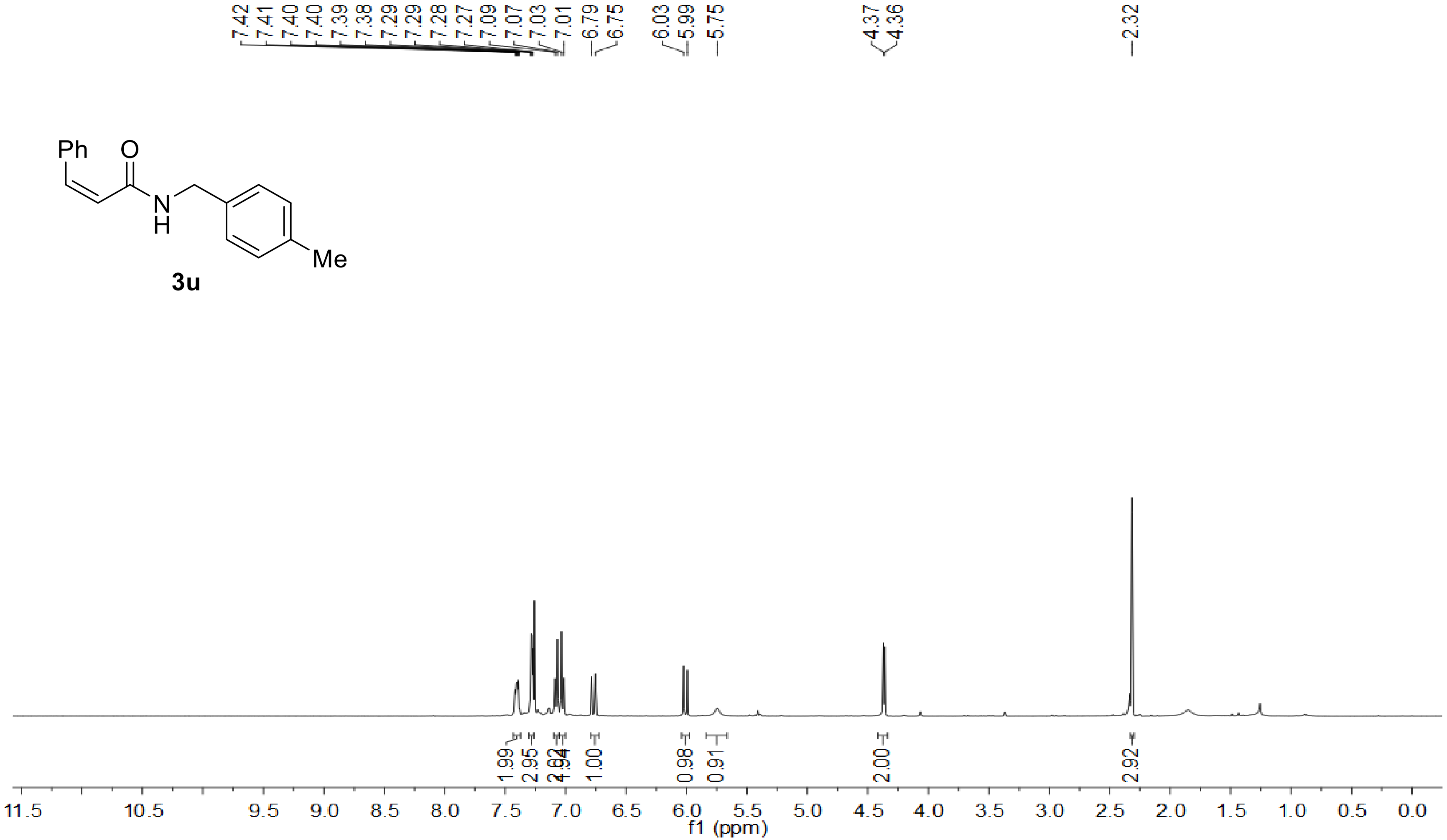

3u

Supplementary Figure 71. ^{1}H NMR spectrum of (*Z*)-*N*-(4-methylbenzyl)-3-phenylacrylamide **(3u)** in $CDCl_3$ (400 MHz) at 23℃.

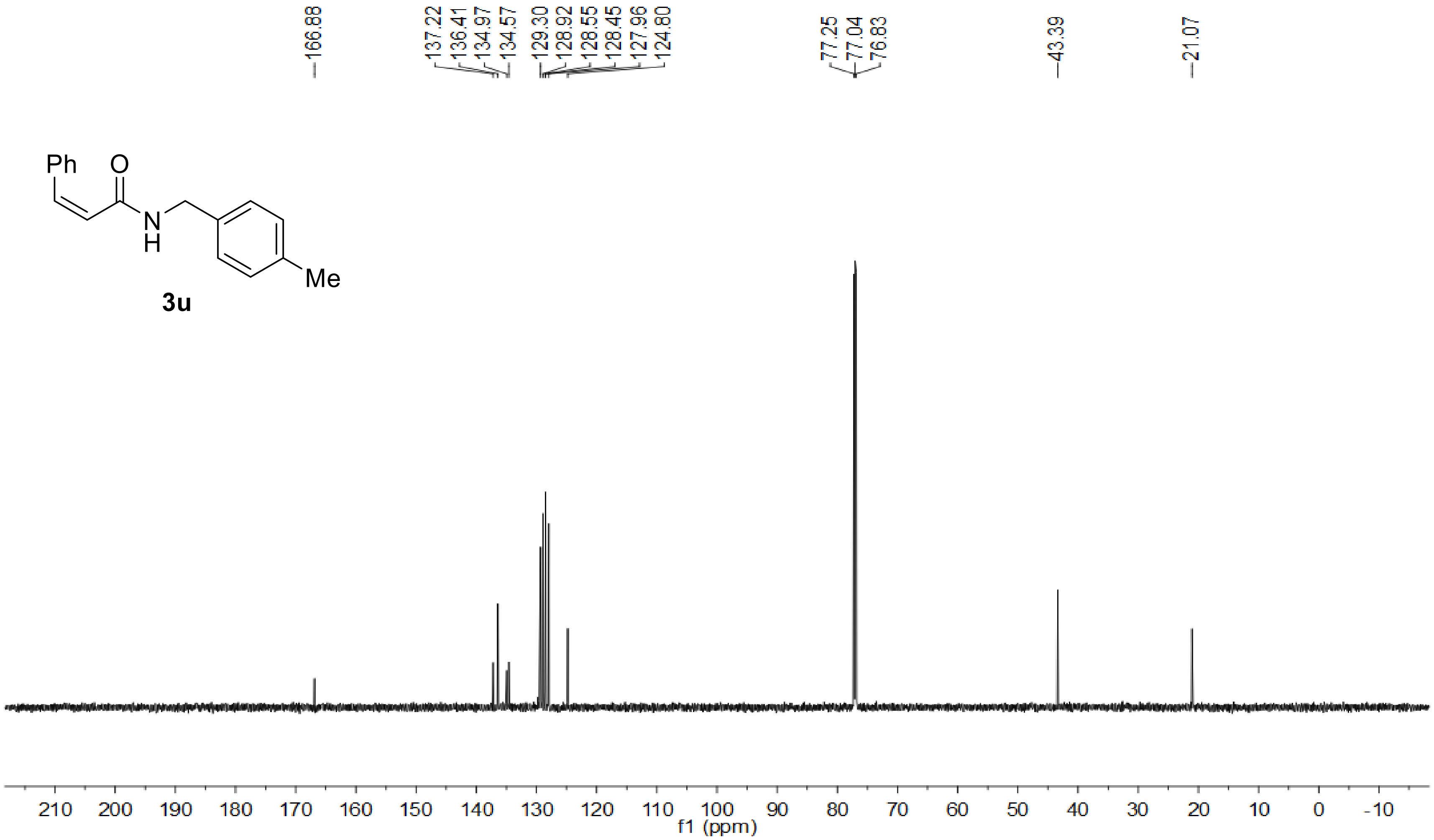

Supplementary Figure 72. ^{13}C NMR spectrum of (*Z*)-*N*-(4-methylbenzyl)-3-phenylacrylamide **(3u)** in $CDCl_3$ (100 MHz) at 23°C.

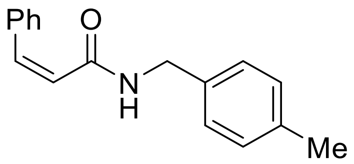

3u

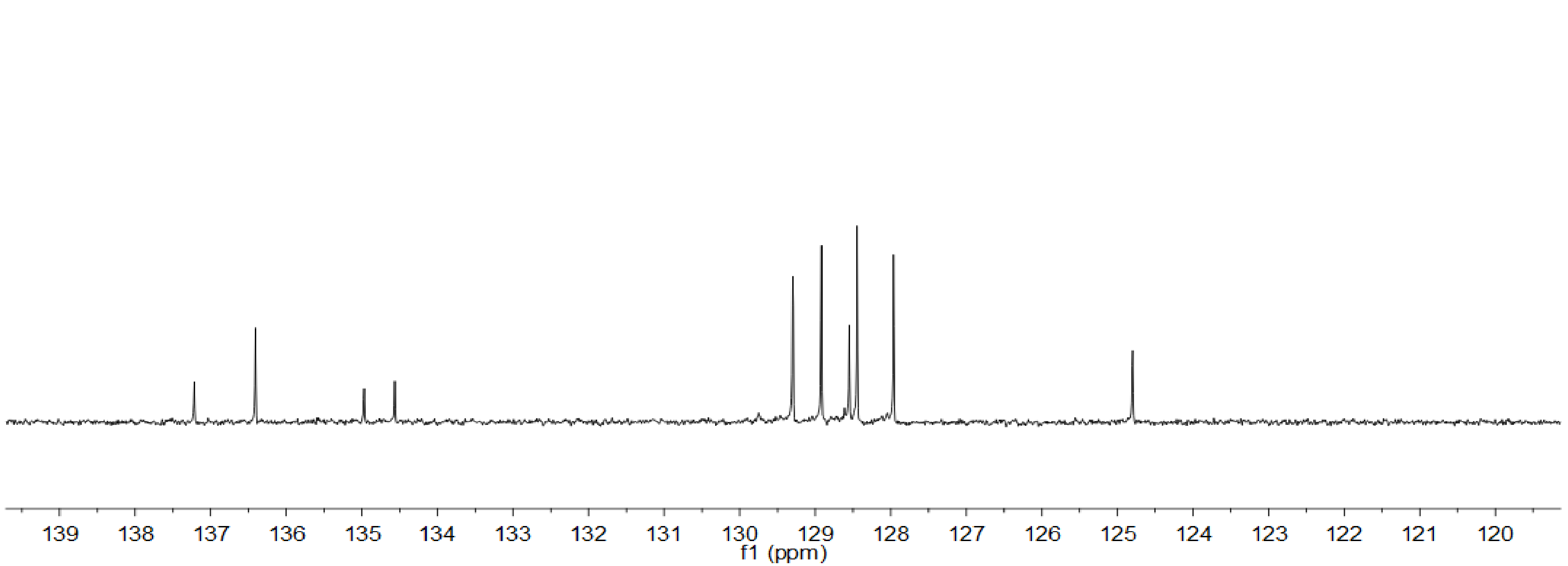

Supplementary Figure 73. Local magnification ^{13}C NMR spectrum of (*Z*)-*N*-(4-methylbenzyl)-3-phenylacrylamide **(3u)** in $CDCl_3$ (100 MHz) at 23℃.

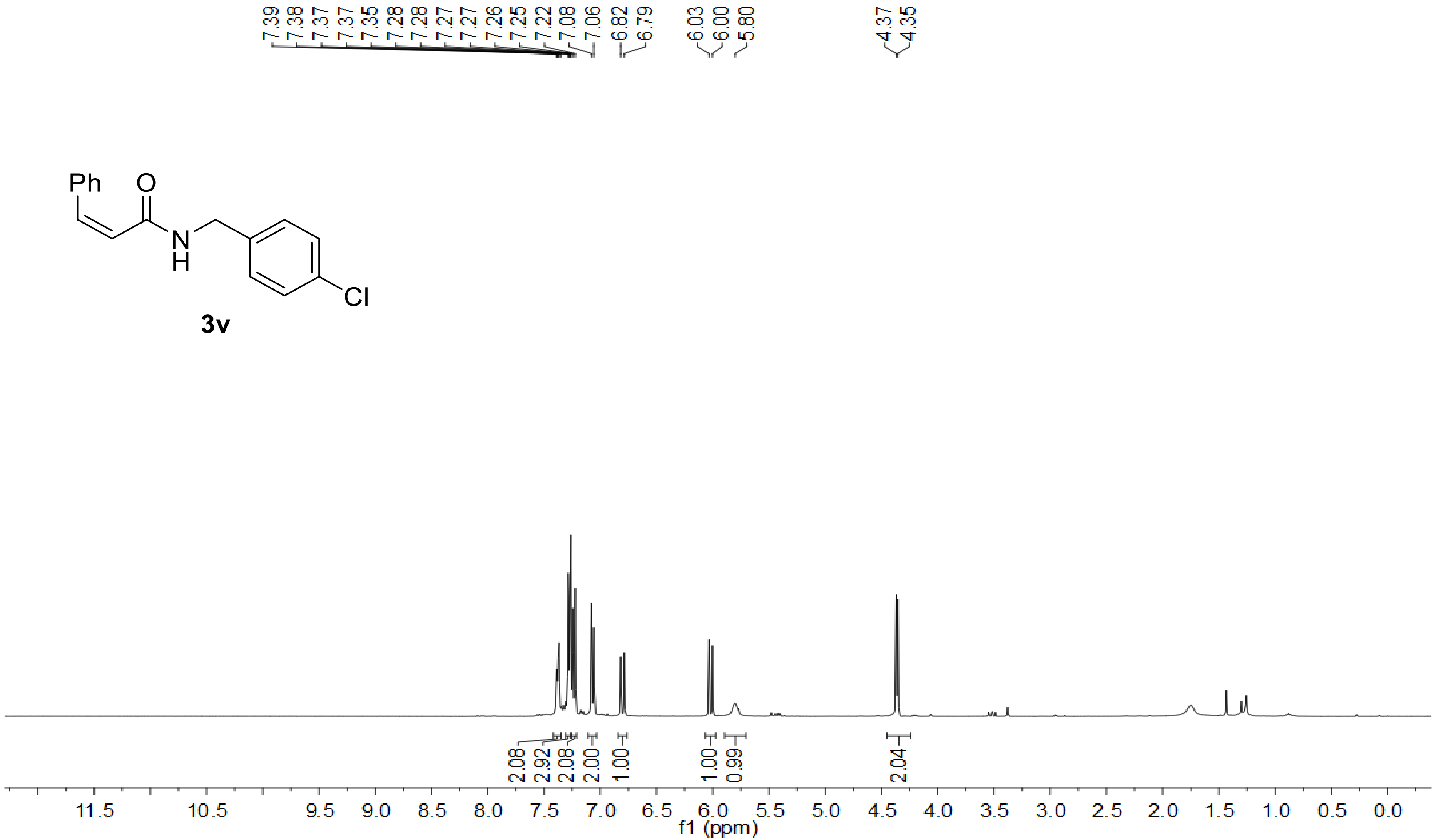

Supplementary Figure 74. ^{1}H NMR spectrum of (*Z*)-*N*-(4-chlorobenzyl)-3-phenylacrylamide (**3v**) in $CDCl_3$ (400 MHz) at 23℃.

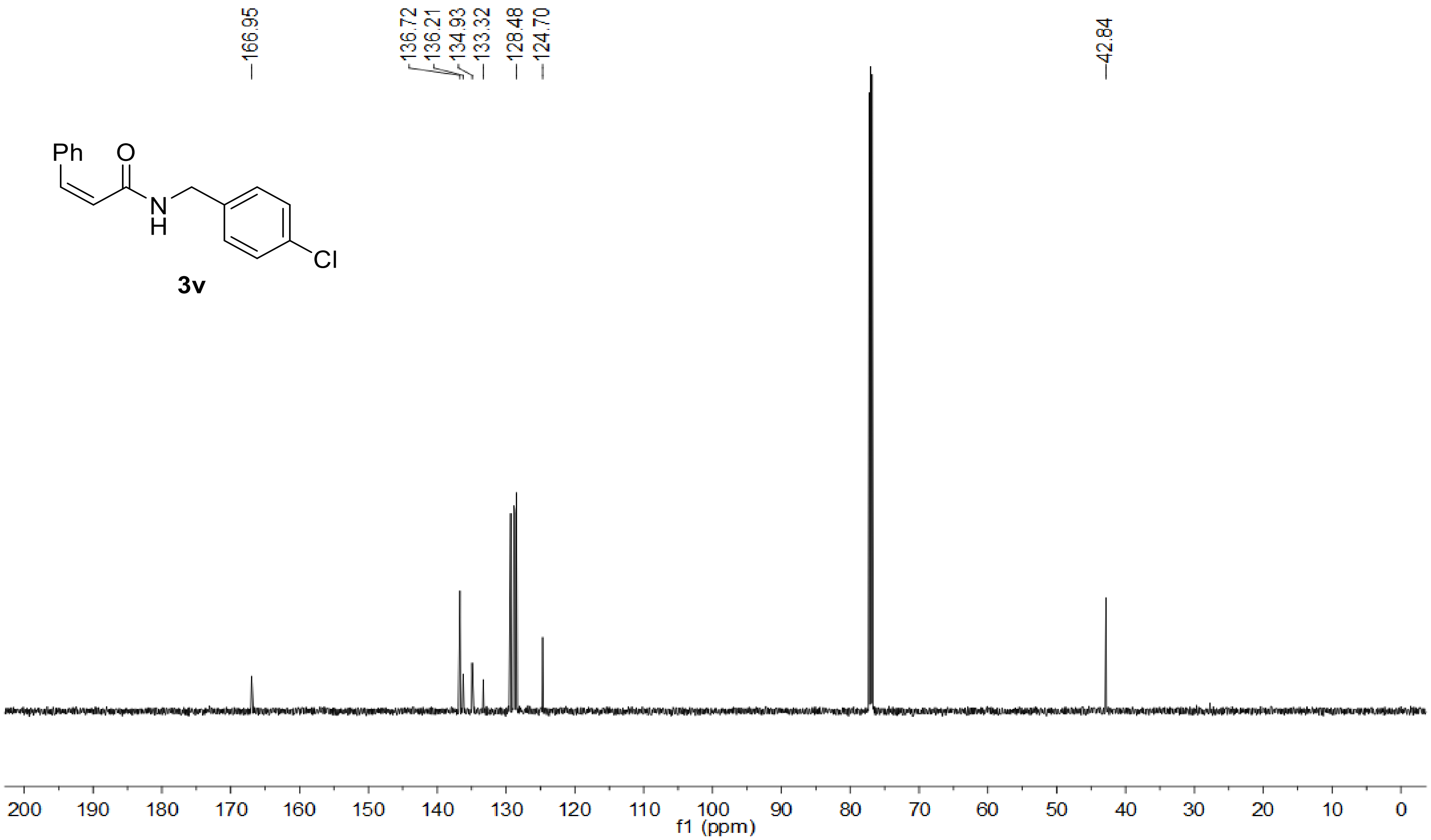

Supplementary Figure 75. ^{13}C NMR spectrum of (*Z*)-*N*-(4-chlorobenzyl)-3-phenylacrylamide (**3v**) in $CDCl_3$ (100 MHz) at 23°C.

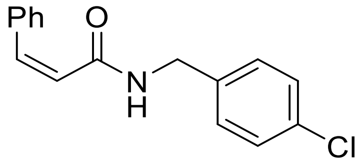

3v

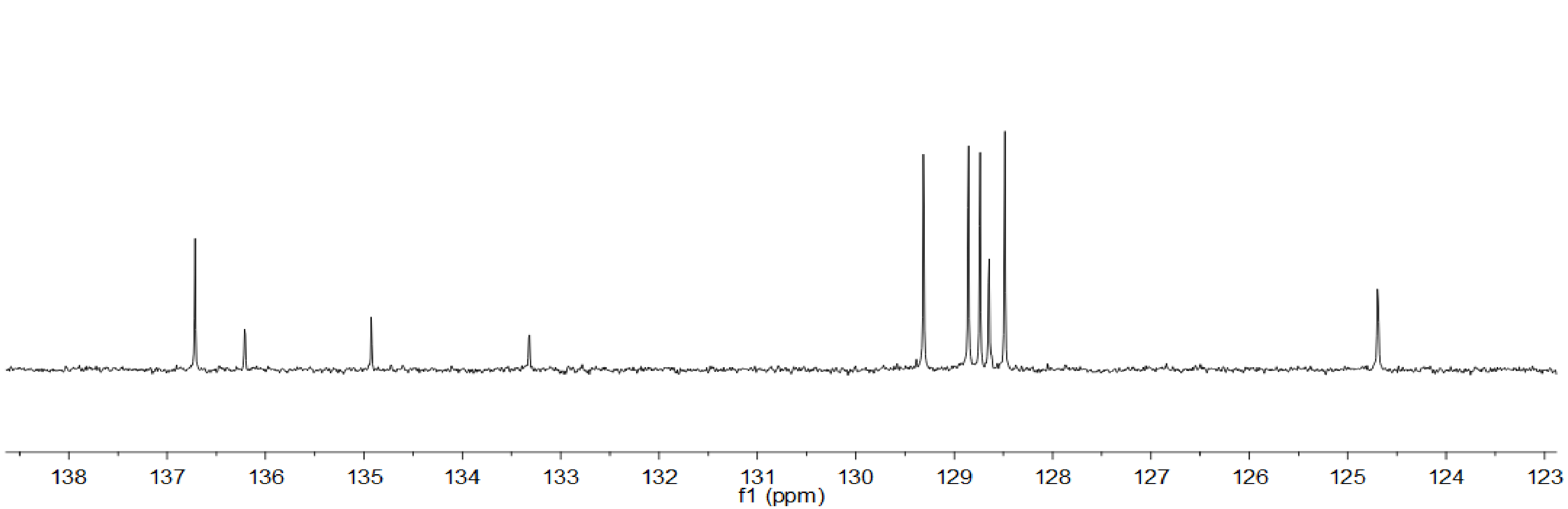

Supplementary Figure 76. Local magnification ^{13}C NMR spectrum of (*Z*)-*N*-(4-chlorobenzyl)-3-phenylacrylamide (**3v**) in $CDCl_3$ (100 MHz) at 23℃.

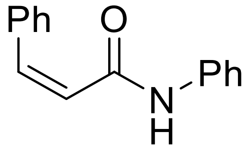

3w

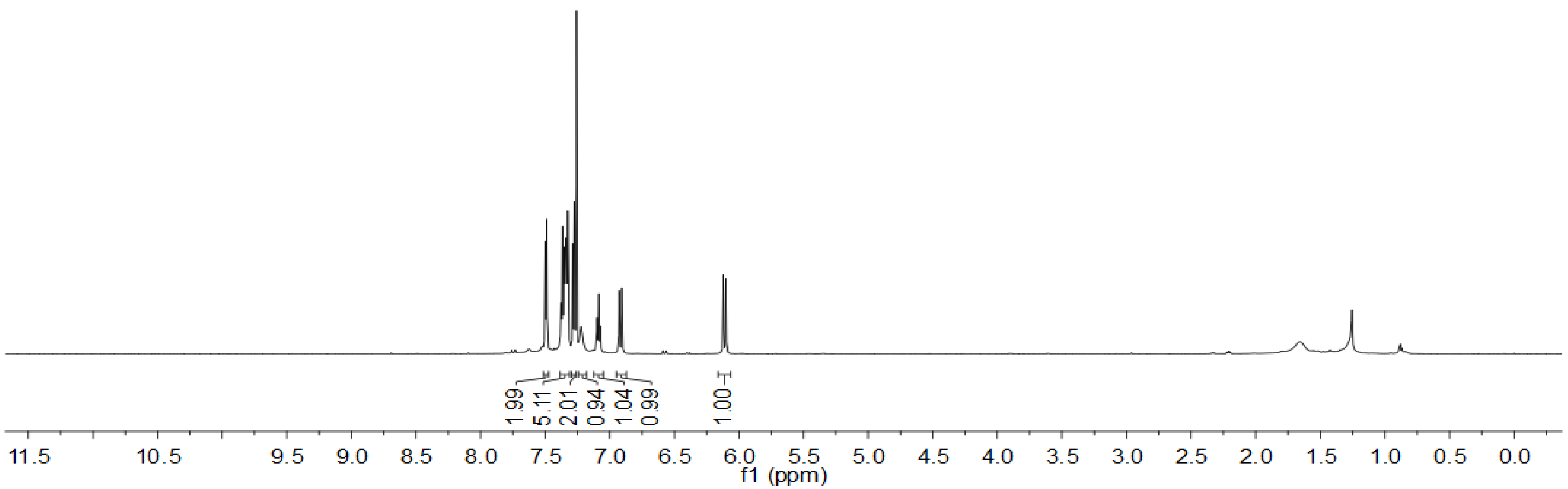

Supplementary Figure 77. ^{1}H NMR spectrum of (*Z*)-*N*,3-diphenylacrylamide (**3w**) in $CDCl_3$ (600 MHz) at 23°C.

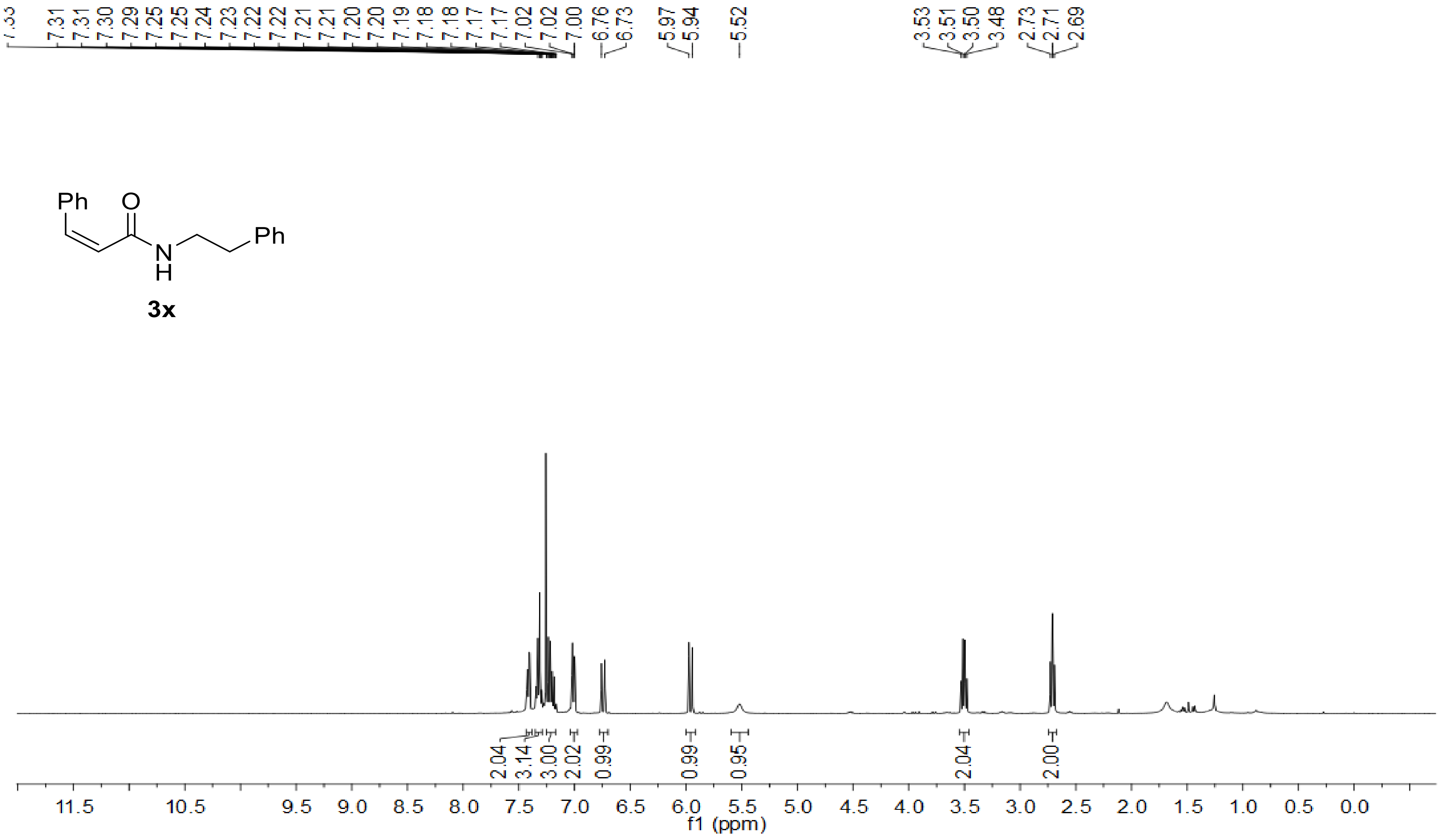

Supplementary Figure 78. ^{1}H NMR spectrum of (*Z*)-*N*-phenethyl-3-phenylacrylamide (**3x**) in $CDCl_3$ (400 MHz) at 23°C.

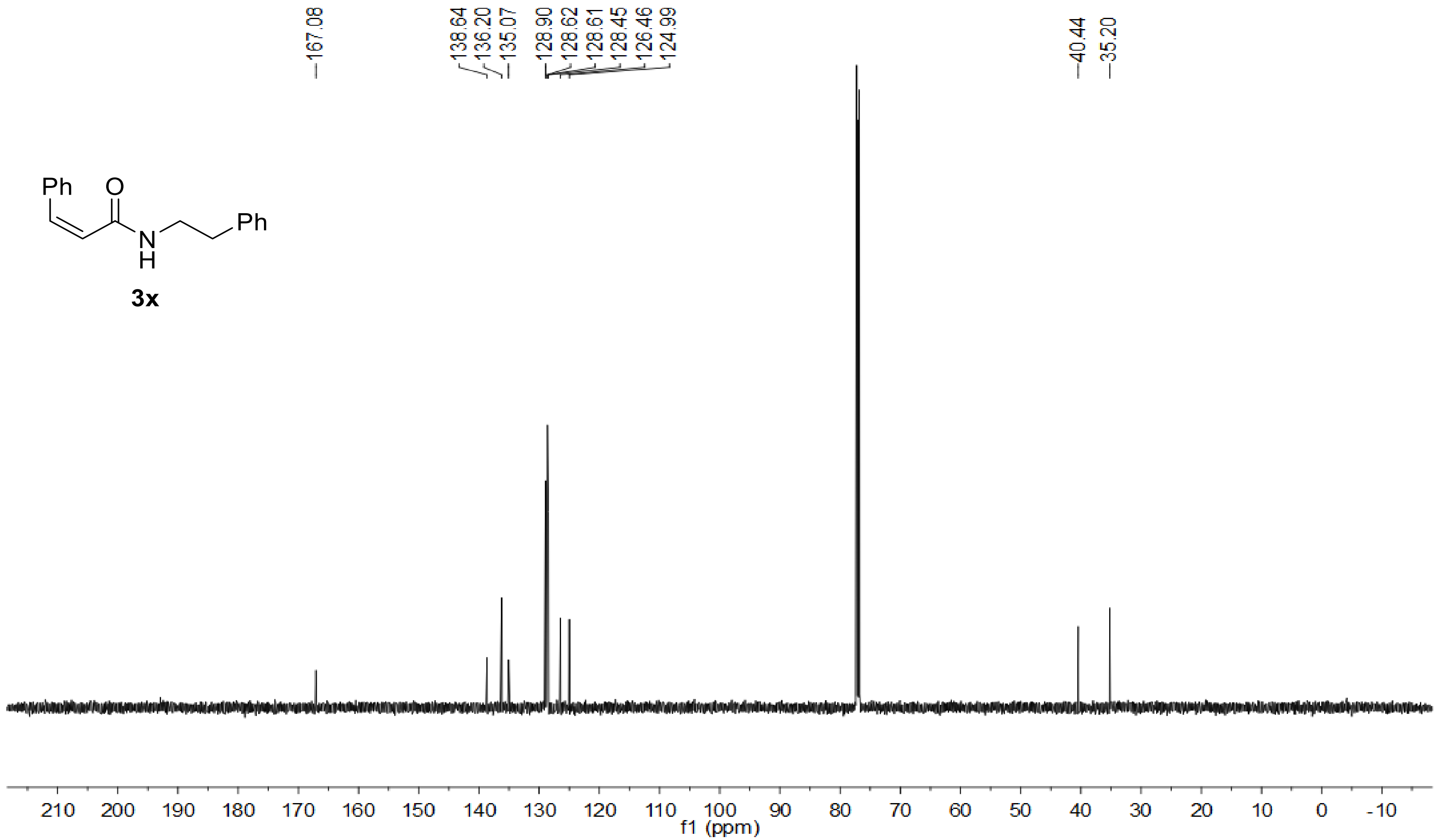

Supplementary Figure 79. ^{13}C NMR spectrum of (*Z*)-*N*-phenethyl-3-phenylacrylamide (**3x**) in $CDCl_3$ (100 MHz) at 23°C.

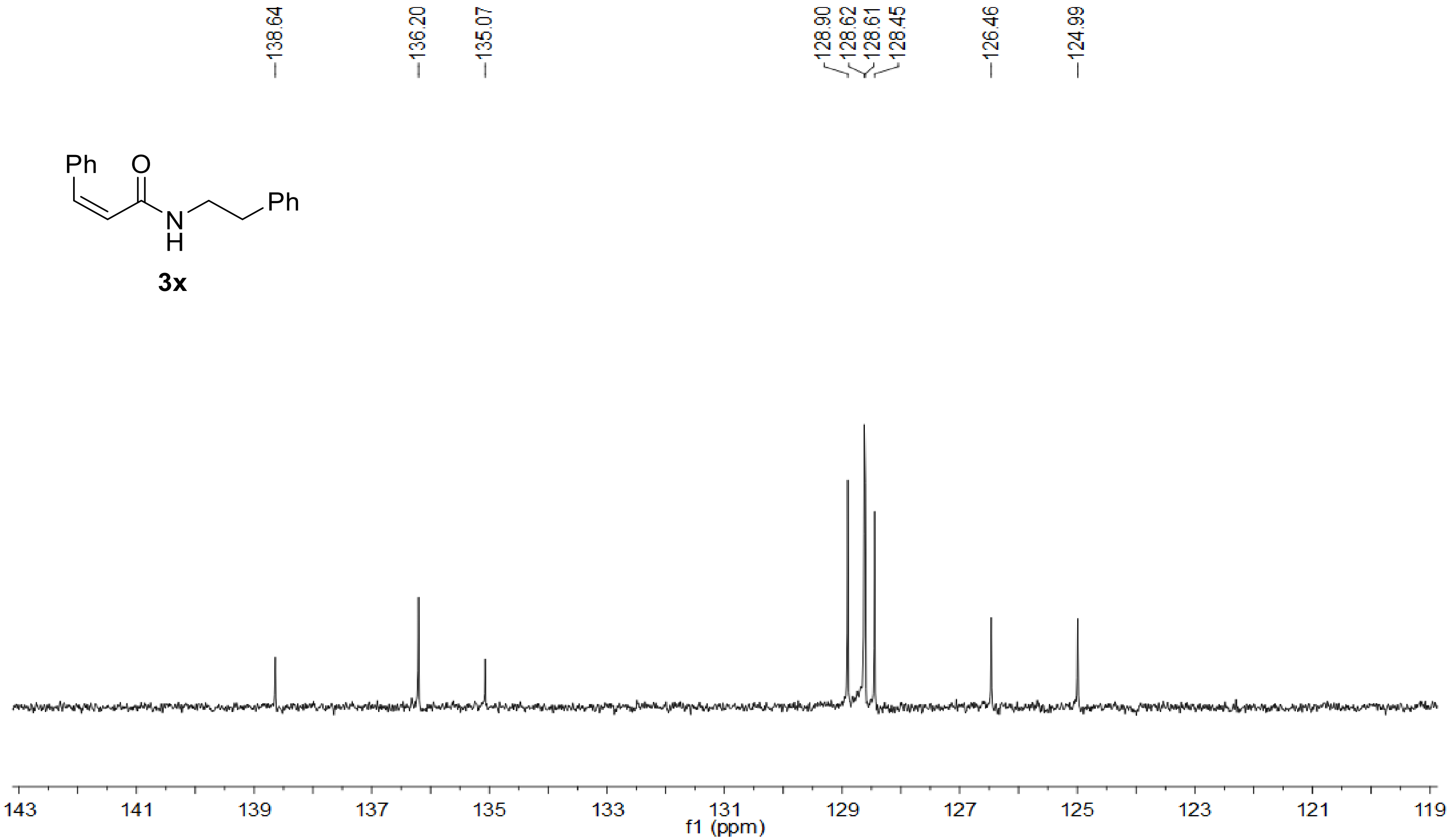

Supplementary Figure 80. Local magnification ^{13}C NMR spectrum of (*Z*)-*N*-phenethyl-3-phenylacrylamide (**3x**) in $CDCl_3$ (100 MHz) at 23°C.

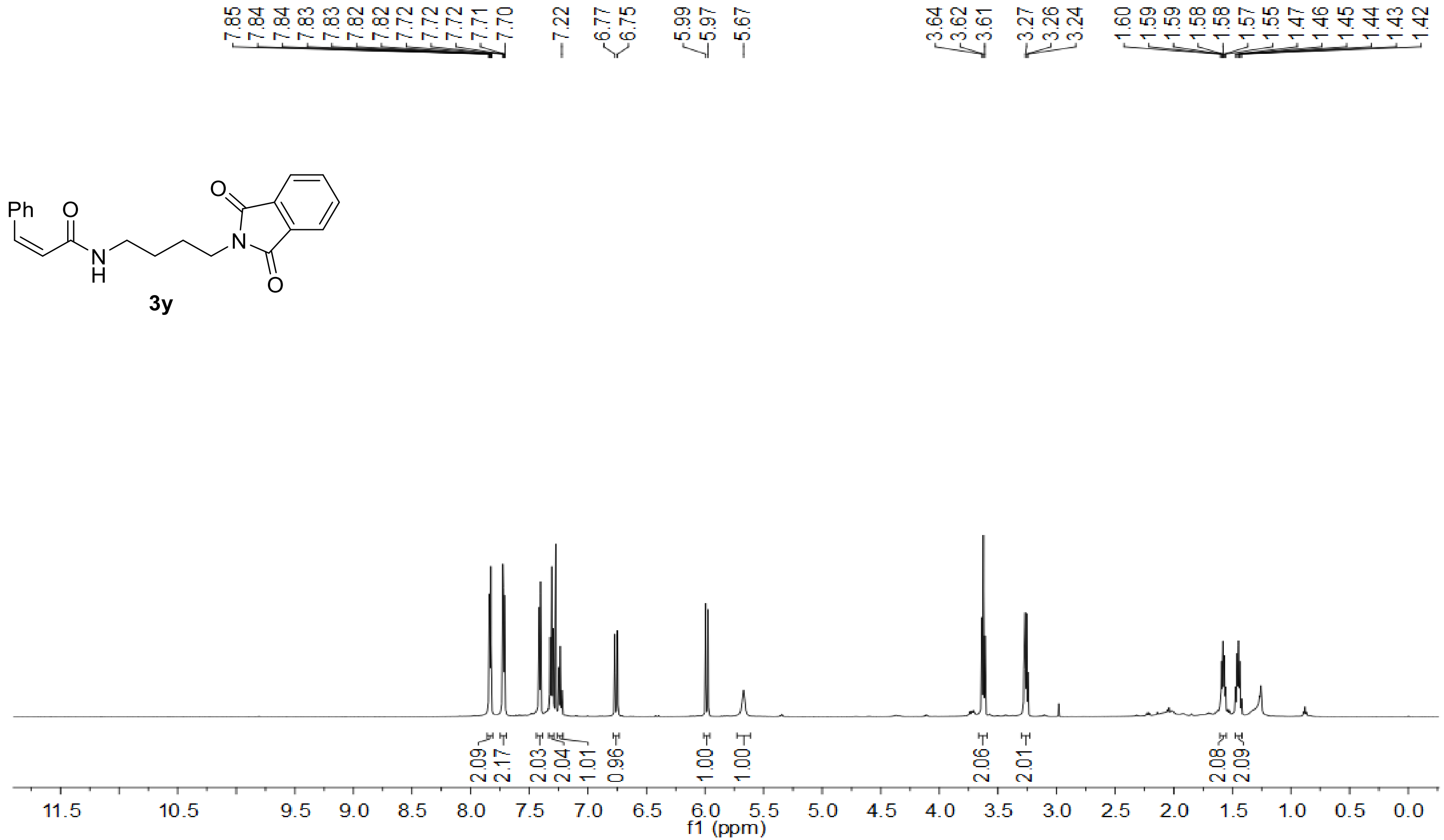

Supplementary Figure 81. ^{1}H NMR spectrum of (*Z*)-*N*-(4-(1,3-dioxoisoindolin-2-yl)butyl)-3-phenylacrylamide (**3y**) in $CDCl_3$ (600 MHz) at 23℃.

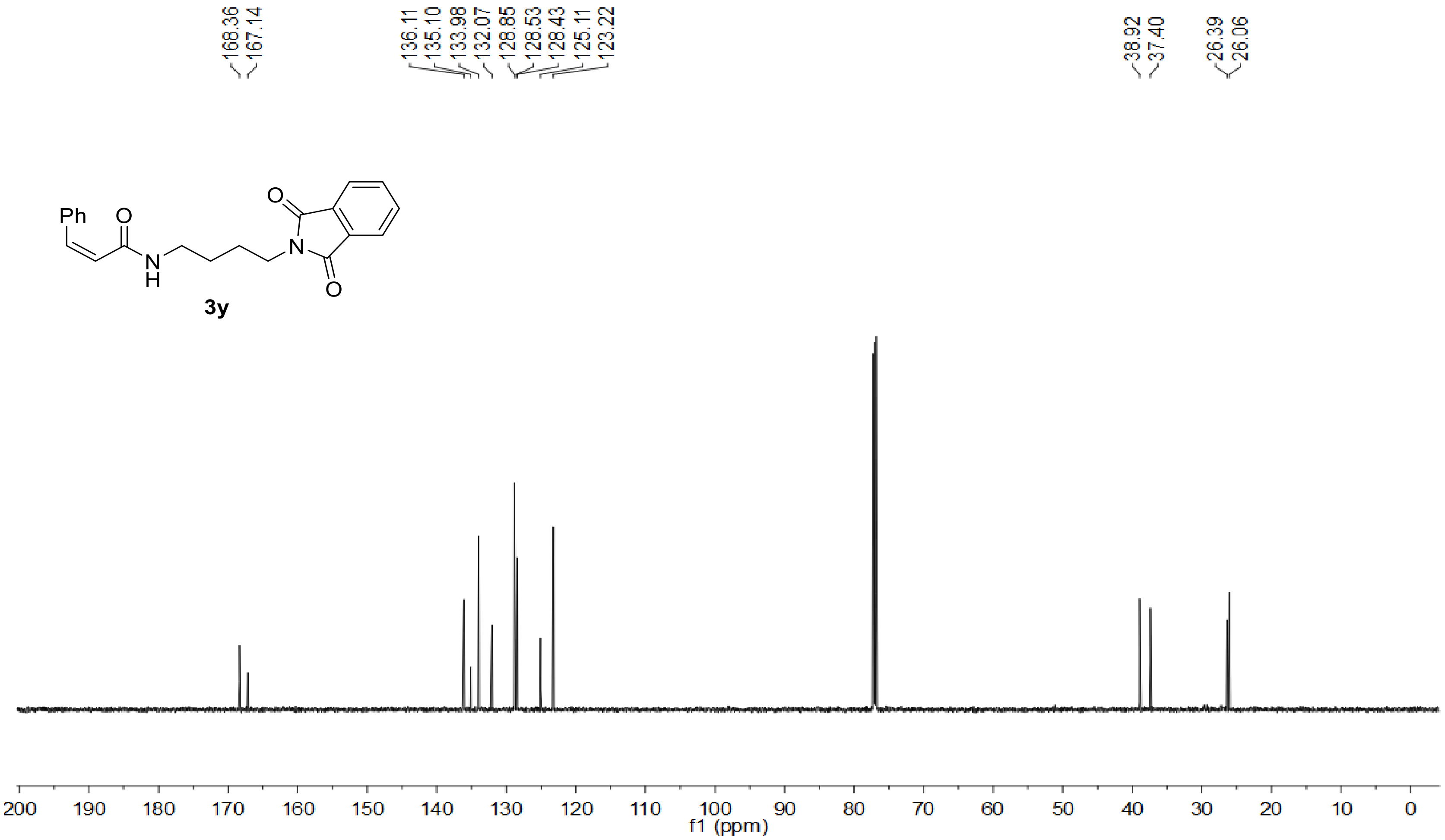

Supplementary Figure 82. ^{13}C NMR spectrum of (*Z*)-*N*-(4-(1,3-dioxoisoindolin-2-yl)butyl)-3-phenylacrylamide (**3y**) in $CDCl_3$ (100 MHz) at 23℃.

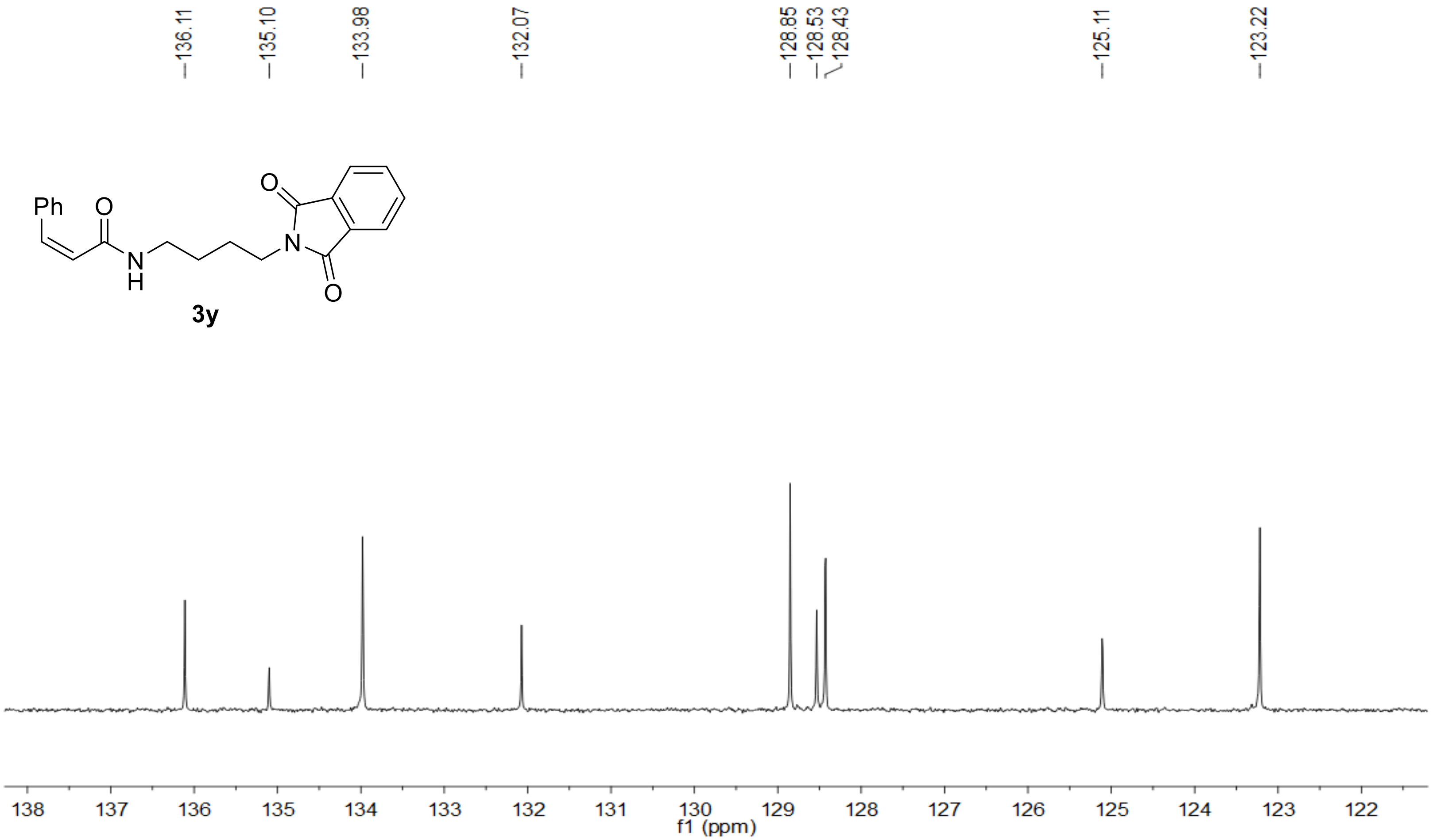

Supplementary Figure 83. Local magnification ^{13}C NMR spectrum of (*Z*)-*N*-(4-(1,3-dioxoisoindolin-2-yl)butyl)-3-phenylacrylamide (**3y**) in $CDCl_3$ (100 MHz) at 23℃.

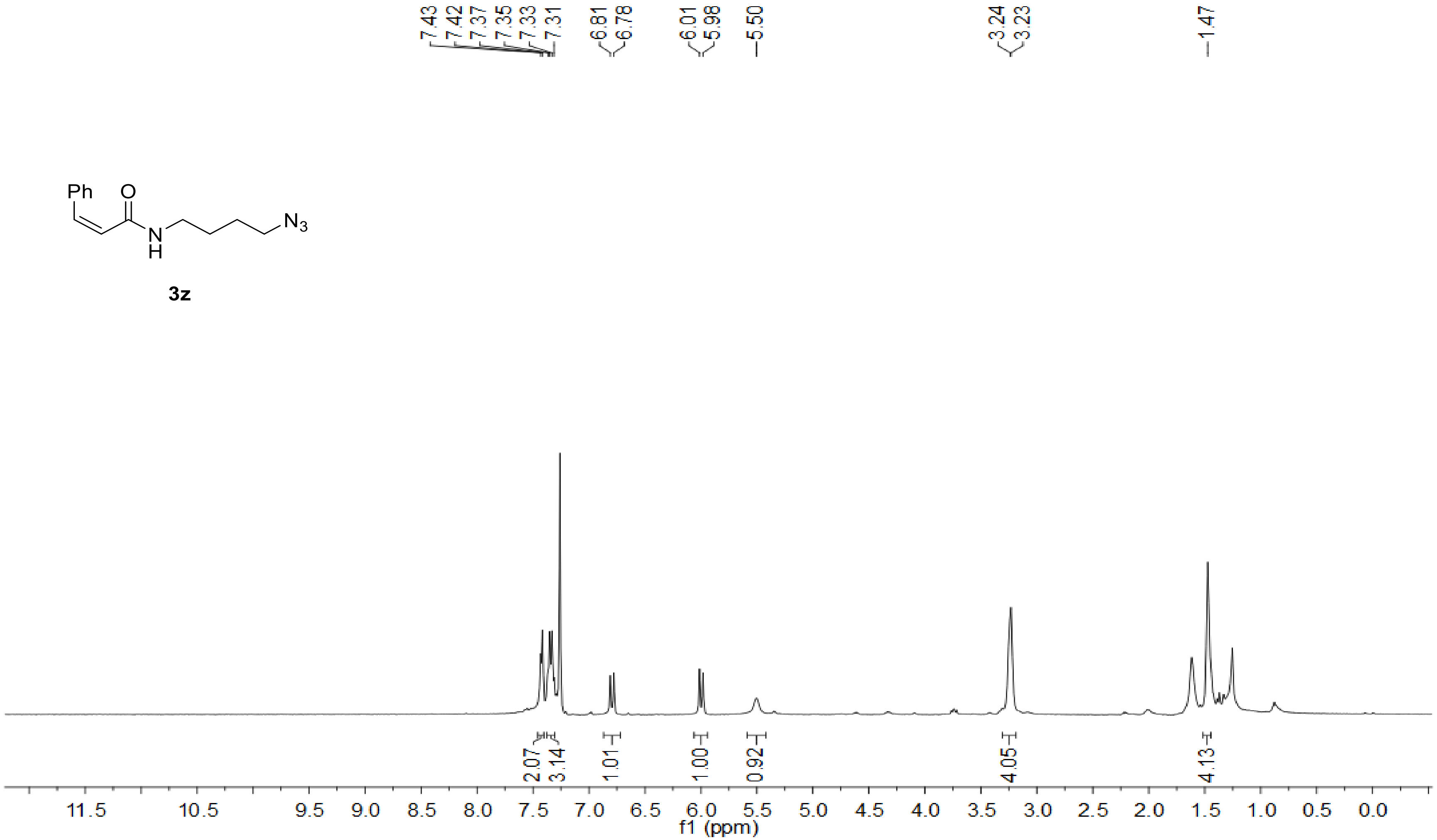

Supplementary Figure 84. ^{1}H NMR spectrum of (*Z*)-*N*-(4-azidobutyl)-3-phenylacrylamide (**3z**) in $CDCl_3$ (400 MHz) at 23°C.

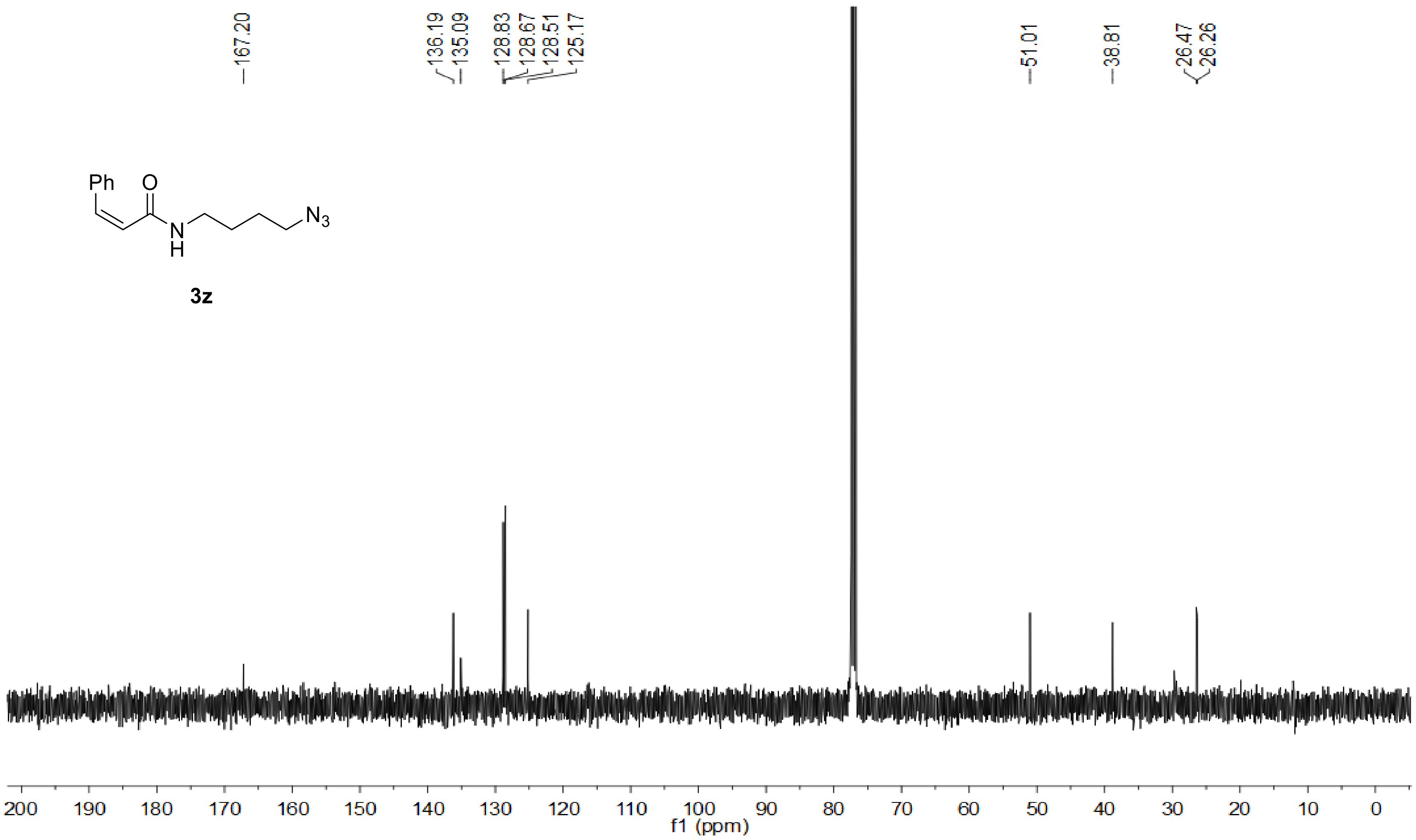

Supplementary Figure 85. ^{13}C NMR spectrum of (*Z*)-*N*-(4-azidobutyl)-3-phenylacrylamide (**3z**) in $CDCl_3$ (100 MHz) at 23℃.

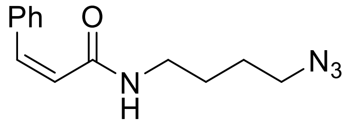

3z

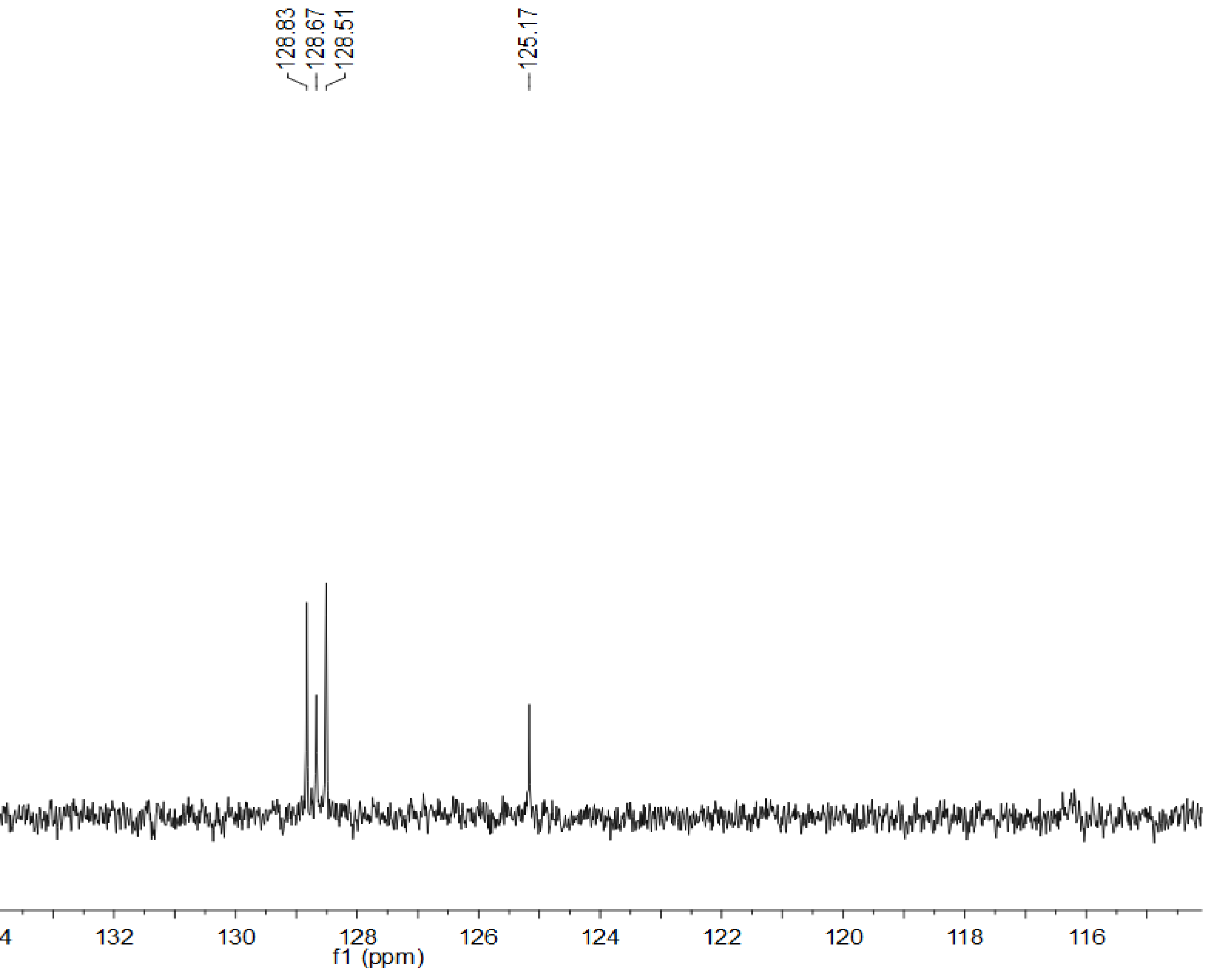

Supplementary Figure 86. Local magnification ^{13}C NMR spectrum of (*Z*)-*N*-(4-azidobutyl)-3-phenylacrylamide (**3z**) in $CDCl_3$ (100 MHz) at 23℃.

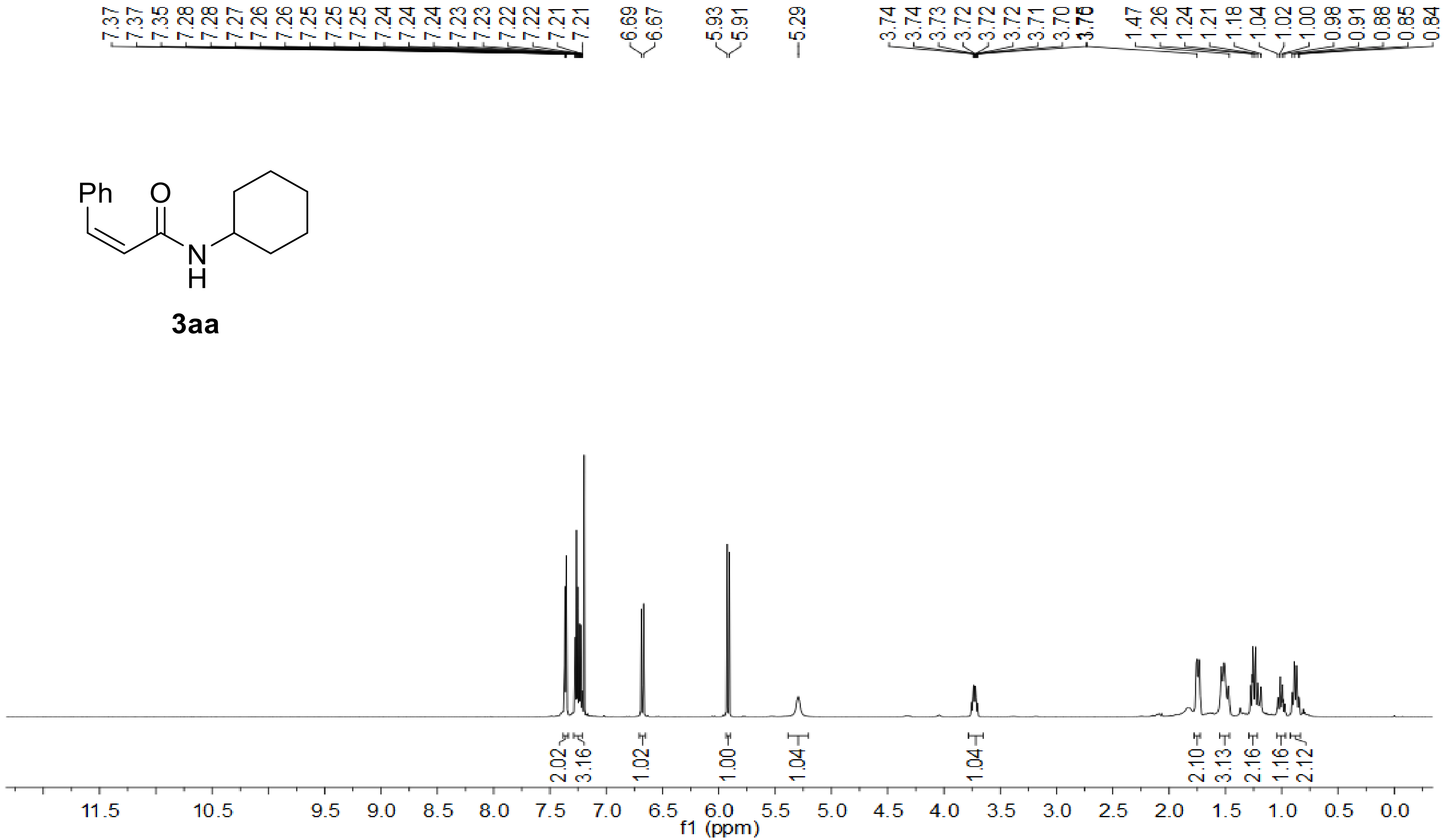

3aa

Supplementary Figure 87. ^{1}H NMR spectrum of (*Z*)-*N*-cyclohexyl-3-phenylacrylamide (**3aa**) in $CDCl_3$ (600 MHz) at 23°C.

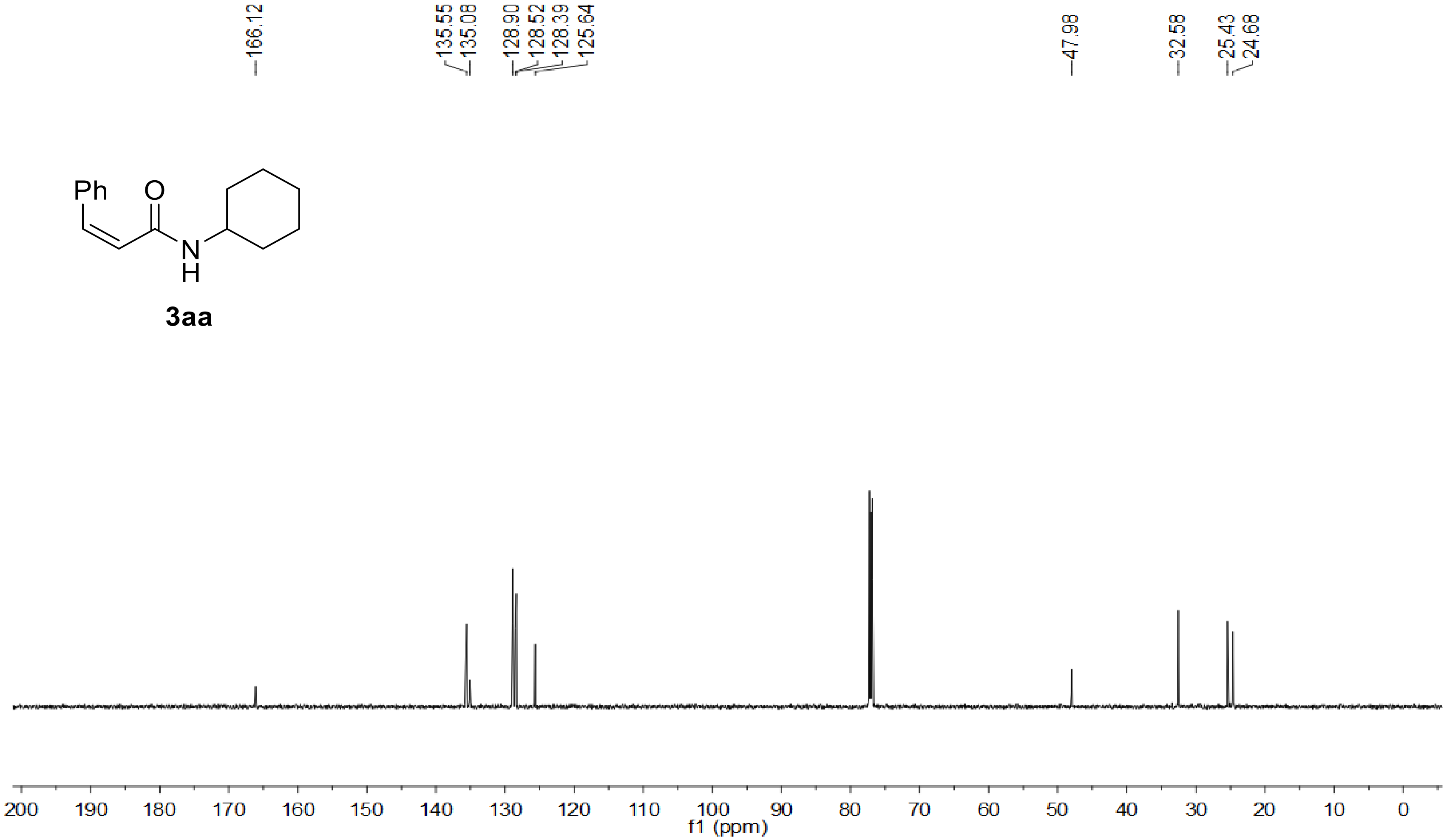

Supplementary Figure 88. ^{13}C NMR spectrum of (*Z*)-*N*-cyclohexyl-3-phenylacrylamide (**3aa**) in $CDCl_3$ (100 MHz) at 23℃.

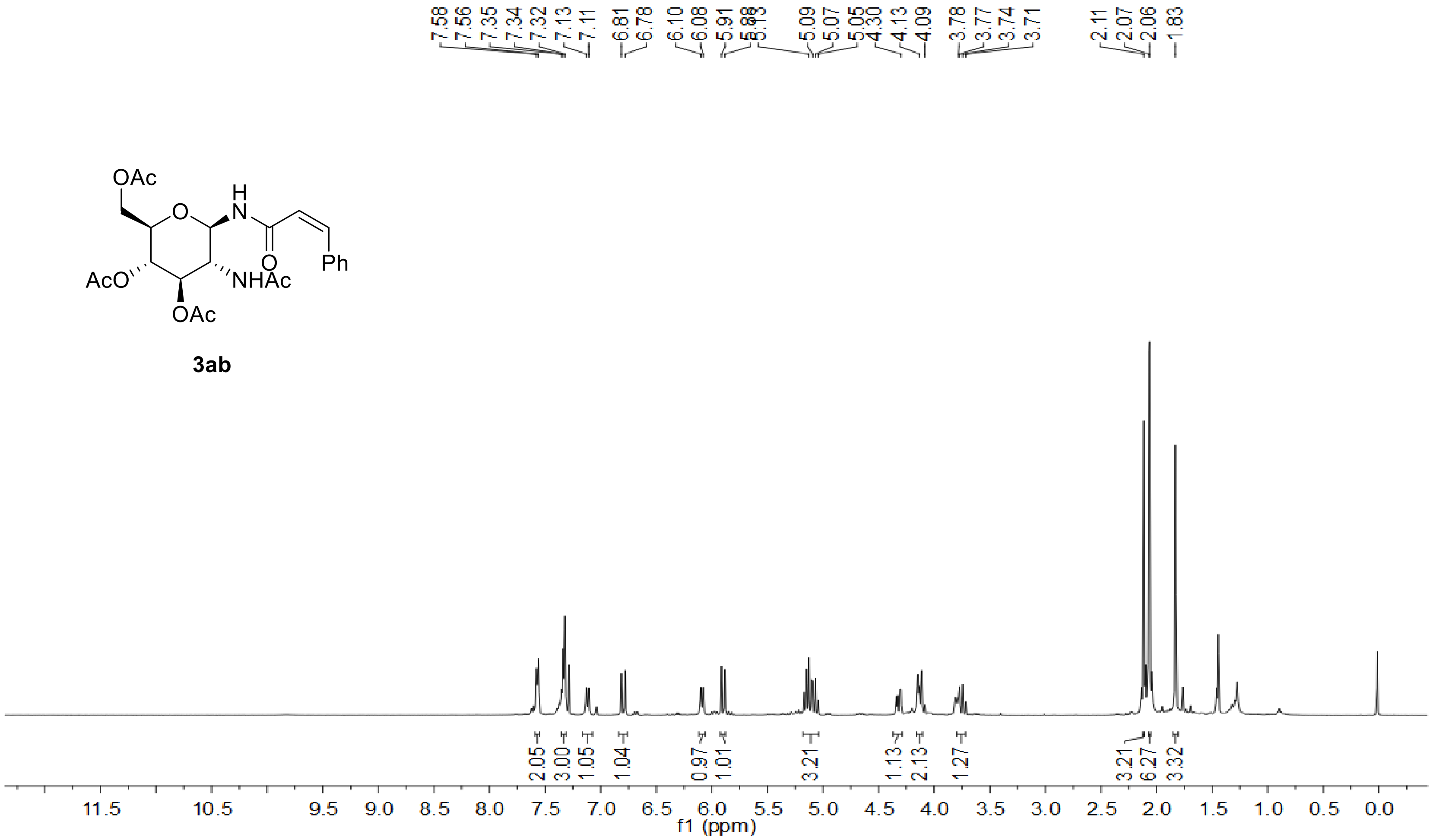

Supplementary Figure 89. ^{1}H NMR spectrum of (2*R*,3*S*,4*R*,5*R*,6*R*)-5-acetamido-2-(acetoxymethyl)-6-((*Z*)-3-phenylacrylamido)tetrahydro-2*H*-pyran-3,4-diyl diacetate (**3ab**) in $CDCl_3$ (400 MHz) at 23℃.

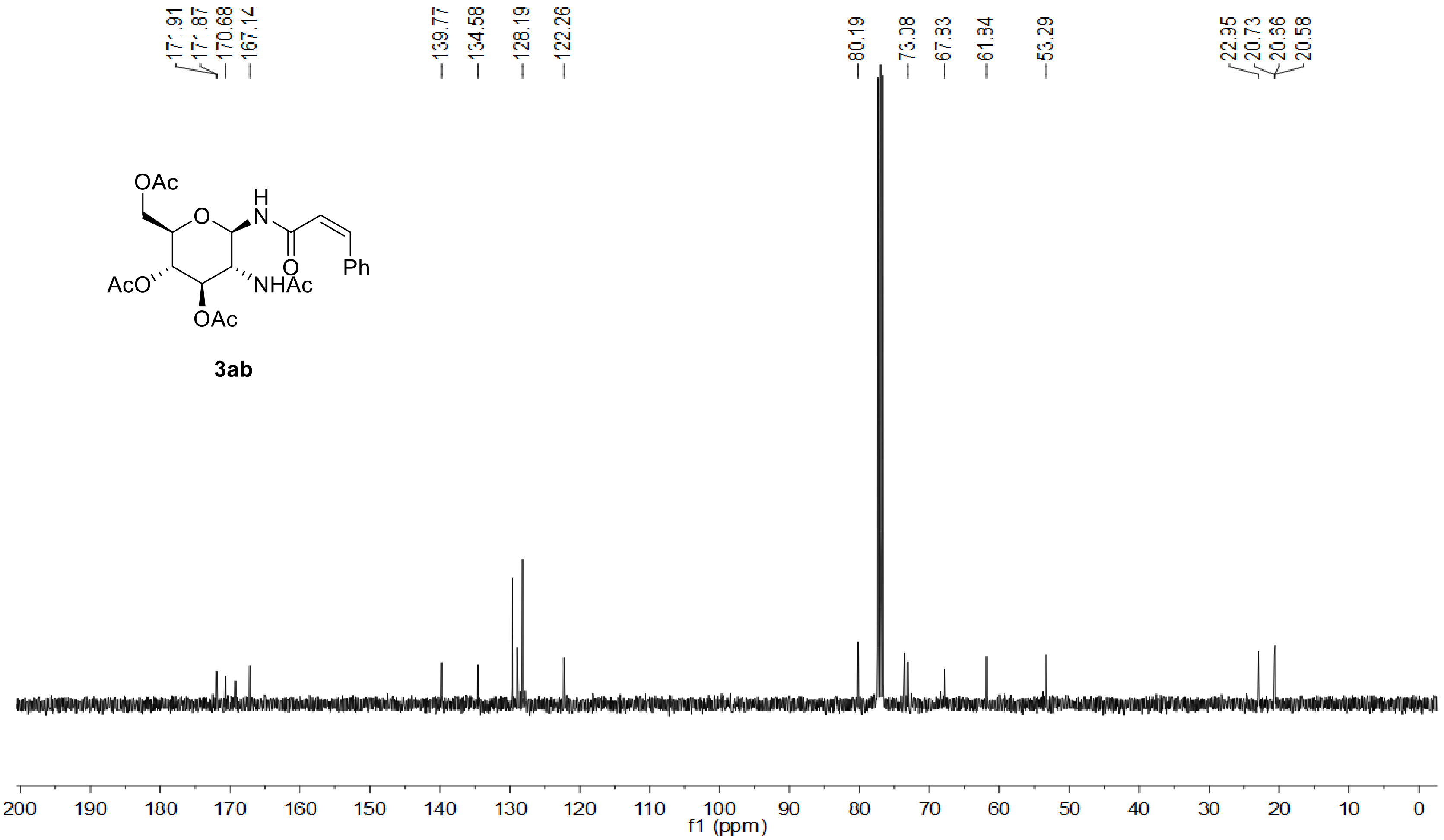

Supplementary Figure 90. ^{13}C NMR spectrum of (2*R*,3*S*,4*R*,5*R*,6*R*)-5-acetamido-2-(acetoxymethyl)-6-((*Z*)-3-phenylacrylamido)tetrahydro-2*H*-pyran-3,4-diyl diacetate (**3ab**) in $CDCl_3$ (100 MHz) at 23℃.

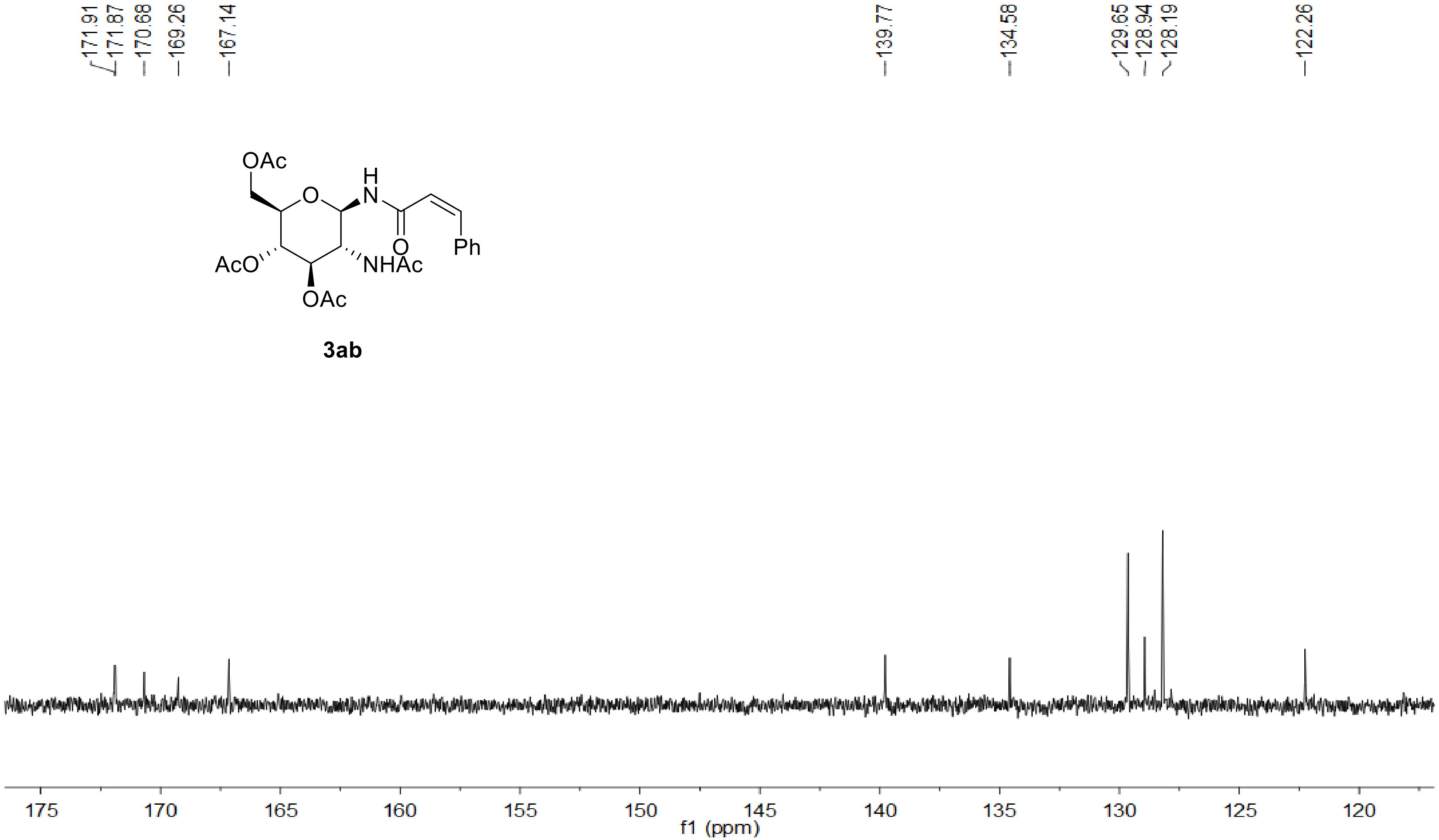

Supplementary Figure 91. Local magnification ^{13}C NMR spectrum of (*2R*,*3S*,*4R*,*5R*,*6R*)-5-acetamido-2-(acetoxymethyl)-6-((*Z*)-3-phenylacrylamido)tetrahydro-2*H*-pyran-3,4-diyl diacetate (**3ab**) in $CDCl_3$ (100 MHz) at 23℃.

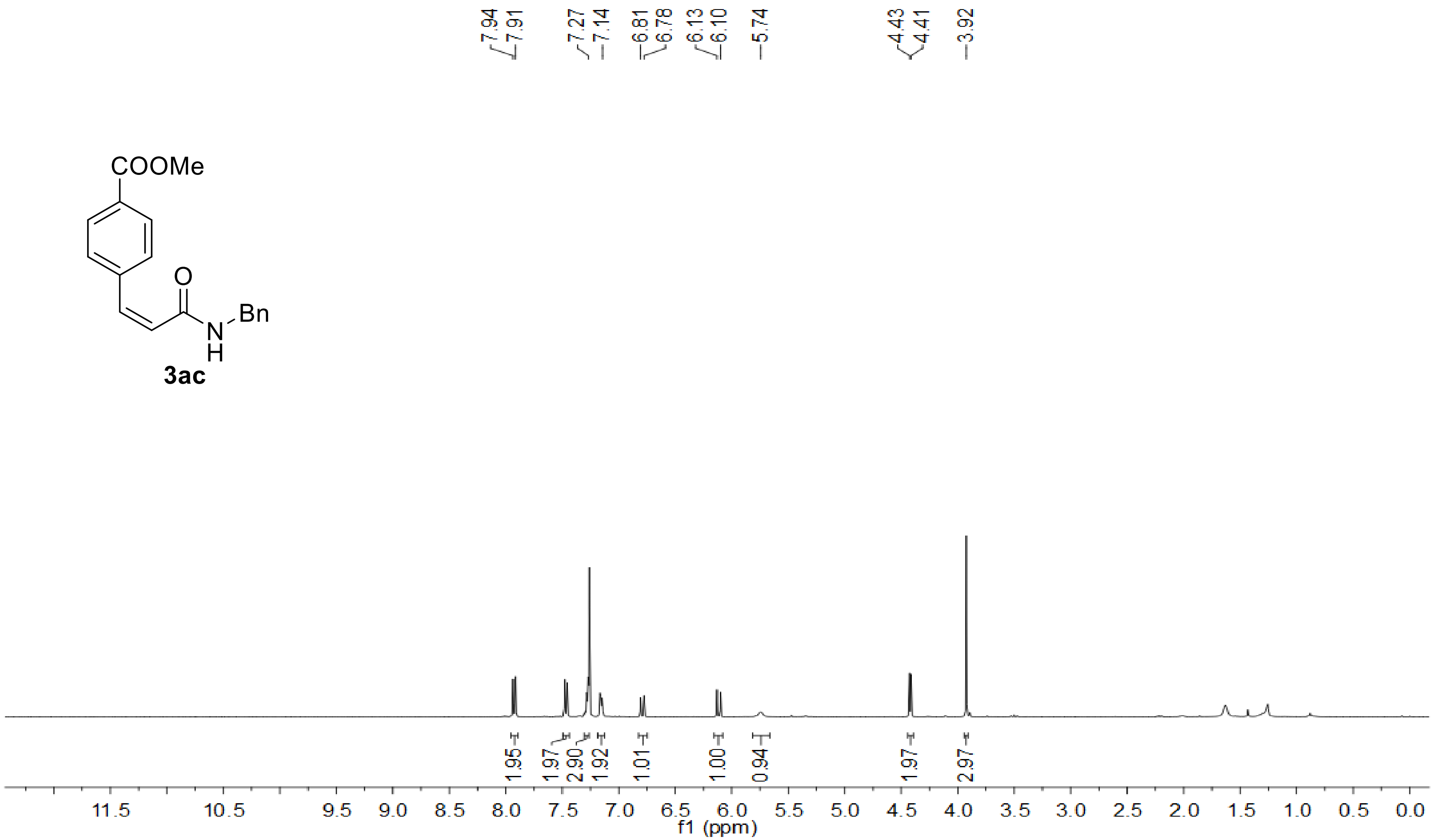

Supplementary Figure 92. ^{1}H NMR spectrum of methyl (*Z*)-4-(3-(benzylamino)-3-oxoprop-1-en-1-yl)benzoate (**3ac**) in $CDCl_3$ (400 MHz) at 23℃.

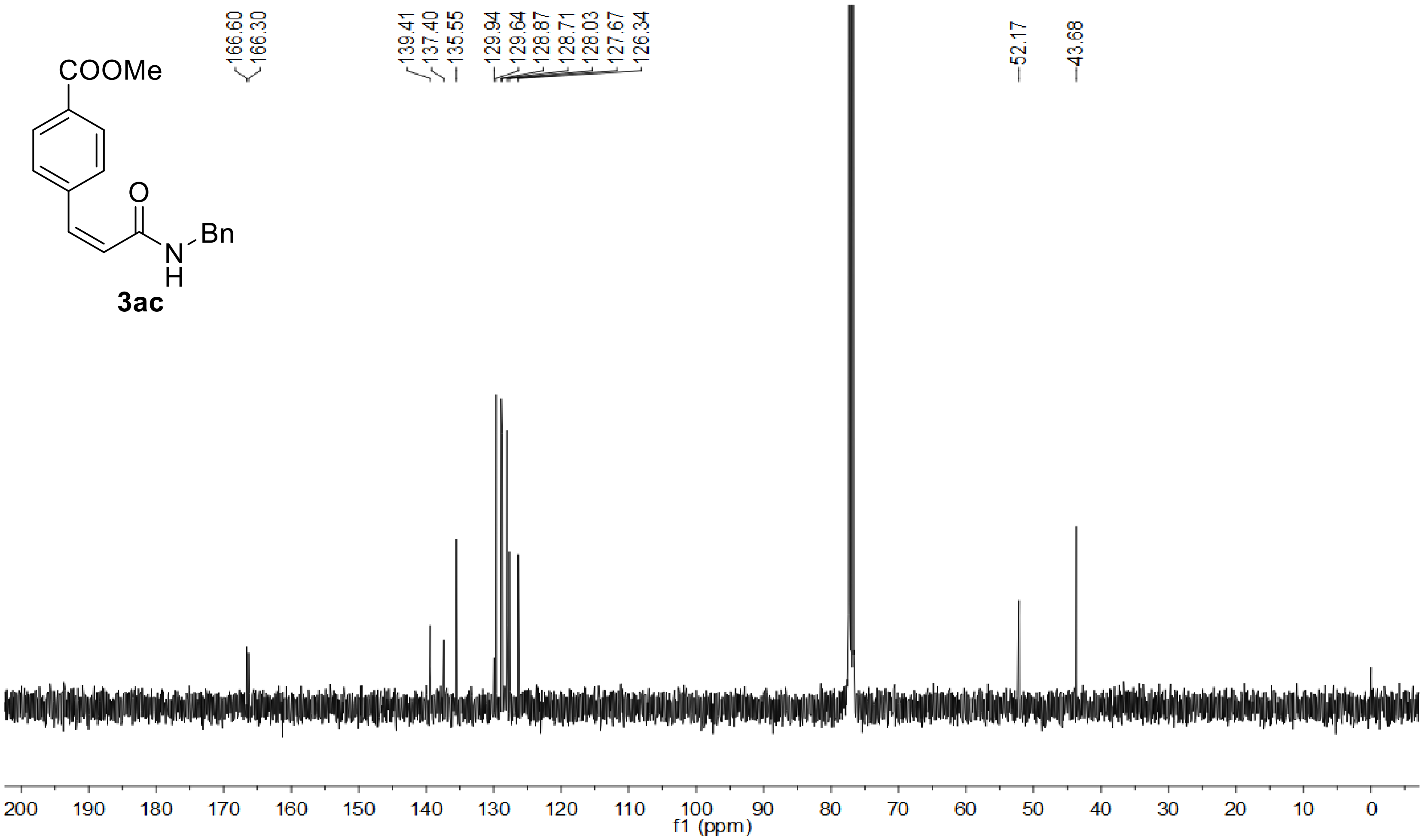

Supplementary Figure 93. ^{13}C NMR spectrum of methyl (*Z*)-4-(3-(benzylamino)-3-oxoprop-1-en-1-yl)benzoate (**3ac**) in $CDCl_3$ (100 MHz) at 23°C.

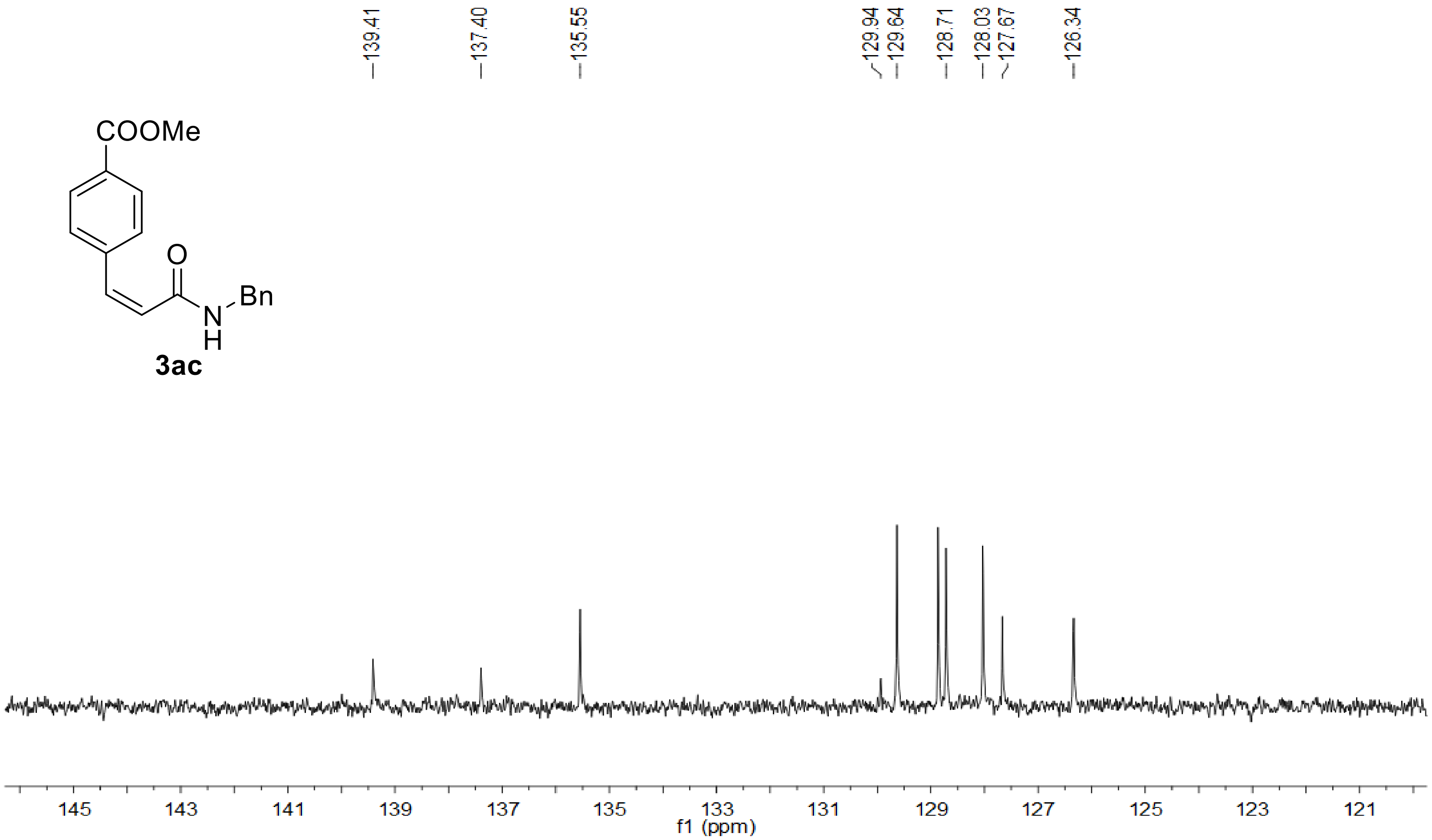

Supplementary Figure 94. Local magnification ^{13}C NMR spectrum of methyl (*Z*)-4-(3-(benzylamino)-3-oxoprop-1-en-1-yl)benzoate (**3ac**) in $CDCl_3$ (100 MHz) at 23℃.

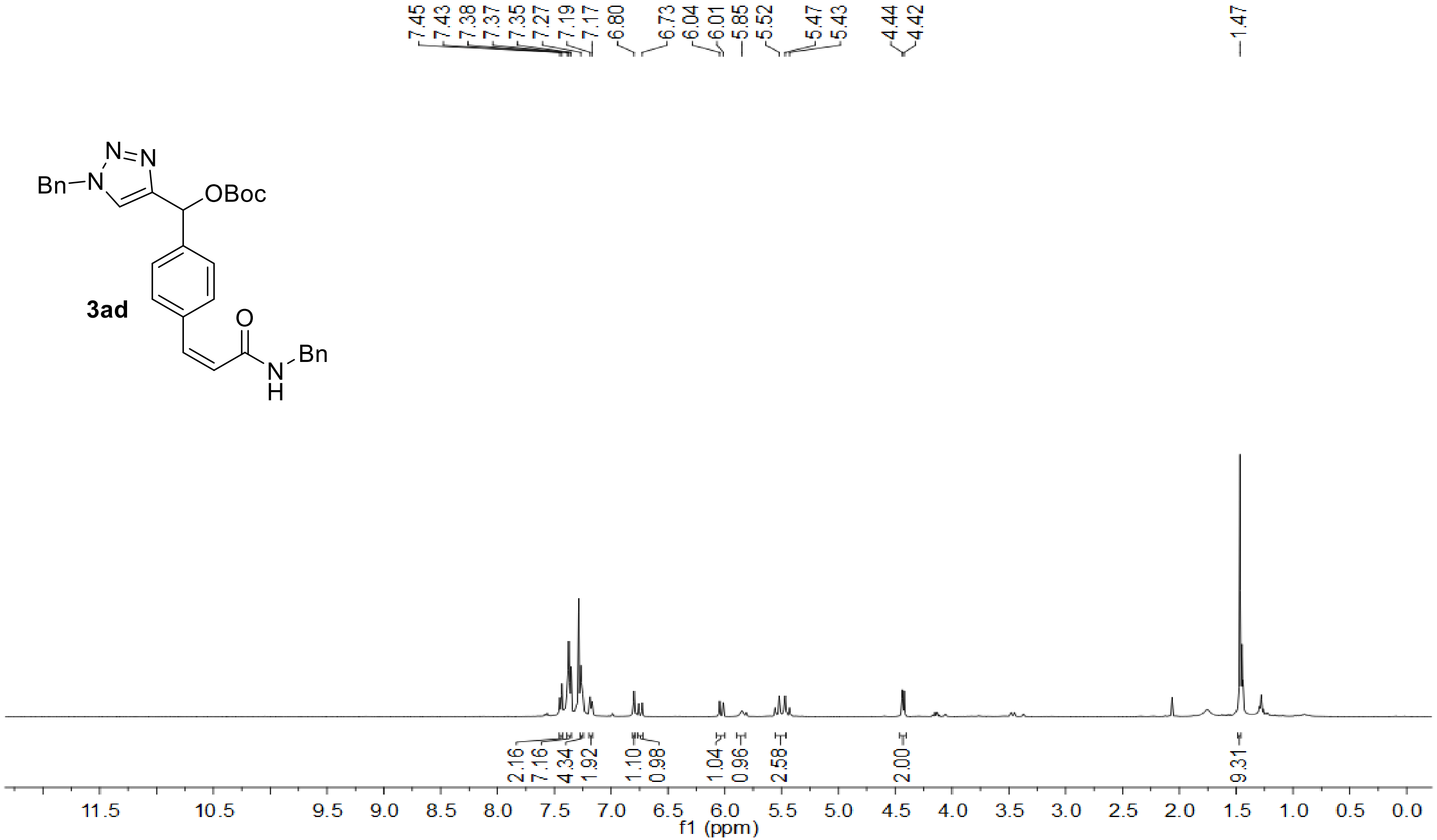

Supplementary Figure 95. ^{1}H NMR spectrum of (*Z*)-(1-benzyl-1*H*-1,2,3-triazol-4-yl)(4-(3-(benzylamino)-3-oxoprop-1-en-1-yl)phenyl)methyl *tert*-butyl carbonate (**3ad**) in $CDCl_3$ (400 MHz) at 23℃.

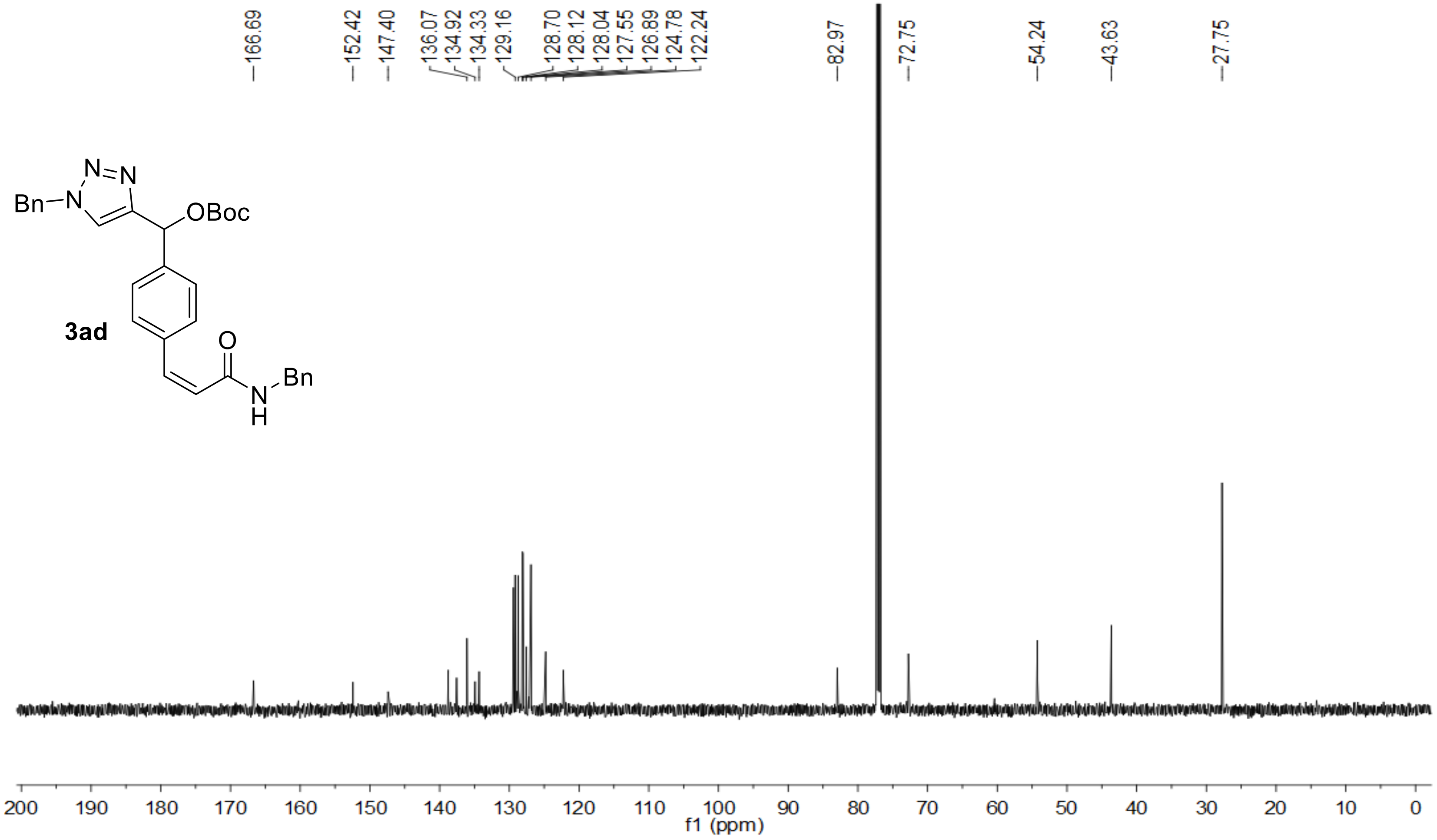

Supplementary Figure 96. ^{13}C NMR spectrum of (*Z*)-(1-benzyl-1*H*-1,2,3-triazol-4-yl)(4-(3-(benzylamino)-3-oxoprop-1-en-1-yl)phenyl)methyl *tert*-butyl carbonate (**3ad**) in $CDCl_3$ (100 MHz) at 23℃.

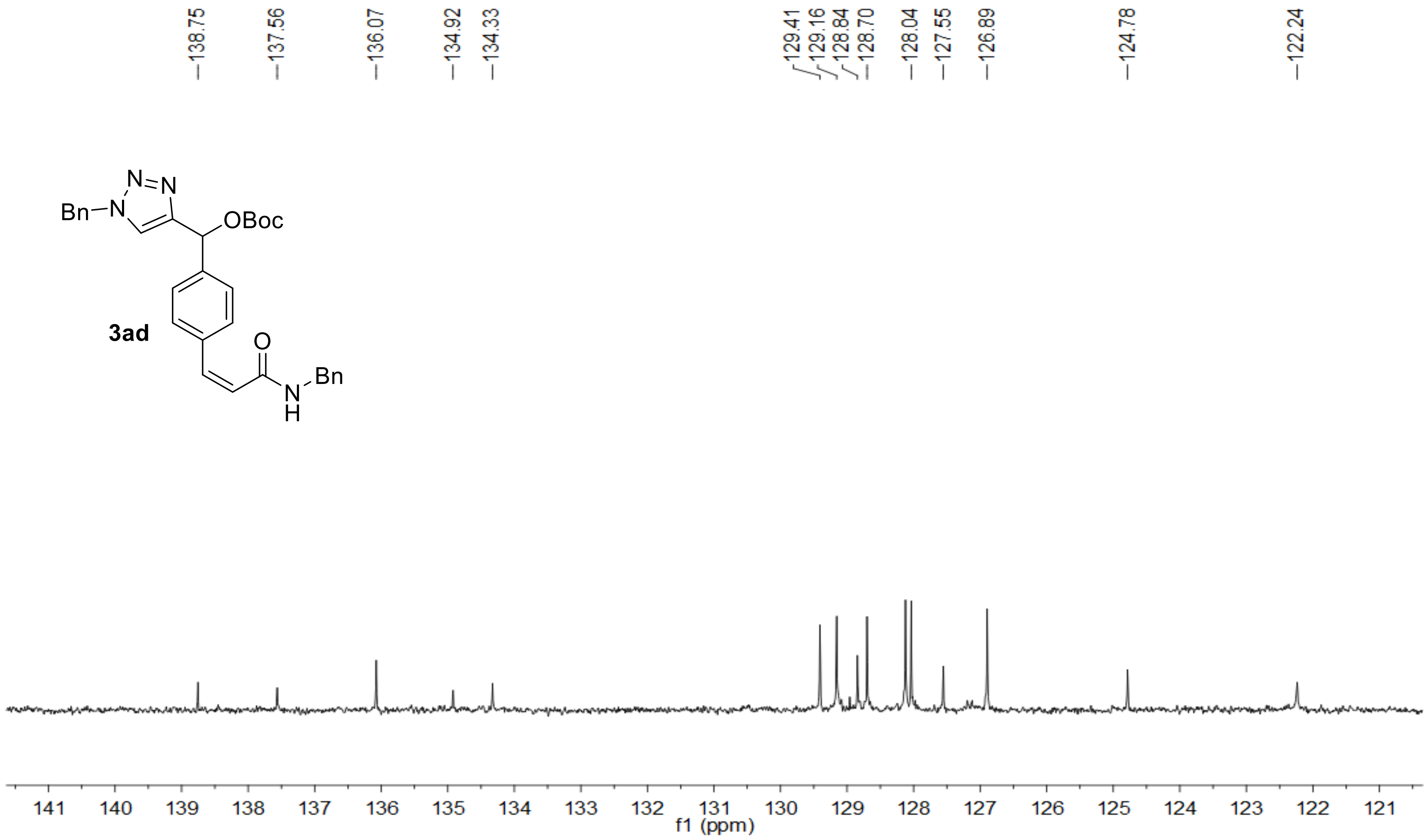

Supplementary Figure 97. Local magnification ^{13}C NMR spectrum of (*Z*)-(1-benzyl-1*H*-1,2,3-triazol-4-yl)(4-(3-(benzylamino)-3-oxoprop-1-en-1-yl)phenyl)methyl *tert*-butyl carbonate (**3ad**) in $CDCl_3$ (100 MHz) at 23℃.

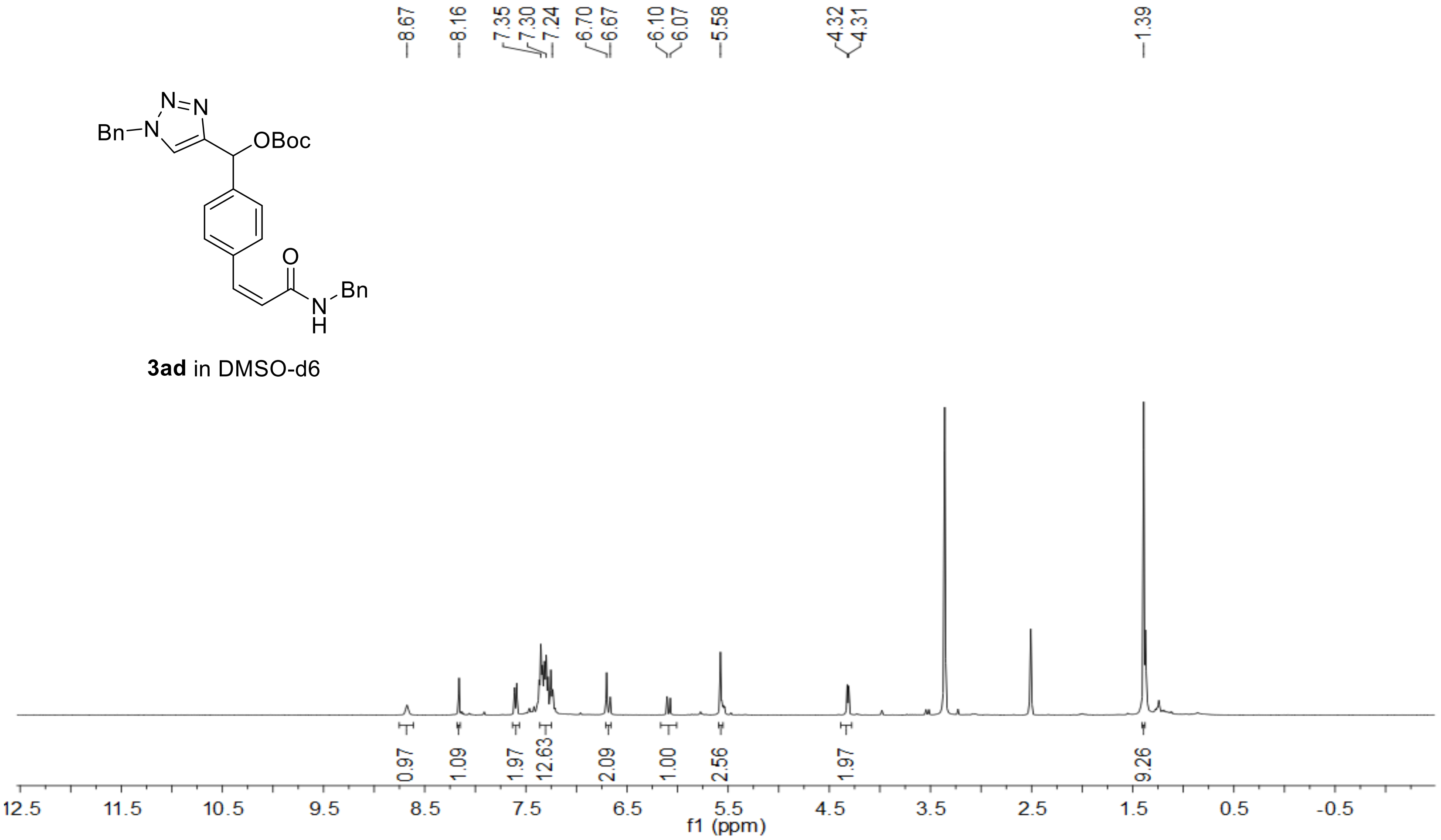

Supplementary Figure 98. ^{1}H NMR spectrum of (*Z*)-(1-benzyl-1*H*-1,2,3-triazol-4-yl)(4-(3-(benzylamino)-3-oxoprop-1-en-1-yl)phenyl)methyl *tert*-butyl carbonate (**3ad**) in DMSO-d6 (400 MHz) at 23℃.

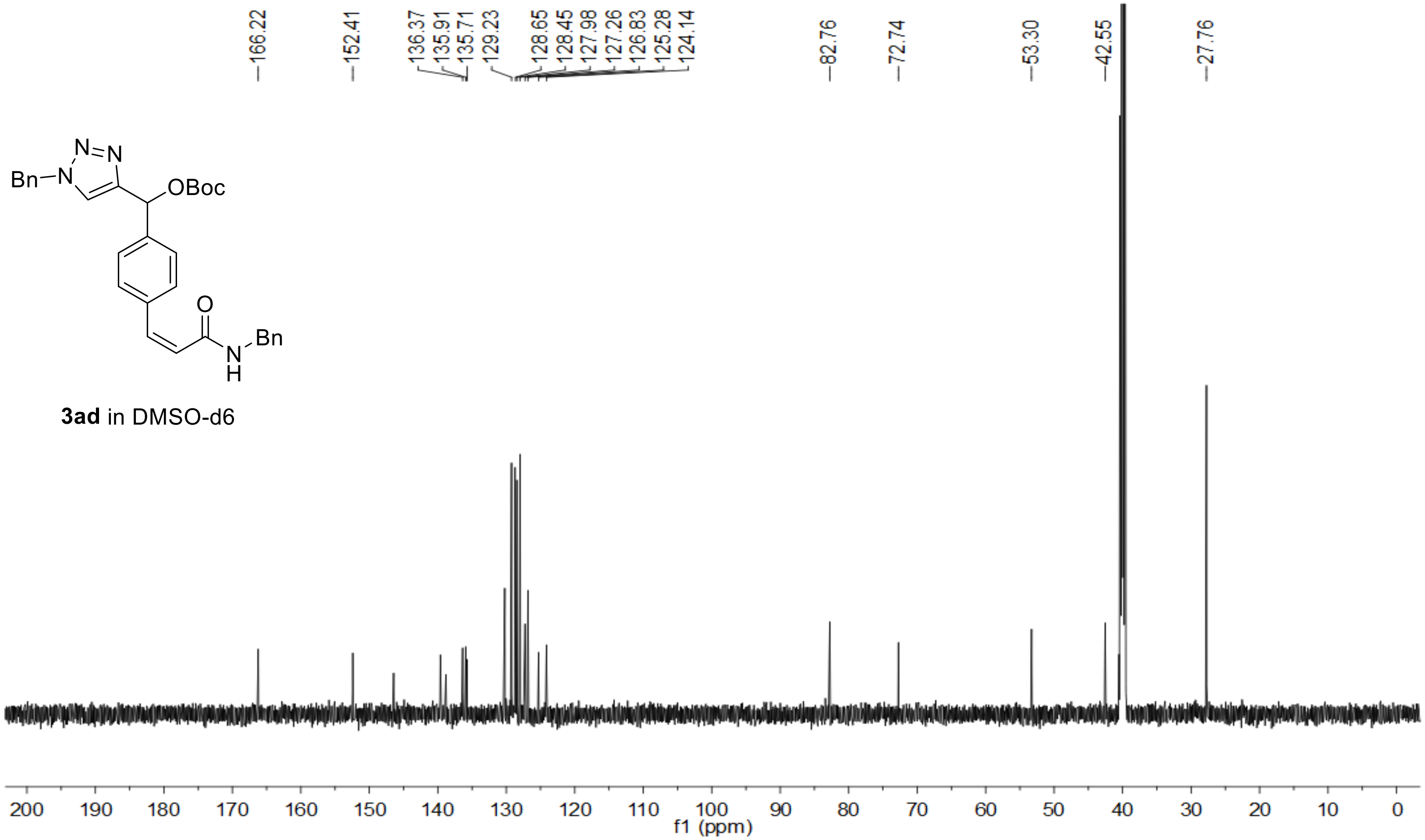

Supplementary Figure 99. ^{13}C NMR spectrum of (*Z*)-(1-benzyl-1*H*-1,2,3-triazol-4-yl)(4-(3-(benzylamino)-3-oxoprop-1-en-1-yl)phenyl)methyl *tert*-butyl carbonate (**3ad**) in DMSO-d6 (100 MHz) at 23℃.

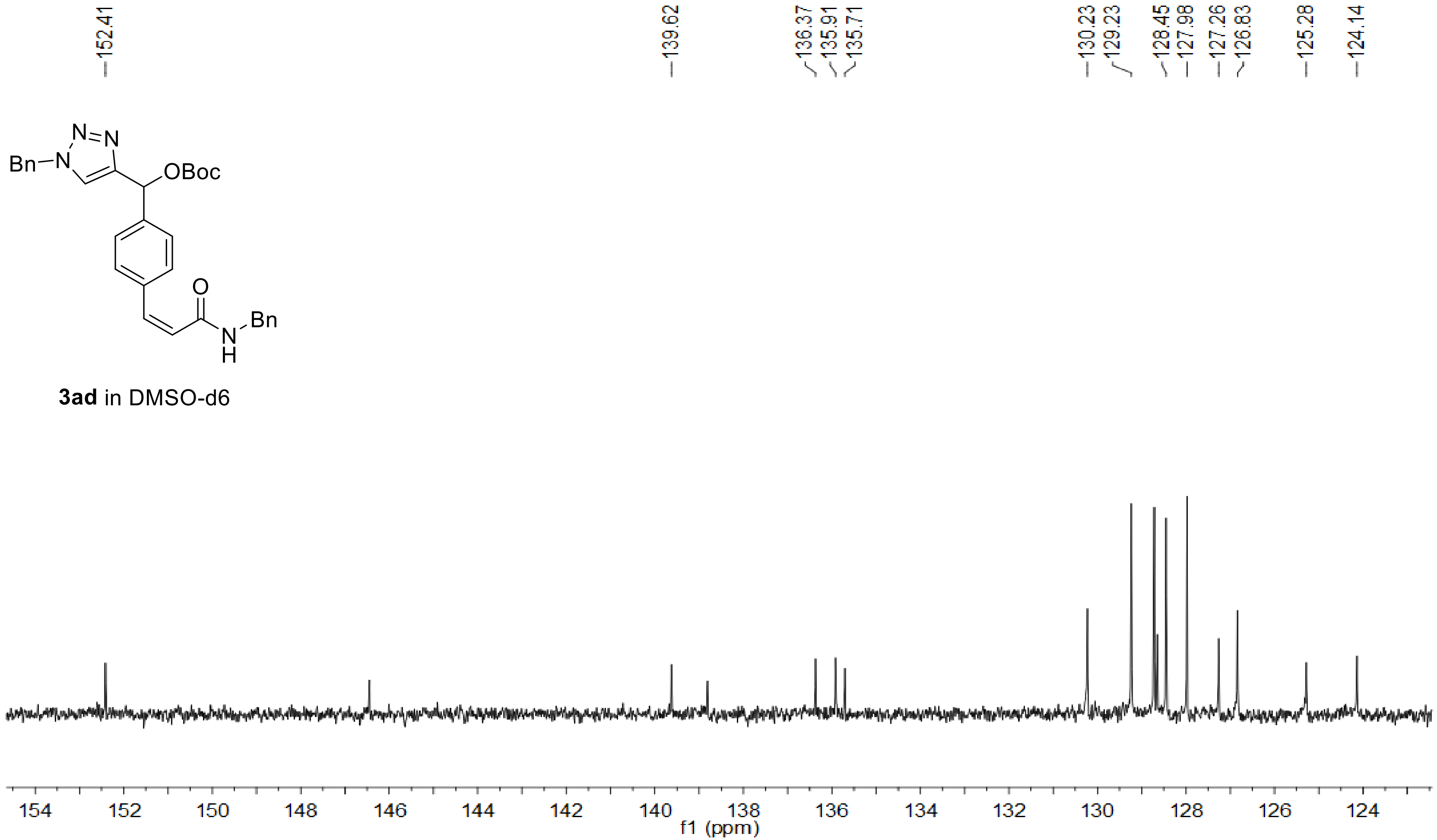

Supplementary Figure 100. Local magnification ^{13}C NMR spectrum of (*Z*)-(1-benzyl-1*H*-1,2,3-triazol-4-yl)(4-(3-(benzylamino)-3-oxoprop-1-en-1-yl)phenyl)methyl *tert*-butyl carbonate (**3ad**) in DMSO-d6 (100 MHz) at 23°C.

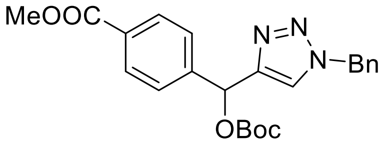

4a

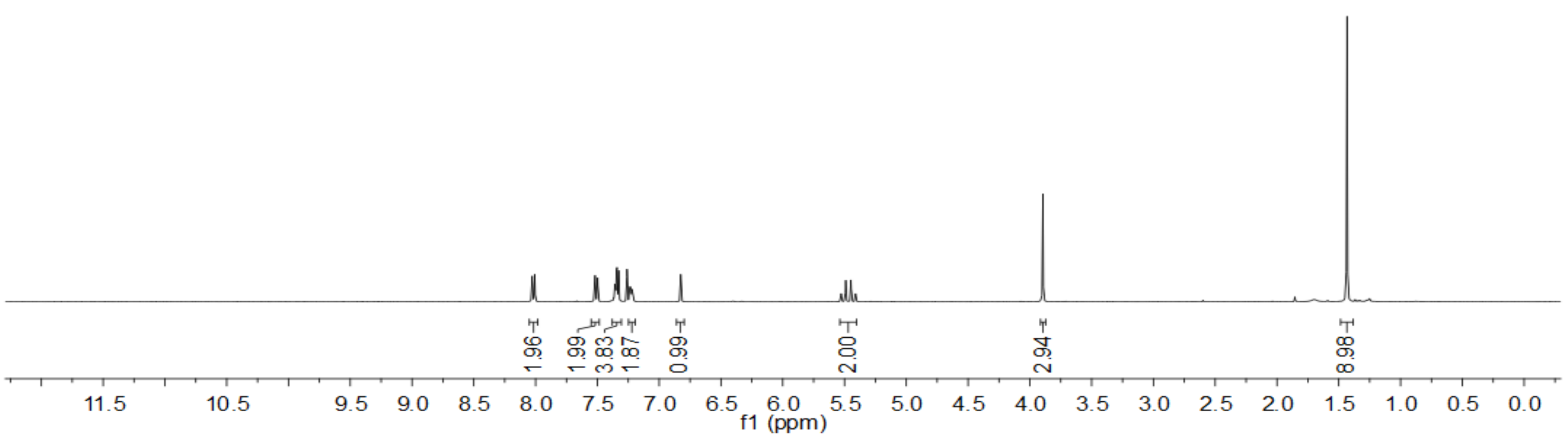

Supplementary Figure 101. ^{1}H NMR spectrum of methyl 4-((1-benzyl-1*H*-1,2,3-triazol-4-yl)((*tert*-butoxycarbonyl)oxy)methyl)benzoate (**4a**) in $CDCl_3$ (400 MHz) at 23℃.

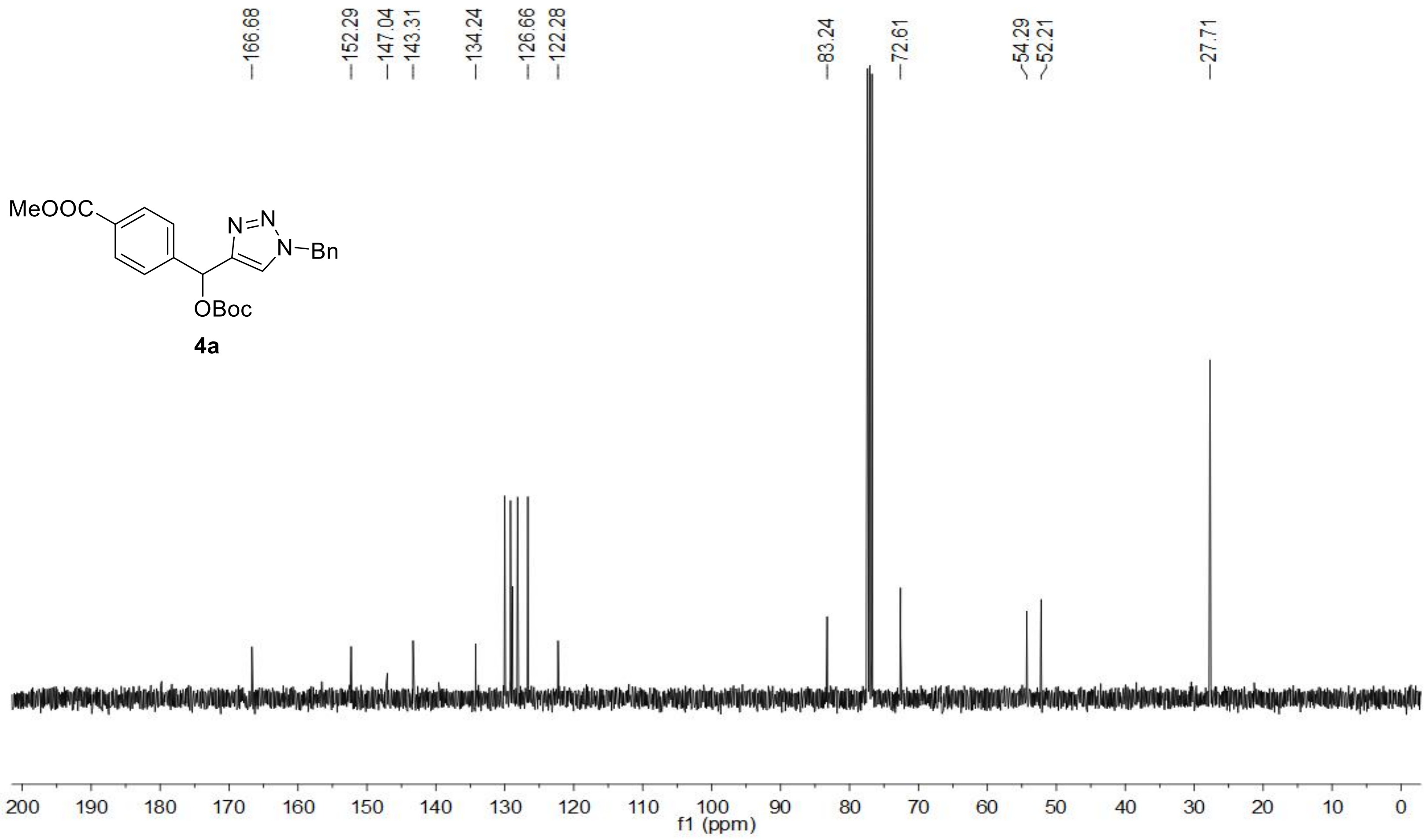

Supplementary Figure 102. ^{13}C NMR spectrum of methyl 4-((1-benzyl-1*H*-1,2,3-triazol-4-yl)((*tert*-butoxycarbonyl)oxy)methyl)benzoate (**4a**) in $CDCl_3$ (100 MHz) at 23℃.

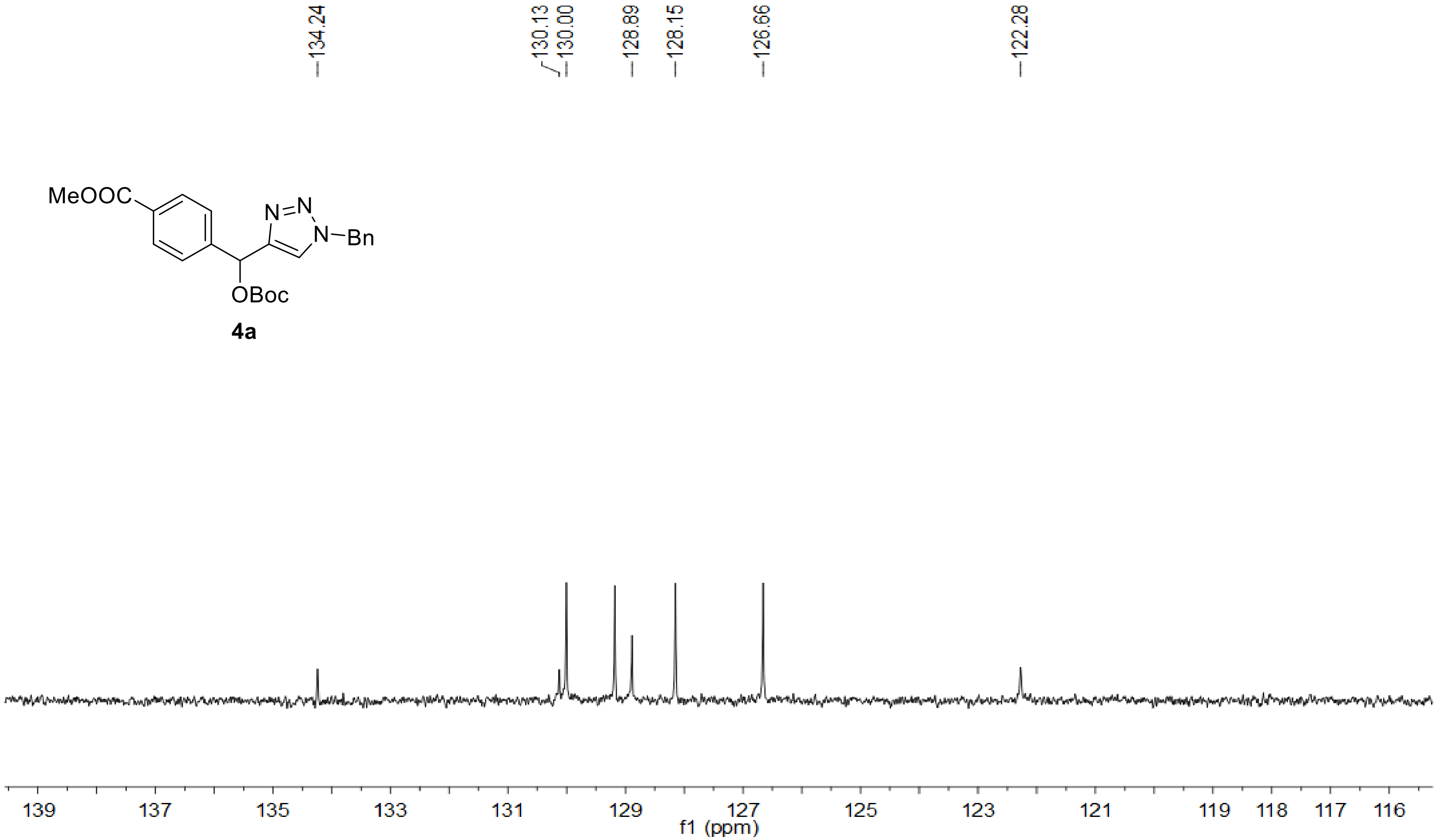

Supplementary Figure 103. Local magnification ^{13}C NMR spectrum of methyl 4-((1-benzyl-1*H*-1,2,3-triazol-4-yl)((*tert*-butoxycarbonyl)oxy)methyl)benzoate (**4a**) in $CDCl_3$ (100 MHz) at 23℃.

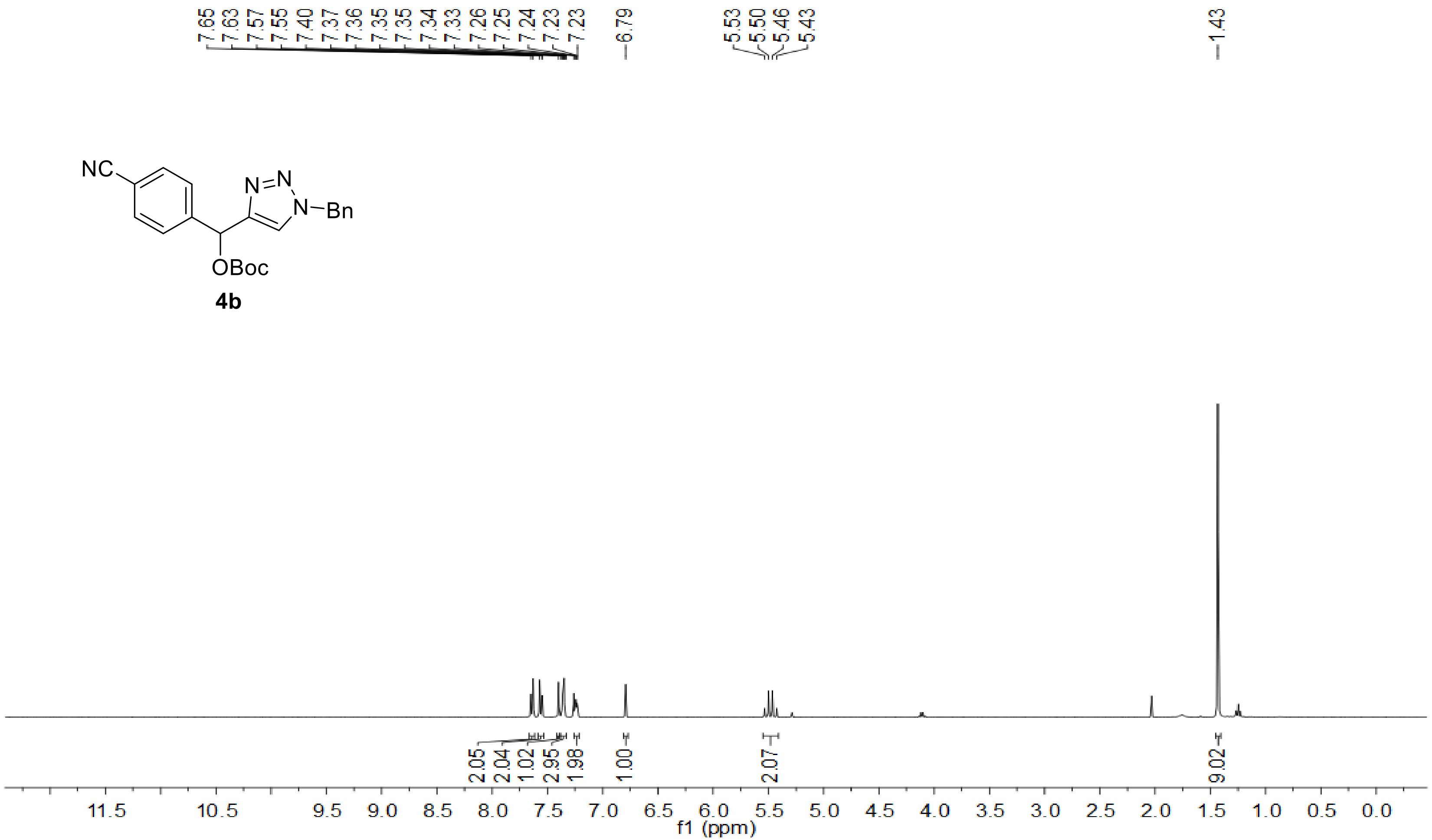

Supplementary Figure 104. ^{1}H NMR spectrum of (1-benzyl-1*H*-1,2,3-triazol-4-yl)(4-cyanophenyl)methyl *tert*-butyl carbonate (**4b**) in $CDCl_3$ (400 MHz) at 23℃.

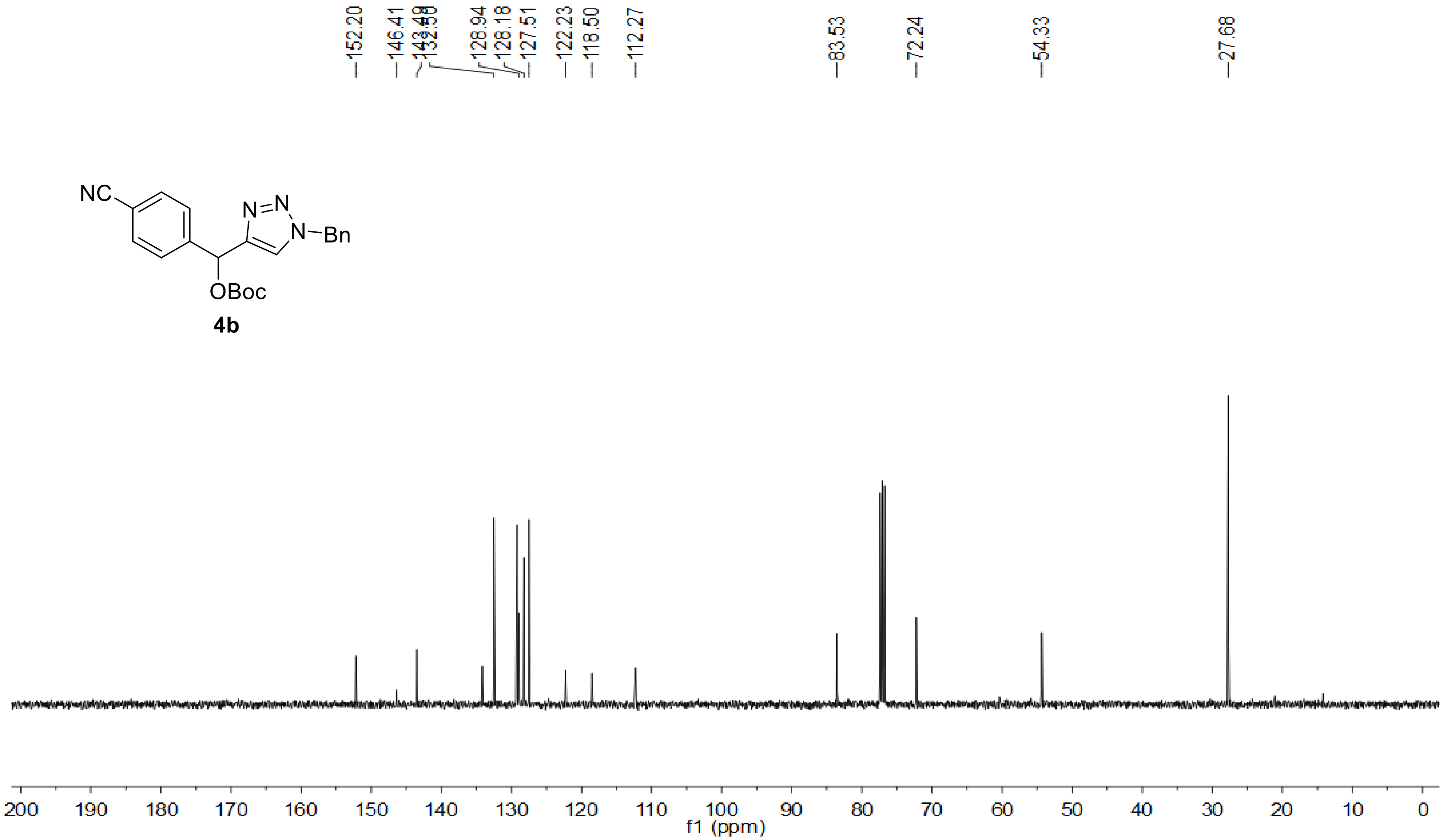

Supplementary Figure 105. ^{13}C NMR spectrum of (1-benzyl-1*H*-1,2,3-triazol-4-yl)(4-cyanophenyl)methyl *tert*-butyl carbonate (**4b**) in $CDCl_3$ (100 MHz) at 23℃.

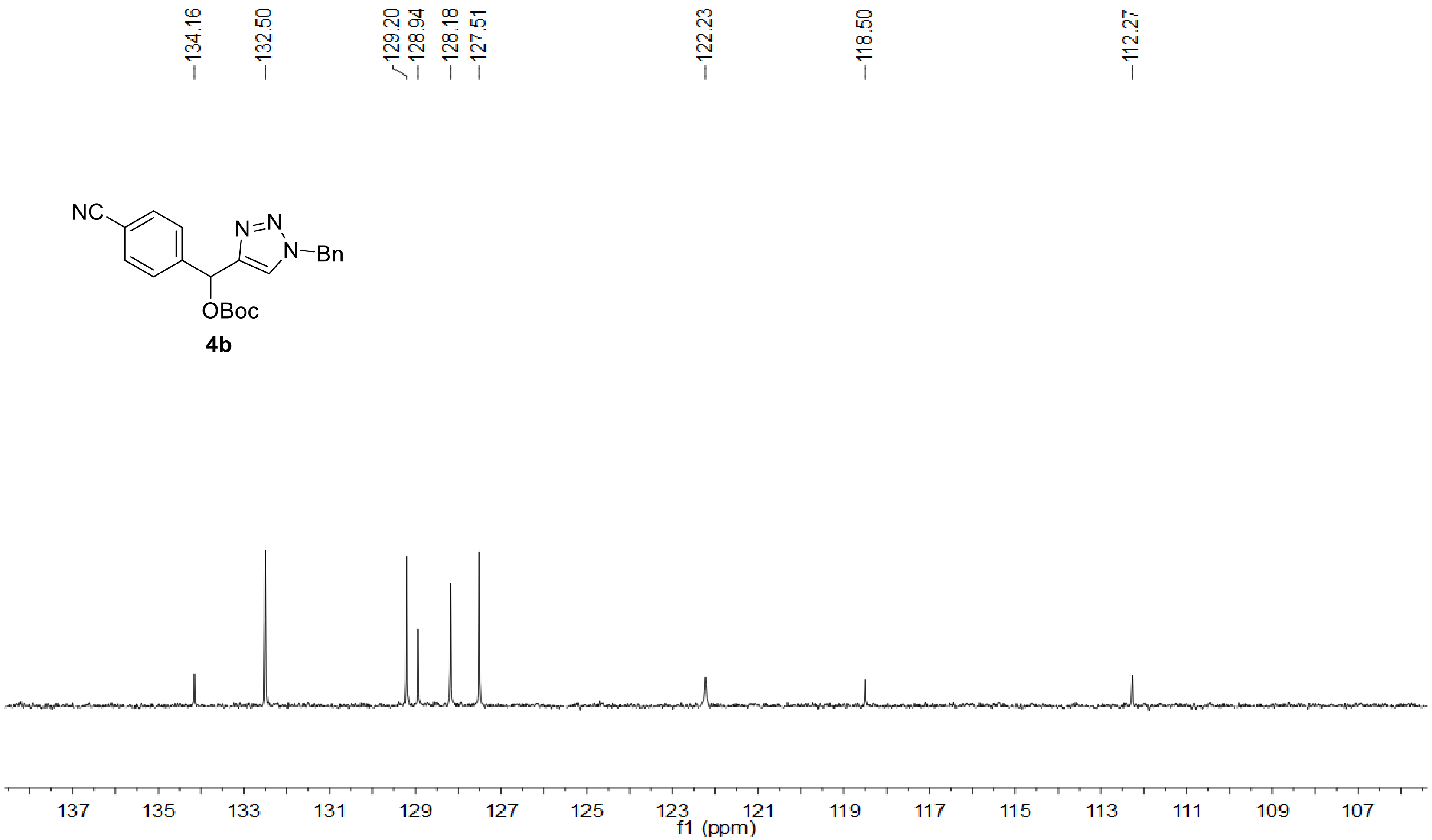

Supplementary Figure 106. Local magnification ^{13}C NMR spectrum of (1-benzyl-1*H*-1,2,3-triazol-4-yl)(4-cyanophenyl)methyl *tert*-butyl carbonate (**4b**) in $CDCl_3$ (100 MHz) at 23℃.

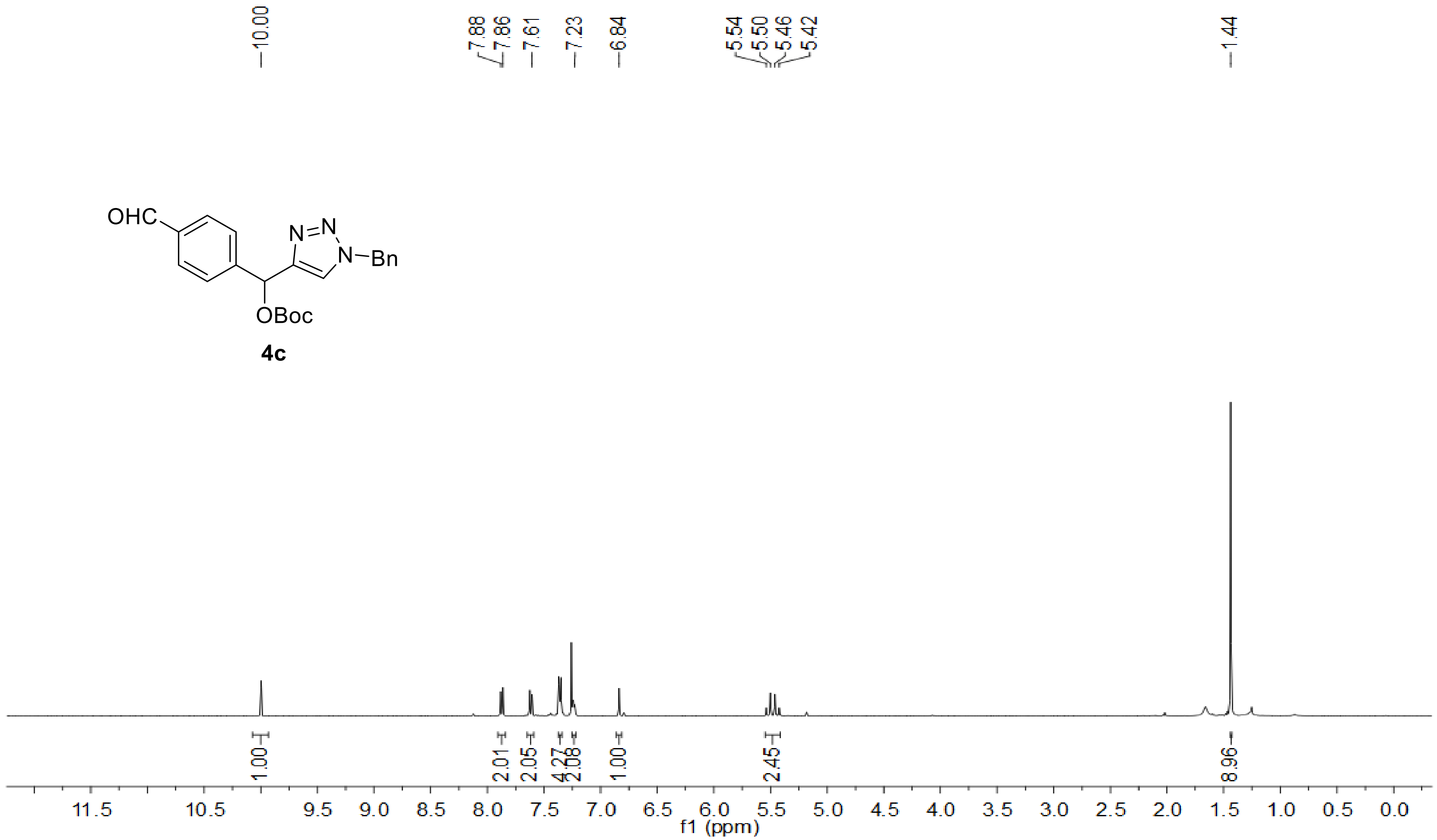

Supplementary Figure 107. ^{1}H NMR spectrum of (1-benzyl-1*H*-1,2,3-triazol-4-yl)(4-formylphenyl)methyl *tert*-butyl carbonate (**4c**) in $CDCl_3$ (400 MHz) at 23℃.

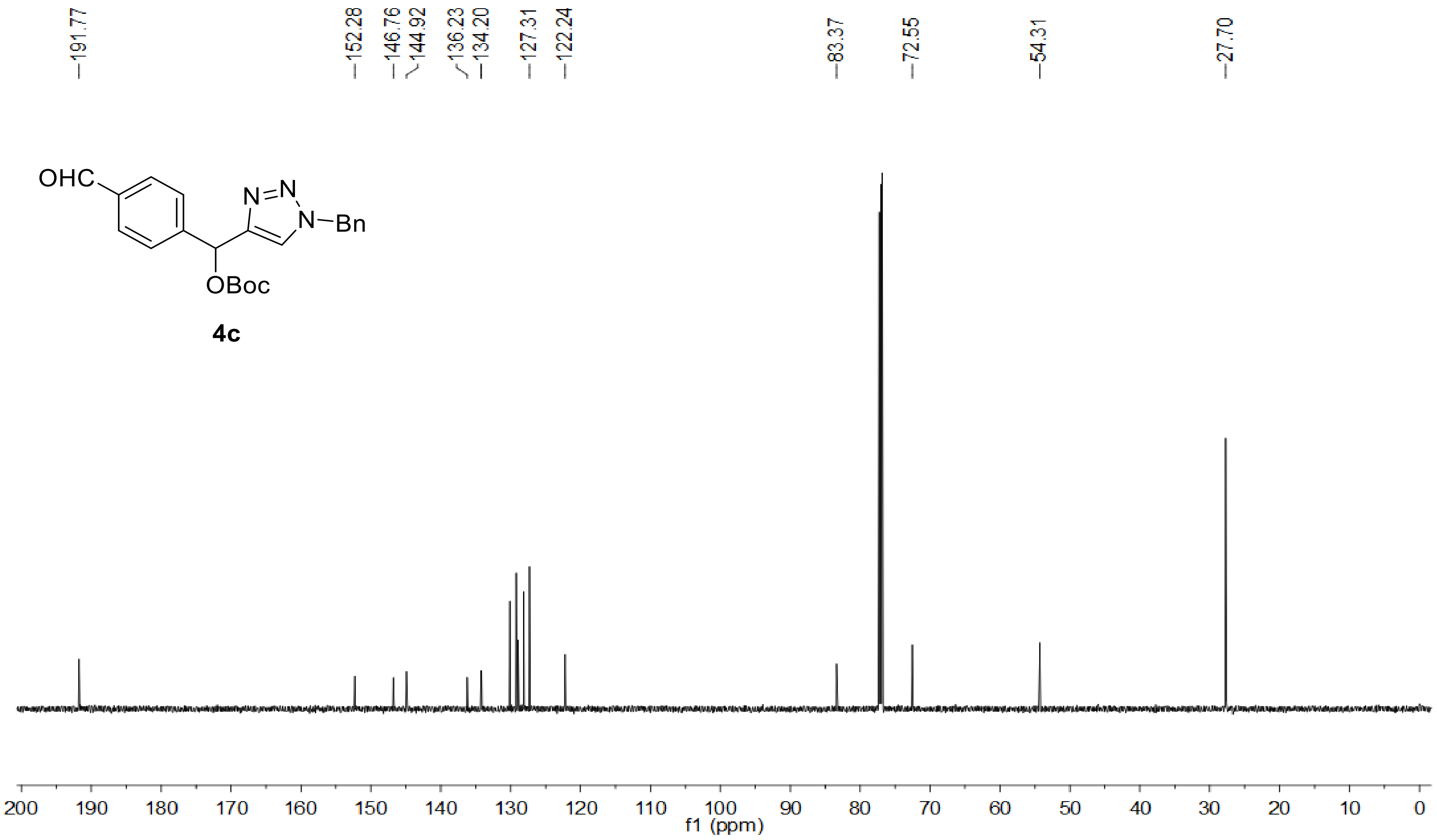

Supplementary Figure 108. ^{13}C NMR spectrum of (1-benzyl-1*H*-1,2,3-triazol-4-yl)(4-formylphenyl)methyl *tert*-butyl carbonate (**4c**) in $CDCl_3$ (100 MHz) at 23℃.

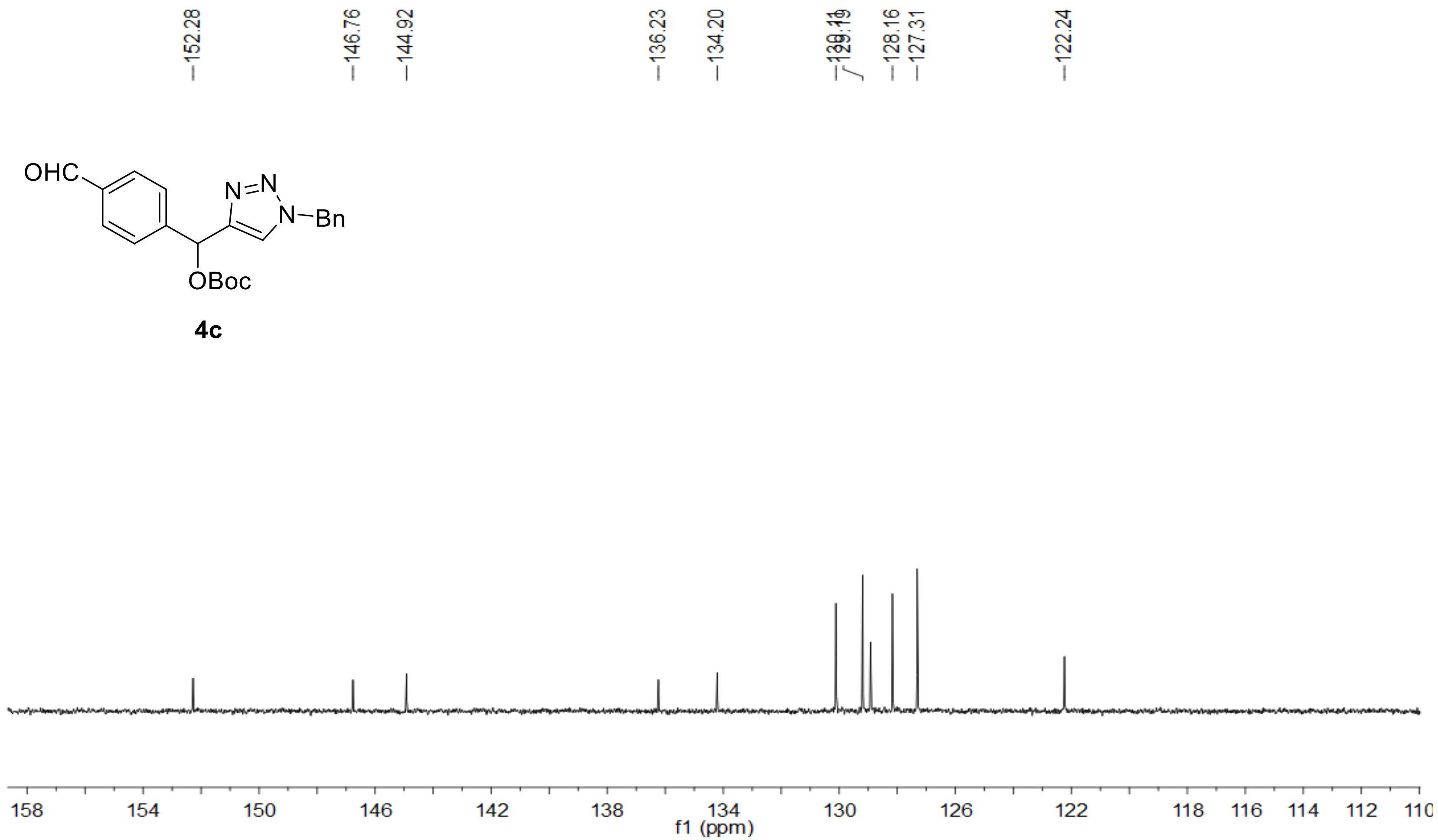

Supplementary Figure 109. Local magnification ^{13}C NMR spectrum of (1-benzyl-1*H*-1,2,3-triazol-4-yl)(4-formylphenyl)methyl *tert*-butyl carbonate (**4c**) in $CDCl_3$ (100 MHz) at 23℃.

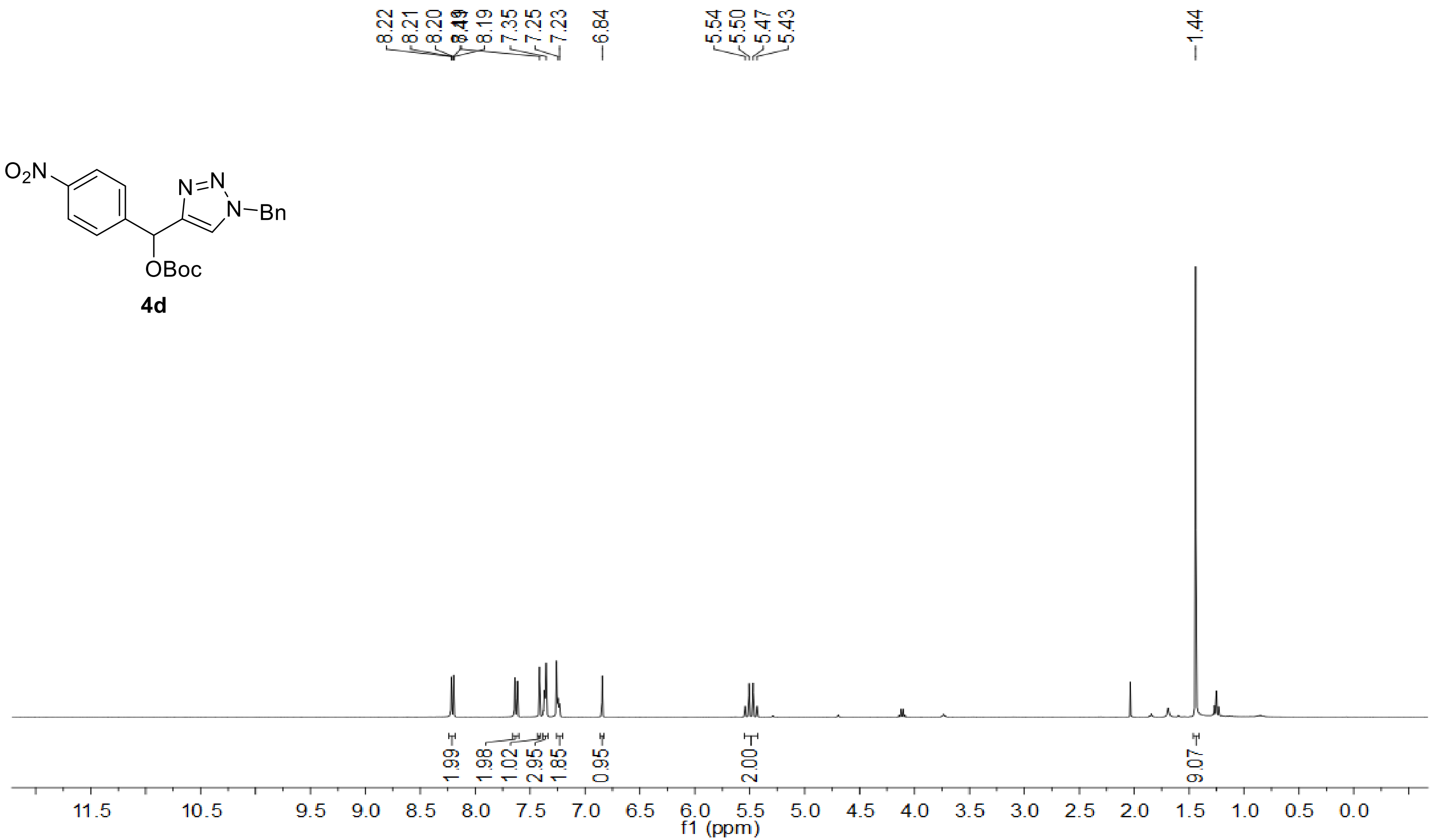

Supplementary Figure 110. ^{1}H NMR spectrum of (1-benzyl-1*H*-1,2,3-triazol-4-yl)(4-nitrophenyl)methyl *tert*-butyl carbonate (**4d**) in $CDCl_3$ (400 MHz) at 23℃.

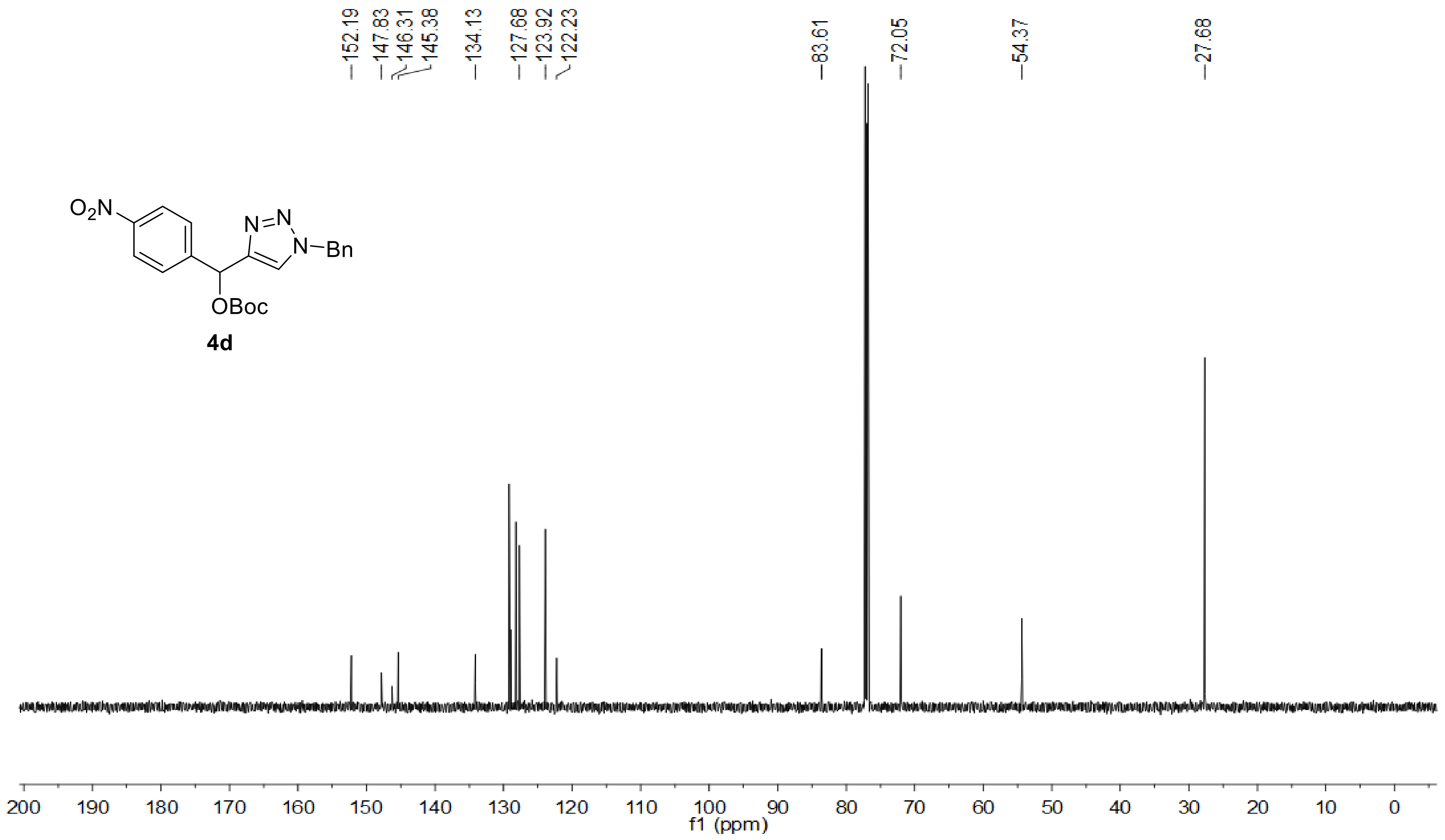

4d

Supplementary Figure 111. ^{13}C NMR spectrum of (1-benzyl-1*H*-1,2,3-triazol-4-yl)(4-nitrophenyl)methyl *tert*-butyl carbonate (**4d**) in $CDCl_3$ (100 MHz) at 23℃.

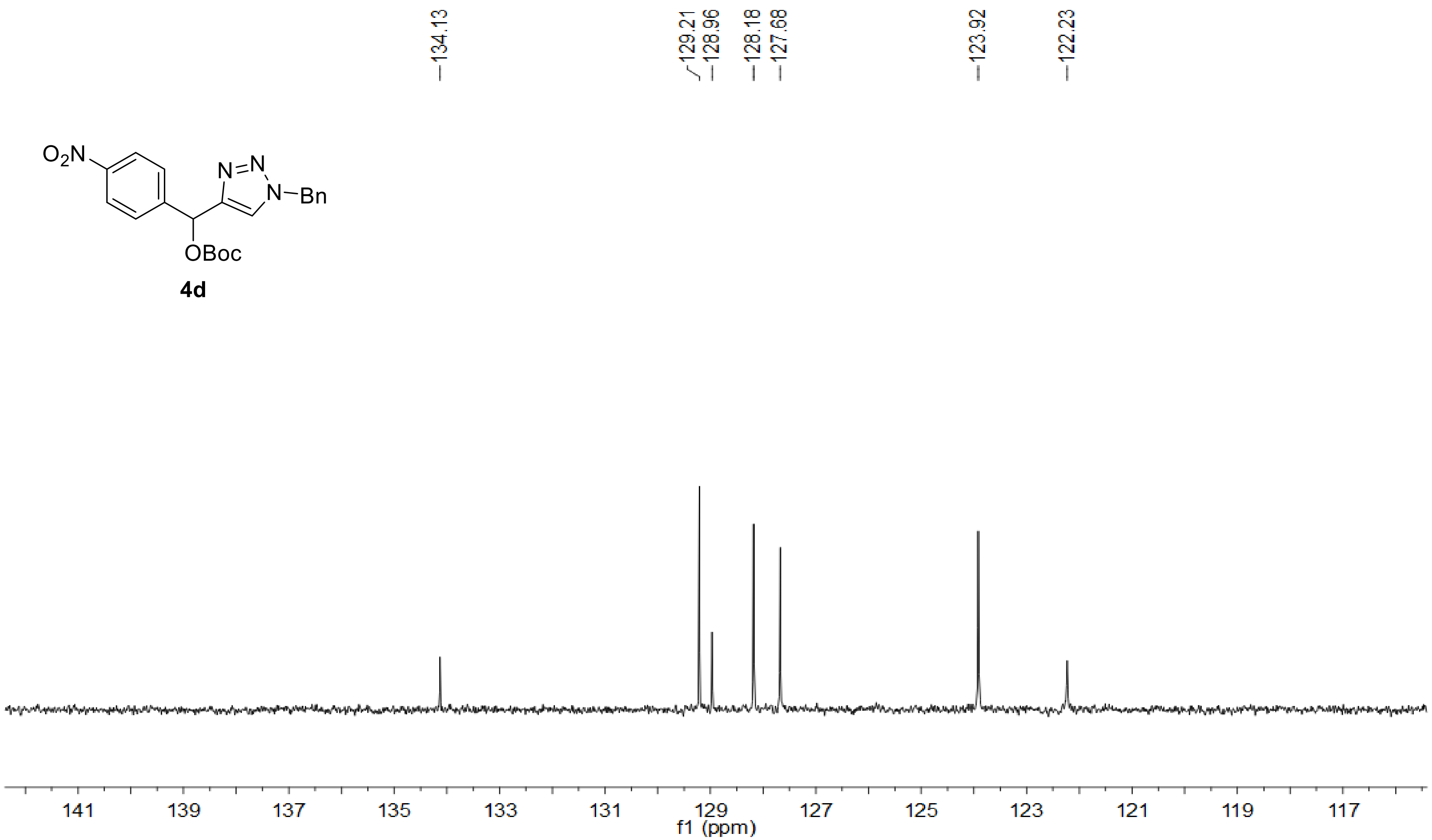

Supplementary Figure 112. Local magnification ^{13}C NMR spectrum of (1-benzyl-1*H*-1,2,3-triazol-4-yl)(4-nitrophenyl)methyl *tert*-butyl carbonate (**4d**) in $CDCl_3$ (100 MHz) at 23℃.

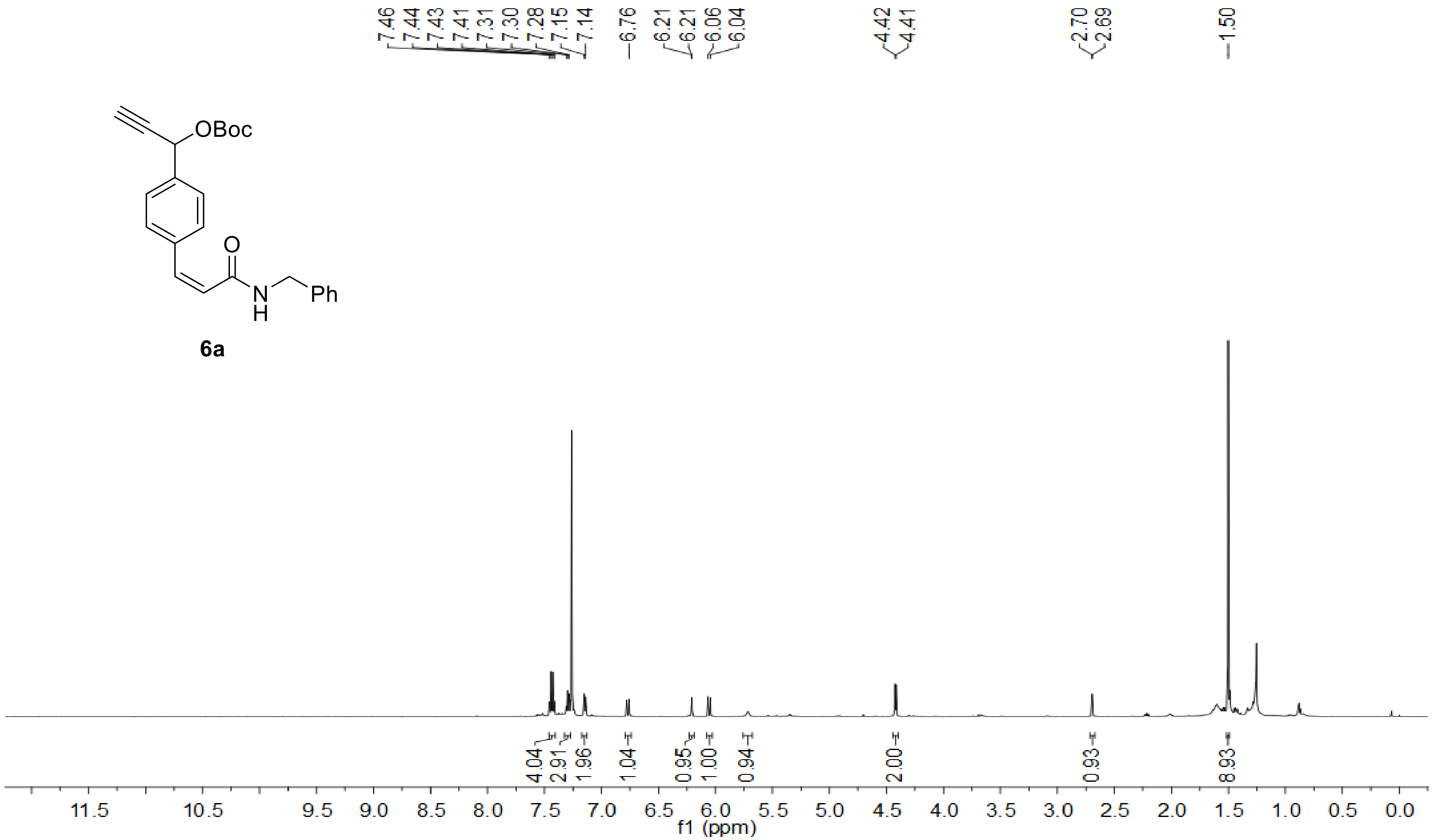

Supplementary Figure 113. ^{1}H NMR spectrum of (*Z*)-1-(4-(3-(benzylamino)-3-oxoprop-1-en-1-yl)phenyl)prop-2-yn-1-yl *tert*-butyl carbonate (**6a**) in $CDCl_3$ (600 MHz) at 23℃.

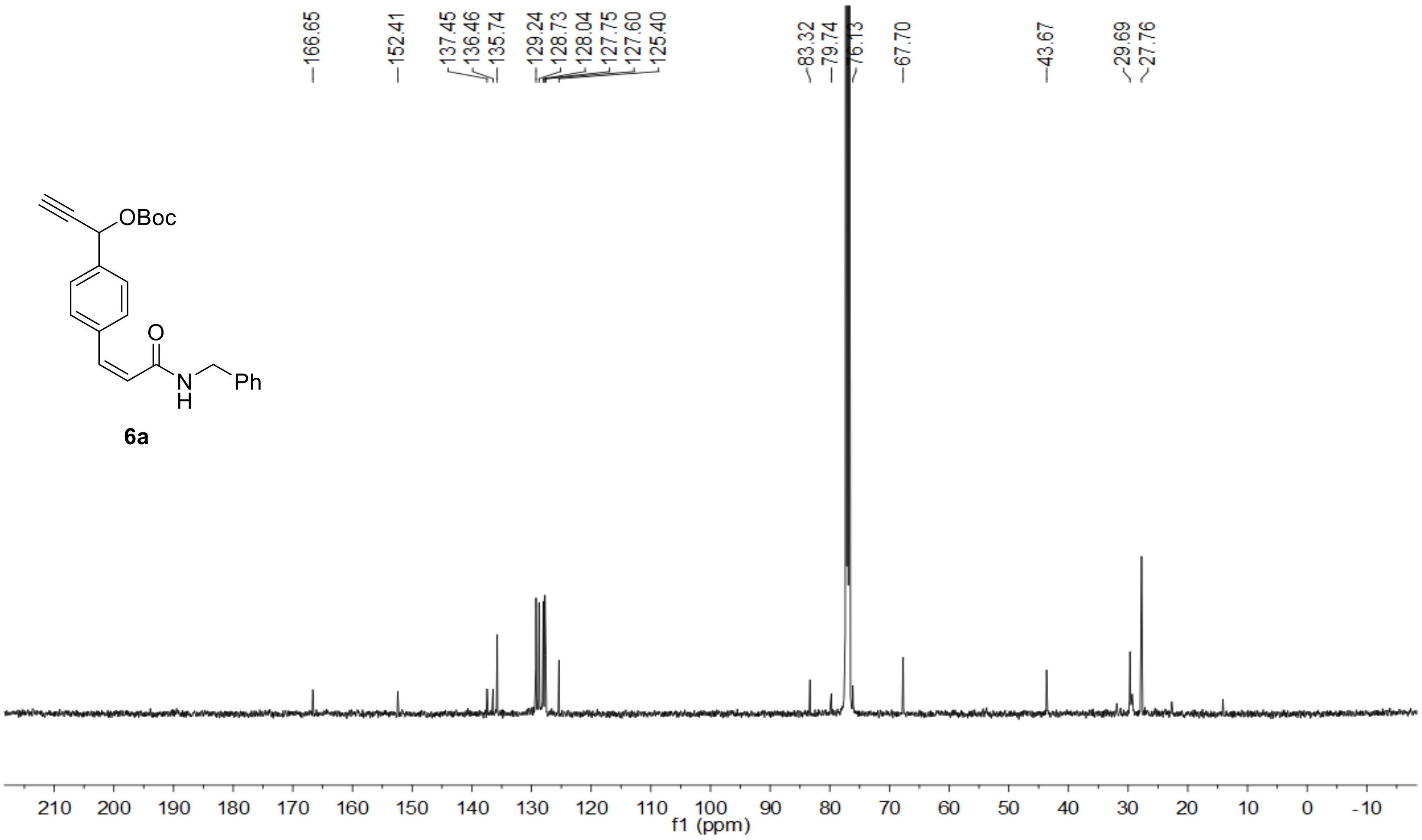

6a

Supplementary Figure 114. ^{13}C NMR spectrum of (*Z*)-1-(4-(3-(benzylamino)-3-oxoprop-1-en-1-yl)phenyl)prop-2-yn-1-yl *tert*-butyl carbonate (**6a**) in $CDCl_3$ (100 MHz) at 23℃.

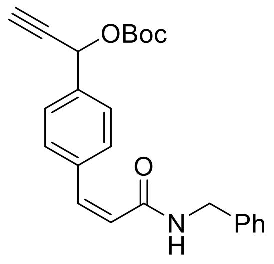

6a

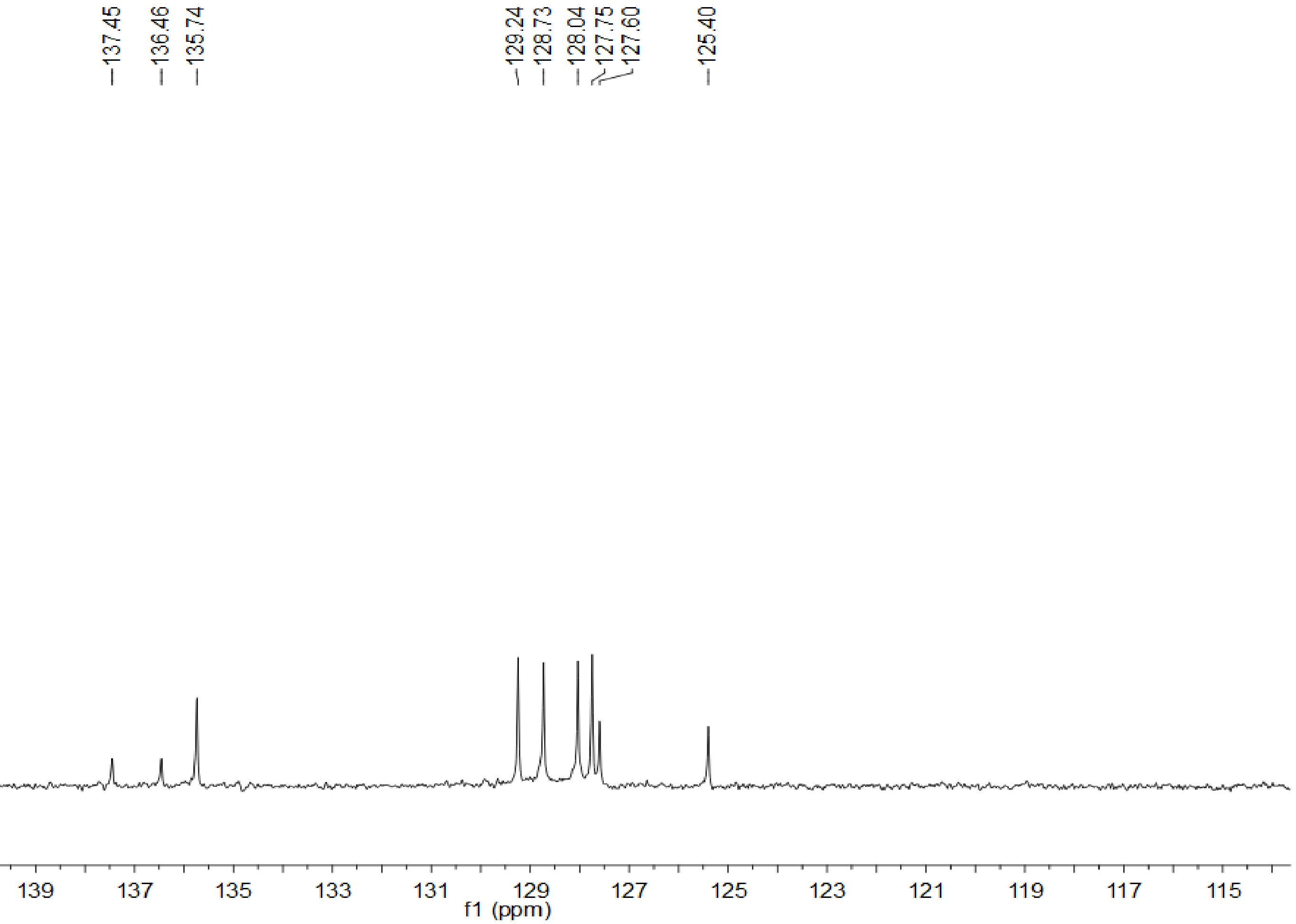

Supplementary Figure 115. Local magnification ^{13}C NMR spectrum of (*Z*)-1-(4-(3-(benzylamino)-3-oxoprop-1-en-1-yl)phenyl)prop-2-yn-1-yl *tert*-butyl carbonate (**6a**) in $CDCl_3$ (100 MHz) at 23℃.

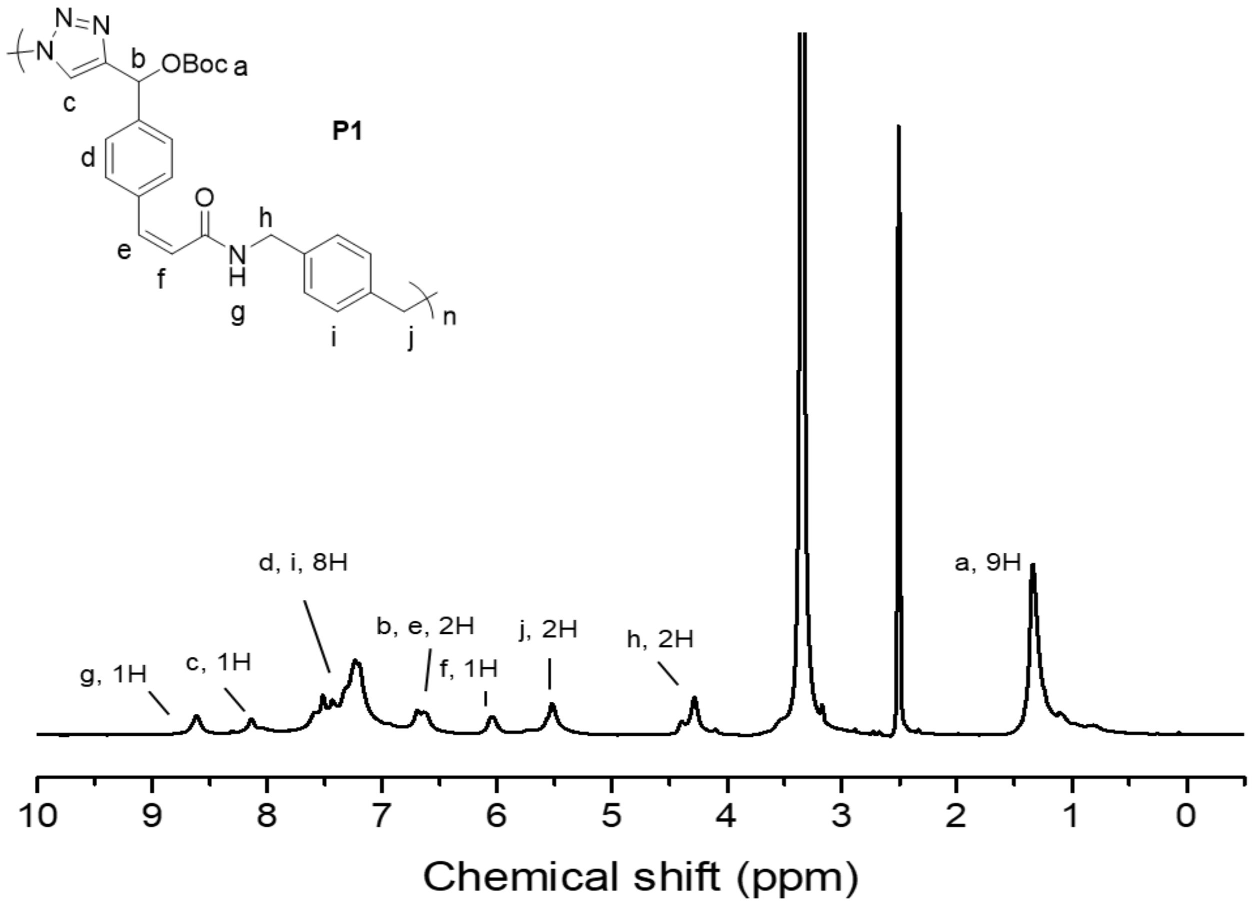

Supplementary Figure 116. ^{1}H NMR spectrum of **P1** in DMSO-d6 (400 MHz) at 23°C.

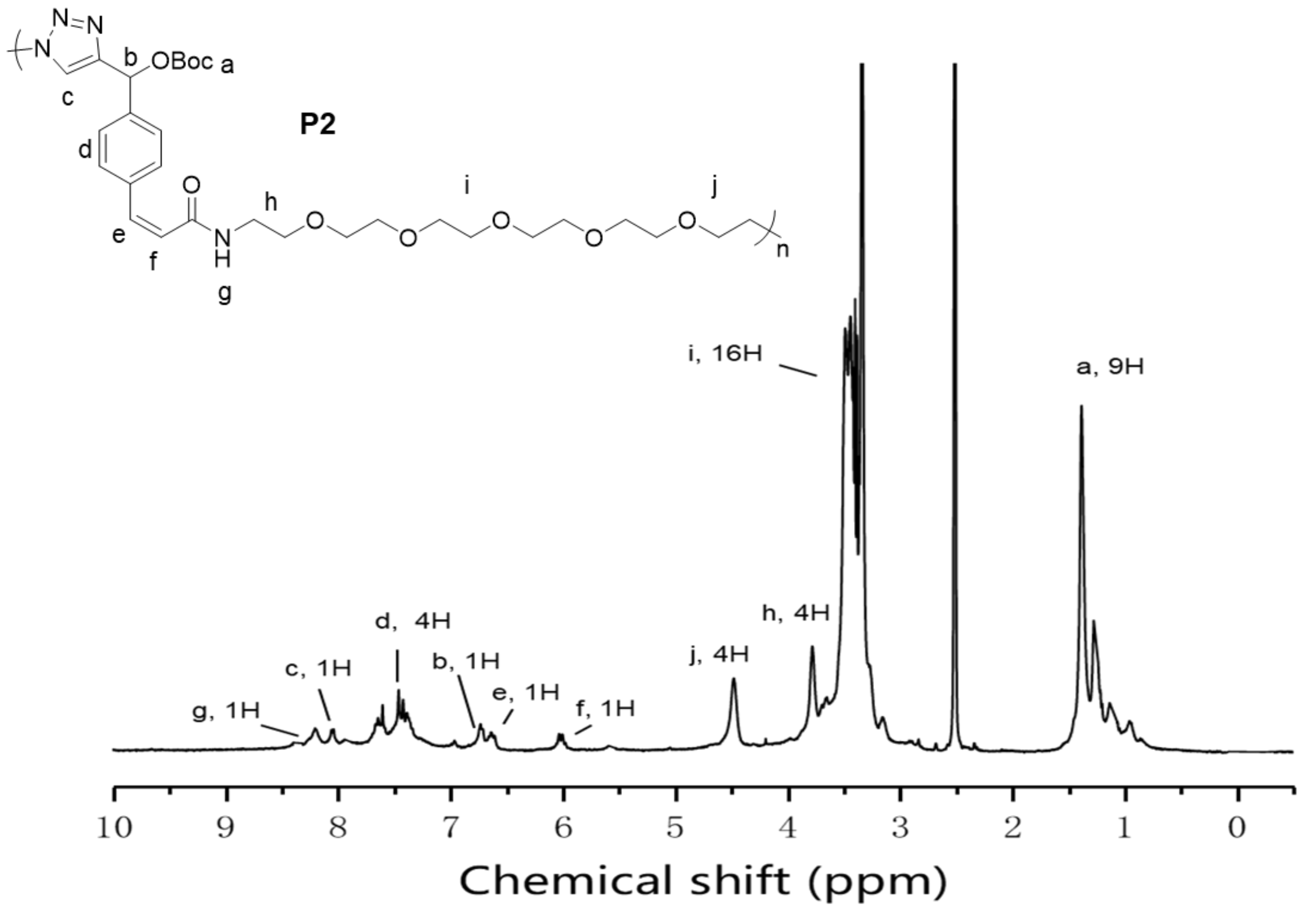

Supplementary Figure 117. ^{1}H NMR spectrum of **P2** in DMSO-d6 (400 MHz) at 23℃.

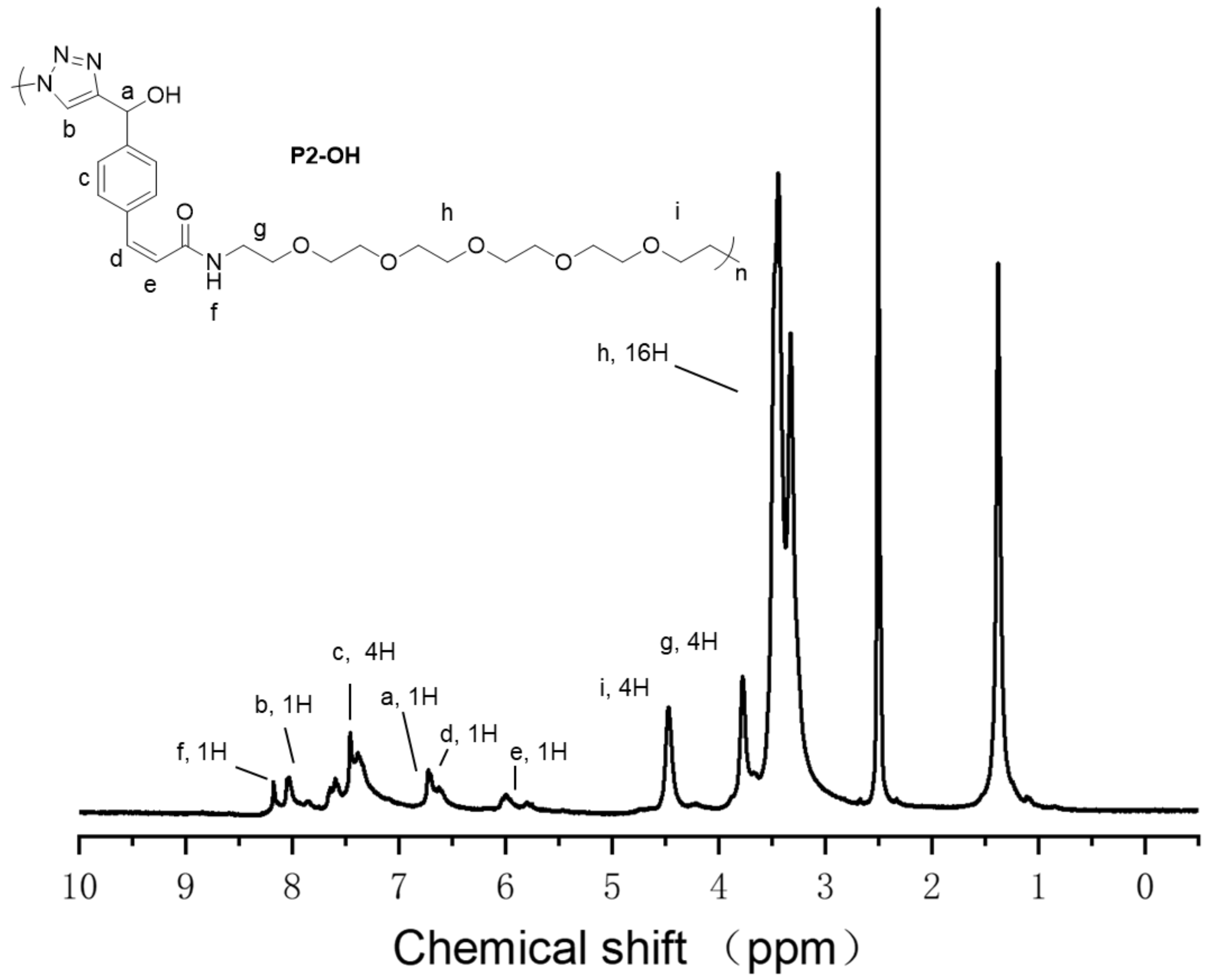

Supplementary Figure 118. ^{1}H NMR spectrum of **P2-OH** in DMSO-d6 (400 MHz) at 23℃.

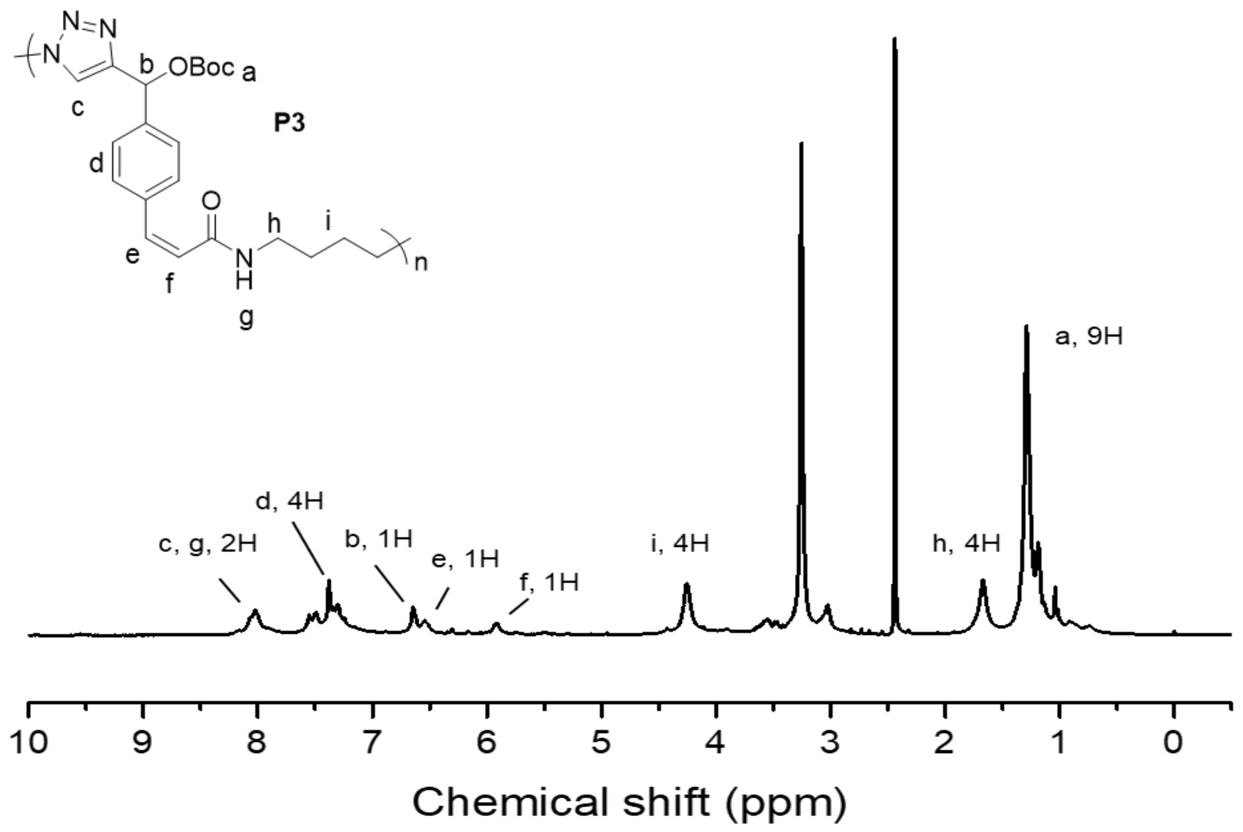

Supplementary Figure 119. ^{1}H NMR spectrum of **P3** in DMSO-d6 (600 MHz) at 23℃.

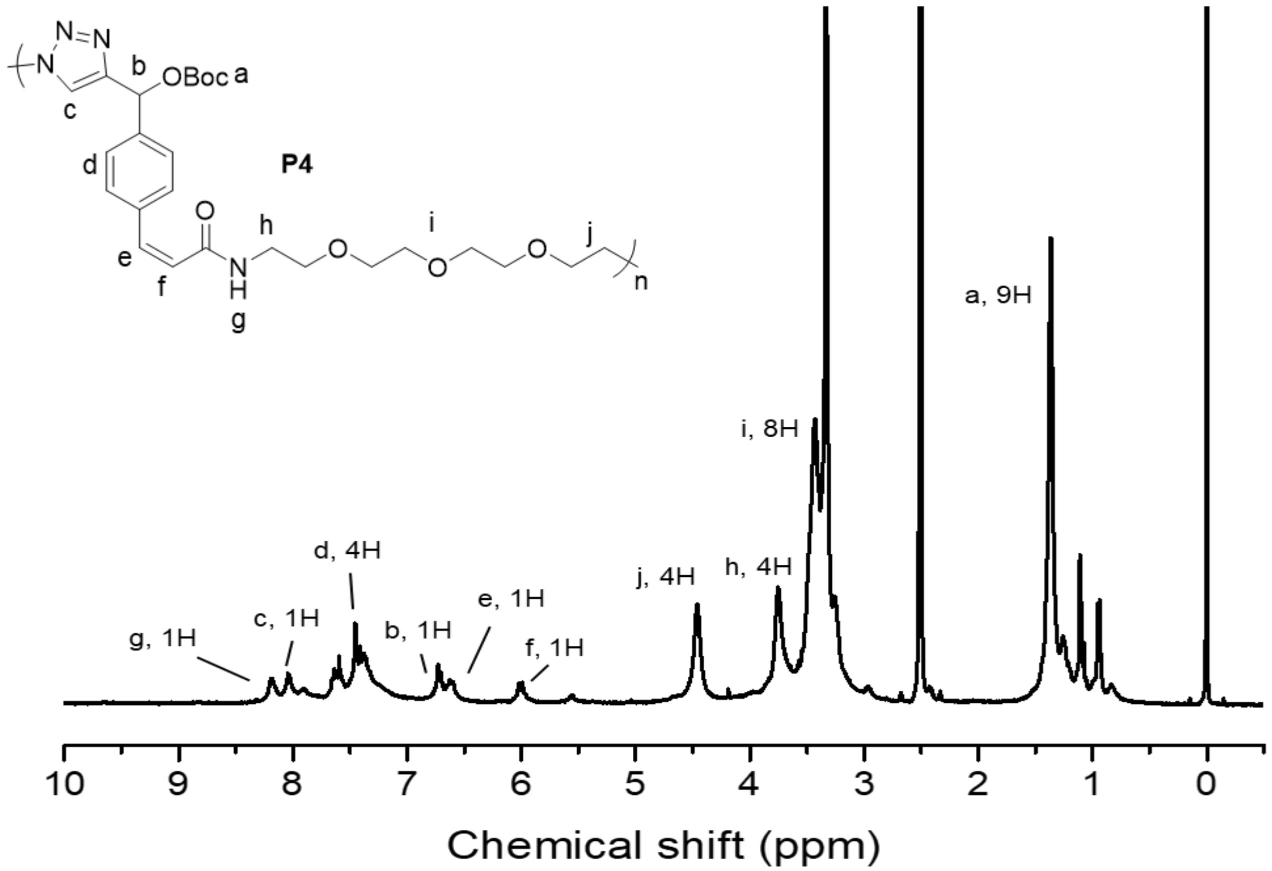

Supplementary Figure 120. ^{1}H NMR spectrum of **P4** in DMSO-d6 (400 MHz) at 23℃.

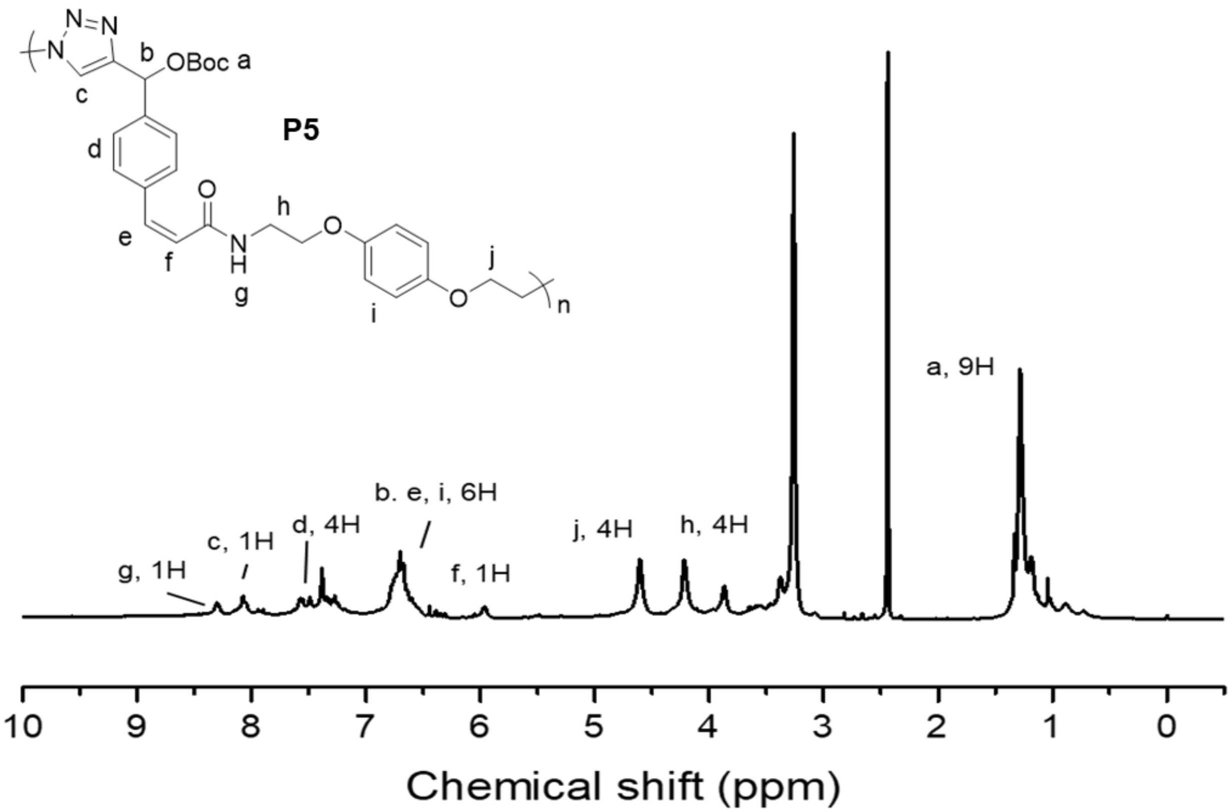

Supplementary Figure 121. ^{1}H NMR spectrum of **P5** in DMSO-d6 (600 MHz) at 23°C.

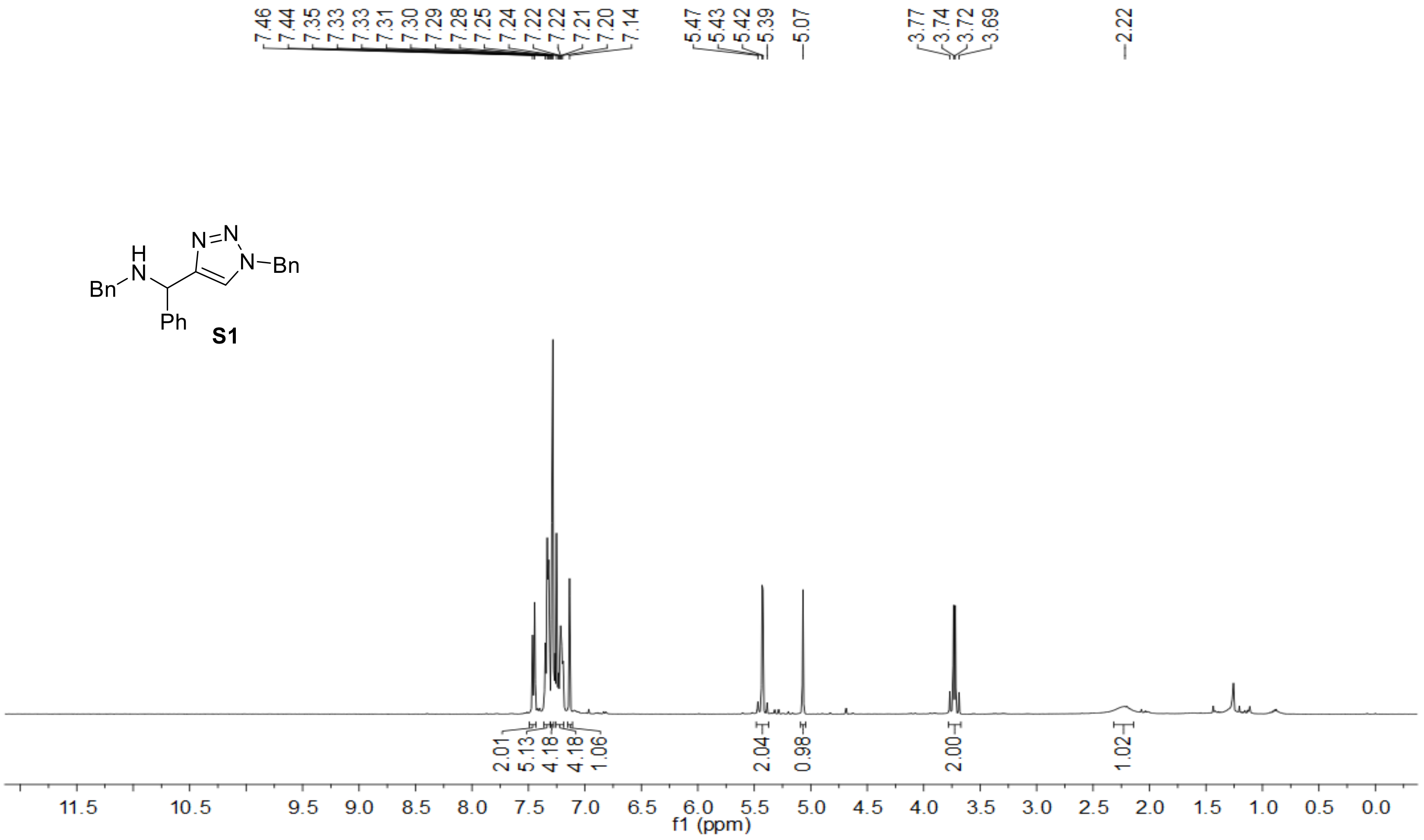

Supplementary Figure 122. ^{1}H NMR spectrum of *N*-benzyl-1-(1-benzyl-1*H*-1,2,3-triazol-4-yl)-1-phenylmethanamine (**S1**) in $CDCl_3$ (400 MHz) at 23℃.

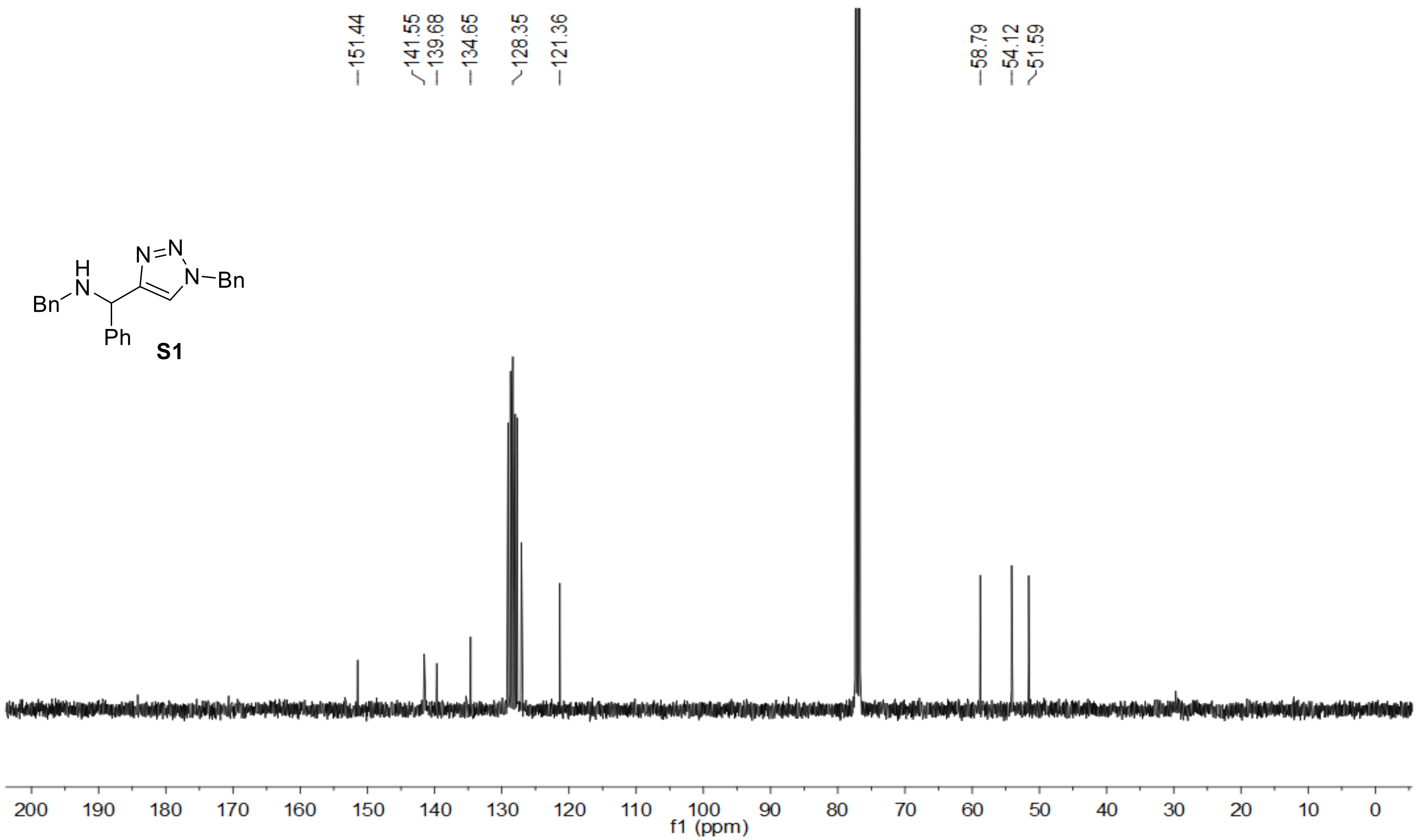

Supplementary Figure 123. ^{13}C NMR spectrum of *N*-benzyl-1-(1-benzyl-1*H*-1,2,3-triazol-4-yl)-1-phenylmethanamine (**S1**) in $CDCl_3$ (150 MHz) at 23℃.

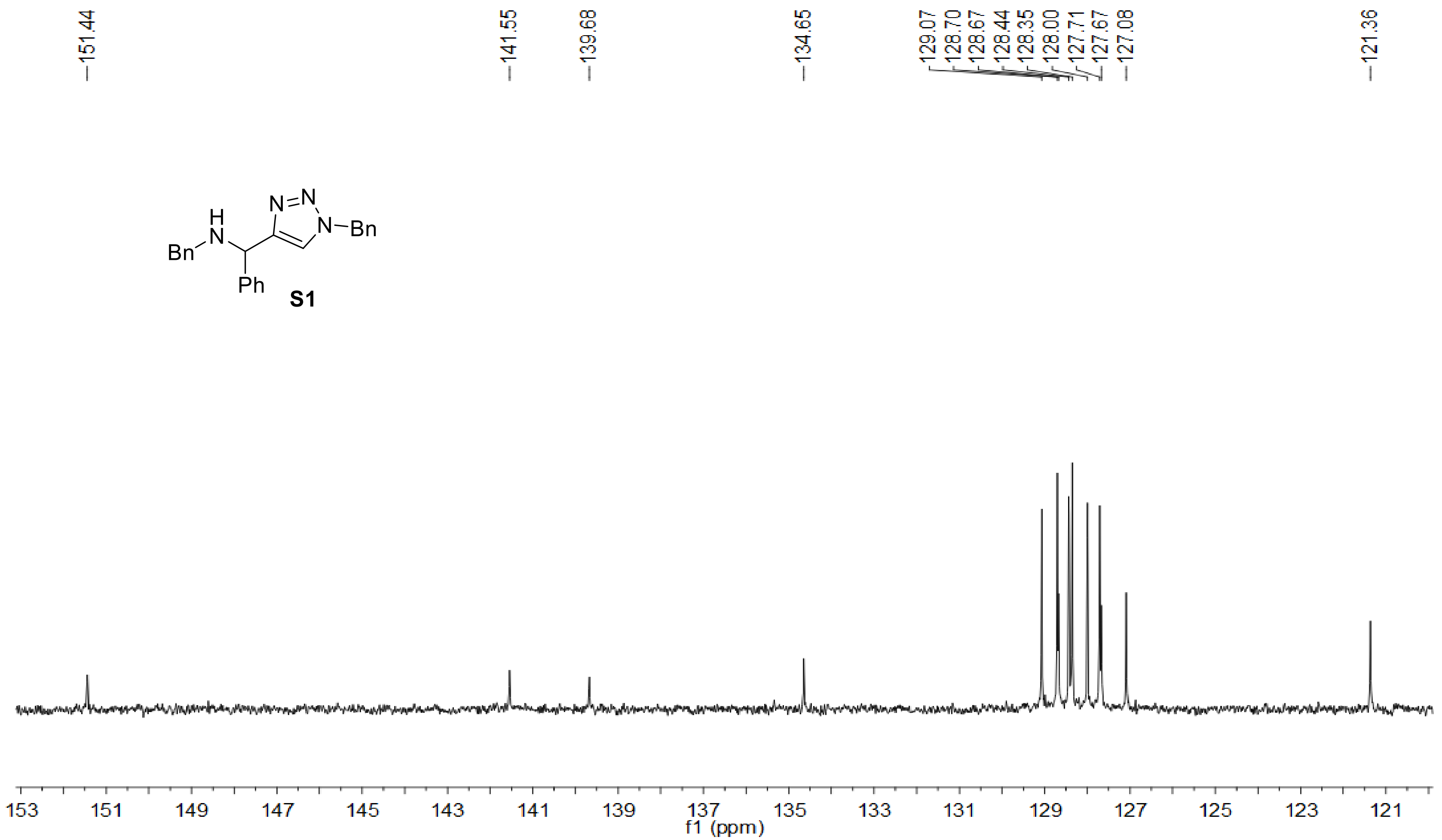

Supplementary Figure 124. Local magnification ^{13}C NMR spectrum of *N*-benzyl-1-(1-benzyl-1*H*-1,2,3-triazol-4-yl)-1-phenylmethanamine (**S1**) in $CDCl_3$ (150 MHz) at 23℃.

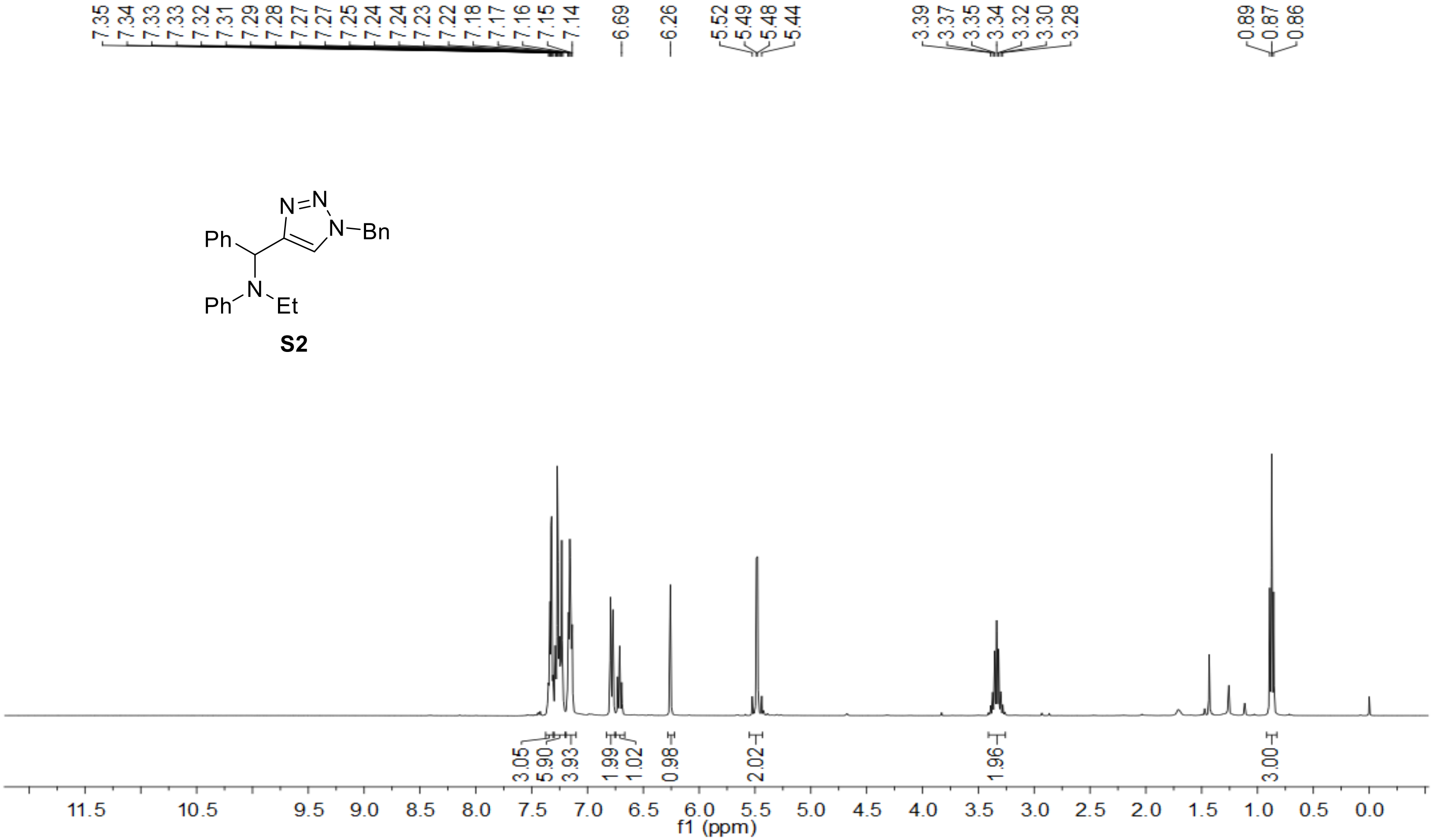

Supplementary Figure 125. ^{1}H NMR spectrum of *N*-((1-benzyl-1*H*-1,2,3-triazol-4-yl)(phenyl)methyl)-*N*-ethylaniline (**S2**) in $CDCl_3$ (400 MHz) at 23℃.

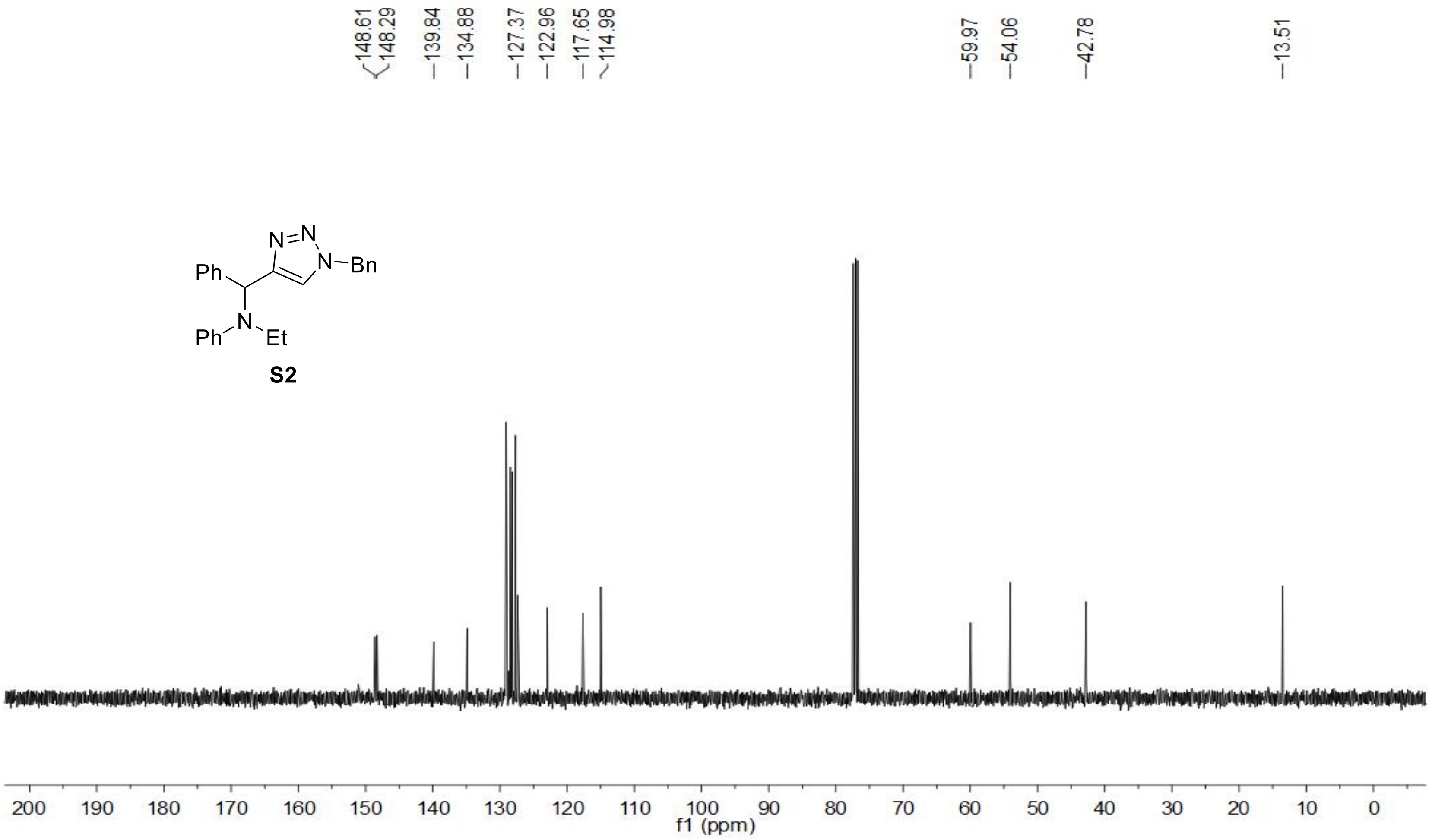

Supplementary Figure 126. ^{13}C NMR spectrum of *N*-((1-benzyl-1*H*-1,2,3-triazol-4-yl)(phenyl)methyl)-*N*-ethylaniline (**S2**) in $CDCl_3$ (100 MHz) at 23℃.

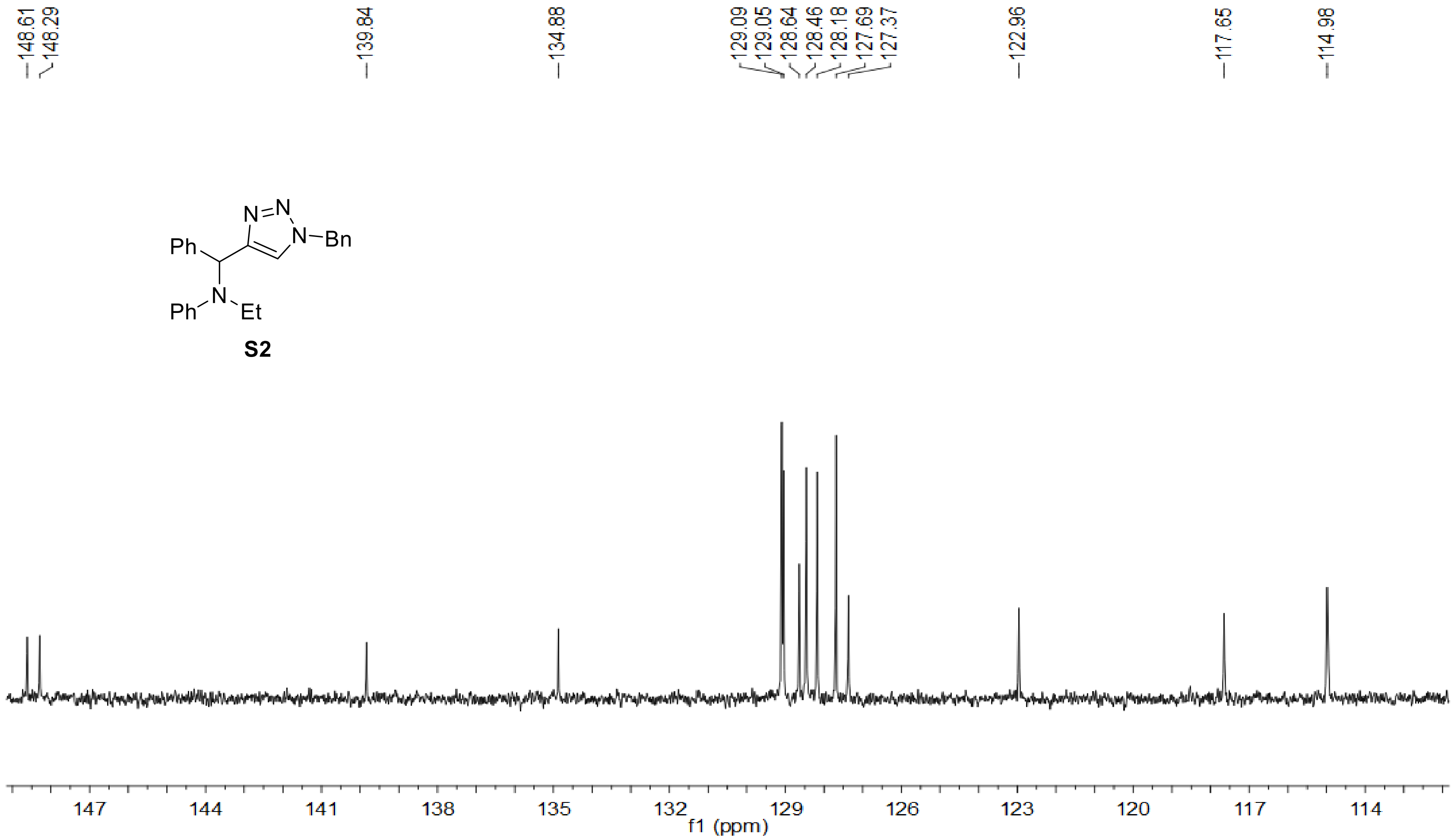

Supplementary Figure 127. Local magnification ^{13}C NMR spectrum of *N*-((1-benzyl-1*H*-1,2,3-triazol-4-yl)(phenyl)methyl)-*N*-ethylaniline (**S2**) in $CDCl_3$ (100 MHz) at 23℃.

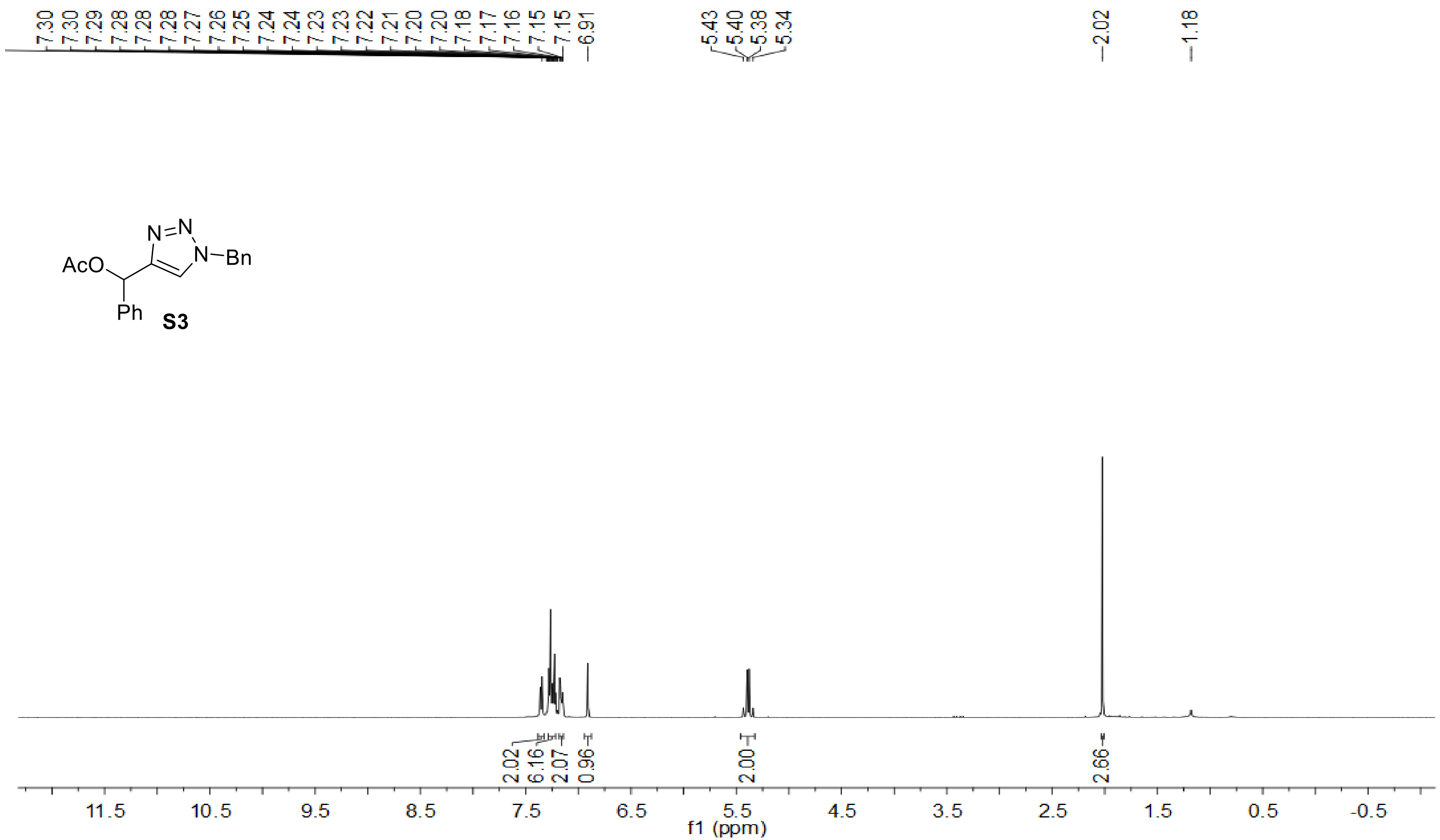

Supplementary Figure 128. ^{1}H NMR spectrum of (1-benzyl-1*H*-1,2,3-triazol-4-yl)(phenyl)methyl acetate (**S3**) in $CDCl_3$ (400 MHz) at 23°C.

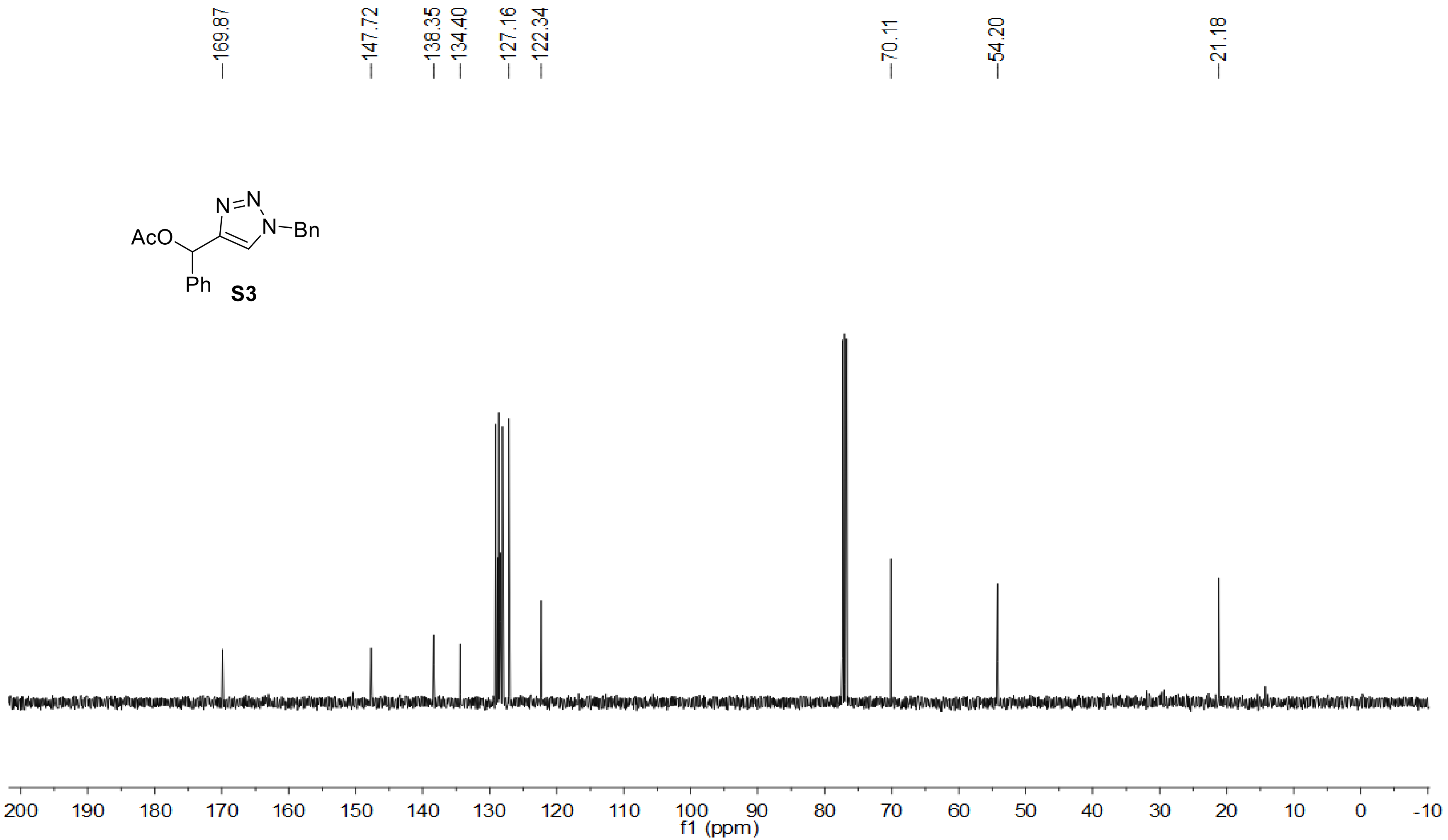

Supplementary Figure 129. ^{13}C NMR spectrum of (1-benzyl-1*H*-1,2,3-triazol-4-yl)(phenyl)methyl acetate (**S3**) in $CDCl_3$ (100 MHz) at 23℃.

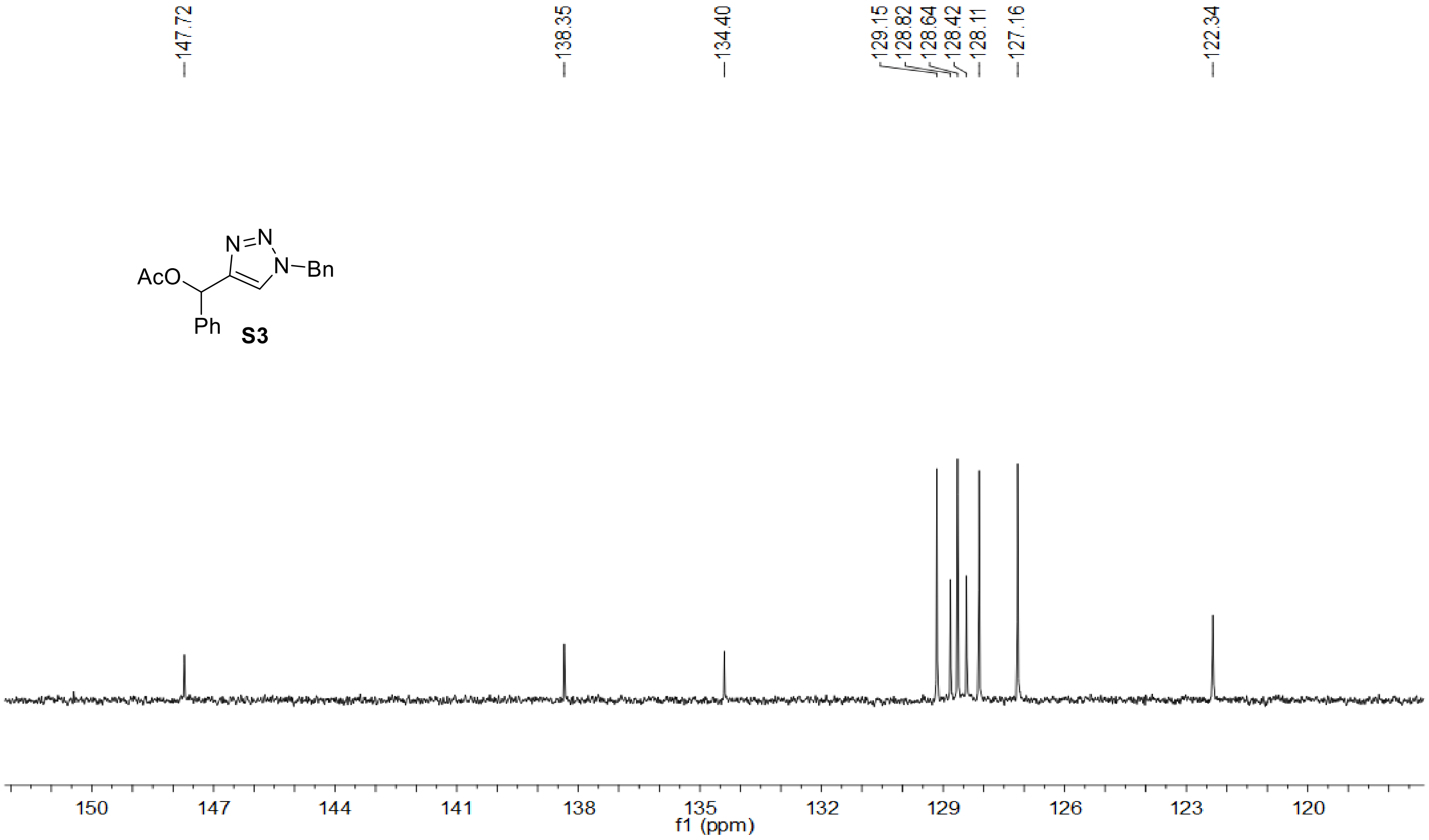

Supplementary Figure 130. Local magnification ^{13}C NMR spectrum of (1-benzyl-1*H*-1,2,3-triazol-4-yl)(phenyl)methyl acetate (**S3**) in $CDCl_3$ (100 MHz) at 23℃.

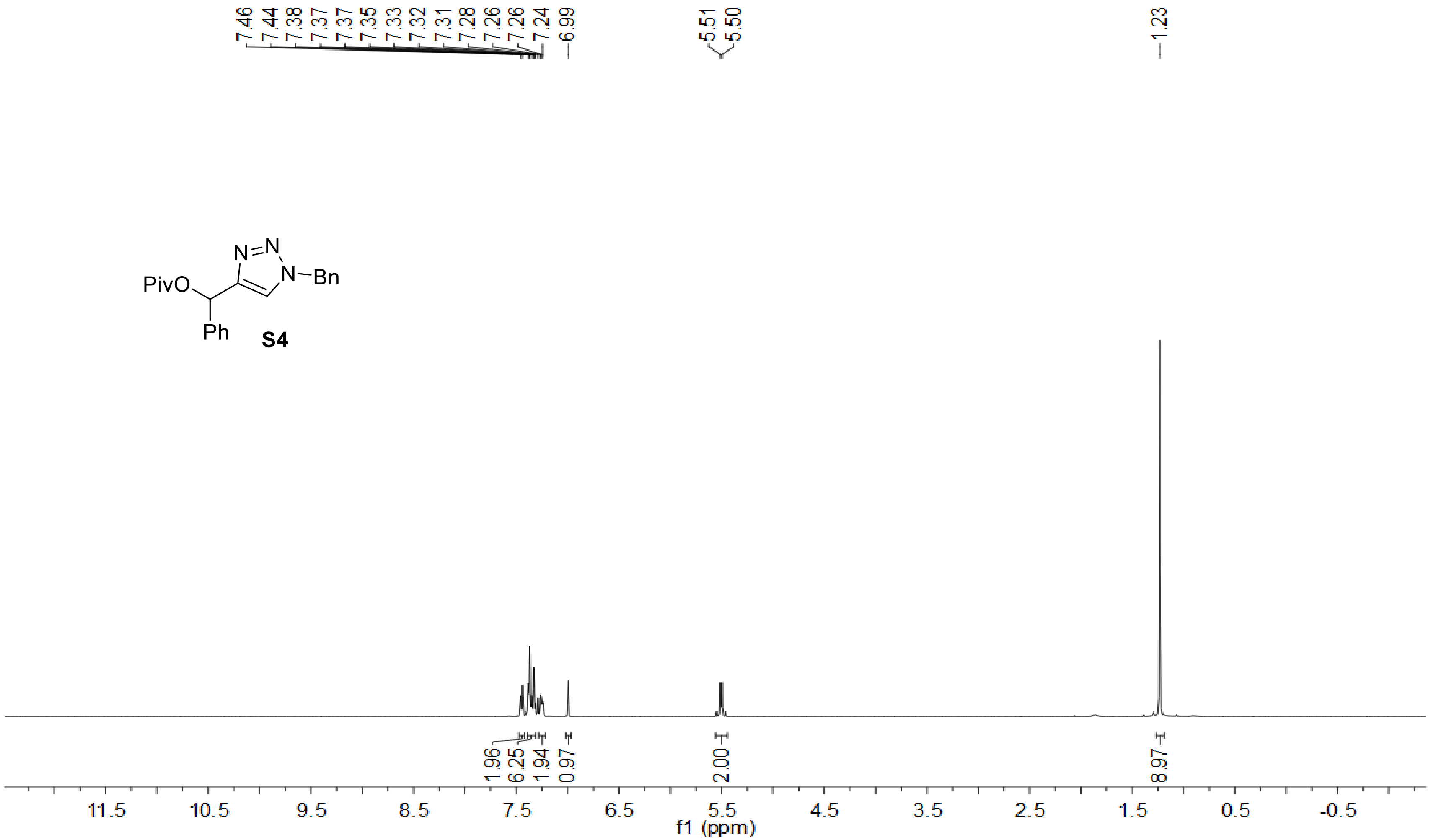

Supplementary Figure 131. ^{1}H NMR spectrum of (1-benzyl-1*H*-1,2,3-triazol-4-yl)(phenyl)methyl pivalate (**S4**) in $CDCl_3$ (400 MHz) at 23°C.

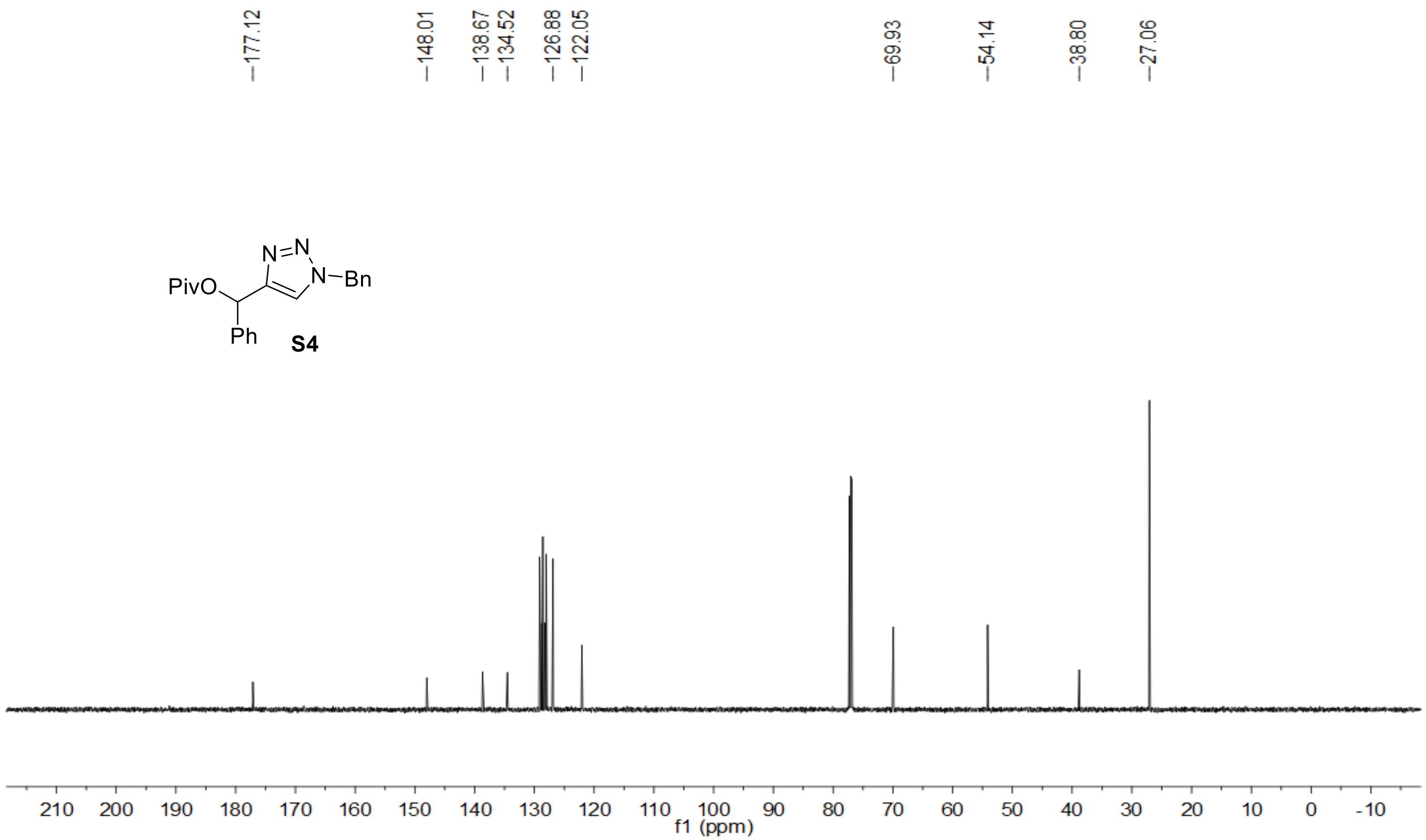

Supplementary Figure 132. ^{13}C NMR spectrum of (1-benzyl-1*H*-1,2,3-triazol-4-yl)(phenyl)methyl pivalate (**S4**) in $CDCl_3$ (100 MHz) at 23℃.

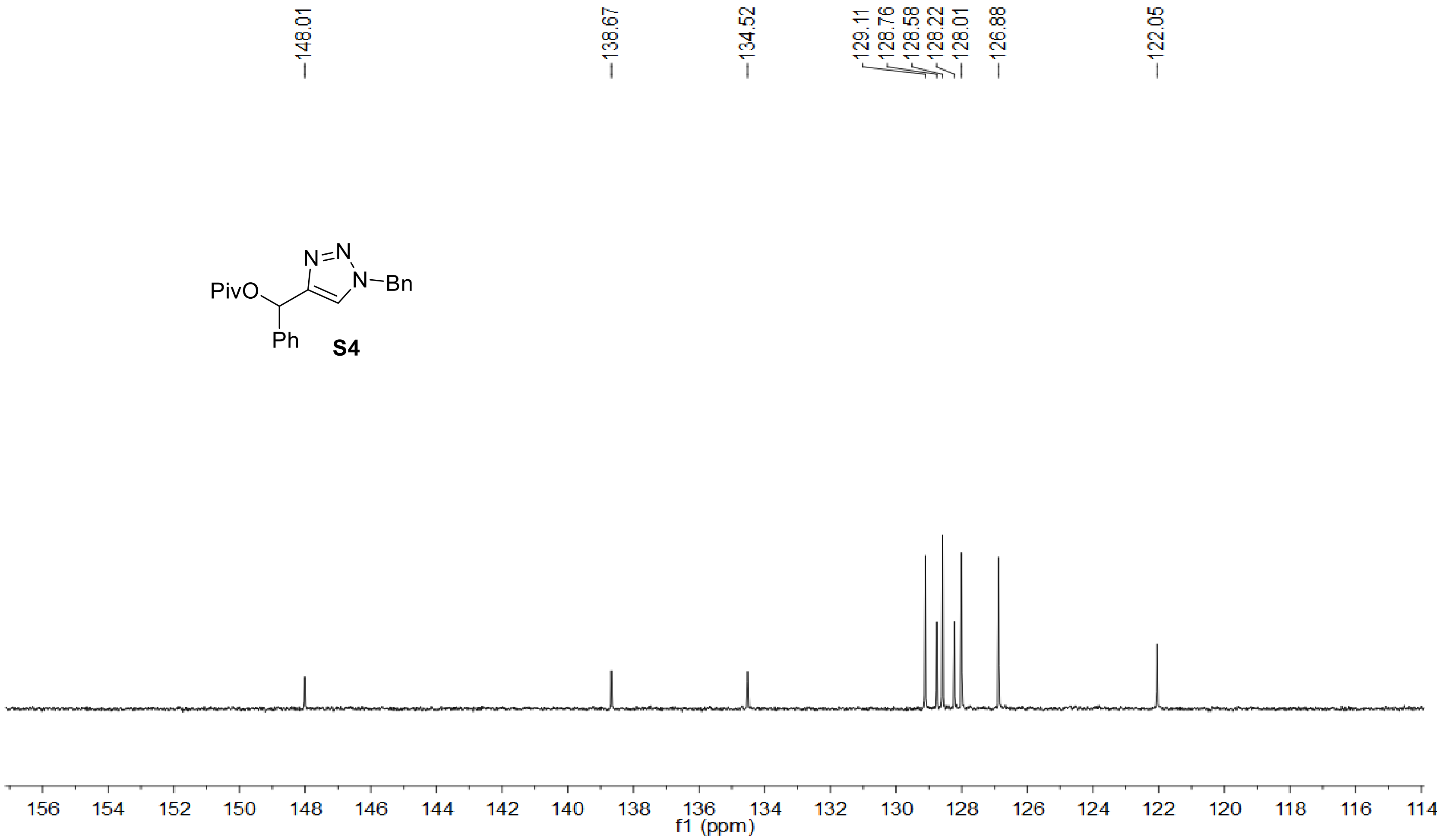

Supplementary Figure 130. Local magnification ^{13}C NMR spectrum of (1-benzyl-1*H*-1,2,3-triazol-4-yl)(phenyl)methyl pivalate (**S4**) in $CDCl_3$ (100 MHz) at 23℃.

4 Supplementary References

1 Chassaing, S.; Kueny-Stotz, M.; Isorez, G.; Brouillard, R. Rapid Preparation of 3-Deoxyanthocyanidins and Novel Dicationic Derivatives: New Insight into an Old Procedure. *Eur. J. Org. Chem*. **2007**, *15*, 2438.

2 Shen, R.; Luo, B.; Yang, J.; Zhang, L.; Han, L.-B. Convenient Synthesis of Allenylphosphoryl Compounds via Cu-Catalysed Couplings of P(O)H Compounds with Propargyl Acetates. Chem. Commun. **2016**, *52*, 6451.

3 Frisch, M. J. et al. Gaussian 16, Revision A.03, Gaussian, Inc., Walling ford CT, 2016.

4 Kojima, S.; Hidaka, T.; Ohba, Y. Synthesis of β-Monosubstituted α,β-Unsaturated Amides with *Z*-Selectivity Using Diphenylphosphonoacetamides. *Heteroatom Chem*. **2004**, *15*, 515.

5 Ueda, S.; Okada, T.; Nagasawa, H. Oxindole Synthesis by Palladium-Catalyzed Aromatic C-H Alkenylation. *Chem. Commun.* **2010**, *46*, 2462.

6 Lee, H.; Cho, S.; Lee, Y.; Jung, B. Stereoselective Formal Hydroamidation of Si-Substituted Arylacetylenes with DIBAL-H and Isocyanates: Synthesis of (*E*)- and (*Z*)-α-Silyl-α,β-unsaturated Amides. *J. Org. Chem*. **2020**, *85*, 12024.
