# Supplementary material for: Copper-catalyzed Z-selective synthesis of acrylamides and polyacrylamides via alkylidene ketenimines
Source: Nat Commun. 2022 Jul 27;13:4362. doi: 10.1038/s41467-022-32082-w (PMC9329291; doi:10.1038/s41467-022-32082-w)
Supplement: Supplementary file 3 — Description of Additional Supplementary Files [file 41467_2022_32082_MOESM3_ESM.docx]

**Description of Additional Supplementary Files**

File Name: Supplementary Data 1

Description: Coordinate data set for DFT optimized geometry of **3a’**

File Name: Supplementary Data 2

Description: Coordinate data set for DFT optimized geometry of **3a”**
